# A new cynodont from the Upper Triassic Los Colorados Formation (Argentina, South America) reveals a novel paleobiogeographic context for mammalian ancestors

**DOI:** 10.1038/s41598-022-10486-4

**Published:** 2022-04-25

**Authors:** L. C. Gaetano, F. Abdala, F. D. Seoane, A. Tartaglione, M. Schulz, A. Otero, J. M. Leardi, C. Apaldetti, V. Krapovickas, E. Steimbach

**Affiliations:** 1 Instituto de Estudios Andinos “Don Pablo Groeber” (IDEAN, UBA-CONICET), C1428EGA Ciudad Autónoma de Buenos Aires, Argentina; 2 Evolutionary Studies Institute, University of the Witwatersrand, WITS, Johannesburg, 2050 South Africa; 3 Unidad Ejecutora Lillo, CONICET-Fundación Miguel Lillo, T4000JFE San Miguel de Tucumán, Argentina; 4 Forschungs-Neutronenquelle Heinz Maier-Leibnitz (FRM II), Technische Universität München, 85747 Garching, Germany; 5 División Paleontología de Vertebrados (Anexo Laboratorios), Facultad de Ciencias Naturales Y Museo, Universidad Nacional de La Plata, B1900AVW La Plata, Argentina; 6 Departamento de Biodiversidad y Biología Experimental, Facultad de Ciencias Exactas y Naturales, Universidad de Buenos Aires, C1428EGA Ciudad Autónoma de Buenos Aires, Argentina; 7 Instituto y Museo de Ciencias Naturales, Universidad Nacional de San Juan, J5400DNQ San Juan, Argentina

**Keywords:** Palaeontology, Palaeontology

## Abstract

Probainognathia is a derived lineage of cynodonts which encompass Mammalia as their crown-group. The rich record of probainognathians from the Carnian of Argentina contrasts with their Norian representation, with only one named species. Here we describe a new probainognathian, *Tessellatia bonapartei* gen. et sp. nov., from the Norian Los Colorados Formation of the Ischigualasto-Villa Unión Basin of Argentina. The new taxon, represented by a partial cranium with associated lower jaws, was analyzed through neutron and X-rays micro-tomography (μCT). The high-resolution neutron μCT data allowed the identification of a unique character combination, including features inaccessible through traditional techniques. We constructed the largest phylogenetic data matrix of non-mammalian cynodonts. The new species and its sister taxon, the Brazilian *Therioherpeton cargnini*, are recovered as probainognathians, closely related to Mammaliamorpha. We conducted the first quantitative paleobiogeographic analysis of non-mammalian cynodonts, focusing in probainognathians. The results indicate that Probainognathia and Mammaliamorpha originated in southwestern Gondwana (in the Brazilian Paraná Basin), which was an important center of diversification during the Triassic. Finally, the Chinese Lufeng Basin is identified as the ancestral area of Mammaliaformes. These new findings, besides adding to the knowledge of the poorly represented Norian cynodonts from the Los Colorados Formation, are significant to improve our understanding of probainognathian diversity, evolution, and paleobiogeographic history.

## Introduction

Probainognathia (Synapsida: Cynodontia) is one of the two main clades of derived cynodonts (Eucynodontia), represented today by extant Mammalia^1,2^. With the exception of the highly-specialized, herbivorous tritylodontids, non-mammaliaform probainognathians are mostly small- to medium-sized faunivorous forms. The oldest probainognathian remains are known from Middle Triassic deposits from Gondwana. After an initial diversification during the early Late Triassic (Carnian), basal (non-mammaliaform) probainognathians are also found in Norian, Rhaetian, and Jurassic-to-Cretaceous deposits^3^. The abundant Carnian record of the lineage in Argentina is in strong contrast with their poor Norian representation. Among the six Norian probainognathian species previously recognized from Gondwana, four were reported from Brazil, one from Argentina, and one from southern Africa (but see Supplementary Information); most of them only represented by a single or a few fragmentary specimens. This is in concordance with the general scarcity of cynodonts from Norian strata globally^3,4^ (see Supplementary Information).

The Norian Los Colorados Formation of the Ischigualasto-Villa Unión Basin of Argentina is renowned for its vertebrate fossil record^5,6^. One of the youngest dicynodonts of Gondwana, *Jachaleria colorata*, is represented in the lower levels of the unit^7–9^. The upper levels of the Los Colorados Formation represent a different faunal assemblage ^10^ that has provided one of the oldest turtles^11,12^; basal and derived representatives of the crocodylian lineage^5,13–16^; and a number of relatively well-represented dinosaurs^5,17–22^. The latter is key to the understanding of the rise of this group as it is the earliest assemblage where dinosaurs are dominant components of the ecosystem^23^. Non-mammaliaform cynodonts (NMC) are scarcely represented in this unit^14,24,25^. Only two partial skulls of *Chaliminia musteloides*^24,25^ and a few fragmentary postcranial elements of an unnamed taxon^5,14,26^ have been reported. They are among the oldest tritheledontids, a lineage of mainly Gondwanan cynodonts that have been proposed to be closely related to basal mammals^25,27–29^. Since 2014, renewed exploration efforts in the Los Colorados Formation led to new findings at the Parque Nacional Talampaya (La Rioja Province, north-western Argentina), including five cynodont specimens whose preliminary study suggests that at least three of them might represent previously unrecognized taxa^30^. Recent geochronological studies of the unit suggest that the richest and most renowned faunal assemblage (‘La Esquina local fauna’), found in the upper levels of Los Colorados Formation, might be mid-Norian (~ 220–211 Ma) in age^31,32^.

**Figure 1:**
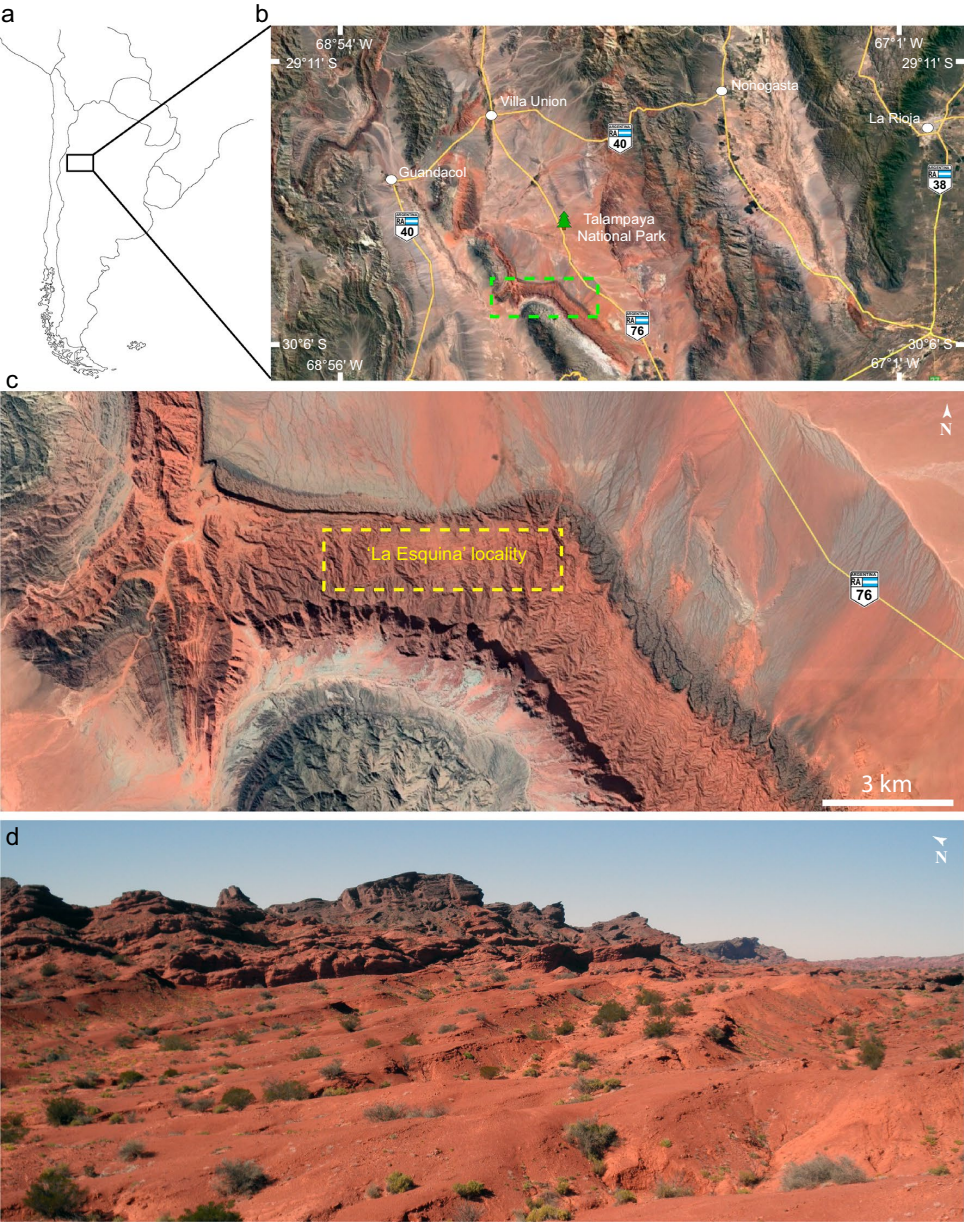
Geographic location of the ‘La Esquina’ locality where *Tessellatia bonapartei* holotype (PULR-V121) was found. (**a**) Southern South America; (**b**) satellite image of north-western Argentina depicting the study area (green, dotted-line rectangle) within the Talampaya National Park, La Rioja Province; (**c**) satellite image showing the main outcrops of the Los Colorados Formation where the rich faunal assemblage of the ‘La Esquina’ locality was found (yellow, dotted-line rectangle); (**d**) photograph of the outcrops at the ‘La Esquina’ locality portraying the levels from where *Tessellatia bonapartei* holotype (PULR-V121) was recovered. Map drawn with Adobe Illustrator CC 18 (https://www.adobe.com/products/illustrator.html). Satellite images from Google Earth, accessed December 2021.

Herein we describe a new species of derived NMC found in the upper levels of the Los Colorados Formation (Fig. 1) represented by a partial cranium with associated lower jaws. The specimen was analyzed through neutron and X-rays micro-tomography (μCT). Although only rarely used to analyze fossil specimens, it has become clear that neutron μCT complements X-ray μCT, including circumventing some problems that might arise from the latter methodology depending on the characteristics of the fossil sample studied^33,34^. In addition, we constructed the most comprehensive data-matrix for non-mammalian cynodonts published to date, allowing us to analyze the phylogenetic relationships of the new species and the main hypotheses regarding the ancestry of mammaliaforms and their close relatives. We also present the first quantitative paleobiogeographic analysis of non-mammalian cynodonts, focusing in probainognathians. Our results provide new insights regarding the evolutionary and paleobiogeographic history of Probainognathia. In particular, the new finding improves our knowledge on the diversity of the poorly represented Norian forms in the rich fauna of the Los Colorados Formation, northwestern Argentina.

## Results

SYSTEMATIC PALEONTOLOGY

THERAPSIDA^35^

CYNODONTIA^36^

EUCYNODONTIA^37^

PROBAINOGNATHIA^38^

*Tessellatia* gen. nov.

**Type species.** *Tessellatia bonapartei.*

**Etymology.** From the Latin *tessella* (each one of the tiles composing a mosaic), in reference to the combination of basal and derived features recognized in this taxon.

**Diagnosis.** Same as for species.

**Figure 2:**
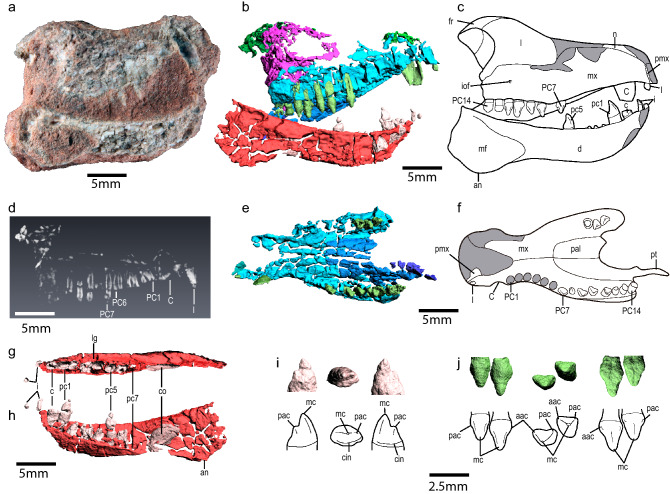
The holotype of *Tessellatia bonapartei,* gen. et sp. nov. (PULR-V121). (**a**) Cranium as preserved in right lateral view; (**b**) 3D model of the cranium in right lateral view; (**c**) interpretation line drawing of the skull and right lower jaw (restored to its natural position) in right lateral view; (**d**) digital render of the skull showing the roots of the anterior upper postcanines, not visible externally; (**e**) 3D model of the osseous secondary palate and upper teeth in ventral view; (**f**) interpretation line drawing of the osseous secondary palate and upper teeth in ventral view; (**g**) right lower jaw in dorsal view; (**h**) right lower jaw in medial view; (**i**) 3D model of the pc4 in labial, occlusal and lingual views (from left to right); (**j**) 3D model of PC9 and PC10 in labial, occlusal and lingual views (from left to right). *aac* anterior accessory cusp, *an* angular process, *C*/*c* upper/lower canines, *cin* cingulum, *co* coronoid, *d* dentary, *fr* frontal, *I*/*i* upper/lower incisors, *iof* infraorbital foramen, *l* lacrimal, *mc* main cusp, *mf* masseteric fossa, *mx* maxilla, *n* nasal, *pac* posterior accessory cusp, *pal* palatine, *PC*/*pc* upper/lower postcanines, *pmx* premaxilla, *pt* pterygoid. Scale bars a-h: 5 mm; i-j: 2.5 mm.

*Tessellatia bonapartei* sp. nov. (Fig. 2).

**Etymology.** In honor to the late Dr. José F. Bonaparte, who worked unrelentingly to broaden our knowledge of Mesozoic ecosystems and described the first cynodont remains from the Los Colorados Formation.

**Holotype.** PULR-V121, partial cranium, represented by the snout and orbital region, with associated lower jaws.

**Locality and horizon.** Upper third of the Los Colorados Formation, Talampaya National Park, La Rioja Province, Argentina. The specimen was found in a massive- to parallel-laminated sandy mudstone interval locally interbedded with parallel- to rippled- laminated sandstone, representing deposition in a floodplain setting sporadically affected by sandy splays from the fluvial channels. See Supplementary Information online for further details on the geological framework.

**Diagnosis.** Small probainognathian with the antorbital region notably longer than the height of the skull at the level of the anterior margin of the orbit. Autapomorphies of *Tessellatia bonapartei* are: masseteric fossa and low coronoid process posterior to the lower tooth row; angular process ventrally projected, well-developed, and semicircular in outline; broad groove between the lateral wall of the dentary and the postcanine alveoli; and upper postcanine count larger (14) than the lower postcanines (7), with the last six upper postcanines lacking an opposing lower tooth. Differs from other non-mammaliaform cynodonts except *Riograndia guaibensis* and the early mammaliaform *Morganucodon* in the presence of a large upper canine and a small lower canine. Differs from other non-mammaliaform probainognathians (except *Aleodon cromptoni*, *Prozostrodon brasiliensis*, *Pseudotherium argentinus*, and *Tritylodon longaevus*) in the subequal contribution to the secondary bony palate of the maxilla and palatine. Shares with many derived non-tritylodontid probainognathians and the mammaliaform *Morganucodon* an anteriorly elevated alveolar margin of the dentary. Shares with some derived probainognathians traditionally grouped in Tritheledontidae (*Chaliminia musteloides*, *Elliotherium kersteni*, *Irajatherium hernandezi*, and *Pachygenelus monus*), the presence of a ventrally bowed bony secondary palate with deep, narrow, lateral groove for the lower postcanines; posterior upper postcanines with convex labial and concave lingual surfaces, and bearing a centrally placed, symmetrical main cusp with convex mesial and distal margins flanked by smaller, lingually placed accessory cusps; and lower postcanines, comparatively larger than uppers, with a large, asymmetrical, mesial main cusp followed by smaller distal accessory cusps. Differs from *Chaliminia musteloides*, the only other named cynodont known from the Los Colorados Formation, in having the alveolar margin of the maxilla sigmoidal, with its dorsal-most point at the level of the canine; the horizontal ramus of the dentary comparatively low and lacking a well-developed platform lateral to the posterior postcanines; and the posteriormost lower incisor smaller than the canines and postcanines.

### Anatomical remarks

Selected traits are discussed below in a comparative framework. Relevant references of the taxa considered are provided in Supplementary Table S2. A complete anatomical description of *Tessellatia bonapartei* and further comparisons are provided in the Supplementary Information.

#### Skull

The bones of the skull are fragmentary, including portions of the right premaxilla, maxillae, right nasal, frontals, lacrimals, palatines, and pterygoids (Fig. 2). Two elements are too incomplete to be confidently determined, but they might represent parts of the basisphenoid and prootic (Supplementary Figs. S3–S4). The skull is relatively high and inflated immediately anterior to the orbits, at the level of the lacrimals. The snout of *Tessellatia bonapartei* is constricted posterior to the canines, resulting in an expanded rostrum anteriorly (Fig. 2e–f), a condition similar to that of *Riograndia guaibensis*, *Irajatherium hernandezi*, *Pachygenelus monus*, *Prozostrodon brasiliensis*, *Pseudotherium argentinus*, and *Brasilodon tetragonus* (but not *Therioherpeton cargnini*, *Elliotherium kersteni*, and *Chaliminia musteloides*) among derived non-tritylodontid probainognathians (NTP).

**Figure 3:**
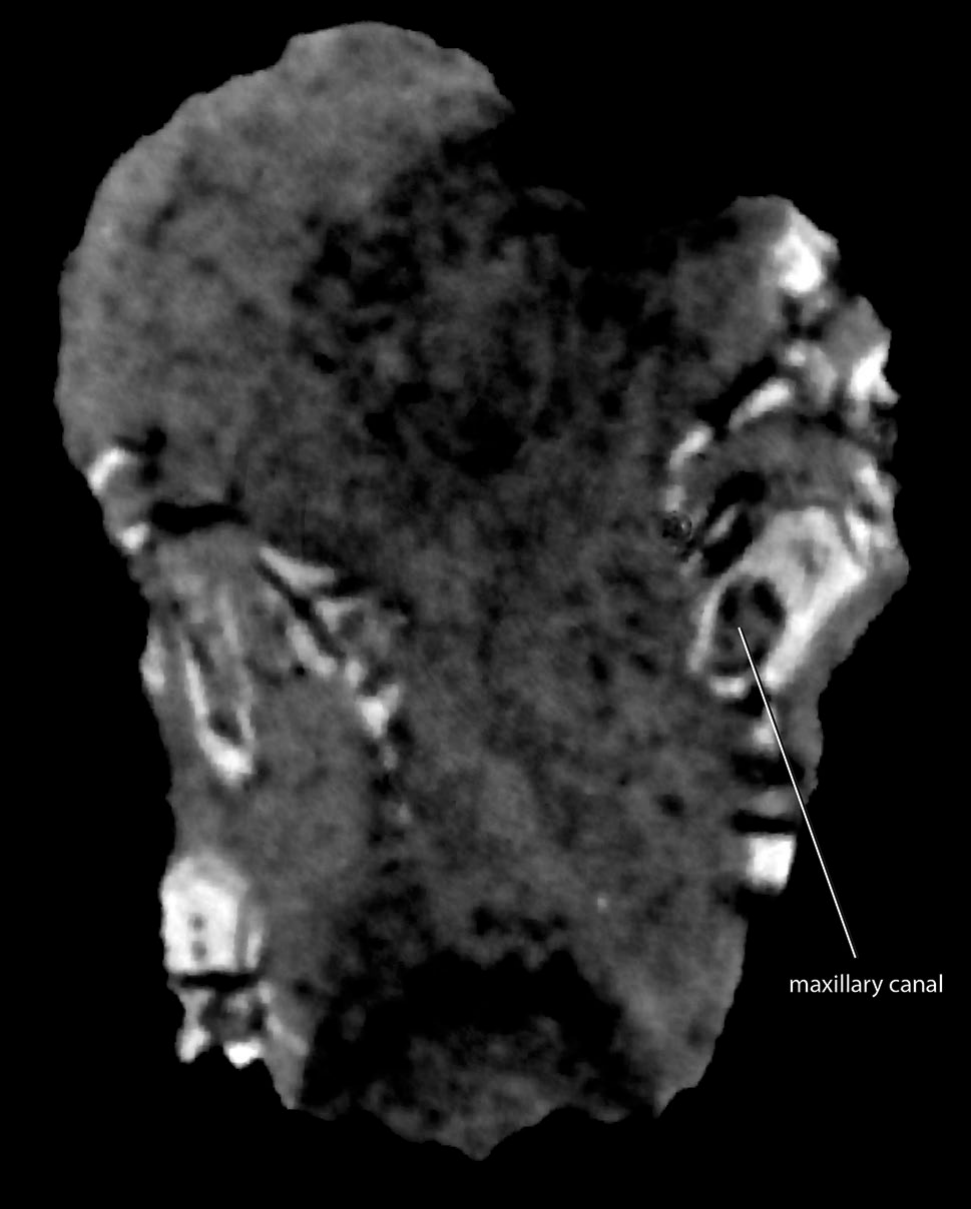
The holotype of *Tessellatia bonapartei*, gen. et sp. nov. (PULR-V121). Neutron tomography image of a transversal cross-section of the cranium showing the ossified maxillary canal.

The alveolar margin of the maxilla has a sigmoidal outline in lateral view (Fig. 2b–c). The maxillary canal in *Tessellatia bonapartei* is completely ossified (Fig. 3) and separated from the maxillary sinus, as in derived probainognathians including mammaliaforms. The short infraorbital canal and posteriorly placed infraorbital foramen of *Tessellatia bonapartei* (Fig. 2c; infraorbital foramen 2 in^39^; see ^40^), is shared with derived probainognathians, suggesting the presence of a mobile rhinarium with sensitive vibrissae (see^40,41^).

A remarkably large facial exposure of the lacrimal (Fig. 2b, c) is a distinctive feature of *Tessellatia bonapartei* that, among probainognathians, is only recognized in some tritylodontids. Unlike in other probainognathians, *Tessellatia bonapartei* shares with its sister-group *Therioherpeton kersteni*, some tritylodontids, and *Morganucodon*, a short lacrimal-frontal contact and frontals with short anterolateral projections.

*Tessellatia bonapartei* is more comparable to early-diverging probainognathians in the presence of an orbital process of the palatine that is excluded from the orbital wall and not contacting the frontal. Unlike in derived NTP, in which the secondary palate extends to the end of the tooth row or posterior to it, the osseous secondary palate of *Tessellatia bonapartei* ends anteriorly to the antepenultimate upper postcanine (PC13; Fig. 2e, f). On the other hand, *Tessellatia bonapartei* shares with some derived probainognathians (e.g., *Chaliminia musteloides*, *Elliotherium kersteni*, *Irajatherium hernandezi*, and *Pachygenelus monus*), traditionally grouped in Tritheledontidae (see^25^), the presence of a ventrally bowed osseous secondary palate reaching the level of the crown of the upper postcanines (Fig. 2b, c). In these forms, deep, narrow grooves are present medial to the upper tooth rows, posterior to the maxilla-palatine suture (Fig. 2e, f).

#### Lower jaw

The dentary is relatively robust, high, and lateromedially wide (Fig. 2b, c, h). Unique to *Tessellatia bonapartei* is the presence of a broad groove between the lateral wall of the dentary and the postcanine alveoli (Fig. 2g). This groove starts at the posterior half of pc2 and ends laterally to the penultimate lower postcanine (pc6). The anterior region of the dentary is upwardly bent regarding the postcanine line in *Tessellatia bonapartei* (Fig. 2b, c, h) as well as in many derived NTP and the mammaliaform *Morganucodon*. The shape and development of the angular process of the dentary of *Tessellatia bonapartei* distinguishes it from other Triassic cynodonts.

#### Dentition

The upper dentition includes at least one incisor, one canine, and 14 postcanines (1 + ?I/1C/14PC) (Fig. 2b–f). The lower dentition includes one incisor preserved in situ in the dentary and an isolated fragment of another, one canine, and seven postcanines (2 + ?i/1c/7pc) (Fig. 2b, c, g, h). Incisors are small, non-procumbent teeth (Fig. 2b–h), unlike those of some tritheledontids (see^25^) such as *Chaliminia musteloides*, *Pachygenelus monus,* and *Riograndia guaibensis*. The presence of a large upper canine (1.5 or more the mesiodistal length of the first postcanine) in conjunction with a reduced lower one (the same mesiodistal length or less of the first postcanine) (Fig. 2b–h) is only recorded in *Tessellatia bonapartei* and *Riograndia guaibensis* among NMC and shared with the early mammaliaform *Morganucodon*. On the other hand, in *Prozostrodon brasiliensis*, *Pachygenelus monus*, and *Brasilodon tetragonus*, as well as in more basal probainognathians (*Trucidocynodon riograndensis*, *Probainognathus jenseni*, *Lumkuia fuzzi*, *Aleodon*, and *Chiniquodon*), both the upper and lower canines are enlarged. Both lower and upper canines are reduced in *Chaliminia*. The general structure of the postcanines of *Tessellatia bonapartei* (Fig. 2i, j) is similar to those of tritheledontids (see^25^). A reduced number of lower postcanines (7) compared to the number of upper ones (14) is a distinctive feature of *Tessellatia bonapartei* among NMC (Fig. 2b–h). It is noteworthy that the lower tooth row is very short when compared to the upper one. The last lower postcanine (pc7) would have occluded approximately between PC8 and PC9. In this scenario, the last five upper postcanines lacked a lower counterpart.

#### *Comparisons with* Chaliminia musteloides

*Chaliminia musteloides,* represented by cranial remains, is the only previously named cynodont from the Los Colorados Formation. The known specimens are comparable in size to *Tessellatia bonapartei*. Hence, we deem it important to highlight some additional differences of the published specimens of *Chaliminia musteloides* (the holotype PVL 3857 and the referred specimen PULR 088) with the holotype of *Tessellatia bonapartei*. *Tessellatia bonapartei* has a proportionally longer snout when compared to the height of the skull at the level of the anterior margin of the orbit (Supplementary Table S3). When compared to the holotype of *Chaliminia musteloides*, *Tessellatia bonapartei* has a comparatively slenderer dentary. On the other hand, the proportions of the horizontal ramus of the dentary are similar between *Tessellatia bonapartei* and the referred specimen of *Chaliminia musteloides* (PULR 081) (Supplementary Table S4). However, it is important to take into account that the anterior end of the skull and lower jaws are missing in *Tessellatia bonapartei*; a longer skull and dentary would mean a longer snout and a more slender horizontal ramus of the dentary, accentuating the differences in these proportions with the specimens of *Chaliminia*.

In addition, *Tessellatia bonapartei* (Fig. 2) differs from *Chaliminia musteloides* in the presence of a constriction in the snout posterior to the canines that results in an expanded rostrum anteriorly; a strongly concave posterior margin of the secondary palate; a notably short lower tooth row with the ascending coronoid process of the dentary well-posterior to the last lower postcanine, slightly sloping dorsally at the level of the orbit; the masseteric fossa does not reach the level of the last lower postcanine; a broad groove between the lateral wall of the dentary and the postcanine alveoli; and small non-procumbent lower incisors (Fig. 2). *Tessellatia bonapartei* lacks the strong osseous platform lateral to the last lower postcanines observed in the holotype of *Chaliminia musteloides.* The lower canine of *Tessellatia bonapartei* has approximately the same diameter than the first lower postcanine, as measured at the upper portion of the root, whereas the lower canine of *Chaliminia musteloides* is markedly larger than the postcanines. Unlike the condition of *Chaliminia musteloides*, there is no lower postcanine diastema in *Tessellatia bonapartei*.

Despite sharing the general morphology, there are some differences in the postcanine dentition between *Tessellatia bonapartei* and *Chaliminia musteloides*. Unlike the convex labial and concave lingual surface of the upper postcanines of *Tessellatia bonapartei*, in *Chaliminia musteloides* there is a central blunt crest separating mesial and distal depressions labially whereas the lingual surface is almost flat. Contrary to what is observed in *Tessellatia bonapartei*, the lower postcanines of *Chaliminia musteloides* are mesiodistally shorter than the upper ones. In *Chaliminia musteloides*, the lower postcanines show more conspicuous, higher distal accessory cusps, more closely placed to the main cusp than in *Tessellatia bonapartei*.

### Phylogenetic results

The parsimony analysis of 151 characters and 73 operational taxonomic units (OTUs) resulted in 29,304 most parsimonious trees (MPT) of 786 steps. The strict consensus is poorly resolved. There is a large polytomy including a wide array of taxa. A basal group of Cynodontia (represented by late Permian forms), Epicynodontia, and Eucynodontia, as usually conceived^2,28,42^, are not recovered. Only Cynognathia and a clade including some derived probainognathians are recovered by our analysis. Tritylodontidae, the only clade with strong support, is nested among the latter group.

**Figure 4:**
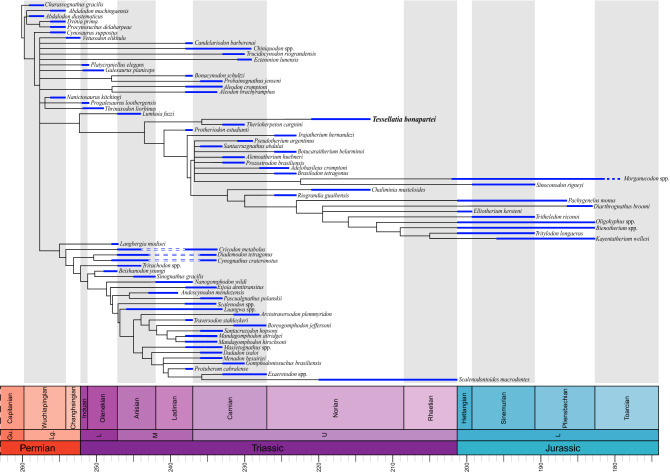
Equal length calibrated, strict consensus tree resulting from analysis of the pruned data matrix. The represented temporal extension of the taxa corresponds to that of the levels/units in which they are represented.

A second analysis excluding three wild-card taxa (i.e., *Diegocanis elegans*, *Charruodon tetracuspidatus*, and *Microconodon tenuirostris*) retrieved 200 MPT of 782 steps, whose strict consensus is better resolved than with the complete dataset (Fig. 4). A basal group of late Permian cynodonts is followed by *Cynosaurus suppostus*, a node that perhaps represents the Epicynodontia level. The next node is a large polytomy including several terminals. Traditional taxa of Probainognathia, such as *Chiniquodon* spp., *Trucidocynodon riograndensis*, *Ecteninion lunensis*, *Candelariodon barberenai*, *Probainognathus jenseni* + *Bonacynodon schultzi*, and *Aleodon cromptoni* + *Aleodon brachyrhamphus*, are recovered as part of the mentioned polytomy that also includes basal members of Epicynodontia. In any case, resolving this huge polytomy that includes Eucynodontia or obtaining a clade including all taxa traditionally considered Probainognathia is not our goal. Our aim is to evaluate the position of *Tessellatia bonapartei* among other probainognathian, which our matrix and methodology allows us to do. Cynognathia and Tritylodondidae form monophyletic groups as in the complete analysis. This is not the case for Tritheledontidae, whose members are found to belong to the Mammaliamorpha clade (as defined by^43^), as stem-taxa to Tritylodontidae or to Mammaliaformes. According to this, Mammaliamorpha includes tritylodontids, mammaliaforms, and a number of derived probainognathians. *Tessellatia bonapartei* is recovered as sister-group of the Brazilian *Therioherpeton cargnini* based on two unambiguous synapomorphies: axis of the posterior region of the maxillary tooth row directed towards the center of the subtemporal fossa (character 98:1) and transverse axis of crown strongly oblique to midline axis of the skull (character 114:1). The clade formed by *Tessellatia bonapartei* + *Therioherpeton cargnini* is in a basal position among the reduced-probainognathian clade, as the sister-group to the group including *Protheriodon estudianti* and Mammaliamorpha. The clade *Protheriodon estudianti* + Mammaliamorpha is supported by two unambiguous synapomorphies: a relatively long secondary palate (character 48:1; which is shorter in *Tessellatia bonapartei* and *Therioherpeton cargnini*) and snout longer than the temporal region (character 10:0; unknown in *Tessellatia bonapartei* and shorter in *Therioherpeton cargnini*). The absence of the prefrontal and postorbital bones (character 4:1 and 6:2), a slender zygomatic arch (character 17:0), a moderate lateral expansion of the braincase (character 28:1), a reduced lower canine (character 96:1), and a unilateral postcanine occlusion without forming a consistent pattern between upper and lower teeth (character 99:1) are unambiguous synapomorphies of the clade formed by *Tessellatia bonapartei* + *Therioherpeton cargnini* and Mammaliamorpha.

A traditional Probainognathia is portrayed in the majority rule consensus tree of the reduced, second analysis (Supplementary Fig. S5). It shows a basal polytomy integrated by *Candelariodon barberenai*, a chiniquodontid clade (*Chiniquodon* + *Aleodon*), and a clade with remaining probainognathians, which includes a polytomy formed by three clades: (*Bonacynodon schultzi* + *Probainognathus jenseni*), (*Trucidocynodon riograndensis* + *Ecteninion lunensis*), and the other probainognathians. In this topology, the placement of *Lumkuia fuzzi*, the oldest and only basal Probainognathia from South Africa, is counterintuitive, and different to previous phylogenies in which it was recovered as the basal-most Probainognathia (e.g.,^2,43^) or as the basal-most Eucynodontia, outside of Cynognathia and Probainognathia (e.g.^42^). In our majority rule tree, basal-most Probainognathia are represented by a group of largely South American Late Triassic taxa.

### Probainognathian paleobiogeography

A Bayesian Binary Markov Chain Monte Carlo (BBM) analysis of 100,000 cycles and 100 chains was performed, including nine a priori determined areas of provenance (Supplementary Table S1) and allowing the maximum number of areas per node. The study was based on the probainognathian clade as shown in the majority rule consensus tree (Supplementary Fig. S5) obtained from the analysis of the pruned dataset (70 OTUs, 151 characters). Additionally, we performed a Hausdorf’s^44^ manual optimization of Probainognathia, Mammaliamorpha, and Tritylodontidae nodes (Supplementary Tables S5, S6, S7, S8).

**Figure 5:**
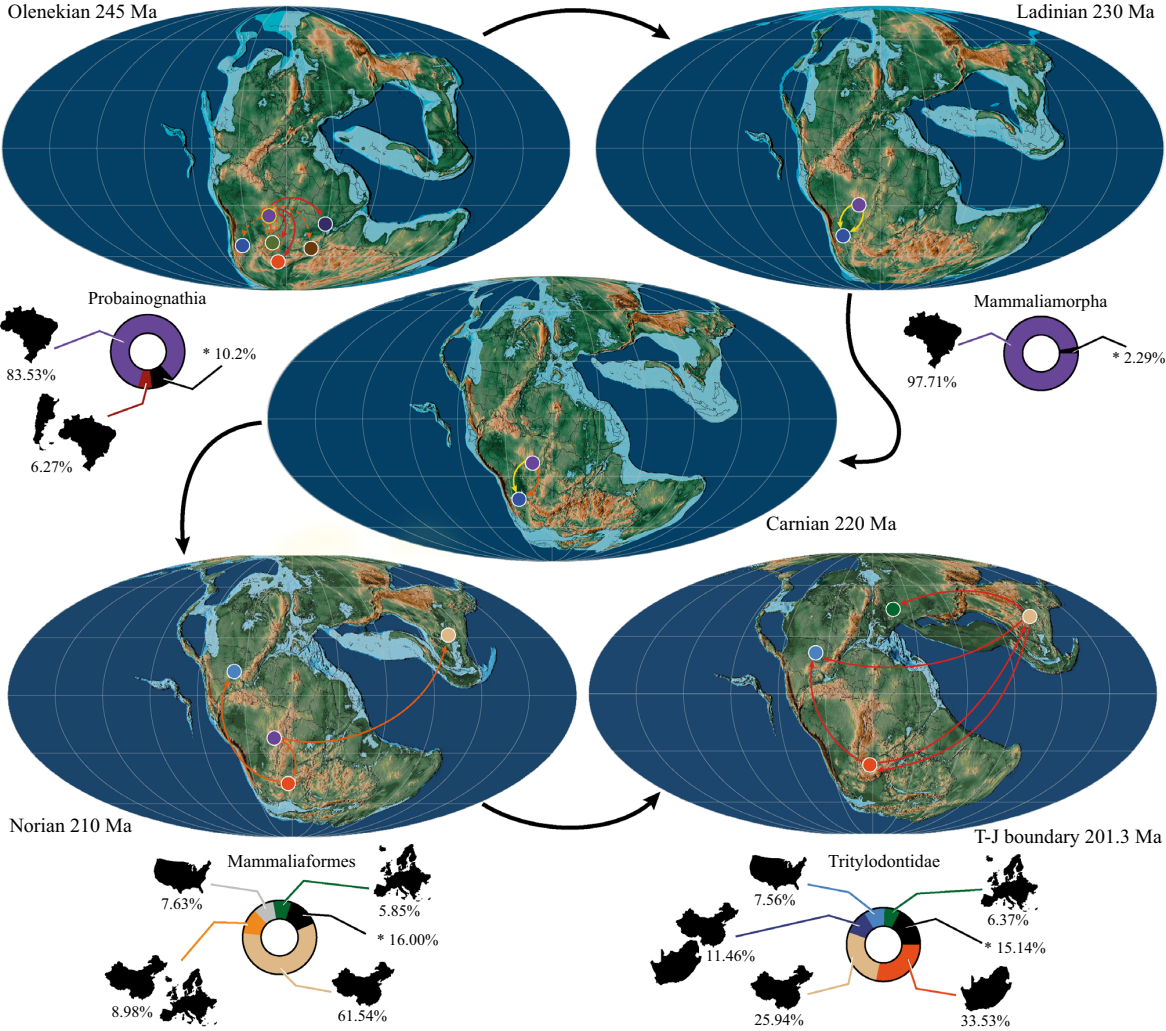
Paleogeographic reconstructions depicting dispersal events in different time-slices according to the RASP analysis and the time-calibrated majority rule consensus tree of the pruned data matrix. Areas are represented by circles: *violet* Paraná Basin (Brazil), *blue* Ischigualasto-Villa Unión Basin (Argentina), *musk green* Otiwarongo Basin (Namibia), *orange* Karoo Basin (South Africa), *dark brown* Morondava Basin (Madagascar), *dark blue* Ruhuhu Basin (Tanzania), *light blue* North American basins, *light brown* Lufeng Basin (China), and *dark green* European basins. Arrows in each time-slice represent dispersions at different moments of the corresponding age: *yellow* early, *orange* middle, and *red* late. Yellow-circled area in the paleogeographic reconstruction of the Olenekian time-slice represents that Probainognathia originated in the Paraná Basin (Brazil) by the early Olenekian. Dotted lines in the paleogeographic reconstruction of the Olenekian time-slice indicate that after the dispersion from the Paraná Basin (Brazil) to the Otiwarongo Basin (Namibia), a new joint area (Paraná- Otiwarongo basins) was established and from there dispersions occurred to the Ischigualasto-Villa Unión Basin (Argentina) and the Morondava Basin (Madagascar). Pie charts showing the probability of different ancestral areas for selected major clades. Asterisk depicts the probability of all those areas that have less than 5% probability of being the ancestral area of a node taken together. Paleogeographic reconstructions from^69^.

The results (Fig. 5) indicate that the paleobiogeographic history of probainognathians was dominated by dispersions (22 events) and sympatric speciation events (18 events), followed by vicariances (13 events) and with a single extinction event. Probainognathia originated in southwestern Gondwana (Paraná Basin, Brazil; 83.53%) and this was followed by a large and relatively quick increase in the diversity. This diversification event included the origin of Mammaliamorpha in this region (97.71%). Hausdorf’s optimization indicates the same results for Probainognathia and Mammaliamorpha nodes (Supplementary Tables S5−S6).

According to our results, mammaliamorphs thrived in the Paraná Basin and experimented a few dispersions to the Ischigualasto-Villa Unión Basin (Argentina; 2 events), the Lufeng Basin (China; 1 event), and to North American basins (1 event). It is in the Lufeng Basin where the origin of Mammaliaformes is expected (61.54%). However, it must be noted that we did not include *Gondwanadon tapani* and *Tikitherium copei*, from the upper Carnian-lower Norian Tiki Formation (Madhya Pradesh, India), in our analysis as they are both represented by an isolated tooth which is even incompletely preserved in the case of *Gondwanadon*^45–47^. Although never tested phylogenetically, *Gondwanadon* has been considered to be closely related to *Morganucodon*^48,49^. *Tikitherium*, originally interpreted to be related to *Morganucodon*, is now regarded as a docodontan, more closely related to the Rhaetian *Delsatia rhupotopi* and *Woutersia* spp. from France^50^. The Tiki Formation records the only faunal assemblage from the Triassic of Gondwana featuring remains of mammaliaforms (in fact, the oldest record of the group) and the early recognition of two mammaliaforms with clearly different tooth patterns suggests an undocumented worldwide evolution of this group. By the end of the Norian, dispersion events from the Paraná Basin to the Karoo Basin (South Africa) are identified. In this latter region, they diversified and dispersed to the Lufeng Basin (1 event) and to North American basins (1 event). The origin of Tritylodontidae is inferred to have occurred in the Karroo Basin (33.53%), whilst Hausdorf’s optimization indicates that the origin of this group occurred in the Lufeng Basin (Supplementary Table S7).

## Discussion

There is a rich Triassic record of cynodonts in the Ischigualasto-Villa Unión Basin of Argentina, particularly remarkable in the Carnian Chañares and Ischigualasto formations^51,52^. The representation of this group becomes extraordinarily scarce in the fossil-rich Los Colorados Formation (‘La Esquina local fauna’), the youngest Triassic unit of the basin, where only one species represented by two small skulls and a second putative small taxon known by a few fragmentary postcranial remains are known to date^14,24–26^. The finding of *Tessellatia bonapartei*, thus reinforces the knowledge of a subdued fauna of small animals in the Los Colorados Formation, only represented by cynodonts so far. *Tessellatia bonapartei* displays a mosaic of basal and derived features. Despite this, it is unambiguously recovered as a derived probainognathian. *Tessellatia bonapartei* bears a postcanine morphology very similar to that of taxa traditionally grouped in Tritheledontidae. Although this might point to a similar diet, the distinctive features in the dentition of *Tessellatia bonapartei* (e.g., a notably reduced number of lower postcanines regarding upper ones, large upper canine in conjunction with a small lower one, and small-sized incisors) suggest a unique feeding-habit.

The cynodonts from the Los Colorados Formation fill a temporal gap in the South American record of Probainognathia, between the early Norian forms from the Brazilian Caturrita Formation and the late Norian-Rhaetian, still undescribed, cynodonts from Quebrada del Barro Formation, San Juan, Argentina^8^. The faunal association from the Los Colorados Formation also contrasts with these latter South American assemblages because it is numerically dominated by large taxa (e.g., sauropodomorphs), with only a few known specimens of the rare small-sized cynodonts present (see Supplementary Information). On the other hand, small cynodonts are diverse and/or abundant in the Caturrita and Quebrada del Barro formations^8,53^.

Contrasting with the limited record of Probainognathia (and cynodonts) of the Los Colorados Formation, the upper Carnian-Norian record of the Santa Maria and Caturrita formations (Brazil) shows a burst of diversity of small derived probainognathians with at least eight species^4^. In fact, the Brazilian record of the group provides most of the information regarding Probainognathia during that time in the world. A second, very important pulse of diversification of this lineage is clearly headed by mammaliamorphs mostly from the Lower Jurassic. In this case, tritylodontids make the great difference because extreme modifications of the skull, dentition and, together with early mammaliaforms (i.e., *Morganucodon* and allies), of the postcranium. Tritylodontids were also abundant and diverse, with a cosmopolitan distribution, and the only cynodont lineage of this time reaching comparatively large body sizes.

Basal and derived probainognathians are recognized during the Carnian, including some relatively early-diverging forms that attained the largest body-masses of the Triassic representatives of the clade (e.g., *Chiniquodon*, *Trucidocynodon riograndensis*;^51,54^). During the Norian, only small-sized probainognathians are represented. This scenario suggests that the Carnian-Norian transition represented a pivotal moment for the diversity and ecology of cynodonts in general and probainognathians in particular, probably related to the Carnian Pluvial Episode^55^.

According to our phylogenetic results (Fig. 4), the two taxa from Los Colorados are not closely related. *Tessellatia bonapartei* is an early-diverging taxon whereas the comparatively more crownward positioned *Chaliminia musteloides* is more closely related to tritylodontids than to mammaliaforms. The calibrated phylogeny together with the quantitative paleobiogeographic analysis of probainognathians (Fig. 5) presented here point to two major diversification events. One of them took place during the Middle Triassic and the earliest Late Triassic and included the origin of Mammaliamorpha. The second occurred mainly in the Norian and the Rhaetian and is represented by the origin of tritylodontids and closely allied forms. It is by mid-Norian times that the origin of Mammaliaformes would have occurred; however, mammaliaforms are not recognized until the latest Triassic and Jurassic. The results of our analysis imply long ghost-lineages for many taxa, including *Tessellatia bonapartei*, suggesting a still unrecorded history of Probainognathia.

Dispersal events from the Paraná Basin during the Carnian-earliest Norian were restricted to the Ischigualasto-Villa Unión Basin whereas during the late-Norian-Rhaetian dispersions are from the Paraná Basin to the Karoo Basin. This suggests that, during these two time-lapses, barriers or favorable conditions to dispersal were alternatively in place in the Ischigualasto-Villa Unión and Karoo basins preventing or encouraging dispersions from the Paraná Basin. It is also interesting to note that the three dispersion events from the Paraná Basin to the Ischigualasto-Villa Unión Basin represented by *Tessellatia bonapartei*, *Chaliminia musteloides*, and *Pseudotherium argentinus* were not followed by diversifications. These results highlight the importance of improving the knowledge on Norian probainognathians.

Our study reveals a new species of a probainognathian cynodont, adding to the poorly known cynodont diversity from the rich Norian faunal assemblage of the Los Colorados Formation. The phylogenetic evaluation of *Tessellatia bonapartei* through the most comprehensive non-mammalian cynodonts data-matrix assembled to date together with the first quantitative paleobiogeographic analysis of probainognathians highlight a previously unrecognized evolutionary and biogeographic history.

## Methods

### Micro-tomography

The small size and delicate bones make it impossible to remove the hard rock matrix without damaging the specimens and losing important information. In order to overcome this issue, PULR-V121 was analyzed through X-ray micro-tomography in YPF Tecnología S.A. (Y-TEC, Ensenada, Buenos Aires, Argentina) using the Bruker SkyScan 1173 instrument. The equipment was set up to 100 kV and 80µA. A total of 900 images of the specimen were captured through a 360° tomography (rotation step 0.4°) with an exposure time of 250 ms with two frames averaged. The experimental design resulted in a 40.01 µm pixel size. The tomographic reconstruction was then produced with the software NRecon 1.6.9.8 (https://www.microphotonics.com/micro-ct-systems/nrecon-reconstruction-software). Although obtaining acceptable results, the resolution was not ideal. Regrettably, the reconstructed images proved to be difficult to interpret, as bone and matrix were in some regions indistinguishable from each other. We acknowledged that this issue was probably technique-related due to the presence of ferruginous material in the sediment. Hence, we decided to perform a preliminary neutron-tomography at the RA-6 facility (Comisión Nacional de Energía Atómica, San Carlos de Bariloche, Argentina). Considering the promissory results obtained and the hypothesis that this specimen may represent a previously unregistered taxon of the very poorly represented mammalian ancestors in the late Late Triassic of Argentina, we performed a neutron tomography with the highest possible spatial resolution at the ANTARES instrument^56,57^ in the Forschungs-Neutronenquelle Heinz Maier-Leibnitz Zentrum (FRM II, Garching, Germany).

The specimen did not show previous radioactivity before introducing it directly to the reactor hall at ANTARES instrument. For the neutron tomography, PULR-V121 was wrapped in aluminum foil together with two additional specimens from the same stratigraphic levels (PULR-V222 and PULR-V223) to maximize the available beam-time. The package was placed in a 5 cm long slot of an aluminum cylinder. A small aluminum plate was fixed to the cylinder using aluminum tape to act as a floor. This setup feature stabilized the specimens during the tomography and allowed to place them as close as possible to the detector (Supplementary Fig. S1). At ANTARES, a collimation ratio of L/D = 500 was used. The Andor Neo sCMOS detector was equipped with a 100 mm Zeiss Milvus f2.0 lens which allowed us to obtain high resolution images, with a 19.74 µm pixel size. We performed a standard (white-beam), 360° tomography employing a Gd_2_O_2_S based neutron scintillator of 6 cm × 6 cm of 20 µm thickness. The exposure time was 17 s and each angular position (every ~ 0.192°) was acquired three times for improving statistics. The neutron tomography took circa 17 h and 42 min. In order to normalize the images obtained, 19 open beam (open shutter, no sample in the beam) and 5 dark field (closed shutter) images were taken before and after the tomographic acquisition of the fossil remains, respectively.

The images were normalized and filtered using Image-J v. 1.52p^58^ software and then reconstructed with Octopus Reconstruction 8.9.3.4 software (https://octopusimaging.eu) at the Heinz Maier-Leibnitz Zentrum facility. Posteriorly, the reconstructed images were subjected to a new filtering process with the Inverse Scale Space Filter (ISS) module implemented in KipTool^59–61^. The ISS, an edge preserving de-noising filter based on the equation formulated by Burger et al.^62^, notably increased the sharpness of the images without sacrificing morphological information. The segmentation of the specimen was performed by Dr. Gaetano who also generated the 3D surface model of PULR-V121 using Avizo 7.1 (FEI; https://www.thermofisher.com/ar/es/home/electron-microscopy/products/software-em-3d-vis/avizo-software.html). Approximately two weeks after the neutron tomography, PULR-V121 showed a very low decay ratio and was possible to remove it from the reactor hall.

### Phylogenetic analysis

We put together a data matrix combining those of Liu and Olsen^42^ and Ruta et al.^28^ considering the subsequent modifications to both of them as well as modifying or deleting some of the characters and character states. We also added new characters and included the new specimen PULR-V121 as well as other relevant taxa. As a result, a comprehensive data matrix including 73 taxa and 151 characters was produced. Previous scorings were revised, and corrections implemented (Supplementary Appendices S1-S4). The aim of our phylogenetic analysis is to test the phylogenetic relationships of the new Norian taxon, *Tessellatia bonapartei*.

A first analysis was produced after the complete data matrix. After the recognition of three wild-card taxa (i.e., *Diegocanis elegans*, *Charruodon tetracuspidatus*, and *Microconodon tenuirostris*), we pruned them from the matrix and performed a second analysis considering only the 70 remaining taxa. TNT 1.5 software^63,64^ was used for searching of most parsimonious trees. Routine used was the command xmult = level 10, that produce 14 autoconstrained replications; each replication with random sectorial searches, drifting (36 iters) and fusing (10 round), finding best score 1 time; followed by bb (bbreak) command that perform branch-swapping (tree bisection reconnection) using pre-existing trees. Characters were unordered and equally weighted. The analysis of the complete matrix resulted in 29,304 most parsimonious trees (mpt) of 786 steps; whereas the second analysis resulted in 200 mpt of 782 steps.

Most scholars agree on the putative probainognathian nature of dromatheriids, a group of small cynodonts represented by isolated teeth or fragments of mandible, mostly discovered in the Laurasia subcontinent and India^47^. However, the placement of dromatheriids has yet to be tested cladistically. Unfortunately, removal of *Microconodon tenuirostris* (one of the wildcard taxa in the complete analysis) from our second analysis, does not allow us to evaluate the relationships of this lineage.

### Paleobiogeographic analysis

A biogeographic analysis of the probainognathian clade as represented in the majority rule consensus tree of the pruned (70-taxa) phylogenetic analysis was performed in RASP 4.2 ^65^. The cladogram branch-length was temporally calibrated with RStudio^66^ using PaleoTree package^67^ with EQUAL methodology. The First Appearance Datum (FAD) and Last Appearance Datum (LAD) as well as the geographical distribution of the taxa were obtained from the relevant published sources (see^28^ and^4^ for a review). We considered nine ancestral areas according to the regions that fossils were found (Supplementary Table S1). Most areas are so distant from each other that it is not necessary to determine discontinuity between them. Additionally, almost all of them have endemic taxa. We performed a Bayesian Binary Markov Chain Monte Carlo (BBM;^68^) analysis with the calibrated cladogram, considering 100,000 cycles, 100 chains, and the maximum number of areas (nine) per node. We conducted an optimization analysis to test the possible ancestral area of relevant nodes using the Weighted Ancestral Area Analysis (WAAA;^44^). The number of weighted gain steps (GSW), weighted loss steps (LSW) and the probability index (PI = GSW/LSW) were calculated manually. The PI of each area indicates the probability of that area as a part of the ancestral area. It is important to bear in mind that the incomplete fossil record will bias any quantitative analysis. Hence, the results presented here depend on the known record of the taxa considered and changes are expected as our knowledge improves.

## Supplementary Information

Supplementary Information 1.

Supplementary Information 2.

Supplementary Information 3.

Supplementary Information 4.

Supplementary Information 5.

## Data Availability

The holotype and only known specimen of *Tessellatia bonapartei* is deposited at the Museo de Ciencias Naturales, Universidad Nacional de La Rioja under the collection number PULR-V121. The 3D model of PULR-V121 is freely accessible in MorphoMuseuM (http://morphomuseum.com^70^).

## References

[1] Rowe T (1988). Definition, diagnosis and origin of Mammalia. J. Vertebr. Paleontol..

[2] Hopson JA, Kitching JW (2001). A probainognathian cynodont from South Africa and the phylogeny of non-mammalian cynodonts. B. Mus. Comp. Zool..

[3] Abdala, F. & Gaetano, L. C. Late Triassic cynodont life, time of innovations in the mammal lineage in: *The Late Triassic World*—*Earth in a Time of Transition* (ed Tanner, L. H.) 407–445 (Springer’s Geobiology book series, 2018).

[4] Abdala F (2020). The Triassic cynodonts from Argentina: A Gondwanan perspective. J. S. Am. Earth Sci..

[5] Bonaparte JF (1971). Los tetrápodos del sector superior de la Formación Los Colorados, La Rioja Argentina. Opera Lillo..

[6] Arcucci AB, Marsicano CA, Caselli AT (2004). Tetrapod association and palaeoenviroment of the Los Colorados Formation (Argentina): A significant sample from Western Gondwana at the end of the Triassic. Geobios.

[7] Domnanovich, N. Revisión de los dicinodontes kannemeyéridos (Amniota, Therapsida) de Argentina, relaciones filogenéticas e implicancias paleobiogeográficas. *Universidad de Buenos Aires, Buenos Aires, Argentina. PhD Thesis* 387 (2010).

[8] Martínez R (2015). A new Late Triassic vertebrate assemblage from northwestern Argentina. Ameghiniana.

[9] Colombi, C. *et al*. Bonebed en las facies basales de la Formación Los Colorados (Noriano), Cuenca de Ischigualasto-Villa Unión, San Juan, Argentina. *Resúmenes Reunión de Comunicaciones de la Asociación Paleontológica Argentina, Puerto Madryn* (2018).

[10] Colombi C (2021). A high-precision U-Pb zircon age constraints the timing of the faunistic and palynofloristic events of the Carnian Ischigualasto Formation, San Juan, Argentina. J. S. Am. Earth Sci..

[11] Rougier G, de la Fuente MS, Arucci AB (1995). Late Triassic turtles from South America. Science.

[12] Sterli J, de la Fuente MS, Rougier GW (2007). Anatomy and relationships of *Palaeochersis talampayensis*, a Late Triassic turtle from Argentina. Palaeontogr. Abt. A.

[13] Bonaparte, J. F. Los tetrápodos triásicos de Argentina. *Gondwana Stratigraphy, I. U.G.S. Coloquio Mar del Plata 1967*, 307–325 (1969).

[14] Bonaparte, J. F. Annotated list of the South American Triassic tetrapods. In *Proceedings and Papers: Second Gondwana Symposium, South Africa 1970* 665–682 (1970).

[15] Martínez RN, Alcober OA, Pol D (2019). A new protosuchid crocodyliform (Pseudosuchia, Crocodylomorpha) from the Norian Los Colorados Formation, northwestern Argentina. J. Vertebr. Paleont..

[16] Leardi JM, Yañez I, Pol D (2020). South American crocodylomorphs (Archosauria; Crocodylomorpha): A review of the early fossil record in the continent and its relevance on understanding the origins of the clade. J. S. Am. Earth Sci..

[17] Bonaparte JF (1978). *Coloradia brevis* n. g. et n. sp. (Saurischia Prosauropoda), dinosaurio Plateosauridae de la Formación Los Colorados, Triásico Superior de la Rioja, Argentina. Ameghiniana.

[18] Bonaparte JF (1999). Evolución de las vértebras presacras en Sauropodomorpha. Ameghiniana.

[19] Arcucci AB, Coria RA (2003). A new Triassic carnivorous dinosaur from Argentina. Ameghiniana.

[20] Martínez RN, Alcober OA, Heredia G, Colombi CE (2004). Nuevos prosaurópodos de la Formación Los Colorados (Triásico Superior-Noriano), La Rioja, Argentina. Ameghiniana.

[21] Ezcurra MD, Apaldetti C (2011). A robust sauropodomorph specimen from the Upper Triassic of Argentina and insights on the diversity of the Los Colorados Formation. Proc. Geol. Assoc..

[22] Ezcurra MD (2017). A new early coelophysoid neotheropod from the Late Triassic of northwestern Argentina. Ameghiniana.

[23] Irmis RB (2011). Evaluating hypotheses for the early diversification of dinosaurs. Earth Environ. Sci. T. R. So. Edinburgh.

[24] El Bonaparte JF (1980). primer ictidosaurio (Reptilia, Therapsida) de América del Sur, *Chaliminia musteloides*, del Triásico Superior de La Rioja. II Congr. Argent. Paleontol. Bioestratigrafía Buenos Aires.

[25] Martinelli AG, Rougier GW (2007). On *Chaliminia musteloides* (Eucynodontia: Tritheledontidae) from the Late Triassic of Argentina, and a phylogeny of Ictidosauria. J. Vertebr. Paleontol..

[26] Gaetano LC, Abdala F, Govender R (2017). The postcranial skeleton of the Lower Jurassic *Tritylodon longaevus* from Southern Africa. Ameghiniana.

[27] Luo, Z.-X. Sister-group relationships of mammals and transformations of diagnostic mammalian characters in: *In the Shadow of the Dinosaurs* (eds Fraser, N. C., Sues, H.-D.) 98–128 (Cambridge University Press, 1994).

[28] Ruta M, Botha-Brink J, Mitchell SA, Benton MJ (2013). The radiation of cynodonts and the ground plan of mammalian morphological diversity. Proc. R. Soc. B.

[29] Martinelli AG (2017). The African cynodont *Aleodon* (Cynodontia, Probainognathia) in the Triassic of southern Brazil and its biostratigraphic significance. PLoS ONE.

[30] Gaetano LC (2019). New cynodont specimens from Los Colorados Formation. Publ. Electrón. Asoc. Paleontol. Argent..

[31] Kent DV, Santi Malnis P, Colombi CE, Alcober OA, Martínez R (2014). Age constraints on the dispersal of dinosaurs in the Late Triassic from magnetochronology of Los Colorados Formation (Argentina). PNAS.

[32] Desojo JB (2020). The Late Triassic Ischigualasto Formation at Cerro Las Lajas (La Rioja, Argentina): Fossil tetrapods, high-resolution chronostratigraphy, and faunal correlations. Sci. Rep..

[33] Schwarz D, Meyer CA, Lehmann EH, Vontobel P, Bongartz G (2005). Testing Neutron tomography in comparison with X-ray computed tomography as a technique for the investigation of the internal structure of sauropod vertebrae and ribs. Palaeontol. Electron..

[34] Laaß M, Schillinger B (2015). Reconstructing the auditory apparatus of therapsids by means of neutron tomography. Phys. Proc..

[35] Broom R (1905). On the use of the term Anomodontia. Rec. Albany Mus..

[36] Owen, R. *Palaeontology, or a Systematic Summary of Extinct Animals and their Geological Relations* 2nd edn, 1–463. (Adam and Charles Black, 1861).

[37] Kemp, T.S. *Mammal-like Reptiles and the Origin of Mammals* 1–363 (Academic Press, 1982).

[38] Hopson JA (1990). Cladistic analysis of therapsid relationships. J. Vertebr. Paleontol..

[39] Kermack KA, Mussett F, Rigney HW (1981). The skull of Morganucodon. Zool. J. Linn. Soc..

[40] Benoit J (2020). The evolution of the maxillary canal in Probainognathia (Cynodontia, Synapsida): Reassessment of the homology of the infraorbital foramen in mammalian ancestors. J. Mammal. Evol..

[41] Benoit J, Manger PR, Rubidge BS (2016). Palaeoneurological clues to the evolution of defining mammalian soft tissue traits. Sci Rep.

[42] Liu J, Olsen P (2010). The phylogenetic relationships of Eucynodontia (Amniota: Synapsida). J. Mammal Evol..

[43] Abdala F (2007). Redescription of *Platycraniellus elegans* (Therapsida, Cynodontia) from the Lower Triassic of South Africa, and the cladistic relationships of eutheriodonts. Palaeontology.

[44] Hausdorf B (1998). Weighted area analysis and a solution of the redundant distribution problem. Syst. Biol..

[45] Datta PM, Das DP (1996). Discovery of the oldest fossil mammal from India. India Min..

[46] Datta PM (2005). Earliest mammal with transversely expanded upper molar from the Late Triassic (Carnian) Tiki Formation, South Rewa Gondwana Basin, India. J. Vert. Paleontol..

[47] Bhat MS, Ray S, Datta PM (2021). New cynodonts (Therapsida, Eucynodontia) from the Late Triassic of India and their significances. J. Paleontol..

[48] Kielan-Jaworowska, Z., Cifelli, R. & Luo, Z.-X. *Mammals from the age of dinosaurs: origin, evolution and structure* 1–630 (Columbia University Press, 2004).

[49] Debuysschere M, Gheerbrant E, Allain R (2015). Earliest known European mammals: A review of the Morganucodonta from Saint-Nicolas-de-Port (Upper Triassic, France). J. Syst. Palaeontol..

[50] Luo Z-X, Martin T (2007). Analysis of molar structure and phylogeny of docodont genera. Bull. Carnegie Mus. Nat. Hist..

[51] Mancuso AC (2014). The Chañares formation: A window to a Middle Triassic tetrapod community. Lethaia.

[52] Martínez R (2011). New basal dinosaur from the dawn of the dinosaur era in Southwestern Pangea. Science.

[53] Martinelli AG, Eltink E, Da-Rosa ÁAS, Langer MC (2017). A new cynodont (Therapsida) from the *Hyperodapedon* Assemblage Zone (upper Carnian-Norian) of southern Brazil improves the Late Triassic probainognathian diversity. Pap. Palaeontol..

[54] Oliveira TV, Soares MB, Schultz CL (2010). *Trucidocynodon riograndensis* gen. nov. et sp. Nov. (Eucynodontia), a new cynodont from the Brazilian Upper Triassic (Santa Maria Formation). Zootaxa.

[55] Ruffell A, Simms MJ, Wignall PB (2016). The Carnian humid episode of the late Triassic: a review. Geol. Mag..

[56] Calzada, E., Gruenauer, F., Muehlbauer, M., Schillinger, B. & Schulz, M. New design for the ANTARES-II facility for neutron imaging at FRM II. *Nucl. Instrum. Methods Phys. Res. Sect. A. Accel. Spectrom. Detect. Assoc. Equip*. (2009).

[57] Schulz M, Schillinger B (2015). Cold neutron radiography and tomography facility. J. Large Scale Res. Facil..

[58] Rasband, W. S., ImageJ, U. S. National Institutes of Health, Bethesda, Maryland, USA. https://imagej.nih.gov/ij/ (1997–2018).

[59] Kaestner AP, Schulz M (2015). Processing neutron imaging data—quovadis?. Phys. Proc..

[60] Kaestner, A. & Carminati, C. Neutronimaging/KipTool: First official release of KipTool. 10.5281/zenodo.2578798 (2019).

[61] Carminati C, Strobl M, Kaestner A (2019). KipTool, a general purpose processing tool for neutron imaging data. SoftwareX.

[62] Burger M, Gilboa G, Osher S, Xu J (2006). Nonlinear inverse scale space methods. Commun. Math. Sci..

[63] Goloboff P, Farris JS, Nixon K (2008). TNT, a free program for phylogenetic analysis. Cladistics.

[64] Goloboff P, Catalano S (2016). TNT version 1.5, including a full implementation of phylogenetic morphometrics. Cladistics.

[65] Yu Y, Blair C, He XJ (2020). RASP 4: Ancestral state reconstruction tool for multiple genes and characters. Mol. Biol. Evol..

[66] R Core Team. R: A language and environment for statistical computing. R Foundation for Statistical Computing, Vienna, Austria. https://www.R-project.org/ (2021).

[67] Bapst DW (2012). Paleotree: an R package for paleontological and phylogenetic analyses of evolution. Methods Ecol. Evol..

[68] Ronquist F, Huelsenbeck JP (2003). MrBayes3: Bayesian phylogenetic inference undermixed models. Bioinformatics.

[69] Scotese, C. R. *Atlas of Earth History* Vol. 1, Paleogeography 52 (PALEOMAP Project, 2001).

[70] Gaetano LC (2022). 3D model related to the publication: A new cynodont from the Upper Triassic Los Colorados Formation (Argentina, South America) reveals a novel paleobiogeographic context for mammalian ancestors. MorphoMuseumM.

