# Supplementary material for: A new cynodont from the Upper Triassic Los Colorados Formation (Argentina, South America) reveals a novel paleobiogeographic context for mammalian ancestors
Source: Sci Rep. 2022 Apr 25;12:6451. doi: 10.1038/s41598-022-10486-4 (PMC9038739; doi:10.1038/s41598-022-10486-4)
Supplement: Supplementary file 1 — Supplementary Information 1. [file 41598_2022_10486_MOESM1_ESM.pdf]

SUPPLEMENTARY INFORMATION

A new cynodont from the Upper Triassic Los Colorados Formation (Argentina, South America) reveals a novel paleobiogeographic context for mammalian ancestors

L. C. GAETANO, F. ABDALA, F. D. SEOANE, A. TARTAGLIONE, M. SCHULZ, A. OTERO, J. M. LEARDI, C. APALDETTI, V. KRAPOVICKAS, and E. STEINBACH.

INDEX

1. Geological framework
2. Associated fauna
3. Methods
   3.a. Institutional abbreviations
   3.b. X-ray and neutron tomography
   3.c. Phylogenetic analysis
   3.d. Paleobiogeographic analysis
4. Results
   4.a. Description
   4.b. Comparisons
   4.c. Phylogenetic results
   4.d. Probainognathian paleobiogeography
5. Comparison between Norian faunal assemblages with probainognathian cynodonts
6. References

FIGURES AND TABLES INDEX

Figure S1. Experimental design for the CT-scan at the ANTARES instrument.
Figure S2. Biogeographic areas considered in the paleobiogeographic analysis.
Figure S3. Tomographic reconstruction of the basisphenoid of *Tessellatia*.
Figure S4. Tomographic reconstruction of the prootic of *Tessellatia*.
Figure S5. Majority rule consensus tree of the pruned (70-taxa) data matrix.
Figure S6. RASP analysis results

Table S1. Temporal and geographic distribution of probainognathian taxa.

Table S2. Main references employed for comparisons.

Table S3. Measurements of the snout of selected probainognathians.

Table S4. Measurements of the dentary of selected probainognathians.

Table S5. Estimations of ancestral areas for Probainognathia node.

Table S6. Estimations of ancestral areas for Mammaliamorpha node.

Table S7. Estimations of ancestral areas for Tritylodontidae node.

Table S8. Event matrix, event route, and probability for each node obtained by BBM analysis.

1. Geological framework

In central-western Argentina, a series of extensional depocenters record non-marine Triassic deposits (Stipanicic, 2002). One of the most relevant is the Ischigualasto-Villa Unión Basin, located in La Rioja and San Juan provinces (Fig. 1). The basin fill is 3500m thick and records fluvial, lacustrine, and eolian deposits (Stipanicic and Bonaparte, 1979). The Ischigualasto-Villa Unión Basin is divided into several stratigraphic units, the Lopingian – Lower Triassic Talampaya-Tarjados succession (fluvial-eolian) that unconformably rests on Paleozoic deposits of the Paganzo Basin and the Upper Triassic Agua de la Peña Group, unconformably overlying the Talampaya and Tarjados formations (Romer and Jensen, 1966; Martínez et al., 2011; Gulbranson et al., 2015; Kent et al., 2015; Marsicano et al., 2016). The Agua de la Peña Group is composed by, from bottom to top, the Chañares, Ischichuca, Los Rastros, Ischigualasto, and Los Colorados formations. The basal three stratigraphic units (Chañares, Ischichuca, and Los Rastros formations) were deposited in fluvial and lacustrine environments, while the upper two (Ischigualasto and Los Colorados formations) were deposited in fluvial environments (López-Gamundí et al., 1989; Milana and Alcober, 1994).

Los Colorados Formation at La Esquina locality, within the Talampaya National Park, is 700m thick and records sandstones and mudstones that represent fluvial sedimentation (Caselli et al., 2001). It comprises sandy fluvial channels of moderate to high sinuosity and over bank deposits with well-developed floodplains (Santi Malnis et al., 2020). The new specimen is preserved in a small concretion recovered from the upper third of the unit in massive- to parallel- laminated sandy mudstones interbedded with parallel- to rippled- laminated sandstones accumulated in floodplain deposits occasionally affected by crevasse splays from the fluvial channels (Gaetano et al., 2019).

The age of the Los Colorados Formation was suggested as 227–213 Ma (Norian) based on magnetostratigraphic studies in the nearby La Sal-Río Salado area (Kent at. al., 2014).

2. Associated fauna

The upper levels of the Los Colorados Formation ('La Esquina' faunal assemblage) have provided one of the oldest turtles (Rougier et al., 1995; Sterli et al., 2007); basal and derived representatives of the crocodylian lineage (Bonaparte, 1969, 1970, 1971; Martínez et al., 2019); dinosaurs (Bonaparte, 1971, 1978, 1999; Arcucci and Coria, 2003; Martínez et al., 2004; Ezcurra and Apaldetti, 2011; Ezcurra, 2017); and non-mammaliaform cynodonts (Bonaparte, 1970, 1971, 1980; Martinelli and Rougier, 2007). One of the youngest dicynodonts of Gondwana, *Jachaleria colorata*, is represented in the lower levels of the unit, belonging to a different faunal association (Domnanovich, 2010; Martínez et al., 2015; Colombi et al. 2018).

Several specimens of *Palaeochersis talampayensis*, the only record of turtles in the Formation (Rougier et al., 1995), have been found in a single outcrop. The length of the skull and the shell of the holotype was approximately 9cm and 50cm, respectively (Rougier et al., 1995; Sterli et al., 2007).

Various clades of pseudosuchians have been reported from the Los Colorados Formation. The known specimens display a wide variety of body sizes, including small taxa as well as very large ones. Most forms are large taxa (femoral length [FL] > 150mm). In particular, the largest taxa are represented by the apex predators of the association *Riojasuchus tenuiceps* (FL [PVL 3827] = 172.8mm) and *Fasolasuchus tenax* (FL = 694.5mm). The only herbivorous pseudosuchian (but see Taborda et al., 2021 for an alternative interpretation) is within these large sizes (FL = 182.2mm). The only named species of a non-crocodylform crocodylomorph, *Pseudhesperosuchus jachaleri*, represents the largest taxon among these forms (skull length [SL] = 147mm; FL = 159.8mm), although a smaller indeterminate form is known (see Leardi et al, 2020). On the other hand, the two named species of crocodyliforms are markedly smaller in size (*Hemiprotosuchus leali*, SL = 79.7mm; *Coloradisuchus abelini*, SL = 63.2mm).

The faunal assemblage from the upper levels of the Los Colorados Fm. is dominated by dinosaurs, both in abundance and taxonomic diversity (Caselli et al., 2001; Arcucci et al., 2004; Ezcurra and Apaldetti, 2011; Otero et al., 2019). The dinosaur records in this unit includes coelophysoid theropods and early branching sauropodomorphs.

Among theropod dinosaurs, two species of neotheropods have been reported from the upper levels of the Los Colorados Fm., *Zupaysaurus rougieri* (SL [PULR 076] = 500mm approximately; Arcucci and Coria, 2003; Ezcurra and Novas, 2007) and *Powellvenator podocitus* (FL [PVL 3848] = 100mm approximately; Ezcurra 2017). *Powellvenator* is a non-coelophysid Coelophysoidea, whereas *Zupaysaurus* was originally interpreted as an early tetanuran theropod, but later reinterpreted as a non-averostran neotheropod (Ezcurra and Novas 2007; Nesbitt et al 2009), and more recently as an early member of the Averostra-line of Neotheropoda (Ezcurra, 2017; Spiekman et al., 2021). *Zupaisaurus* is at the moment the largest predator dinosaur recorded from the Los Colorados Fm. Fragmentary remains of an unnamed Coelophysoidea have been recently reported from the same levels of the Los Colorados Fm. (Sotomayor et al 2019).

Sauropodomorph dinosaurs are the most abundant component among the vertebrate assemblage of the Los Colorados Formation. Furthermore, the upper levels of this formation bear the highest sauropodomorph diversity among Late Triassic South American dinosaur-bearing assemblages (Martínez et al., 2015; McPhee et al., 2017; Apaldetti et al., 2018, 2021). Recently, sauropodomorph remains have also been recognized in the “dicynodont bone-bed” discovered in the most-basal deposits of Los Colorados Fm. (Colombi et al 2018; CA pers. obs.). So far, three species of disparate size have been described from the upper portion of the unit: the medium-sized massospondylid *Coloradisaurus brevis* (Bonaparte, 1978; Apaldetti et al., 2013), the large-sized *Riojasaurus incertus* (Bonaparte, 1971; Bonaparte and Pumares, 1995), and the giant *Lessemsaurus sauropoides* (Bonaparte, 1999; Pol and Powell, 2007; Apaldetti et al., 2018). Estimations of body mass were performed through the free software RStudio version 1.3.1093 (RStudio Team, 2020) using the quadratic equation provided by Campione (2017), which utilizes the minimum shaft circumference of the femur for bipedal taxa. The results suggest that *Coloradisaurus brevis* had of body mass of about 380kg (FL [PVL 5904] = 500mm), *Riojasaurus incertus* weighted 2200kg (FL [PVL 3808] = 600mm), and *Lessemsaurus sauropoides* reached 8500kg (FL [PVL 4822/65] = 820mm).

Coloradisaurus and *Riojasaurus* have traditional been considered early-diverging Sauropodomorpha, although some authors have recently depicted to *Riojasaurus* more closely related to Sauropodiformes than to earliest forms (McPhee et al., 2018, 2019), whereas the robust *Lessemsaurus* is related to the origin of sauropod dinosaurs and to the early evolution of gigantism among Sauropodomorpha (Apaldetti et al., 2018; McPhee et

al., 2018). An additional, indeterminate, sauropodomorph (PULR 136) has been reported from the Los Colorados Formation, consisting of a robust tibia (length = 400 mm) and caudal remains (Ezcurra and Apaldetti, 2011). Moreover, an undescribed almost complete skeleton has also been reported (PVSJ 2003#06; Martínez et al., 2004).

Non-mammaliaform cynodonts are also present in this unit, although scarcely represented (Bonaparte, 1970, 1980; Martinelli and Rougier, 2007). Only two partial skulls of *Chaliminia musteloides* (Bonaparte, 1980; Martinelli and Rougier, 2007) and a few fragmentary postcranial elements of an unnamed taxon (Bonaparte, 1970, 1971; Gaetano et al., 2017) have been reported. Cynodonts represent the smallest components of the faunal association with an estimated skull length of *Chaliminia* not surpassing 30mm.

3. Methods

3.a. Institutional abbreviations

BP, Evolutionary Studies Institute (formerly Bernard Price Institute for Palaeontological Research), University of the Witwatersrand, Johannesburg, South Africa; **CAPA/UFSM**, Centro de Apoio à Pesquisa Paleontológica, Universidade Federal de Santa Maria, São João do Polêsine, Brazil; **MCN-PV**, Museu de Ciências Naturais, Fundação Zoobotânica do Rio Grande do Sul (FZBRS), Rio Grande do Sul, Brazil; **MVP**: Museu Vicente Pallotti, Santa Maria, Rio Grande do Sul, Brazil; **NMQR**, National Museum, Bloemfontein, South Africa; **PULR**, Paleontología, Universidad Nacional de La Rioja, Argentina; **PVL**, Colección Paleontología de Vertebrados, Instituto Miguel Lillo, Universidad Nacional de Tucumán, Tucumán, Argentina; **PVSJ**, Paleontología de vertebrados, Instituto y Museo de Ciencias Naturales, Universidad Nacional de San Juan, San Juan, Argentina; **SAM-PK**, Iziko South African Museum, Cape Town, South Africa; **UFRGS-PV-T**, Laboratório de Paleontologia de Vertebrados, Universidade Federal do Rio Grande do Sul, Triassic Collection, Rio Grande do Sul, Brazil.

3.b. X-ray and neutron tomography

The small size and delicate bones make it impossible to remove the hard rock matrix without damaging the specimens and losing important information. In order to overcome this issue, PULR-V121 was analyzed through X-ray micro-tomography in YTEC (Ensenada, Buenos Aires, Argentina) using the Bruker SkyScan 1173 instrument. The equipment was set up to 100kV and 80µA. A total of 900 images of the

specimen were captured through a 360° tomography (rotation step 0.4°) with an exposure time of 250ms with two frames averaged. The experimental design resulted in a 40.01µm pixel size. The tomographic reconstruction was then produced with the software NRecon v. 1.6.9.8. Although obtaining acceptable results, the resolution was not ideal. Regrettably, the reconstructed images proved to be difficult to interpret, as bone and matrix were in some regions indistinguishable from each other. We acknowledged that this issue was probably technique-related due to the presence of ferruginous material in the sediment. Although only scarcely used to analyze fossil specimens, it has become clear that neutron µCT complements very well-with X-ray µCT as well as allows to circumvent some specific problematics that might arise from the latter methodology depending on the characteristics of the fossil sample studied (Schwarz et al. 2005; Laaβ and Schillinger, 2015). Hence, we decided to perform a preliminary neutron-tomography at the RA-6 facility (Comisión Nacional de Energía Atómica, Bariloche, Argentina). Considering the promising results obtained and the hypothesis that this specimen may represent a previously unidentified taxon of the very poorly represented mammalian ancestors in the Late Triassic of Argentina, we performed a neutron tomography with the highest possible spatial resolution at the ANTARES instrument (Calzada et al., 2009; Schulz and Schillinger, 2015) in the Forschungs-Neutronenquelle Heinz Maier-Leibnitz Zentrum (FRM II, Garching, Germany).

The specimen did not show previous radioactivity before introducing it directly to the reactor hall at ANTARES instrument. For the neutron tomography, PULR-V121 was wrapped in aluminum foil together with two additional specimens from the same stratigraphic levels (PULR-V222 and PULR-V223) to reduce the required beam-time. The package was placed in a 5cm long slot of an aluminum cylinder. A small aluminum plate was fixed to the cylinder using aluminum tape to act as a floor. This setup feature stabilized the specimens during the tomography and allowed to place them as close as possible to the detector (Fig. S1). At ANTARES, a collimation ratio of L/D=500 was used. The Andor Neo sCMOS detector was equipped with a 100mm Zeiss Milvus f2.0 lens which allowed us to obtain high resolution images, with a 19.74µm pixel size. We performed a standard (white-beam), 360° tomography employing a Gd_2O_2S based neutron scintillator of 6 cm x 6 cm of 20µm thickness. The exposure time was 17s and each angular position (every ~0.192°) was acquired three times for improving statistics. The neutron tomography took circa 17 hours and 42 minutes. In order to normalize the

images obtained, 19 open beam (open shutter, no sample in the beam) and 5 dark field (closed shutter) images were taken before and after the tomographic acquisition of the fossil remains, respectively.

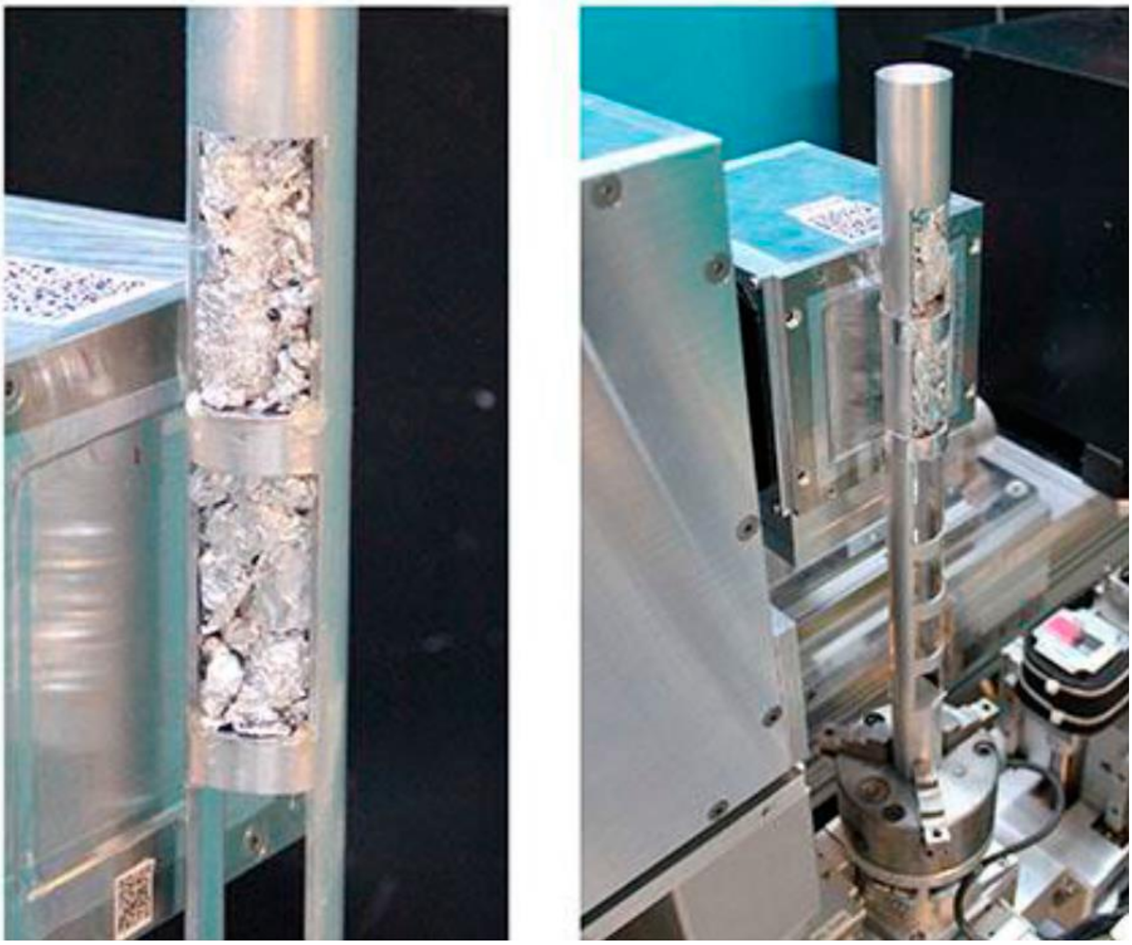

Figure S1. Experimental design for the CT-scan at the ANTARES instrument showing the specimens wrapped in aluminum foil and placed in an aluminum cylinder as close as possible to the detector.

The images were normalized and filtered using Image-J v. 1.52p (Rasband, 1997-2018) software and then reconstructed with Octopus Reconstruction v. 8.9.3.4 software at the Heinz Maier-Leibnitz Zentrum facility. Afterwards, the reconstructed images were subjected to a new filtering process with the Inverse Scale Space Filter (ISS) module implemented in KipTool (Kaestner and Schulz, 2015; Kaestner and Carminati, 2019, Carminati et al., 2019). The ISS, an edge preserving de-noising filter based on the equation formulated by Burger et al. (2006), notably increased the sharpness of the images without sacrificing morphological information. The segmentation of the specimen was

performed by Dr. Gaetano and the resultant 3D model of PULR-V121 is freely accessible in MorphoMuseuM (http://morphomuseum.com; Gaetano et al., 2022). Approximately two weeks after the tomography, the induced radioactivity of PULR-V121 had sufficiently decayed to remove it from the reactor hall.

3.c. Phylogenetic analysis

In 2010, Liu and Olsen presented a very complete phylogenetic analysis of non-mammalian cynodonts based on previously published information on cynodont relationships including Hopson and Barghusen (1986), Rowe (1988), Wible (1991), Crompton and Luo (1993), Luo and Crompton (1994), Luo (1994), Martínez et al. (1996), Luo et al. (2002), Hopson and Kitching (2001), Abdala and Ribeiro (2003), Bonaparte et al. (2003, 2005), Martinelli et al. (2005), Abdala (2007), and Martinelli and Rougier (2007). With 31 taxa and 145 cranial, dental, and postcranial characters, Liu and Olsen's (2010) was the most taxonomically and anatomically comprehensive cynodont data matrix ever presented. Later, this dataset was modified and updated by several subsequent authors, among them Martínez et al. (2013), Soares et al. (2014), Gaetano and Abdala (2015), Martinelli et al. (2016 and 2017b), and Wallace et al. (2019).

Concomitantly, Ruta et al. (2013) compiled a data matrix of 54 cynodont taxa and 150 characters, including cranial, dental, and postcranial traits. This dataset was mainly based on the previous work of Hopson and Kitching (2001) and Abdala (2007), but several other sources were also considered (Bonaparte et al., 2001; Martinelli et al., 2005; Abdala et al., 2006; Oliveira, 2006; Sidor and Hancox, 2006; Martinelli and Rougier, 2007; Kammerer et al., 2008; Reichel et al., 2009; Gao et al., 2010; Liu and Olsen, 2010; Oliveira et al., 2010; Kammerer et al., 2012). Later, Martinelli et al. (2017a) introduced modifications to the scoring of certain taxa (*Chiniquodon*, *Aleodon brachyrhamphus*, *Probainognathus*).

In the present study, we put together a data matrix combining those of Liu and Olsen (2010) and Ruta et al. (2013) considering the subsequent modifications to both of them as well as modifying or deleting some of the characters and character states (see Supplementary Appendices S1-2). We also added new characters. In addition, we included the new specimen PULR-V121 as well as other relevant taxa (see Supplementary Appendices S1-S3). As a result, a comprehensive data matrix including 73 taxa and 151 characters was produced. Previous scorings were revised, and corrections implemented (see Supplementary Appendices S1-S2, S4).

A first analysis was produced after the complete data matrix and a second analysis was performed on a matrix having 70 taxa (three wildcard taxa were eliminated of the matrix: *Diegocanis*, *Charruodon*, and *Microconodon*). TNT 1.5 software program (Goloboff et al. 2008; Goloboff and Catalano, 2016) was used for searching of most parsimonious trees. The routine used was the command xmult=level 10, that produce 14 autoconstrained replications; each replication with random sectorial searches, drifting (36 iters) and fusing (10 round), finding best score 1 time; followed by bb (bbreak) command that perform branch-swapping (tree bisection reconnection) using pre-existing trees. Characters were unordered and equally weighted.

3.d. Paleobiogeographic analysis

A paleobiogeographic analysis in RASP 4.2 (Yu et al., 2020) requires two set of data: a phylogeny and a geographic provenance data matrix of the taxa studied. The phylogeny employed is the majority rule consensus of the pruned data matrix (70-taxa) presented in this study, focusing only on the clade Probainognathia (32-taxa; Fig. S5). Taxa included in this analysis are detailed in Table S1.

The cladogram branch-length was temporally calibrated with RStudio (R Core Team, 2021) using PaleoTree package (Bapst, 2012) with EQUAL methodology. The First Appearance Datum (FAD) and Last Appearance Datum (LAD) as well as the geographical distribution of the taxa were obtained from: Abdala et al. (2020) for *Alemoatherium*, *Aleodon brachyramphus*, *Aleodon cromptoni*, *Botucaraitherium*, *Brasilodon*, *Candelariodon*, *Chaliminia*, *Chiniquodon*, *Irajatherium*, *Lumkuia*, *Protheriodon*, *Prozostrodon*, *Pseudotherium*, *Riograndia*, *Santacruzgnathus*, and *Therioherpeton*; and Ruta et al. (2013) for *Adelobasileus*, *Bienotherium*, *Diarthrognathus*, *Elliotherium*, *Kayentatherium*, *Morganucodon*, *Oligokyphus*, *Pachygenelus*, *Sinoconodon*, *Tritheledon*, and *Tritylodon*. We considered nine ancestral areas according to the regions where fossils were found (Fig. S2; Table S1). Areas are so distant from each other that it is not necessary to determine discontinuity between them. Additionally, except from the European basins and the Otiwarongo Basin (Namibia), all of them have endemic taxa. We performed a Bayesian Binary Markov Chain Monte Carlo (BBM; Ronquist and Huelsenbeck, 2003) analysis with the calibrated cladogram, considering 100,000 cycles, 100 chains, and the maximum number of areas (nine) per node.

Due to the polytomies present in the phylogeny, we were unable to perform other analyses, such as an S-DIVA or DEC, in order to compare the results obtained with the Bayesian analysis. On the other hand, we conducted an optimization analysis to test the possible ancestral areas of relevant nodes, as Probainognathia (node 55), Mammaliamorpha (node 49), and Tritylodontidae (node 42), using the Weighted Ancestral Area Analysis (WAAA; Hausdorf, 1998). The number of weighted gain steps (GSW), weighted loss steps (LSW), and the probability index (PI = GSW/LSW) were calculated manually. The PI of each area indicates the probability of that area as a part of the ancestral area (see Tables S5–7 in section 4.d). The considered areas in the study are explained below (Fig. S2; Table S1):

Area A: Ischigualasto-Villa Unión Basin. This area corresponds to the fossil localities from La Rioja (Los Colorados Formation) and San Juan (Ischigualasto Formation) provinces (north-west Argentina). According to the species found and absolute dating studies (see Abdala et al., 2020), this area represents deposits of the Carnian and Norian. The three genera found in this area are endemic.

Area B: Paraná Basin. This area corresponds to the deposits from southeastern Brazil (Santa Maria and Caturrita formations), encompassing Ladinian, Carnian, and Norian ages. Twelve genera have been found in this area and ten are endemic.

Area C: Lufeng Basin. This area corresponds to the Lufeng Formation (southwest China), where four genera were found and two of them are endemic. Despite sharing two genera with European and North American basins, the distance between these regions allows considering this area as a separated one. The ages of the deposits from the Lufeng Basin are Rhaetian–Bathonian.

Area D: Karoo Basin. This area includes the six genera found in South Africa, coming from Burgersdorp and Elliot formations, with ages spanning Anisian through Hettangian–Pliensbachian. Five of the six genera found are endemic and the only genus shared is with North America, justifying its consideration as a separate area.

Area E: European basins. Two genera were found in different European outcrops including several quarries in France (Syren quarry) and UK (Duchy, Ewenny, Pontalun, Windsor Hill, and Pant Fissure Systems 2, 4, and 5 quarries at St. Bride's Island) as well as in the Forest Marble Formation (Great Oolite Group, UK), Klettgau Formation (Switzerland), and Exter Formation (Keuper Group, Germany). Due to the proximity of these regions, we considered them as part of a single area. The two genera are shared with the Lufeng Basin and the North American basins, but because of the distance between

these three areas we considered them as separated. The ages of the European deposits are Rhaetian–Bathonian.

Area F: North American basins. This area is the amalgamation of three depocenters represented by the Tecovas Formation (Palo Duro Basin), the Kayenta Formation (Colorado Plateau), and the McCoy Brook Formation (Nova Scotia). The first two are very close to each other justifying their joint consideration. Despite the outcrops of the McCoy Brook Formation are relatively distant from those of the USA, they are treated as part of the same area because considering the Canadian area as a separate one did not modify the result of the analysis due to the nested position of the taxa (see below). Five genera were found in this area and two of them are endemic. The ages of the North American bearing deposits are Carnian-Norian and Rhaetian–Bathonian.

Area G: Ruhuhu Basin. This area represents Manda beds deposits of Tanzania and was considered a separate area due to the presence of the endemic taxon *Aleodon brachyramphus*. The age of the deposits is Anisian.

Area H: Otiwarongo Basin. The two genera found in Namibia, coming from Omingonde Formation, were included in this area. Although both genera are shared with other areas (Ischigualasto-Villa Unión, Paraná, and Morondava basins), the species *Chiniquodon omaruruensis* is endemic of this area, and that allowed us to define this area as an independent one. The Namibian deposit is assigned a Ladinian–Carnian age.

Area I: Morondava Basin. This area is represented in Madagascar. In this area was found one genus shared with others three areas (Ischigualasto-Villa Unión, Paraná, and Otiwarongo basins), but in the Morondava Basin only lived one species, *Chiniquodon kalanoro*, which is endemic. Due to the presence of an endemic taxon and the distance between the Morondava Basin and other areas, we consider this region as a separate one. The known specimen comes from of Isalo II beds and the age of the deposit is Carnian.

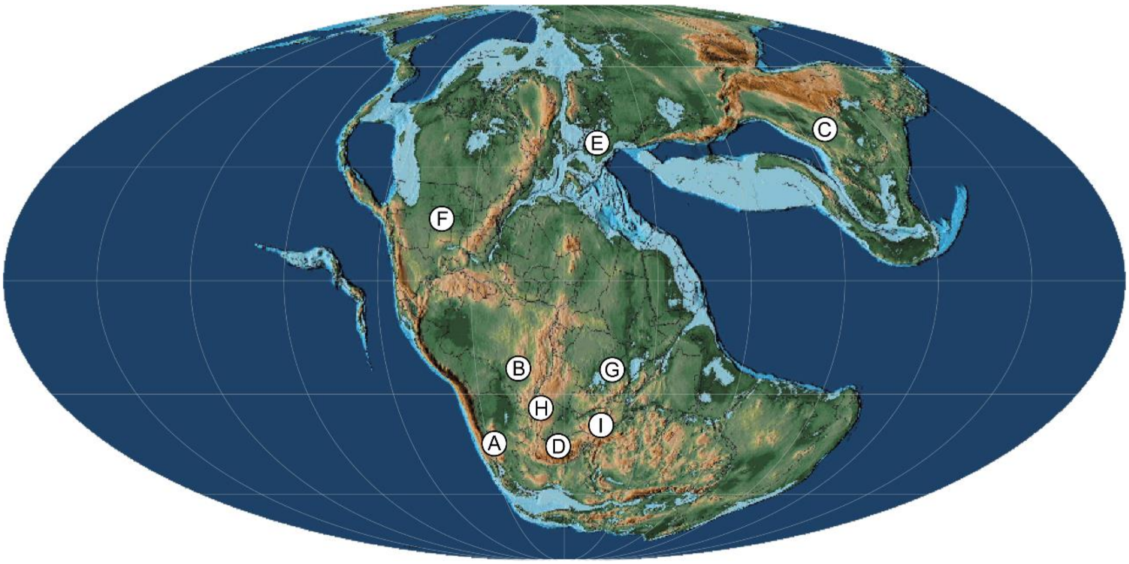

Figure S2. Paleogeographic reconstruction of the Norian time-slice (210 Ma) showing the nine biogeographic areas considered in the paleobiogeographic analysis. Abbreviations: A, Ischigualasto-Villa Unión Basin; B, Paraná Basin; C, Lufeng Basin; D, Karoo Basin; E, European basins; F, North American basins; G, Ruhuhu Basin; H, Otiwarongo Basin; I, Morondava Basin. Reconstruction taken from Scotese (2001).

TABLE S1. Temporal and geographic distribution of probainognathian taxa. *Indicates monospecific genus.

**Taxon**	**Area**	**Age**	**Country**
*Adelobasileus**	F	Carnian-Norian	USA
*Alemoatherium**	B	Carnian	Brazil
Aleodon brachyramphus	G	Ladinian-Carnian	Tanzania
Aleodon cromptoni	BH	Ladinian-Carnian	Brazil, Namibia
Bienotherium	C	Hettangian-Pliensbachian	China
*Botucaraitherium**	B	Norian	Brazil
*Brasilodon**	B	Norian	Brazil
*Candelariodon**	B	Ladinian	Brazil
*Chaliminia**	A	Norian	Argentina
Chiniquodon	ABHI	Ladinian-Carnian	Argentina, Brazil, Namibia, Madagascar
*Diarthrognathus**	D	Pliensbachian	South Africa
*Elliotherium**	D	Hettangian	South Africa

*Irajatherium**	B	Norian	Brazil
*Kayentatherium**	F	Sinemurian-Pliensbachian	USA
*Lumkuia**	D	Anisian	South Africa
Morganucodon	CEF	Rhaetian-Bathonian	China, Europe, USA
Oligokyphus	CEF	Hettangian–Pliensbachian	China, Europe, USA
*Pachygenelus**	DF	Hettangian–Pliensbachian	South Africa, Canada
*Protheriodon**	B	Ladinian	Brazil
*Prozostrodon**	B	Carnian	Brazil
*Pseudotherium**	A	Carnian	Argentina
*Riograndia**	B	Norian	Brazil
*Santacruzgnathus**	B	Carnian	Brazil
*Sinoconodon**	C	Sinemurian	China
*Therioherpeton**	B	Carnian	Brazil
*Tessellatia**	A	Norian	Argentina
*Tritheledon**	D	Sinemurian	South Africa
*Tritylodon**	D	Hettangian-Sinemurian	South Africa

4. Results.

4.a. Description

We present herein the complete description of the specimen.

Skull. The skull is only partially preserved, represented mostly by the antorbital region. The elements recovered are fragmentary, including portions of the right premaxilla, maxillae, right nasal, frontals, lacrimals, palatines, and pterygoids. Two bones too incomplete to confidently determine might represent parts of the basisphenoid and prootic.

The skull is relatively high and inflated immediately anterior to the orbits, at the level of the lacrimals. Anteriorly, the long snout narrows strongly and is interpreted to have been comparatively low. The narrowest region of the snout is represented by a constriction just posterior to the canines; anteriorly, it slightly widens into a slightly expanded rostrum in dorsal view.

The right premaxilla is represented by a rectangular strip of the facial region and a small portion of the palatal process. The contact with the maxilla is approximately vertical and anteriorly concave. It is placed anterior to the most posterior and single upper incisor preserved, which is in turn apparently placed in the maxilla.

The right maxilla is better preserved than the left one. The alveolar margin is partially preserved in both maxillae showing a sigmoidal outline in lateral view. Anteriorly, the alveolar border is concave, reaching its dorsal-most point at the level of the canine, whereas it is straight to slightly convex in the posterior half of the maxilla. The facial process of the maxilla extends over the lateral surface of the snout anterior to the lacrimal. Posterior to the level of the PC7, the maxilla becomes relatively low, only slightly higher than the roots of the postcanines. Posteriorly, the maxilla tappers into an incompletely preserved zygomatic process, which was posterior to the tooth row. The palatal processes of both maxillae are almost complete, except for their anterior region. A paracanine fossa is absent, which is concordant with the lack of large lower canines. The contact with the palatines is U-shaped and occurs a bit anterior to the level of the PC7.

Together with the maxillae, the palatal processes of the palatines form a ventrally bowed secondary osseous palate that extends to the level of the PC11. Posteriorly, the palatines almost reach the level of the tips of the upper postcanines. The secondary osseous palate is posteroventrally oriented, forming a ~19° angle to the horizontal. Deep, narrow grooves are present medial to the upper tooth rows, posterior to the maxillae-palatines suture. The dorsal plate of the palatine extends posteriorly as a high crest that ends in a ventromedially–pointing triangular projection ('choanal crest' of Barghusen, 1986) at the level of the last two upper postcanines. The orbital process of the palatine is anteroporteriorly wide and dorsoventrally long. It ascends obliquely, forming an angle of ~115° to the palatal process, and contacts the lacrimal anteroventrally. The orbital process of the palatine is thus excluded from the orbital wall and a contact with the orbital process of the frontal is precluded by the lacrimal.

Part of the anterior (palatal) process of the right pterygoid is preserved in contact with the palatine. A groove is defined in the lateral margin of the primary palate, immediately medial to the posterior crest of the palatine. The contribution of the pterygoid to this crest is difficult to ascertain, specially posteriorly where the pterygoid is expected to form the choanal crest. Posteromedially, a plate-like structure formed by the two pterygoids and, probably, also by the rostrum of the parasphenoid constitutes the base of the skull posterior to the unpreserved vomers. As preserved, this portion of the roof of the

primary palate is well posterior to the end of the osseous secondary palate. Only the anteriormost portion of the median ridge is preserved. Anteriorly, this ridge bifurcates into two diverging crests. Laterally, conspicuous lateral ridges are present. Together, these structures define the medial trough of the pterygoid. Dorsally, there is a well-developed median crest that also bifurcates anteriorly.

Two fragmentary bones, moved from their original position, are preserved posterior to the pterygoid. Although a confident determination is impossible to make, the visible morphological traits and the complexity of these elements suggest that they might in fact represent part of the basisphenoid and prootic bones. Despite their incompleteness, and that the segmentation process might have resulted in some artifacts (e.g., false or occluded foramina), we attempt a description of these elements.

The anterior portion of the basisphenoid is preserved anterolaterally displaced from its original position (Fig. S3). The cultriform process is missing. Ventrally, it is concave and a median keel is absent. The right basipterygoid process for contact with the pterygoid is present and projected ventrolaterally and slightly posteriorly. The tuberculum sellae is preserved as well as the anterior portion of the sella turcica. A foramen is tentatively recognized on the left side, piercing the tuberculum sellae. This foramen could represent the internal carotid foramen. A second, larger foramen goes through the base of the basipterygoid process.

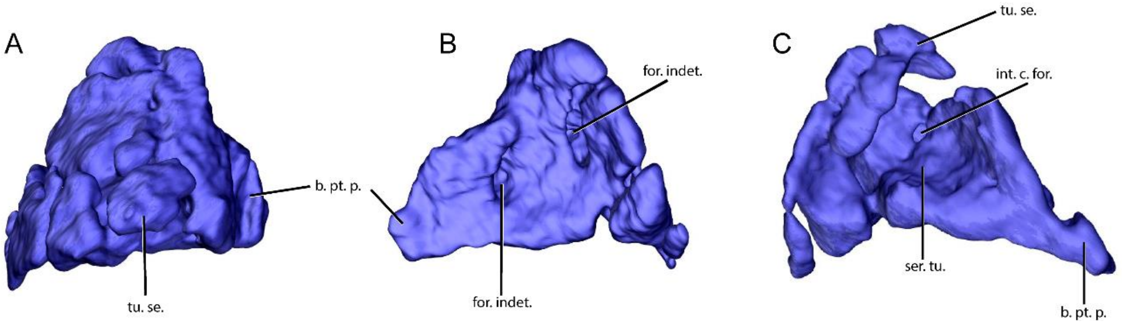

Figure S3. Tomographic reconstruction of the basisphenoid in (a) dorsal, (b) ventral, and (c) posterior views. Abbreviations: *b.pt.p.* basipterygoid process, *for. indet.* indeterminate foramen, *int.c.for.* foramen for the internal carotid, *se.tu.* sella turcica, *tu.se.* tuberculum sellae.

The prootic is represented by part of the promontorium and the lateral lamina (Fig. S4). We acknowledge the paper by Kermack et al. (1981) to be the best illustrated publication regarding the petrosal anatomy of an early mammaliaform (i.e.,

Morganucodon), better than any other concerning non-mammaliaform cynodonts. Hence, despite not being the natural orientation of the prootic and the relatively derived condition of *Morganucodon*, we follow Kermack et al. (1981) in the orientation of this element and rely on their paper for the identification of the recognizable structures.

The promontorium is conical. The medial edge of the promontorium contacted the basisphenoid and basioccipital bones (b. sph. f. and b. occ. f.). Part of the recess for the semilunar ganglion (r. s. l. ga.) is interpreted to be present lateral and dorsal to the promontorium. Only what is interpreted to be the base of the jugular notch (ju. n.) is preserved and would represent the prootic contribution to the jugular foramen.

A very small region corresponding to the base of the anterior lamina (ant. lam. pet.) is present laterally as a continuation of the lateral flange of the prootic. A relatively large, unidentified foramen is observed laterally piercing the base of the anterior lamina of the petrosal. However, we were not able to segment the opening of this foramen on the other side of the anterior lamina, which cast doubts on our interpretation.

Cranially (see dorsal and medial views), there is a groove that leads to a large opening. It is clear that this opening corresponds to two foramina and that the bony wall between them has been lost. These foramina are interpreted as the foramen for superior vestibular nerve VIII (for. sup. vest.) and foramen for cranial nerve VIII (for. VIII; cochlear, inferior, and posterior vestibular nerves). Posterior to these foramina there are two openings that are best interpreted as artifacts. Laterally to the VIII foramina and medially to the recess for the semilunar ganglion, there are two additional foramina. One of them is interpreted as for the cranial opening of the aqueductus fallopii (cr. op. aq. fal.; cranial nerve VII) whereas the other one remains indeterminate as there is no foramen in this position in *Morganucodon*. Posteriorly, medial to the inflated vestibular area, there is a small aquaeductus vestibuli foramen (aq. vest.) for the ductus endolymphaticus. A deep sulcus leads to this foramen The relative position of the aquaeductus vestibuli foramen is concordant to where the crus commune is expected. Anteromedially to the aquaeductus vestibuli foramen, a larger undetermined foramen is present. This foramen pierces the prootic posteromedially and is also very close to the vestibular area; although it is more anteriorly positioned than expected, it could be alternatively interpreted as the aquaeductus vestibuli foramen instead of the previously described smaller foramen.

In anterior view, two foramina are present ventral to the recess for the semilunar ganglion. The larger more medial of them is foramen C. Ventrolaterally to it, a smaller foramen is interpreted to be foramen D. A large foramen is present near the base of the

anterior lamina laterally; the exit of this foramen could not be recognized, and a certain identification of this foramen is not possible.

On the medial half of the ventral face of the promontorium there are four well defined foramina that are not present in *Morganuconodon*. These foramina are unexpected and could be artifacts of the segmentation process.

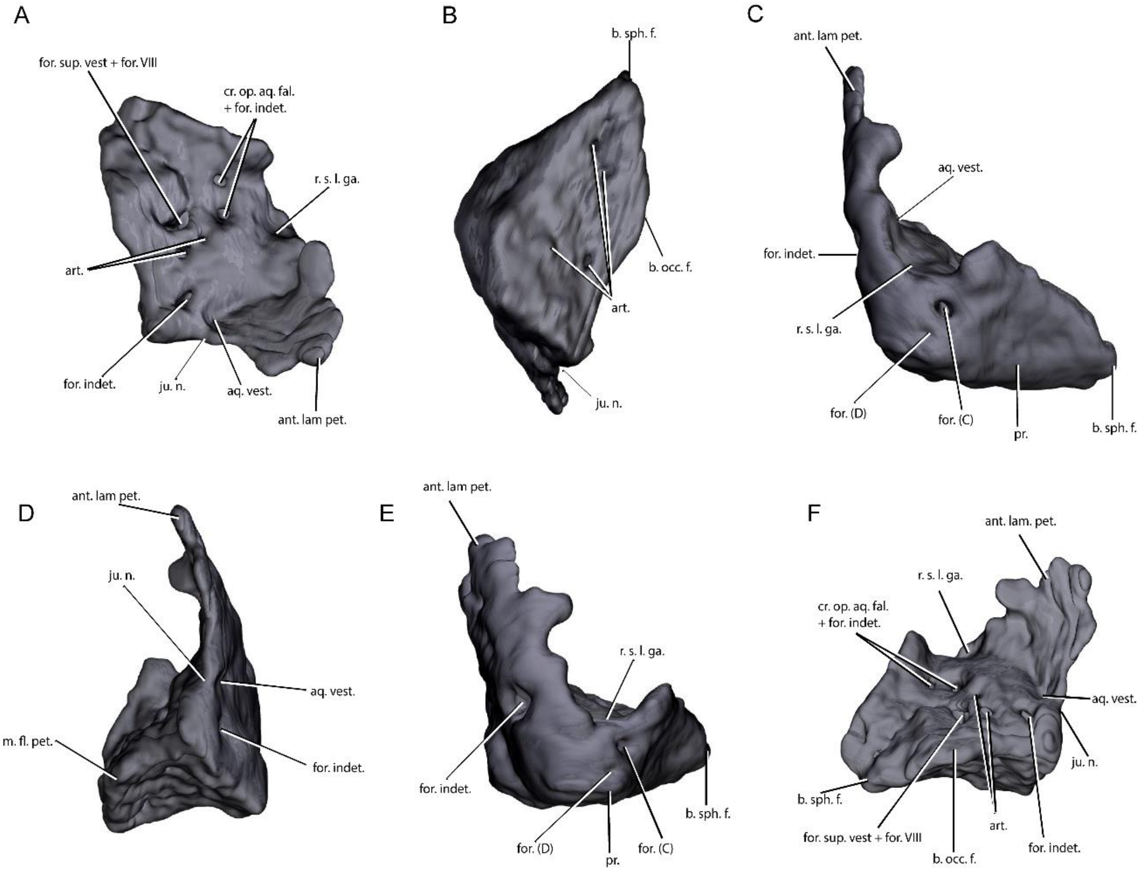

Figure S4. Tomographic reconstruction of the prootic in (a) dorsal, (b) ventral, (c) anterior, (d) posterior, (e) lateral, (f) medial views. Abbreviations: *ant. lam. pet.* anterior lamina of prootic, *aq. vest.* aquaeductus vestibuli foramen, *art.* artifact, *b. occ. f.* basioccipital facet, *b. sph. f.* basisphenoid facet, *cr. op. aq. fal.* cranial opening of the aqueductus fallopii, *for. (C)* foramen C, *for. (D)* foramen D, *for. indet.* indeterminate foramen, *for. sup. vest.* foramen for superior vestibular nerve VIII, *for. VIII* foramen for cranial nerve VIII, *ju. n.* jugular notch, *m. fl. pet.* medial flange, *pr.* promontorium, *r. s. l. ga.* recess for the semilunar ganglion.

The maxillary canal in *Tessellatia* is completely ossified and separated from the maxillary sinus. A short canal, which can be traced back in the CT-slices to the maxillary canal, leads to a small foramen at the level of the PC13 on the maxilla. These structures are best interpreted as the infraorbital canal and foramen (infraorbital foramen 2 in

Kermack et al., 1981, see Benoit et al., 2020). Alternatively, they might represent the zygomaticofacial canal and foramen (infraorbital foramen 3 in Kermack et al., 1981, see Benoit et al., 2019). However, the zygomaticofacial foramen is located at the anteroventral border of the lacrimal (Hahn, 1985), not on the maxilla, usually at the suture between the jugal and lacrimal bones (see Benoit et al., 2020). Another possibility is that they might be homologous with the middle alveolar canal and foramen. However, this foramen is placed ventral to the base of the zygomatic arch in derived probainognathians such as *Pachygenelus*, *Tritylodon*, and *Brasilodon* (see Benoit et al., 2020).

Only the anterior portion of the poorly preserved right nasal is present. It is disconnected from surrounding bones. The lacrimal is a large bone that constitutes ~40% of the length of the antorbital region. It contacts the maxilla ventrally and excludes it from the margin of the orbit anteroventrally. The lacrimal forms the anterior margin of the orbit which is placed at the level of the penultimate postcanine (PC13). Additionally, it comprises almost the entire anterior surface of the orbital cavity, except for the dorsomedial corner, which is constituted by the frontal.

The suture between right and left frontals is only subtly hinted at in the CT data. As preserved, they are somewhat distorted and displaced from their original position. The frontal is a triangular-shaped bone. It is widest anteriorly and contacts the nasals in a W-shaped suture. It narrows posteriorly into a pointy tip. The prefrontal and postorbital are interpreted to be absent. There is no hint of presence of a postorbital process of the frontal.

Lower jaw. The right and left dentaries were recovered. The right dentary is better preserved than the left one; the symphyseal region as well as the posterior portion are lacking in both of them. The dentaries are only slightly divergent from each other, giving the lower jaws a low-angled V-shaped aspect in ventral view. The dentary is relatively robust, high, and lateromedially wide. It is lateromedially wide in the anterior portion and thinner posterior to the tooth row, with a constriction separating these two regions in dorsal view. There is a broad groove between the lateral wall of the dentary and the postcanine alveoli. This groove starts at the posterior half of pc2 and ends lateral to the penultimate lower postcanine (pc6). The wall of the dentary medial to the tooth row is damaged in both elements.

The anterior region of the dentary is upwardly bent, making the dorsal margin of the horizontal ramus of the dentary concave in lateral view. Posterior to the tooth row, the coronoid process remains low, sloping dorsally with a very low angle. The anterior region

of the coronoid process is inflated lateromedially; posteriorly the coronoid process becomes thinner. The oval masseteric fossa is restricted to the region of the dentary posterior to the teeth and ventral to the anterior portion of the coronoid process. The ventral margin of the dentary is sigmoidal in lateral view. Posteriorly, the dentary flares ventrally into a semicircular lamina, with its most ventral portion representing the angular process.

Medially, the pterygoid fossa is present posteriorly to the coronoid bone. The coronoid boss is only slightly developed. The right coronoid is preserved in place, posterior to the last lower postcanine alveolus and ventral to the anterior coronoid ridge. It is a relatively large element, occupying almost the entire height of the dentary. The coronoid is incompletely preserved but it can be ascertained that it had an oval outline with its long axis obliquely oriented, forming an angle of approximately 50º with the horizontal. The coronoid is lateromedially flat in general terms. It shows a thicker anterior margin and becomes thinner posteriorly. A shallow sulcus, interpreted as the Meckelian groove, is present medially close to the ventral margin of the dentary at the level of pc4-pc6, disappearing anteriorly.

Upper teeth. The upper dentition includes at least one incisor, one canine, and a minimum of eight postcanines (1+?I/1C/14PC). Only a fragment of an upper incisor is present. It is followed by a pre-canine diastema and thus interpreted as the most posterior incisor. As judged from the basal part of the crown, the preserved incisor was smaller than any of the preserved upper postcanines. It is compressed labiolingually and probably was not recurved, as both the mesial and distal margins are straight and parallel to each other. The incisor is not procumbent but somewhat distally oriented. A subtle central concavity is present along the lingual and labial surfaces of the crown, resulting in an 8-shaped cross-section.

The upper canine is only represented by part of the lingual external surface. It was notably large and only slightly curved distally. The basal portion of the canine crown is approximately twice the anteroposterior length of those of the postcanines and about four times larger than the incisor.

The last eight posterior upper postcanines (PC7-14) are present on the right side of the skull whereas only three posterior postcanines (PC9-11) are preserved on the left maxilla. The upper postcanine tooth row is laterally convex in ventral view. All the upper postcanines are erupted but the right PC8, which is represented by a fragment of the crown

inside the crypt. The crowns of the right and left PC10 and the right PC12 are at the same level but in a lower position regarding the adjacent teeth suggesting that they are still in the process of erupting and that they belong to the same teeth generation. Hence, a replacing pattern of alternate teeth, similar to what is observed in the lower postcanines, likely occurred in the uppers as well.

The crowns of the right PC7, PC9, and PC10 are relatively well preserved whereas those of PC11 and PC12 are damaged. The crowns of the right PC13 and PC14 are poorly represented. The left upper postcanines are only represented by the root of PC9, PC10, and PC11. The alveoli of the postcanines are deep and circular, lacking internal lateral or medial crests. Accordingly, the postcanines bear a single root, approximately circular in cross-section and without evidence of subdivision, that is not fused to the alveolar wall. The height of the root is approximately twice that of the crown. The crown of the postcanines are separated from each other and the teeth are not imbricated or interlocked.

The anteriormost postcanine preserved (PC7) is relatively compressed labiolingually when compared to the more posterior teeth, which become proportionally labiolingually wider and mesiodistally shorter posteriorly, with a triangular shape in occlusal aspect. The long axis of the crown is oriented mesiodistally in PC7 whereas it is mesiolingual-distolabially oriented in the more posterior teeth. The upper postcanines present a centrally placed, symmetrical main cusp with convex mesial and distal margins is flanked by smaller accessory cusps. All recovered postcanines share steep labial and lingual surfaces of the crown. The labial surface of the crown is strongly convex whereas, lingually, the mesial and distal margins of the main cusp are inflated and crest-like, defining a central concave area.

PC7 bears a main cusp with a long mesial margin and a shorter distal one. Mesiodistally aligned with the main cusp, there is a mesially projected bulging at the base of the crown that is interpreted as an accessory cusp. Separated from the main cusp by a shallow and broad valley, there is a small, apicodistally projected distal accessory cusp, placed lingually on the crown.

Only the main cusp of PC8 is preserved, deep inside the crypt. It shares the convex labial surface of other postcanines but a lingual central concavity is not observed, probably due to its poor preservation. As preserved, the main cusp appears to be comparatively more acute than in the other postcanines.

PC9 is a bit larger than PC7. The main cusp appears to be higher than in PC7 and PC10. The mesial margin of the main cusp is only slightly longer than the distal one. The

mesial and distal accessory cusps are lingually placed on the crown. In labial aspect, the mesial and distal accessory cusps are not as conspicuous as in PC7. Lingually, the bulged mesial and distal margins of the main cusp converge with the accessory cusps. Both accessory cusps are crest-like, mesiolingually and distolingually projected, and longer apicobasally than in PC7.

Both right and left PC10 are preserved. The right PC10 is lower and mesiodistally shorter than the preceding tooth, with a blunter main cusp. The left PC10 shows a higher, slender, and more acute main cusp than its right counterpart. The main cusp of the left PC10 is slenderer and only slightly lower than that of the right PC9. Similar to PC9, the main cusp of PC10 has a mesial margin that is only slightly longer than the distal one and is flanked by crest-like accessory cusps. The accessory cusps are smaller and more lingually placed than in the preceding tooth, being only barely observable labially, especially the mesial one. The concavity of lingual surface of the tooth is less pronounced than in PC9.

The right and left PC11 are preserved, showing a general morphology similar to that of PC10 and PC9 although some differences are recognized between these elements. In addition, the right and left PC11 differ in several traits that might result from preservation issues as they appear to represent equivalent elements from the same teeth generation. In PC11, the crown is lower and the main cusp is blunter than in more anterior teeth and the distal accessory cusp is placed relatively higher on the crown. The cusps in the right PC11 form a more acute triangle when compared to more anterior upper postcanines, with the accessory cusps more lingually placed and the mesial accessory cusp obscured from the labial view. This is not the case in the left PC11, in which the angle between the cusps is wider, more obtuse than in PC10 and similar to what is observed in PC9. In the left PC11, the mesial accessory cusp is clearly visible in labial view, similar to the condition in PC9. In the right PC11, the accessory cusps are not crest-like but relatively more robust when compared to the left PC11 or more anterior teeth.

The crown of the right PC12 is fractured, with some regions missing. From the preserved portion of PC12, it can be inferred that it was similar to the right PC11 but with a more acute, higher main cusp, probably due to the absence of wear. According to the size of the root of the last upper postcanine, it was not reduced but similar in size to the preceding teeth.

Lower teeth. The lower dentition preserves at least one incisor, one canine, and seven postcanines (2+?i/1c/7pc). It is noteworthy that the lower tooth row is very short when compared to the upper one. The last lower postcanine (pc7) would have occluded approximately between PC8 and PC9. In this scenario, the last five upper postcanines lacked a counterpart in the lower dentition. Additionally, the lower postcanines are larger (i.e., higher and mesiodistally longer) when compared to the preserved upper postcanines.

A single incisor is preserved on the anteriormost tip of the right and left dentaries, representing the same locus. These teeth are relatively small, lower than the postcanines, and with a single distally recurved cusp. The root is circular in cross section whereas the crown is somewhat labiolingually compressed. The lingual surface of the crown is steeper than the more convex labial surface. Posterior to this incisor, there is a short diastema.

A small root fragment is preserved anteriorly, midway between both dentaries. Due to its position, we interpret it as a lower anterior incisor, probably i1, although it is not possible to ascertain if it is a right or left element. This fragment is similar in shape and size to the incisors in place on the dentaries.

The first tooth posterior to the incisor is interpreted as the canine. Regrettably, only the root of the right canine is preserved. It is oval in cross-section and similar in size to that of the pc1, suggesting that the canine might have been a relatively small tooth, probably not much higher than the postcanines but larger than the incisor. There is no postcanine diastema.

On the right dentary, pc1, pc2, pc4, and pc5 and the roots of pc3 and pc6 are preserved. Posterior to the right pc6, there is an empty alveolus for the last lower postcanine (pc7), which has not been recovered. On the left dentary, pc1 and pc2 and the roots of pc3 and pc5 are present. Replacement of alternate teeth is recognized in both dentaries at the same time. The right pc1, pc3, pc5, and pc6 are fully erupted elements whereas pc2 and pc4 are still in their crypts. On the left dentary, pc1 is completely erupted and pc2 is in its crypt. All postcanines are of the same size. The alveolus for the pc7 does not seem to be reduced which lead us to infer that this tooth was similar in size to the more anterior elements. The postcanines are inserted in deep oval alveoli, whose base is very close to the ventral margin of the dentary. There are no crests on the internal lateral or medial walls of the alveoli. The postcanine roots are oval in cross-section and lack incipient division.

The first postcanine (pc1) bears a large, asymmetrical main cusp, with a convex mesial margin and a slightly concave distal one. This cusp occupies almost the entire

length of the crown and is not distally recumbent but straight. Distally, a small, distally pointing cusp is present. The base of the crown in the right pc1 is fractured; however, it appears that the base of the crown is somewhat inflated lingually, with both the main and the distal cusp are labially placed on the crown. An incipient distolingual bulging of the base of the crown is observed in the left pc1. The second postcanine (pc2) and pc4 present the same morphology. They show a recumbent main cusp with a longer convex mesial margin and a shorter, steeper, and only slightly convex to straight mesial one. This cusp occupies approximately 2/3 of the length of the crown. Separated by a relatively wide valley, a small distal cusp pointing apico-distally is present at half-length of the main cusp. A small distal bulging at the base of the crown represents a third cusp. The labial surface of the cusps is more convex than the lingual one, that is almost flat. The base of the crown is expanded lingually into a cingulum which is not defined by a crest and does not reach the mesial margin of the crown. Hence, the cusps are labially placed on the crown. The crown of pc5 is damaged and only the main cusp and the lingual cingulum are preserved. These structures and the general morphology of the crown in pc 5 are similar to that of pc2 and pc4.

4.b. Comparisons

A list of the references and specimens employed for the following comparisons are presented in Table S2.

Table S2. Main references employed for comparisons.

Taxon	Reference	Observations
Aleodon brachyrhamphus	Crompton, 1955; Abdala and Smith, 2009.	early Probainognathia
Aleodon cromptoni	Martinelli et al., 2017a.	early Probainognathia
Chiniquodon	Huene, 1936; Bonaparte, 1966; Romer, 1969; Romer and Lewis, 1973; Martínez and Foster, 1996; Abdala and Giannini, 2002; Kemp, 2007, 2009; Oliveira et al., 2009; Kammerer et al., 2010; Martinelli et al., 2017a; Mocke et al., 2020.	early Probainognathia
Diegocanis	Martínez et al., 2013.	early Probainognathia
Ecteninion	Martínez et al., 1996.	early Probainognathia
Lumkuia	Hopson and Kitching, 2001.	early Probainognathia
Probainognathus	Romer, 1970.	early Probainognathia

Trucidocynodon	Oliveira et al., 2010.	early Probainognathia
Botucaraitherium	Soares et al., 2014.	derived Probainognathia
Brasilodon	Bonaparte et al., 2003, 2005; Bonaparte, 2013; Rodrigues et al., 2014; Ruf et al., 2014.	derived Probainognathia
Chaliminia	Bonaparte, 1971; Martinelli and Rougier, 2007.	derived Probainognathia
Diarthrognathus	Crompton, 1958; Crompton, 1963; Gow, 1980, 1994.	derived Probainognathia
Elliotherium	Sidor and Hancox, 2006.	derived Probainognathia
Irajatherium	Martinelli et al., 2005, Oliveira et al., 2010.	derived Probainognathia
Morganucodon	Kermack et al., 1973, 1981; Jenkins and Parrington, 1976.	derived Probainognathia
Pachygenelus	Watson, 1913; Gow, 1980, 2001; Shubin et al., 1991; Allin and Hopson, 1992; Crompton and Luo, 1993; Hopson and Rougier, 1993; Wible and Hopson, 1993; Luo and Crompton, 1994.	derived Probainognathia
Prozostrodon	Barberena et al., 1987; Bonaparte and Barberena, 2001; Pacheco et al., 2017; Guignard et al., 2018.	derived Probainognathia
Pseudotherium	Wallace et al., 2019.	derived Probainognathia
Riograndia	Bonaparte et al., 2001; Soares et al., 2011.	derived Probainognathia
Sinoconodon	Patterson and Olson, 1961; Crompton and Sun, 1985; Crompton and Luo, 1993.	derived Probainognathia
Therioherpeton	Bonaparte and Barberena, 1975; Bonaparte and Barberena, 2001; Oliveira, 2006.	derived Probainognathia
Bienotherium	Chow, 1962; Hopson, 1964.	Tritylodontidae
Bienotheroides	Sun and Li, 1985; Sun, 1986; Sun and Cui, 1989; Maisch et al., 2004; Watabe et al., 2007.	Tritylodontidae
Bocatherium	Clark and Hopson, 1985.	Tritylodontidae
Dianzhongia	Cui, 1981.	Tritylodontidae
Kayentatherium	Sues, 1986; Sues and Jenkins, 2006.	Tritylodontidae
Oligokyphus	Kühne, 1956; Young, 1974; Sues, 1985.	Tritylodontidae
Tritylodon	Owen, 1884; Broom, 1910; Broili an Schroder, 1936; Ginsburg, 1962; Gow, 1986, 1991; Gaetano et al., 2017.	Tritylodontidae
Yunnania	Cui, 1976.	Tritylodontidae

Comparisons with Chalimina musteloides

Chaliminia musteloides is the only named cynodont represented by cranial remains previously described from the Los Colorados Formation. Hence, we present here a detailed comparison of the known specimens with the holotype of *Tessellatia bonapartei*.

Tessellatia is comparable in size with the two known specimens of *Chaliminia* (PVL3857 and PULR088). Although incomplete, the proportions of the snout of *Tessellatia* differ from those of *Chaliminia*. *Tessellatia* shows a proportionally longer snout when compared to the height of the skull at the level of the anterior margin of the orbit. The ratio between the height of the skull at the level of the anterior margin of the orbit and the length between the anterior margin of the orbit and the anterior end of the maxilla is 0.58 in *Tessellatia* and 1.06 in the holotype of *Chaliminia* (Table S3). The proportions of the dentary of *Tessellatia* also differ from those of the holotype of *Chaliminia*, with *Tessellatia* having a comparatively more slender dentary. The height to length ratio of the horizontal ramus of the dentary is 0.23 in *Tessellatia* whereas in the holotype of *Chaliminia* this ratio is 0.38. On the other hand, in the referred specimen of *Chaliminia* (PULR081), this ratio is very similar (0.22) to that of *Tessellatia* (Table S4). We are aware that the tips of the skull and lower jaws are missing in *Tessellatia*; a longer skull and dentary would mean an even longer snout and a slenderer horizontal ramus, accentuating the differences in these proportions with the specimens of *Chaliminia*.

Table S3. Measurements (in cm) of the snout of selected probainognathians.

	Skull height (at the level of the anterior margin of the orbit)	Snout length (from the anterior margin of the orbit to the anterior end of the maxilla)	Height to length ratio of the snout
Brasilodon (UFRGS-PV1043T)	0.74	1.46	0.51
Chaliminia (PVL3857)	1.39	1.31	1.06
Prozostrodon (UFRGS-PV0248T)	1.95	3.13	0.62
Pseudotherium (PVSJ882)	0.84	1.86	0.45
Riograndia (MCN-PV2264)	0.92	1.09	0.84
Tessellatia (PULR-V121)	1.08	1.86	0.58
Therioherpeton (MVP05.22.04)	0.75	1.13	0.66

Table S4. Measurements (in cm) of the dentary of selected probainognathians. *Measurements of *Morganucodon* after reconstruction in Kermack et al., 1973.

	Dentary height	Horizontal ramus of the dentary length	Height/length ratio
Brasilodon (UFRGS-PV0929T)	0.24	1.27	0.19
Brasilodon (UFRGS-PV1043T)	0.38	1.66	0.23
Chaliminia (PVL081)	0.4	1.82	0.22
Chaliminia (PVL3857)	0.72	1.88	0.38
*Morganucodon**	0.19	1.12	0.17
Pachygenelus (BP/1/4761)	0.53	1.86	0.28
Pachygenelus (BP/1/5110)	0.52	1.31	0.40
Pachygenelus (BP/1/5623)	0.7	1.82	0.38
Pachygenelus (BP/1/5691)	0.55	1.52	0.36
Protheriodon (UFRGS-PV0962T)	0.47	1.81	0.26
Prozostrodon (CAPPA/UFSM0123)	0.74	2.39	0.31
Prozostrodon (UFRGS-PV0248T)	0.55	3.47	0.16
Riograndia (MCN-PV2265)	0.49	1.39	0.35
Tessellatia (PULR-V121)	0.42	1.82	0.23

Tessellatia differs from *Chaliminia* in the presence of a constriction in the snout behind the canines that results in a somewhat expanded rostrum anteriorly. The bony secondary palate in *Tessellatia* ends well anterior to the anterior margin of the orbit, not reaching the end of the upper tooth row as it ends anteriorly to the antepenultimate upper postcanine (PC13). This contrast to the long bony secondary palate of *Chaliminia* that extends posterior to the anterior margin of the orbit and the last upper postcanine. In addition, the posterior margin of the palate in *Tessellatia* is strongly concave whereas it is almost straight in *Chaliminia*.

Tessellatia further differs from *Chaliminia* in having a notably short lower tooth row with the ascending coronoid process of the dentary well-posterior to the last lower postcanine, slightly sloping dorsally at the level of the orbits. Opposite to this, the high coronoid process of *Chaliminia* obscures the last lower postcanine in lateral view. Linked with this feature, in *Tessellatia* the masseteric fossa does not reach the level of the last lower postcanine, whereas it does in *Chaliminia*. There is a strong osseous platform lateral to the last lower postcanine producing a strong lateral ridge in the holotype of *Chaliminia* that is not observed in *Tessellatia*. *Chaliminia* lacks the broad groove between the lateral wall of the dentary and the postcanine alveoli observed in *Tessellatia*. Unlike the

semicircular, very well-developed, ventrally projected angular process of *Tessellatia*, *Chaliminia* has a relatively smaller, somewhat triangular-shaped angular process projecting posteroventrally and laterally (as observed in the left dentary of the holotype). In addition, the ventral margin of the dentary is sigmoidal in *Tessellatia* whereas it is straight to slightly convex in *Chaliminia*.

The small non-procumbent posterior and the also small anterior (interpreted as i1) lower incisors preserved in *Tessellatia* contrast with the relatively large, procumbent lower incisors observed in *Chaliminia*. The relative sizes of the upper and lower canines of *Tessellatia* are different from those of *Chaliminia*. In strong contrast with *Tessellatia*, the upper canine of *Chalimina* is comparatively small, only slightly larger than the upper postcanines. The lower canine of *Tessellatia* has approximately the same diameter than the first lower postcanine, as measured at the upper portion of the root, whereas the lower canine of *Chaliminia* is markedly larger than the postcanines. Unlike in *Chaliminia*, there is no lower post-canine diastema in *Tessellatia*.

The number of upper postcanines in *Chaliminia* is similar to the inferred count in *Tessellatia* (14 upper postcanines). In contrast to the seven lower postcanines of *Tessellatia*, it has been suggested that *Chaliminia* presented 13 lower postcanines in accordance to the number of upper postcanines (Bonaparte, 1980; Martinelli and Rougier, 2007). In the holotype of *Chaliminia*, there are 10 lower postcanines in the left dentary, which is missing the anterior portion.

Tessellatia shares with *Chaliminia* the general morphology of upper and lower postcanines. However, some differences are recognized. Unlike the convex labial and concave lingual surface of the upper postcanines of *Tessellatia*, in *Chaliminia* there is a central blunt crest separating mesial and distal depressions labially whereas the lingual surface is almost flat. Contrary to what is observed in *Tessellatia*, the lower postcanines of *Chaliminia* are mesiodistally shorter than the upper ones. In *Chaliminia*, the lower postcanines show more conspicuous, higher distal accessory cusps, more closely placed to the main cusp than in *Tessellatia*.

Comparisons with other probainognathians

In the following section we provide comparisons between *Tessellatia* and known non-mammalian probainognathians, including tritylodontids and basal mammaliaforms.

Tessellatia has the antorbital region, measured from the anterior margin of the orbit to the anterior end of the maxilla, notably longer than the height of the skull at the level

of the anterior margin of the orbit, with a ratio of 0.58. Similar ratios are observed in *Therioherpeton* (0.66) and *Prozostrodon* (0.62). *Brasilodon* (0.51) and *Pseudotherium* (0.45), which have relatively longer snouts when compared to the height at the levels of the orbits. This contrast with the ratios present in *Chaliminia* (1.1) and *Riograndia* (0.84), which have relatively shorter snout relatively to the height at the level of the orbit (Table S3).

Among relatively derived non-tritylodontid probainognathians, *Tessellatia* is similar to *Riograndia*, *Irajatherium*, *Pachygenelus monus*, *Prozostrodon*, *Pseudotherium*, and *Brasilitherium* (but not *Therioherpeton*, *Elliotherium*, and *Chaliminia*) in having the snout constricted behind the canines, resulting in a somewhat pin-shaped rostrum.

The length of the palate in *Tessellatia* is most similar to that observed in *Trucidocynodon* and comparable to that of *Diegocanis* and *Ecteninion*. Unlike this, in derived non-tritylodontid probainognathians (e.g., *Riograndia*, *Elliotherium*, *Chaliminia*, *Pachygenelus monus*, *Prozostrodon*, *Brasilodon*, *Brasilitherium*, and *Pseudotherium*), the secondary palate extends to the end of the tooth row or posterior to it. The only exception is *Therioherpetodon* in which the secondary palate reaches the level of the penultimate postcanine, a condition observed in the more basal probainognathians *Chiniquodon*, *Lumkuia*, and *Probainognathus*.

A ventrally bowed osseous secondary palate reaching the level of the crown of the upper postcanines is observed in *Elliotherium*, *Pachygenelus monus*, and *Chaliminia* and is also present in *Tessellatia*. On the other hand, this feature is lacking in *Therioherpeton*, *Prozostrodon*, *Brasilodon*, *Pseudotherium*, tritylodontids, and mammaliaforms (e.g., *Morganucodon*). This feature was identified as a diagnostic character of Tritheledontidae (see Martinelli and Rougier, 2007). However, the results of our phylogenetic analysis suggest it is more widely distributed (see phylogenetic discussion).

The contribution of the palatal process of the maxilla to the osseous secondary palate of *Tessellatia* is about equal to that of the palatine. This condition is shared with *Aleodon cromptoni*, *Prozostrodon*, *Pseudotherium*, and *Tritylodon*. A relatively long palatal process of the maxilla is recognized in *Elliotherium*, *Oligokyphus*, and most basal probainognathians (*Trucidocynodon*, *Diegocanis*, *Ecteninion*, *Probainognathus*, *Lumkuia*, and *Chiniquodon*) except for *Aleodon cromptoni* and *Aleodon branchyramphus*. The opposite condition (i.e., the palatine being longer than the maxilla in the osseous secondary palate) has been mentioned as a diagnostic feature of Ictidosauria (Martinelli and Rougier, 2007). According to the definition and cladogram

proposed by Martinelli and Rougier (2007), their Ictidosauria includes *Riograndia*, *Irajatherium*, *Chaliminia*, *Elliotherium*, *Pachygenelus*, *Diarthrognathus*, and *Tritheledon*. Among these taxa, this feature is not available for observation in known specimens of *Irajatherium*, *Chaliminia*, *Diarthrognathus*, and *Tritheledon*. According to our phylogenetic analysis, this character is ambiguous at the node Ictidosauria (see phylogenetic discussion). A long palatine contribution to the secondary palate is shared by *Aleodon branchyramphus*, *Therioherpeton*, *Riograndia*, *Pachygenelus monus*, and the tritylodontids *Bocatherium*, *Kayentatherium*, *Bienotheroides*, and *Bienotherium*.

Tessellatia differs from *Prozostrodon*, *Therioherpeton*, *Riograndia*, *Elliotherium*, *Diarthrognathus*, *Brasilodon*, *Pseudotherium*, *Kayentatherium*, *Sinoconodon*, and *Morganucodon* in presenting an orbital process of the palatine excluded from the orbital wall, lacking a contact with the frontal. This condition of *Tessellatia* is also observed in *Chiniquodon* and *Aleodon*. On the other hand, other relatively basal probainognathians such as *Ecteninion* and *Trucidocynodon*, share with more derived forms other than *Tessellatia* the participation of the orbital process of the palatine in the orbital wall, contacting the frontal and lacrimal. *Lumkuia* lacks an orbital process of the palatine.

Tessellatia lacks a maxillary platform lateral to the postcanine tooth row, which constitutes the plesiomorphic condition for probainognathians. On the other hand, *Riograndia*, *Elliotherium* and tritylodontids bear a maxillary platform.

In *Tessellatia*¸ the zygomatic arch is inferred to begin at the end or slightly posterior to the end of the upper tooth row. This condition is most comparable to that of *Riograndia*, *Prozostrodon*, *Therioherpeton*, *Brasilodon*, and *Pseudotherium*, in which the zygomatic arch begins posterior to the end of the upper tooth row. Hence, all upper postcanines are placed anterior to the subtemporal fossa, opposite to the condition in *Elliotherium*, *Chaliminia*, *Pachygenelus monus*, *Diarthrognathus*, and tritylodontids in which the zygomatic arch is placed well anteriorly and the postcanines are posterior to the anterior border of the subtemporal fossa. Martinelli and Rougier (2007) included the latter feature in the diagnosis of Chalimininae, integrated only by *Chaliminia* and *Elliotherium* according to their phylogenetic definition and cladogram. Our observations show that this morphology is more widely distributed phylogenetically than previously recognized.

A remarkably large facial exposure of the lacrimal is a distinctive feature of *Tessellatia*. Among probainognathians, this condition is only additionally recognized in some tritylodontids (e.g., *Bocatherium*, *Bienotheroides*). On the contrary, the lacrimal represents a relatively small proportion of the lateral wall of the snout in most

probainognathians (e.g., *Ecteninion*, *Trucidocynodon*, *Probainognathus*, *Lumkuia*, *Chiniquodon*, *Aleodon cromptoni*, *Prozostrodon*, *Therioherpeton*, *Riograndia*, *Brasilodon*, *Morganucodon*, *Pseudotherium*).

In *Tessellatia*, the frontals lack the long anterolateral projections observed in the derived probainognathians *Elliotherium*, *Riograndia*, *Pachygenelus monus*, *Brasilodon*, *Pseudotherium*, and *Kayentatherium*. In *Therioherpeton*, *Morganucodon*, and the tritylodontids *Bienotherium*, *Tritylodon*, *Yunnania*, and *Oligokyphus*, the frontals lack or have short anterolateral projections as in *Tessellatia*. In these taxa the lacrimal and frontal contact is short as observed in dorsal view. In more basal probainognathians such as *Ecteninion*, *Diegocanis*, *Trucidocynodon*, *Probainognathus*, *Lumkuia*, *Chiniquodon*, and *Aleodon*, the frontals do not bear anterolateral projections and the lacrimal-frontal contact in the interorbital region is precluded by the prefrontal. This latter condition is also observed in *Pseudotherium*.

Tessellatia shares the presence of a completely ossified maxillary canal, separated from the maxillary sinus with derived probainognathians including mammaliaforms (such as *Oligokyphus*, *Kayentatherium*, *Brasilodon*, *Tritylodon*, *Pseudotherium*, *Pachygenelus*, and *Morganucodon*; see Benoit et al., 2016, 2020). The short infraorbital canal and posteriorly placed infraorbital foramen of *Tessellatia*, shared with derived probainognathians, suggest the presence of a mobile rhinarium with sensitive vibrissae (see Benoit et al., 2016, 2020).

The anterior portion of the dentary is missing in the only known specimen of *Tessellatia*. The presence of the fragmentary tooth inferred to represent an anterior incisor (probably i1) together with what is preserved of the anterior portion of the snout suggest that the dentary was slightly longer than preserved. As preserved, the height to length ratio of the horizontal ramus of the dentary in *Tessellatia* (0.23), this ratio was probably lower considering that the dentary is incomplete anteriorly. Similar values for this ratio are observed in *Prozostrodon* holotype (0.16), *Morganucodon* (0.17), *Brasilodon* specimens UFRGS-PV0929T (0.19) and UFRGS-PV1043T (0.23) and *Chaliminia* referred specimen PVL081 (0.22). This ratio is higher in *Prozostrodon* specimen CAPPA/UFSM0123 (0.31), *Riograndia* (0.35), *Pachygenelus* specimens BP/1/5691 (0.36), BP/1/5623 (0.38), and BP/1/5110 (0.4), and in *Chaliminia* holotype PVL3857 (0.38). *Protheriodon* (ratio 0.26) and *Pachygenelus* specimen BP/1/4761 (ratio 0.28) show intermediate values for this ratio (Table S4).

The anterior region of the dentary is elevated regarding the postcanine line in *Tessellatia* as well as in many non-tritylodontid probainognathians (*Botucaraitherium*, *Prozostrodon*, *Irajatherium*, *Chaliminia*, *Pachygenelus monus*, *Diarthrognathus*, *Brasilodon*, and *Morganucodon*). Among derived, non-tritylodontid probainognathians, this condition in not present in *Riograndia*.

Tessellatia, *Prozostrodon*, *Brasilodon*, and *Morganucodon* share a relatively posteriorly placed masseteric fossa, not extending anteriorly to the level of the penultimate lower postcanine. Contrary to this, the masseteric fossa extends comparatively more anteriorly in *Irajatherium*, *Riograndia*, *Chaliminia*, *Pachygenelus monus*, and *Diarthrognathus*.

The shape and development of the angular process of the dentary of *Tessellatia* distinguishes it from other taxa except for one specimen of *Diarthrognathus* (uncatalogued specimen, Field Museum of Chicago), in which it is well-developed and semicircular. This process is well developed but more triangular in other *Diarthrognathus* specimens (Broom's Ictidosaurian NMQR 249 – Crompton, 1963 and BP/1/7968). In some specimens of *Pachygenelus monus* (BP/1/5691; BP/1/5623, Gow, 2001), the angular process of the dentary is prominent but not semicircular whereas in other specimens (SAM-PK-K1329, Gow, 1980; BP/1/4381) it is less developed. In *Morganucodon*, this process is smaller but notably conspicuous. *Prozostrodon*, *Riograndia*, *Irajatherium*, *Chaliminia*, *Botucaraitherium*, *Brasilodon*, and *Brasilitherium* lack a ventrally projected angular process of the dentary.

The presence of a large upper canine (1.5 or more the mesiodistal length of the first postcanine) in conjunction with a reduced lower one (the same mesiodistal length or less of the first postcanine) is only recorded in PULR-121 and *Riograndia* among non-mammaliaform cynodonts and shared with the basal mammaliaform *Morganucodon*. On the other hand, in *Prozostrodon*, *Pachygenelus monus*, and *Brasilodon* as well as in more basal probainognathians (*Trucidocynodon*, *Probainognathus*, *Lumkuia*, *Aleodon*, and *Chiniquodon*), both the upper and lower canines are enlarged.

In *Elliotherium*, *Therioherpeton*, and *Pseudotherium* and in the ecteniniids *Diegocanis* and *Ectenínion*, the upper canine is relatively large whereas the lower one is unknow. A well-developed paracanine fossa is recognized in *Diegocanis* and *Pseudotherium*, suggesting the presence of enlarged lower canines. However, it deserves mentioning that in *Pachygenelus monus* the paracanine fossa is absent despite the relatively large lower canines. In *Diarthrognathus*, the lower canine is relatively large but

the upper one is not preserved in any of the known specimens. Both lower and upper canines are reduced in *Chaliminia*.

A reduced number of lower postcanines regarding the number of upper ones is a distinctive feature of *Tessellatia* among non-mammaliaform cynodonts. Only in *Prozostrodon* a comparable scenario is recognized but, opposite to *Tessellatia*, in *Prozostrodon* the number of lower postcanines (9-10) is larger than of upper ones (7). *Tessellatia* bears 14 upper postcanines, a number similar to *Elliotherium* (13) and *Chaliminia* (13), a diagnostic feature of Chaliminianae (see Martinelli and Rougier, 2007). On the other hand, most prozostrodontians bear fewer upper postcanines (*Prozostrodon* [7], *Therioherpeton* [7], *Riograndia* [9], *Irajatherium* [6 observed, 7 inferred], *Pachygenelus monus* [7 or 10], *Diarthrognathus* [7 or 11], *Brasilodon* [8] *Pseudotherium* [9]). The number of lower postcanines in *Tessellatia* (7) is similar to that observed in *Irajatherium* (8), *Pachygenelus* (7), and *Brasilodon* (7), higher than in *Botucaraitherium* (4), and lower than in *Prozostrodon* (9-10), *Riograndia* (9), *Chaliminia* (13, inferred), and *Diarthrognathus* (9, 11 or 13).

Tessellatia presents the general structure of the upper and lower postcanines diagnostic of tritheledontids (see Martinelli and Rougier, 2007), being very similar to what is observed in *Elliotherium*, *Irajatherium*, *Chaliminia* (see above), and *Pachygenelus*. The upper postcanines bearing a main central cusp and poorly developed, lingually placed accessory mesial and distal cusps. The lower postcanines are characterized by a large main mesial cusp followed by poorly developed distal cusps. *Prozostrodon*, *Therioherpeton*, *Botucaraitherium*, *Brasilodon*, *Brasilitherium*, and basal mammaliaforms (e.g., *Morganucodon*) present labiolingually compressed upper and lower postcanines with mesiodistally aligned cusps, including a large main cusp flanked by one or more lower mesial and distal accessory cusps. The upper postcanines of *Pseudotherium* share this latter morphology but the lower ones are unknown. *Riograndia* shows leaf-shaped, multicusped, labiolingually compressed upper and lower postcanines.

The upper postcanines of *Elliotherium* are only labially exposed. The only significant difference that can be ascertained when compared to *Tessellatia* is that in *Elliotherium* the upper postcanines are relatively small, a trait shared with *Prozostrodon* and *Pseudotherium*. Despite the general similarities, *Irajatherium* differs from *Tessellatia* in having upper postcanines that increase in size posteriorly and bearing more labiolingually compressed regarding the mesiodistal length (except for the last upper postcanine in the holotype), mesial and distal accessory cusps larger and more conspicuous (in PC4-6) and

less lingually placed (in PC4 and PC5, but not in PC6), and mesial accessory cusp larger and placed higher on the crown than the distal accessory cusp (the opposite is observed in *Tessellatia*). Unlike *Tessellatia*, the lower postcanines of *Irajatherium* are relatively smaller and imbricated. They present a mesial accessory cusp, a smaller main cusp, higher and more conspicuous accessory cusps, and a mesiolingual cingulum bearing cuspules. A distolingual bulging or cingulum as that observed in *Tessellatia* is absent in the lower postcanines of *Irajatherium*. In addition, the upper and lower postcanines of *Irajatherium* have incipiently constricted root, a feature absent in *Tessellatia*.

In addition to the general morphology, *Tessellatia* shares with *Pachygenelus monus* presence of single-rooted postcanines and of anterior upper postcanines more labiolingually compressed than more posterior elements. On the other hand, unlike *Tessellatia*, the upper postcanines of *Pachygenelus monus* slightly increase in size posteriorly (Gow, 1980: figure 1 SAM K1329; BP/1/5691), the long axis of posterior upper postcanines is approximately parallel to the tooth row axis (Gow, 1980: figure 1 SAM-PK-K1329), and some upper postcanines have a labial cingulum (Gow, 1980: figure 1 SAM-PK-K1329). In contrast to *Tessellatia*, the lower postcanines of *Pachygenelus monus* bear a comparatively smaller main cusp and more conspicuous, relatively higher distal accessory cusps (the latter feature especially in posterior teeth). In addition, posterior lower postcanines of *Pachygenelus monus* have a mesial accessory cusp and some specimens (Gow, 1980: figure 2 SAM-PK-K1329) present a labial cingulum (absent in Gow, 1980: figure 4 BP/1/4381). Unlike in *Tessellatia*, a complete lingual cingulum is present (Gow, 1980: figure 4 BP/1/4381) or lacking entirely (Gow, 1980: figure 2 SAM-PK-K1329) in *Pachygenelus monus*. On the other hand, the presence of a distolingual cingulum is shared between *Tessellatia* and specimen SAM-PK-K1394 of *Pachygenelus monus* (Gow, 1980: figure 5B).

Unlike *Tessellatia*, in *Diarthrognathus* the upper postcanines are in contact to each other (Broom's Ictidosaurian NMQR 249 – Gow, 1980) and the long axis of each tooth is approximately parallel to the border of the maxilla (Broom's Ictidosaurian NMQR 249 – Gow, 1980). In addition, they lack a mesial accessory cusp (Broom's Ictidosaurian NMQR 249 – Gow, 1980) and present a mesial cingulum (Broom's Ictidosaurian NMQR 249 – Gow, 1980) and a flat lingual surface (not concave as in *Tessellatia*) (Broom's Ictidosaurian NMQR 249 – Gow, 1980). Furthermore, unlike in *Tessellatia*, the lower postcanines of *Diarthrognathus* are not labiolingually compressed (Broom's Ictidosaurian NMQR 249 – Gow, 1980; BP/1/7968; BP/1/4882 – Gow, 1994;

uncatalogued specimen, Field Museum of Chicago specimen) and the long axis of each tooth is oblique to transverse to the long axis of the dentary (Broom's Ictidosaurian NMQR 249 – Crompton, 1963; BP/1/7968; BP/1/4882 – Gow, 1994; Field Museum of Chicago specimen). In *Diarthrognathus*, the lower postcanines bear a relatively lower main cusp (Broom's Ictidosaurian NMQR 249 – Gow, 1980), accessory cusps relatively higher on the crown (Broom's Ictidosaurian NMQR 249 – Gow, 1980), and a strong lingual cingulum with cuspules (Broom's Ictidosaurian NMQR 249 – Gow, 1980).

4.c. Phylogenetic results

The analysis of the complete matrix resulted in 29304 most parsimonious trees (MPT) of 786 steps; whereas the second analysis resulted in 200 MPT of 782 steps. A majority rule consensus tree of the latter analysis is presented in figure S5.

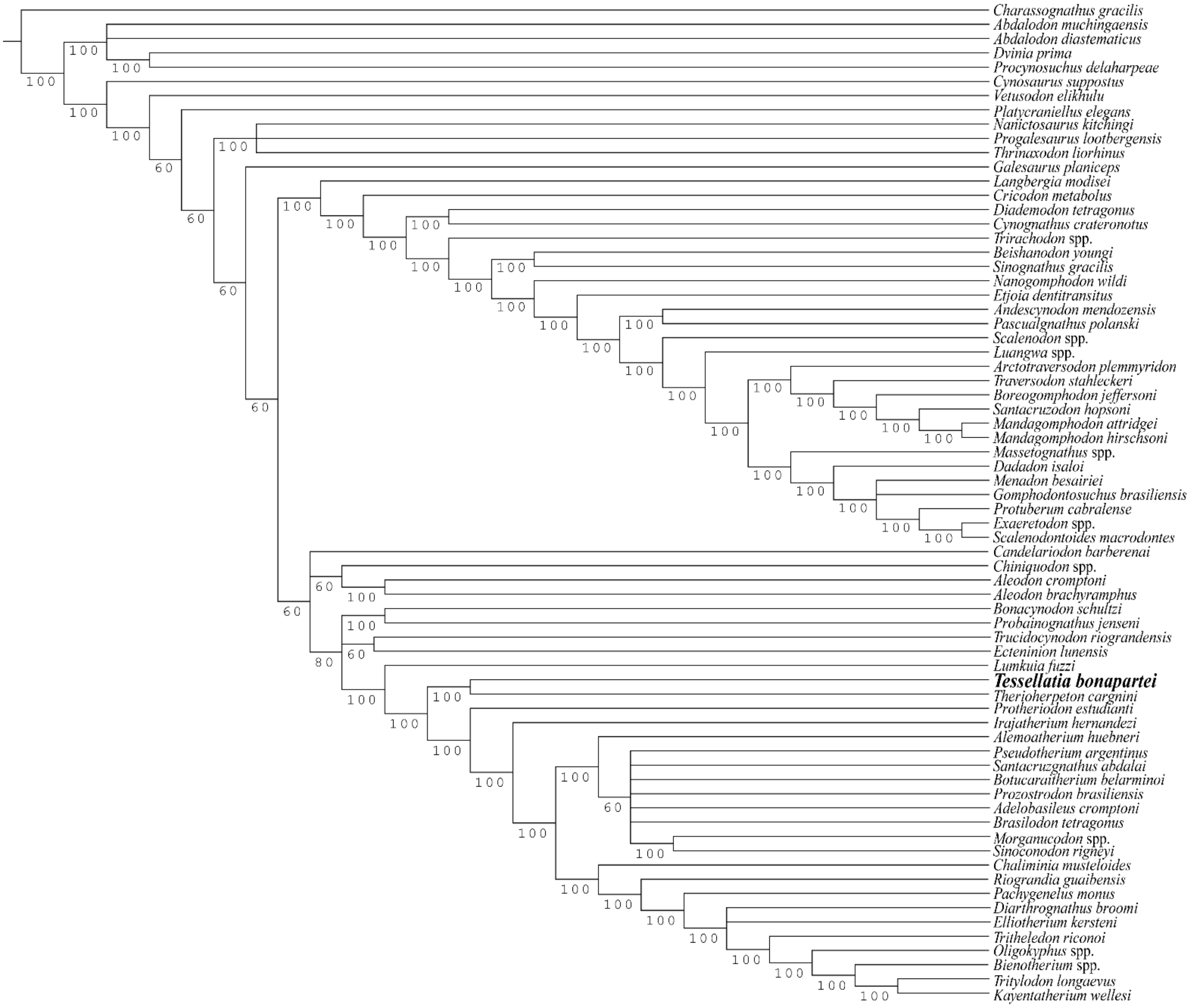

Figure S5. Majority rule consensus tree of the pruned (70-taxa) data matrix.

The synapomorphies of selected Probainognathia clades represented in the majority rule consensus tree follow:

Probainognathia, supported by 9 synapomorphies:

7. Parietal pineal foramen, absent (0>1); 9. Posterior extension of parietal, anterior or reaching the origin of the occipital crest (1>0); 49. Palatine, meets frontal but neither element contribute significantly to medial orbital wall (0>1); 55. Ectopterygoid, absent (1>2); 127. Anapophysis, absent (1>0); 129. Expanded costal plates on ribs, absent (1>0); 134. Procoracoid in glenoid, barely present or absent (0>1); 143. Dorsal profile of ilium, flat to concave (0>1); 145. Diameter of obturator foramen, greater than that of acetabulum (0>1)

Chiniquodontidae (*Chiniquodon*, *Aleodon*), supported by four synapomorphies:

2. Extranasal process of the premaxilla, large but not contacting the nasal (0>1); 47. Length secondary palate relative to anterior border of orbit, longer (0>2); 48. Osseous palate posterior extent in relation to upper tooth row, at same level or posterior (0>1); 52. Length of palatine relative to maxilla in secondary palate, longer (0>2).

Crown-ward Probainognathia minus chiniquodontids and *Candelariodon*, supported by five synapomorphies:

3. Septomaxilla facial process, long (1>0); 11. Frontal-epipterygoid contact, absent (1>0); 69. Pterygoparoccipital foramen, squamosal contributes to enclosure of foramen (0>1); 74. Lateral crest of dentary, absent (2>0); 141. Length of anterior process of ilium anterior to acetabulum relative to diameter of acetabulum, greater than 1.5 (1>2).

Bonacynododon, *Probainognathus*, supported by four synapomorphies:

13. Prootic canal, present (0>1); 19. Maximum dorsal extent of zygomatic arch, above middle of orbit but below upper border (0>1); 87. Upper tooth series extension, anterior to orbit (1>0); 102. Posterior postcanines with strongly curved main cusp, absent (1>0).

Trucidocynodon, *Ecteninion*, supported by three synapomorphies:

12. Epipterygoid ascending process, moderately expanded (2>1); 64. Cavum epiptericum, partial prootic floor (0>1); 97. Canine serrations, present (1>0).

Lumkuia crown-ward Probainognathia, supported by five synapomorphies:

9. Posterior extension of parietal, posterior to the origin of the occipital crest (0>1); 47. Length secondary palate relative to anterior border of orbit, about equal (0>1); 56. Interpterygoid vacuity in adults, present (1>0); 59. Quadrate rami of pterygoid, present

but do not contact quadrate (2>1); 78. Mediolateral thickening of anterior margin of coronoid process, present (0>1).

Crown-ward Probainognathia minus *Lumkuia*, supported by six synapomorphies:

6. Postorbital, absent (0>2); 17. Zygomatic arch dorsoventral height, slender (1>0); 28. Lateral expansion of braincase in parietal region, moderate (0>1); 49. Palatine, meets frontal and both elements contribute significantly to medial orbital wall (1>2); 96. Lower canine, reduced (0>1); 99. Postcanine occlusion, unilateral without forming a consistent pattern between upper and lower teeth (0>1); 102. Posterior postcanines with strongly curved main cusp, absent (1>0).

Tesselatia, *Therioherpeton*, supported by one synapomorphy:

114. Transverse axis of crown strongly oblique to middline axis, present (0>1).

Protheriodon crown-ward Probainognathia, supported by two synapomorphies:

10. Snout in relation to temporal region, longer (1>0); 48. Osseous palate posterior extent in relation to upper tooth row, at same level or posterior (0>1).

Mammaliamorpha, supported by one synapomorphy:

104. Lower postcanine roots, incipiently bifurcated (0>1).

Mammaliamorpha minus *Irajatherium*, supported by four synapomorphies:

62. Parasphenoid ala, absent (1>2); 69. Pterygoparaoccipital foramen, open as a notch (1>2); 147. Head of the femur, subspherical and inflected dorsally (0>1); 148. Greater trochanter separated from femoral head by distinct notch, present (0>1).

Alemoatherium crown-ward Mammaliaformes, supported by one synapomorphy:

88. Lower incisors, four or more (1>0).

Santacruzgnathus, *Botucaraitherium*, *Prozostrodon*, *Adelobasileus*, *Brasilodon*, *Morganucodon*, *Sinoconodon*, supported by one synapomorphy:

115. Lingual cingulum in lower postcanines, small, well developed (0>1 2)

Mammaliaformes, supported by 13 synapomorphies:

1. Internarial bar, absent (0>1); 12. Epipterygoid ascending process, moderately expanded (2>1); 35. Squamosal articulation for lower jaw, wide glenoid cavity ventrally directed (0>2); 36. Hyoid muscle fossa in paroccipital process, present and well developed (0>2); 42. Paracanine fossa in relation to the upper canine, anterior (1>0); 47. Length secondary palate relative to anterior border of orbit, longer (1>2); 51. Palatine, participate in subtemporal border by displacing pterygoid posteriorly (0>1); 56. Interpterygoid vacuity, absent (0>1); 64. Cavum epiptericum, partial prootic floor (0>1); 70. Lateral flange of prootic, includes vertical component so that flange is L shaped and

forms vertical wall adjacent to pterygoparoccipital foramen (0>1); 104. Upper postcanine roots, divided into two longitudinal aligned roots (1>2); 105. Lower postcanine roots, divided (1>2); 122. Postcanine replacement pattern, alternating delayed (0>1).

Chaliminia, *Riograndia*, *Pachygenelus*, *Diarthrognathus*, *Elliotherium*, *Tritheledon*, Tritylodontidae, supported by five synapomorphies:

9. Snout in relation to temporal region, shorter (0>2); 17. Anteroventral corner of zygomatic arch, significantly higher than postcanine line (0>1); 46. Length secondary palate relative to anterior border of orbit, longer (1>2); 52. Maxillary platform lateral to dentition, incipient in posterior portion of teeth row (0>1); 85. Craniomandibular articulation, higher than postcanine line (0>1).

Riograndia, *Pachygenelus*, *Diarthrognathus*, *Elliotherium*, *Tritheledon*, Tritylodontidae, supported by two synapomorphies:

57. Boss crest anterior to the interpterygoid vacuity, reduced or absent (1>0); 88. Upper incisors, fewer than four (1>2).

Pachygenelus, *Diarthrognathus*, *Elliotherium*, *Tritheledon*, Tritylodontidae, supported by three synapomorphies:

2. Extranasal process of the premaxilla, contacting nasal (0>2); 41. Incisive foramen, completely enclosed by premaxilla (2>3); 87. Upper tooth series extension, posterior to anterior border of subtemporal fenestra (1>2).

Diarthrognathus, *Elliotherium*, *Tritheledon*, Tritylodontidae, supported by seven synapomorphies:

8. Parietal region, high (0>1); 15. Prootic and opistothic, fused to form petrosal (0>1); 53. Maxillary platform lateral to dentition, well developed (1>2); 62. Parasphenoid ala, slightly reduced and excluded from fenestra ovalis (2>1); 63. Paroccipital process, contacts quadrate (0>1); 66. Fenestra rotunda and jugular foramen, confluent (1>0); 71. Anterior part of paroccipital process, lateral aspect exposed due to dorsal withdrawal of squamosal (0>1).

Tritheledon, Tritylodontidae, supported by two synapomorphies:

99. Postcanine occlusion, tooth to tooth contact because of widened postcanines (1>3); 101. Postcanine morphology (lowers and uppers), bucco-lingually expanded including multicuspidate with their cusps aligned in series (1>3)

Tritylodontidae, supported by three synapomorphies:

48. Osseous palate posterior extent in relation to upper tooth row, anterior (1>0); 104. Upper postcanine roots, multiple roots more than two (0>3); 121. Posteriormost postcanine gomphodont, present (0>1).

4.d. Probainognathian paleobiogeography

The Bayesian analysis indicates that the paleobiogeographic history of Probainognathia was dominated by dispersive events (30), followed by vicariance events (16), and with only one extinction event. However, eight dispersive events are, in fact, sympatric speciation events that Bayesian analysis in RASP miss-interprets as dispersions (see Seoane et al., 2017 for a discussion on this methodological problem). Sympatric speciation events occurred in nodes 55 (2), 54 (1), 51-48 (1), 44 (2), 39 (1), 38 (2), 37 (1), and 34 (2), where the daughter nodes or taxa were in the same ancestral area. In nodes where the reconstructed ancestral area is the union of more than one individual area and there is recognized a sympatric speciation event, an event is counted for each conforming area. In our study, the only fully-resolved node where this happens is node 34. According to our analysis, the most-probable ancestral area of node 34 is the region integrated by areas B and H. The Bayesian analysis proposes that there are three dispersions at this node. However, only two are necessary to explain its paleobiogeographic history as the ancestral area for node 33 is interpreted to be B and *Chiniquodon* is found in areas B and H but also in areas A and I. Hence, a more parsimonious interpretation is that there were two dispersions events: one to area A and the other to area I. In addition, the sympatric speciation event identified in node 34 (it cannot be interpreted as a vicariance event because the taxa share areas) must be counted as two events, one for each ancestral area (B and H). Finally, six vicariant events occurred in node 38 but only three events have biogeographic meaning whereas the others are best interpreted as overestimations. In summary, the paleobiogeographic history of Probainognathia can be explained by 22 dispersions, 13 vicariances, 18 sympatric speciation events, and one extinction (Fig. S6; Table S8).

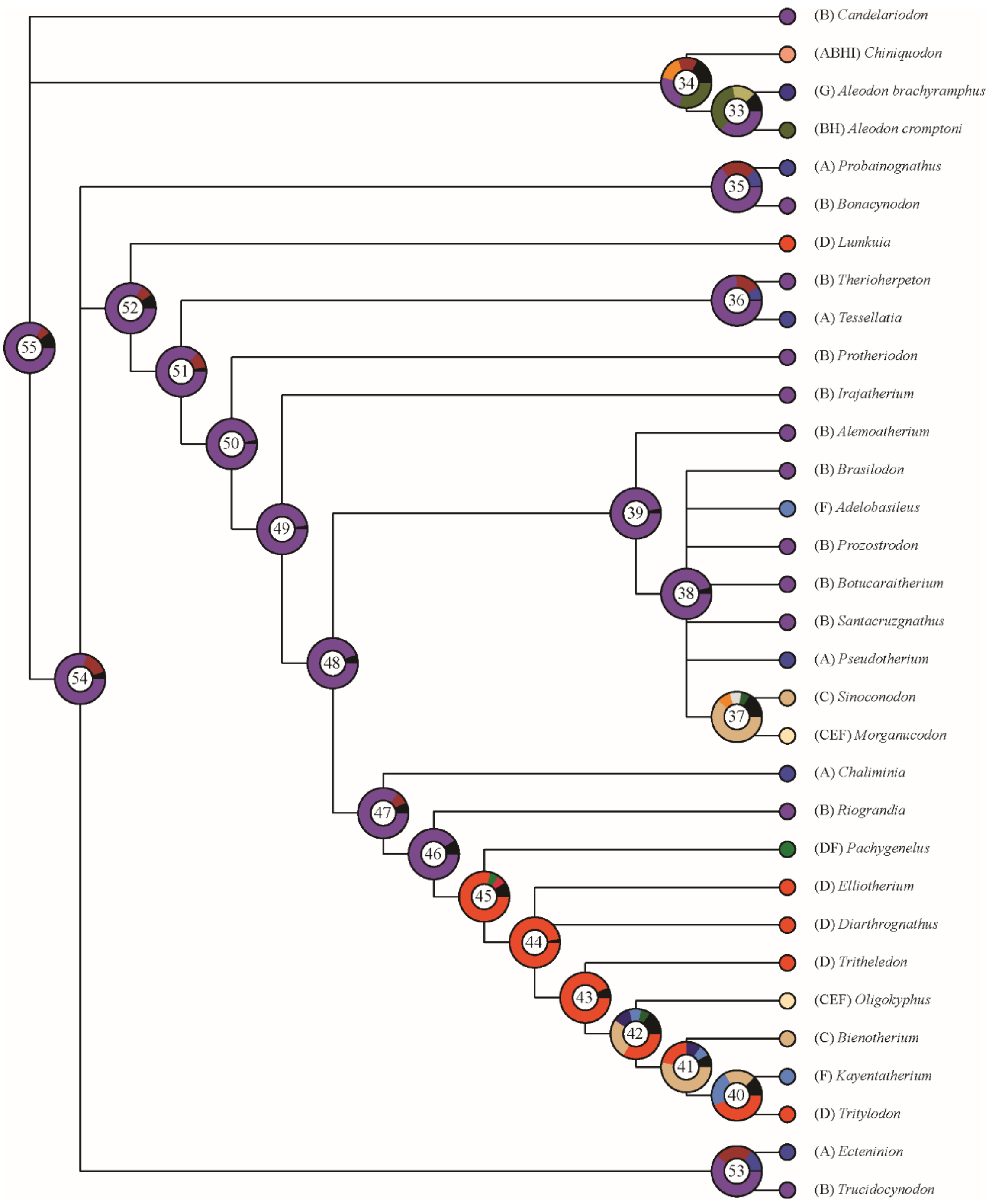

Figure S6. RASP analysis results depicting the probability of different ancestral areas for each node of the Probainognathia clade, as shown in the majority rule consensus tree from the pruned (70-taxa) phylogenetic analysis. Letters between brackets represent geographical distribution areas for each taxon: *A* Ischigualasto-Villa Unión Basin, *B* Paraná Basin, *C* Lufeng Basin, *D* Karoo Basin, *E* European basins, *F* North American basins, *G* Ruhuhu Basin, *H* Otiwarongo Basin, *I* Morondava Basin. The number and pie chart at each node represent the node number and the probability of different ancestral areas for each node. Colors represent different ancestral areas.

The BBM analysis reveals that Probainognathia (node 55) originated in the Paraná Basin (area B, 83.53%) and, in this area, there was a great and fast diversification, where 44% of the probainognathians was found. In the Paraná Basin, it took place the origin of the Mammaliamorpha (node 49, 97.71%) and its posterior diversification. From the Paraná Basin there were six dispersive events, three of them to the Ischigualasto-Villa Unión Basin (nodes 35–36 and 38), one to the North American basins (node 38), one to the Karoo Basins (node 46), and one to the Lufeng Basin (node 38). It is in the Lufeng Basin were Mammaliaformes originated (area C, 61.54%). The optimization methodology of Hausdorf indicates the same probable ancestral areas to Probainognathia (Table S5) and Mammaliamorpha (Table S6).

In the Karoo Basin there was other diversification of Mammaliamorpha, although smaller, with dispersion events to the North American basins (node 45) and the Lufeng Basin (node 42). According to the Bayesian analysis, Tritylodontidae originated in the Karoo Basin (node 42, 33.53%), but rapidly dispersed to the Lufeng, European, and North American basins, becoming extinct in the Karoo Basin. On the other hand, Hausdorf's optimization indicates that Tritylodontidae originated in the Lufeng Basin (Table S7) and, considering that the earliest tritylodontids were found in that area, this result seems more plausible. In addition, this latter interpretation requires fewer assumptions, as four events explain the origin of Tritylodontidae: a dispersion in node 43 from the Karoo Basin to the Lufeng Basin, followed by a vicariance event and two dispersions from the Lufeng Basin (node 42) to the European and North American basins. On the hand, the BBM analysis requires of five events. In node 43 occurred a sympatric speciation event, whereas in node 42 occurred the dispersion from the Karoo Basin to the Lufeng Basin followed by the extinction in the Karoo Basin, and later two dispersions occurred from the Lufeng Basin to the European and North American basins.

Vicariant events occurred in nodes 33, 35–36, 38 (seven events), 40–42, 46–47 and 52–53 (Fig. S6). The final resolution of node 38 depend on resolution of its internal relationships and the polytomy prevents a confident resolution. However, the ancestral area of node 38 is the Paraná Basin and only two daughter taxa and one daughter node have a different area of provenance, so the most conservative analysis indicates that three vicariant events are enough to explain its paleobiogeographic history. The other vicariant events occur at nodes where an ancestral population dispersed to a new area and at least one of the taxa or daughter nodes does not have the same geographic distribution as the

ancestor. This geographic separation after a dispersive event only can be explained by inferring some barrier.

TABLE S5. Estimations of ancestral areas for Probainognathia node (55), using Weighted Ancestral Area Analysis (WAAA, Hausdorf, 1998). GSW, number of weighted gain steps; LSW, number of weighted loss steps; PI, GSW/LSW, probability index for every area.

Areas	GSW	LSW	PI
A	1.1	3	0.37
B	3	2.09	1.44
C	0.19	3.07	0.06
D	0.51	3.08	0.17
E	0.18	3	0.06
F	0.46	3	0.15
G	0.33	3	0.11
H	1	2.33	0.43
I	0.5	3	0.17

TABLE S6. Estimations of ancestral areas for Mammaliamorpha node (49), using WAAA. Abbreviation in Table S2.

Areas	GSW	LSW	PI
A	0.78	2	0.39
B	1.75	1.25	1.4
C	0.39	2	0.2
D	0.35	2.14	0.16
E	0.33	2	0.17
F	0.88	2	0.44

TABLE S7. Estimations of ancestral areas for Tritylodontidae node (42), using WAAA. Abbreviation in Table S2.

Areas	GSW	LSW	PI
C	1.5	0.5	3
D	0.33	2	0.17
E	1	1	1
F	1.33	1	1.33

TABLE S8. Event matrix, event route, and probability for each node obtained by BBM analysis (the dispersion and vicariant events considered as valid are marked in parentheses in ‘Dispersion’ and ‘Vicariance’ columns).

	Event matrix					
Node	Dispersion	Vicariance	Extinction	Sympatry	Event route	Probability
33	2 (2)	1	0	0	B→BGH→G|BH	0.3629
34	3 (2)	0	0	B:1 H:1	BH→BH^B→ABHI^B→ABHI|B	0.1085
35	1 (1)	1	0	0	B→AB→A|B	0.6422
36	1 (1)	1	0	0	B→BA→B|A	0.7464
37	2 (2)	0	0	C:1	C→C^C→CEF^C→C|CEF	0.6154
38	7 (3)	6 (3)	0	B:2	B→B^B^B^B^B^B^B→BFAC^B^B^B^B^B^B→B|F|B|B|B|A|C	0.5901
39	0	0	0	B:1	B→B^B→B|B	0.9303
40	1 (1)	1	0	0	D→FD→F|D	0.4370
41	1 (1)	1	0	0	C→CD→C|D	0.2322
42	3 (3)	1	1	0	D→→^C→CEF^C→CEF|C	0.1782
43	0	0	0	D:1	D→D^D→D|D	0.3138
44	1 (0)	0	0	D:2	D→D^D^D^D→D|D|D	0.9164
45	1 (1)	0	0	D:1	D→D^D→DF^D→DF|D	0.7744
46	1 (1)	1	0	0	B→BD→B|D	0.7182
47	1 (1)	1	0	0	B→AB→A|B	0.7752
48	0	0	0	B:1	B→B^B→B|B	0.7849
49	0	0	0	B:1	B→B^B→B|B	0.9260
50	0	0	0	B:1	B→B^B→B|B	0.9516
51	0	0	0	B:1	B→B^B→B|B	0.6302
52	1 (1)	1	0	0	B→DB→D|B	0.7179
53	1 (1)	1	0	0	B→AB→A|B	0.6067
54	1 (0)	0	0	B:2	B→B^B^B^B→B|B|B	0.2563
55	2 (1)	0	0	B:2	B→B^B^B^B→BH^B^B^B→B|B|BH	0.1985
Total	30 (22)	16 (13)	1	18		

5. Comparison between Norian faunal assemblages with probainognathian cynodonts

The terrestrial Norian record of Probainognathia has its best representation in South America, particularly in the microvertebrate dominated *Riograndia* Assemblage Zone of the Caturrita Formation of southern Brazil producing four members of the group (*Brasilitherium riograndensis* and *Minicynodon maieri* are here considered junior

synonyms of *Brasilodon quadrangularis*, see Schultz et al., 2020). Three representatives of the group, *Riograndia guaibensis, Irajatherium hernandezi,* and *Brasilodon quadrangularis* are well represented in the fauna. The impressive record of Probainognathia in Brazil is complemented by taxa from the upper Carnian *Hyperodapedon* Assemblage Zone of the Santa Maria Formation. Other Norian faunas in South America are represented by the microvertebrate assemblage of Quebrada del Barro Formation in San Juan in which two morphotypes of undescribed small Probainognathia represent 70% of the faunal assemblage (Martínez et al., 2015). Other Norian Probainognathia are documented in the Los Colorados Formation, in which small Probainognathia are an extreme rare component of a fauna dominated by large dinosaurs.

The Norian Laurasian record of the Probainognathia is indeed more limited. In North America, the poorly understood dromatheriids *Dromatherium sylvestre* and *Microconodon tenuirostris* are documented by small mandibles with teeth and isolated teeth in levels of the lower Norian Cumnock Formation of North Carolina (Sues, 2001; Heckert et al., 2012). Different styles of assemblages are represented in this formation, with some dominated by large amphibian and phytosaurs and others, more recently excavated, microvertebrate assemblages extensively dominated by fish (Heckert et al., 2012). In the southwestern United States was recently described a new tiny peculiar cynodont, *Kaitaigidodon venetus*, of unknown relationships represented by two mandibular fragments with teeth from the middle Norian Blue Mesa Member of the Chinle Formation (Klingman et al., 2020). Previous record of isolated bones and teeth attributed to cynodonts (e.g., Kaye and Padian, 1994) were contested by Klingman et al. (2020). The assemblage PFV 456 (Thunderstorm Ridge) were material of *Kataigidodon* was found, is a very diverse microvertebrate fauna in which actinopterygian and coelacanth bones and scales likely make up the majority of the individual elements (Kligman, com. pers. 2021).

The other Norian Probainognathia from west U.S.A. is the tiny braincase of *Adelobasileus cromptoni*, interpreted as a basal mammaliaform from the Tecovas Formation of Texas (Lucas and Luo, 1993). The locality, termed upper Kalgary, is a diverse microvertebrate assemblage dominated by sphenodontians (Heckert, 2004).

The Ørsted Dal Formation of Greenland has probainognathians recorded in two faunas (Clemennsen et al., 2020): isolated teeth of mammaliaforms in the Carlsberg Fjord Member which have a well-represented record of sauropodomorphs and amphibians; and in the Tait Bjerg Member where only remains of small cynodonts are known: *Mitredon*

cromptoni, mammaliaform isolated teeth and skeleton remains of *Haramiyavia clemmenseni*. These faunas are usually interpreted as Rhaetian, but it was recently suggested that the entire Flaming Fjord Group, that include the Ørsted Dal Formation, is upper Norian (Kent and Clemmensen, 2021).

In strata exposed in the Lipie Śląskie clay-pit at Lisowice, Poland was discovered an isolated double rooted tooth of a mammaliaform identified as *Hallautherium* sp. (Świło et al., 2013). This fauna shows a mixture of macrofaunal remains dominated by a large dicynodont, whereas the microfaunal assemblage in which *Hallautherium* was retrieved is dominated by aquatic vertebrae (especially scales and teeth of Actinopterygii) (Świło et al., 2013). The age of the deposit is disputed but recent dating suggests a middle or late Norian age for the fossil assemblage (Kowal-Linka et al., 2019).

The terrestrial Norian fauna from the Lower Elliot Formation of South Africa is usually considered as more similar to that of the Los Colorados Formation due to the predominance of large sauropodomorphs, some of them closely related to the Argentinean forms (Viglietti et al., 2020). The provenance of the only record of a small Probainognathia in the South African fauna, *Elliotherium kersteni* (Sidor and Hancox, 2006), however, was more recently reinterpreted as coming more likely from lower levels of the Lower Jurassic Upper Elliot Formation because association with other fossils and rock types typical of that unit (Bordy et al., 2020).

6. References

Abdala, F. & Ribeiro, A. M. A new traversodontid cynodont from the Santa Maria Formation (Ladinian-Carnian) of southern Brazil, with a phylogenetic analysis of Gondwanan traversodontids. *Zool. J. Linn. Soc*. **139**, 529-545 (2003).

Abdala, F. Redescription of *Platycraniellus elegans* (Therapsida, Cynodontia) from the Lower Triassic of South Africa, and the cladistic relationships of eutheriodonts. *Palaeontology* **50**, 591-618 (2007).

Abdala, F. *et al*. The Triassic cynodonts from Argentina: a Gondwanan perspective. *J. S. Am. Earth Sci.* **104**, 102884; 10.1016/j.jsames.2020.102884 (2020).

Abdala, F. & Giannini, N. P. Chiniquodontid cynodonts: systematic and morphometric considerations. *Palaeontology* **45**, 1151-1170 (2002).

Abdala, F., Neveling, J. & Welman, J. A new trirachodontid cynodont from the lower levels of the Burgersdorp Formation (Lower Triassic) of the Beaufort Group, South

Africa and the cladistic relationships of Gondwanan gomphodonts. *Zool. J. Linn. Soc.* **147**, 383-413 (2006).

Abdala, F. & Smith, R. M. H. A Middle Triassic cynodont fauna from Namibia and its implications for the biogeography of Gondwana. *J. Vertebr. Paleont.* **29**, 837-851 (2009).

Allin, E. F. & Hopson, J. A. Evolution of the auditory system in Synapsida (“mammal-like reptiles” and primitive mammals) as seen in the fossil record in *The Evolutionary Biology of Hearing* (eds. Webster, D. B., Fay, R. R., Popper, A. N.) 587-614 (Springer-Verlag, 1992).

Apaldetti, C., Pol, D., Ezcurra, M. D. & Martínez, R. Sauropodomorph evolution across the Triassic-Jurassic boundary: body size, locomotion, and their influence on morphological disparity. *Sci. Rep.* **11**, 1-11 (2021).

Apaldetti, C., Martínez, R. N., Cerda, I. A., Pol, D. & Alcober, O. An early trend towards gigantism in Triassic sauropodomorph dinosaurs. *Nature ecology & evolution* **2**, 1227-1232 (2018).

Apaldetti, C.; Pol, D. & Yates, A. The postcranial anatomy of *Coloradisaurus brevis* (Dinosauria: Sauropodomorpha) from the Late Triassic of Argentina and its phylogenetic implications. *Palaeontology* **56**, 277-301; 10.1111/j.1475-4983.2012.01198.x (2013).

Arcucci, A. B. & Coria, R. A. A new Triassic carnivorous dinosaur from Argentina. *Ameghiniana* **40**, 217-228 (2003).

Arcucci, A. B., Marsicano, C. A., & Caselli, A. T. Tetrapod association and palaeoenvironment of the Los Colorados Formation (Argentina): a significant sample from Western Gondwana at the end of the Triassic. *Geobios* **37**, 557-568 (2004).

Bapst, D. W. Paleotree: an R package for paleontological and phylogenetic analyses of evolution. *Methods Ecol. Evol.* **3**, 803-807; 10.1111/j.2041-210X.2012.00223.x (2012).

Barberena, M. C., Bonaparte, J. F. & Teixeira, A. M. S. *Thrinaxodon brasiliensis* sp. nov., a primeira ocorrência de cinodontes galessauros para o Triassico do Rio Grande do Sul. *Anais do X Congresso Brasileiro de Geologia (Rio de Janeiro), Volume 1. 19th-25th of July 1987, Rio de Janeiro. Published by Sociedade Brasileira de Paleontologia*, 67-74 (1987).

Barghusen, H. R. On the evolutionary origin of the therian tensor veli palatini and tensor tympani muscles in *The Ecology and Biology of Mammal-like Reptiles* (eds. Hotton

III, N. H., MacLean, P., Roth J., Roth, E.) 256-262 (Smithsonian Institution Press, 1986).

Benoit, J., Manger, P. R. & Rubidge, B. S. Palaeoneurological clues to the evolution of defining mammalian soft tissue traits. *Sci Rep* **6**, 25604; https://doi.org/10.1038/srep25604 (2016).

Benoit, J. *et al*. The evolution of the maxillary canal in Probainognathia (Cynodontia, Synapsida): reassessment of the homology of the infraorbital foramen in mammalian ancestors. *J. Mammal. Evol.* **27**, 329-348 (2020).

Bonaparte, J. F. *Chiniquodon* von Huene (Therapsida, Cynodontia) en el Triásico de Ischigualasto, Argentina. *Acta Geológica Lilloana* **8**, 157-169 (1966).

Bonaparte, J. F.. Evolución de las vértebras presacras en Sauropodomorpha. *Ameghiniana* **36**, 115-187 (1999).

Bonaparte, J. F. & Púmares, J. A. Notas sobre el primer cráneo de *Riojasaurus incertus* (Dinosauria, Prosauropoda, Melanorosauridae) del Triásico Superior de La Rioja, Argentina. *Ameghiniana* **32**, 341-349 (1995).

Bonaparte, J. F., Ferigolo, J. F. & Ribeiro, A. M. A primitive Late Triassic "ictidosaur" from Rio Grande do Sul, Brazil. *Palaeontology* **49**, 931-936 (2001).

Bonaparte, J. F. Los tetrápodos del sector superior de la Formación Los Colorados, La Rioja, Argentina. *Opera Lilloana* **22**, 1-183 (1971).

Bonaparte, J. F. *Coloradia brevis* n. g. et n. sp. (Saurischia Prosauropoda), dinosaurio Plateosauridae de la Formación Los Colorados, Triásico Superior de la Rioja, Argentina. *Ameghiniana* **15**, 327-332 (1978).

Bonaparte, J. F. El primer ictidosaurio (Reptilia, Therapsida) de América del Sur, *Chaliminia musteloides*, del Triásico Superior de La Rioja. *II Congreso Argentino de Paleontología y Bioestratigrafía, Buenos Aires* **1**, 123-133 (1980).

Bonaparte, J. F. Los tetrápodos triásicos de Argentina. *Gondwana Stratigraphy, I. U.G.S. Coloquio Mar del Plata 1967*, 307-325 (1969).

Bonaparte, J. F. Annotated list of the south American Triassic tetrapods. *Proceedings and Papers: Second Gondwana Symposium, South Africa 1970*, 665-682. (1970).

Bonaparte, J. F. Evolution of the brasilodontidae (Cynodontia-Eucynodontia). *Hist. Biol.* **25**, 643-653 (2013).

Bonaparte, J. F. & Barberena, M. C. A possible mammalian ancestor from the Middle Triassic of Brazil. (Therapsida-Cynodontia). *J. Paleontol*. **49**, 931-936 (1975).

Bonaparte, J. F. & Barberena, M. C. On two advanced carnivorous cynodonts from the Late Triassic of Southern Brazil. *Bull. Mus. Comp. Zool.* **156**, 59-80 (2001).

Bonaparte, J. F., Ferigolo, J. & Ribeiro, A. M. A primitive Late Triassic "ictidosaur" from Rio Grande do Sul, Brazil. *Palaeontology* **44**, 623-635 (2001).

Bonaparte, J. F., Martinelli, A. G. & Schultz, C. L. New information on *Brasilodon* and *Brasilitherium* (Cynodontia, Probainognathia) from the Late Triassic of southern Brazil. *Rev. Bras. Palaontol.* **8**, 25-46 (2005).

Bonaparte, J. F., Martinelli, A. G., Schultz, C. L. & Rubert, R. The sister group of mammals: small cynodonts from the Late Triassic of southern Brazil. *Rev. Bras. Palaontol.* **5**, 5-27 (2003).

Bordy, E. M. *et al*. A chronostratigraphic framework for the upper Stormberg Group: implications for the Triassic-Jurassic boundary in southern Africa. *Earth Sci. Rev.* **20**, 103120 (2020).

Broili, F. & Schröder, J. Beobachtungen an Wirbeltieren der Karrooformation. XIX. Ein neuer Fund von *Tritylodon* Owen. *Sitzungsberichte der Bayerischen Akademie der Wissenschaften, Mathematisch-naturwissenschaftliche Abteilung* 187-256 (1936).

Broom, R. A comparison of the Permian reptiles of North America with those of South Africa. *Bull. Am. Mus. Nat. Hist.* **28**, 197-234 (1910).

Burger, M., Gilboa, G., Osher, S. & Xu, J. Nonlinear inverse scale space methods. *Commun. Math. Sci.* **4**,179-212 (2006).

Calzada, E., Gruenauer, F., Muehlbauer, M., Schillinger, B. & Schulz, M. New design for the ANTARES-II facility for neutron imaging at FRM II. *Nucl. Instrum. Methods Phys. Res. Sect. A. Accel. Spectrom. Detect. Assoc. Equip*. (2009)

Campione, N. E. Extrapolating body masses in large terrestrial vertebrates. *Paleobiology* **43**, 693-699; 10.1017/pab.2017.9 (2017).

Carminati, C. Strobl, M. & Kaestner, A. KipTool, a general purpose processing tool for neutron imaging data. *SoftwareX* **10**, 100279; 10.1016/j.softx.2019.100279 (2019).

Caselli, A. T., Marsicano, C. A. & Arcucci, A. B. Sedimentologia y paleontología de la Formacion Los Colorados, Triásico Superior (provincias de La Rioja y San Juan, Argentina). *Revista de la Asociación Geológica Argentina* **56**, 171-188 (2001).

Chow, M.-Z. A tritylodont specimen from Lufeng, Yunnan. *Vertebrata PalAsiatica* **6**, 365-367 (1962). [In Chinese].

Clark, J. M. & Hopson, J. A. Distinctive mammal-like reptile from Mexico and its bearing on the phylogeny of the Tritylodontidae. *Nature* **315**, 398-400 (1985).

Clemmensen, L. B., Kent, D. V., Mau, M., Mateus, O. & Milàn, J. Triassic lithostratigraphy of the Jameson Land basin (central East Greenland), with emphasis on the new Fleming Fjord Group. *Bull. Geol. Soc. Denmark* **68**, 95-132; 10.37570/bgsd-2020-68-05 (2020).

Colombi, C. *et al*. Bonebed en las facies basales de la Formación Los Colorados (Noriano), Cuenca de Ischigualasto-Villa Unión, San Juan, Argentina. *Resúmenes Reunión de Comunicaciones de la Asociación Paleontológica Argentina, Puerto Madryn* (2018).

Crompton, A. W. & Luo, Z.-X. Relationships of the Liassic mammals *Sinoconodon*, *Morganucodon oehleri*, and *Dinnetherium* in *Mammal Phylogeny: Mesozoic Differentiation, Multituberculates, Monotremes, Early Therians and Marsupials* (eds. Szalay, F. S., Novacek, M. J., McKenna, M. C.) 30-44 (Springer-Verlag, 1993).

Crompton, A. W. & Sun, A.-L. Cranial structure and relationships of the Liassic mammal *Sinoconodon*. *Zool. J. Linn. Soc.* **85**, 99-119 (1985).

Crompton, A.W. The cranial morphology of a new genus and species of ictidosaurian. *Proc. Zool. Soc. Lond.* **130**, 183-216 (1958).

Crompton, A. W. On some Triassic cynodonts from Tanganyika. *Proc. Zool. Soc. Lond.* **1125**, 617-669 (1955).

Crompton, A. W. On the lower jaw of *Diarthrognathus* and the origin of the mammalian lower jaw. *Proc. Zool. Soc. Lond*. **140**, 697-753 (1963).

Cui, G. *Yunnania*, a new tritylodontid from Lufeng, Yunnan. *Vert. PalAs.* **25**, 1-7 (1976) [In Chinese with English summary].

Cui, G. A new genus of Tritylodontidae. *Vert. PalAs.* **19**, 5-10 (1981) [InChinese with English summary].

Domnanovich, N. Revisión de los dicinodontes kannemeyéridos (Amniota, Therapsida) de Argentina, relaciones filogenéticas e implicancias paleobiogeográficas. *Universidad de Buenos Aires, Buenos Aires, Argentina. PhD Thesis* 387pp. (2010).

Ezcurra, M. D. & Novas, F. E. Phylogenetic relationships of the Triassic theropod Zupaysaurus rougieri from NW Argentina. *Hist. Biol.* **19**, 35-72 (2007).

Ezcurra, M. D. A new early coelophysoid neotheropod from the Late Triassic of northwestern Argentina. *Ameghiniana* **54**, 506-538 (2017).

Ezcurra, M. D. & Apaldetti, C. A robust sauropodomorph specimen from the Upper Triassic of Argentina and insights on the diversity of the Los Colorados Formation. *Proc. Geologist Assoc*. **123**: 155-164, 10.1016/j.pgeola.2011.05.002 (2011).

Gaetano, L. C. *et al*. 3D model related to the publication: A new cynodont from the Upper Triassic Los Colorados Formation (Argentina, South America) reveals a novel paleobiogeographic context for mammalian ancestors. *MorphoMuseumM* **8**,165; 10.18563/journal.m3.165 (2022).

Gaetano, L. C. *et al*. New cynodont specimens from Los Colorados Formation. *Publicación Electrónica de la Asociación Paleontológica Argentina* **19**, R50 (2019).

Gaetano, L. C., Abdala, F. & Govender, R. The postcranial skeleton of the Lower Jurassic *Tritylodon longaevus* from Southern Africa. *Ameghiniana* **54**, 1-35 (2017).

Gaetano, L. C. & Abdala, F. Analysis of the stapes of gomphodont cynodonts: intraspecific and interspecific variation. *PloS One* **10**, e0131174; 10.1371/ journal.pone.0131174 (2015).

Gao, K.-Q., Fox, R. C., Zhou, C.-F. & Li, D.-Q. A new nonmammalian eucynodont (Synapsida: Therapsida) from the Triassic of Northern Gansu Province, China, and its biostratigraphic and biogeographic implications. *Am. Mus. Novit*. **3685**, 1-25 (2010).

Ginsburg, L. *Likhoelia ellenbergeri*, tritylodonte du Trias supérieur du Basutoland (Afrique du Sud). *Annales de Paléontologie* **48**, 177-194 (1962).

Goloboff, P. & Catalano, S. TNT version 1.5, including a full implementation of phylogenetic morphometrics. *Cladistics* **32**, 221-238 (2016).

Goloboff, P., Farris, J. S. & Nixon, K. TNT, a free program for phylogenetic analysis. *Cladistics* **24**, 774-786 (2008).

Gow, C. E. The dentitions of the Tritheledontidae (Therapsida; Cynodontia). *Proc. R. Soc. London B* **208**, 461-482 (1980).

Gow, C. E. The side wall of the braincase in cynodont therapsids, and a note on the homology of the mammalian promontorium. *S. Afr. J. Zool.* **21**, 136-148 (1986).

Gow, C. E. Vascular system associated with the sidewall of the braincase and the prootic canals of cynodonts, including mammals. *S. Afr. J. Zool.* **26**, 140-144 (1991).

Gow, C. E. New finding od *Diarthrognathus* (Therapsida; Cynodontia) after seventy years. *Palaeontologia Africana* **31**, 51-54 (1994).

Gow, C. E. A partial skeleton of the tritheledontid *Pachygenelus* (Therapsida: Cynodontia). *Palaeontol. Africana* **37**, 93-97 (2001).

Guignard, M. L., Martinelli, A. G. & Soares, M. B Reassessment of the postcranial anatomy of *Prozostrodon brasiliensis* and implications for postural evolution of non-mammaliaform cynodonts, *Journal of Vertebrate Paleontology*, e1511570; 10.1080/02724634.2018.1511570 (2018).

Gulbranson, E. L. *et al*. Paleoenvironments and age of the Talampaya Formation: the Permo-Triassic boundary in northwestern Argentina. *J. S. Am. Earth Sci.* **63**, 310-322; https://doi.org/10.1016/j.jsames.2015.08.008 (2015).

Hahn, G. Zum Bau des infraorbital-foramens bei den Paulchoffatiidae (Multituberculata, Ober Jura). *Berliner Geowiss. Abh. A* **60**, 5-27 (1985).

Hausdorf, B. Weighted area analysis and a solution of the redundant distribution problem. *Syst. Biol.* **47**, 445-456 (1998).

Heckert, A. B. Mitchell, J. S., Schneider, V. P. & Olsen, P. E. Diverse new microvertebrate assemblage from the Upper Triassic Cumnock Formation, Sanford Subbasin, North Carolina, USA. *J. Paleontol*. **86**, 368-390 (2012).

Heckert, A. B. Late Triassic microvertebrates from the lower Chinle Group (Otischalkian-Adamanian:Carnian), southwestern U.S.A. *New Mexico Mus. Nat. Hist. Sci. Bull.* **27**, 1-170; 10.1098/rsbl.2020.0631 (2004).

Hopson, J. A. & Barghusen, H. An analysis of therapsid relationships in *The Ecology and Biology of Mammal-like Reptiles* (eds. Hotton, N., MacLean, P. D., Roth, J. J., Roth, E. C.) 83-106 (Smithsonian Institution Press, 1986).

Hopson, J. A. & Kitching, J. W. A probainognathian cynodont from South Africa and the phylogeny of non-mammalian cynodonts. *Bull. Mus. Comp. Zool.* **156**, 5-35 (2001).

Hopson, J. A. The braincase of the advanced mammal-like reptile *Bienotherium. Postilla* **87**,1-30 (1964).

Hopson, J. A. & Rougier, G. W. Braincase structure in the oldest known skull of a therian mammal: implications for mammalian systematics and cranial evolution. *Am. J. Sci.* **293**, 268-299 (1993).

Huene von, F. Die fossilen Reptilien des südamerikanischen Gondwanalandes. Ergebnisse der Sauriergrabungen in Südbrasilien 1928-29. Lieferung 2, Tübingen: Franz F. Heine, 93-159. (1936)

Jenkins, F. A., Jr. & Parrington, F. R. The postcranial skeletons of the Triassic mammals *Eozostrodon*, *Megazostrodon* and *Erythrotherium*. *Philos. T. R. Soc. Lond.* **273**, 387-431 (1976).

Kaestner, A. & Carminati, C. Neutronimaging/KipTool: First official release of KipTool. http://dx.doi.org/10.5281/zenodo.2578798 (2019).

Kaestner, A. P. & Schulz, M. Processing neutron imaging data – quovadis? *Physcs. Proc.* **69**, 336-342; 10.1016/j.phpro.2015.07.047 (2015).

Kammerer, C. F., Flynn, J. J., Fanivoharimanana, L. & Wyss, R. R. New material of *Menadon besairiei* (Cynodontia: Traversodontidae) from the Triassic of Madagascar. *J. Vert. Paleontol.* **28**, 445-462 (2008).

Kammerer, C. F., Flynn, J. J., Ranivoharimanana, L. & Wyss, R. R. Ontogeny in the Malagasy traversodontid *Dadadon isaloi* and a reconsideration of its phylogenetic relationships *Fieldiana: Life Earth Sci.* **5**, 112-125 (2012).

Kammerer, C. F., Flynn, J. J., Ranivoharimanana, L. & Wyss, A. R.. The first record of a probainognathian (Cynodontia: Chiniquodontidae) from the Triassic of Madagascar. *J. Vertebr. Paleont.* **30**, 1889-1894 (2010).

Kaye, F. & Padian, K. Microvertebrates from the Placerias Quarry: a window on Late Triassic vertebrate diversity in the American Southwest in *In the shadow of the dinosaurs: early Mesozoic tetrapods* (eds. Fraser, N. C., Sues, H.-D.) 171-196 (Cambridge University Press, 1994).

Kemp, T. S. Acoustic transformer function of the postdentary bones and quadrate of a nonmammalian cynodont. *J. Vertebr. Paleont*. **27**, 431-441 (2007).

Kemp, T. S. The endocranial cavity of a nonmammalian eucynodont, *Chiniquodon theotenicus*, and its implications for the origin of the mammalian brain. *J. Vertebr. Paleont*. **29**, 1188-1198 (2009).

Kent, D. V. & Clemmensen, L. B. Northward dispersal of dinosaurs from Gondwana to Greenland at the mid-Norian (215-212 Ma, Late Triassic) dip in atmospheric $p\mathrm{CO}_2$. *PNAS* **118**, e2020778118; 10.1073/pnas.2020778118 (2021).

Kent, D. V., Santi Malnis, P., Colombi, C. E., Alcober, O. A. & Martínez, R. Age constraints on the dispersal of dinosaurs in the Late Triassic from magnetochronology of Los Colorados Formation (Argentina). *PNAS* **111**, 7958-7963 (2014).

Kermack, K. A., Mussett, F. & Rigney, H. W. The lower jaw of *Morganucodon*. *J. Linn. Soc. (Zool.)* **53**, 87-175 (1973).

Kermack, K. A., Mussett, F. & Rigney, H. W. The skull of *Morganucodon*. *Zool. J. Linnean Soc.* **71**, 1-158 (1981).

Kligman, B. T., Marsh, A. D., Sues, H.-D. & Sidor, C. A. A new non-mammalian eucynodont from the Chinle Formation (Triassic: Norian), and implications for the early Mesozoic equatorial cynodont record. *Biol. Lett.* **16**, 20200631; 10.1098/rsbl.2020.0631 (2020).

Kowal-Linka, M., Krzemińska, E. & Czupyt, Z. The youngest detrital zircons from the Upper Triassic Lipie Śląskie (Lisowice) continental deposits (Poland): Implications for the maximum depositional age of the Lisowice bone-bearing horizon. *Palaeogeogr. Palaeocl. Palaeoec.* **514**, 487-501 (2019).

Kühne, W.G. The Liassic therapsid *Oligokyphus*. British Museum of Natural History, London, 149 p. (1956).

Laaß, M. & Schillinger, B. Reconstructing the auditory apparatus of therapsids by means of neutron tomography. *Physcs. Proc.* **69**, 628-635 (2015).

Leardi, J. M., Yañez, I. & Pol, D. South American crocodylomorphs (Archosauria; Crocodylomorpha): a review of the early fossil record in the continent and its relevance on understanding the origins of the clade. *J. S. Am. Earth Sci.* **104**, 102780; 10.1016/j.jsames.2020.102780 (2020).

Liu, J. & Olsen, P. The phylogenetic relationships of Eucynodontia (Amniota: Synapsida). *J. Mammal Evol.* **17**, 151-176 (2010).

López Gamundi, O. R. *et al.* Cuencas intermontanas in *Cuencas Sedimentarias Argentinas* (eds. Chebli, G., Spalletti, L.) 123–167 (Instituto Superior de Correlación Geológica, Serie Correlación Geológica, N°6, 1989).

Lucas, S. G. & Luo, Z.-X. *Adelobasileus* from the Upper Triassic of West Texas: the oldest mammal. *J. Vertebr. Paleontol.* **13**, 309-334 (1993).

Luo. Z.-X. & Crompton, A. W. Transformation of the quadrate (incus) through the transition from non-mammalian cynodonts to mammals. *J Vertebr Paleontol*. **14**, 341-374 (1994).

Luo, Z.-X., Kielan-Jaworowska, Z. & Cifelli, R. L. In quest for a phylogeny of Mesozoic mammals. *Acta Paleont. Pol.* **47**, 1-78 (2002).

Luo, Z.-X. Sister-group relationships of mammals and transformations of diagnostic mammalian characters in: *In the Shadow of the Dinosaurs* (eds. Fraser, N. C., Sues, H.-D.) 98-128 (Cambridge University Press, 1994).

Maisch, M. W., Matzke, A. T. & Sun, G. A new tritylodontid from the Upper Jurassic Shishugou Formation of the Junggar Basin (Xinjiang, NW China). *J. Vertebr. Paleontol.* **24**, 649-656 (2004).

Marsicano, C. A., Irmis, R. B., Mancuso, A. C., Mundil, R. & Chemale, F. The precise temporal calibration of dinosaur origins. *PNAS* **113**, 509-513 (2016).

Martinelli, A. G., Bonaparte, J. F., Schultz, C. L. & Rubert, R.. A new tritheledontid (Therapsida, Eucynodontia) from the Late Triassic of Rio Grande do Sul (Brazil) and its phylogenetic relationships among carnivorous non-mammalian eucynodonts. *Ameghiniana* **42**, 191-208 (2005).

Martinelli, A. G. *et al.* The African cynodont *Aleodon* (Cynodontia, Probainognathia) in the Triassic of southern Brazil and its biostratigraphic significance. *PLoS ONE* **12**, e0177948; 10.1371/journal.pone.0177948 (2017a)

Martinelli, A. G., Eltink, E., Da-Rosa, Á. A. S. & Langer, M. C. A new cynodont (Therapsida) from the *Hyperodapedon* Assemblage Zone (upper Carnian-Norian) of southern Brazil improves the Late Triassic probainognathian diversity. *Pap. Palaeontol.* **3**, 401-423 (2017b).

Martinelli, A. G. & Rougier, G. W. On *Chaliminia musteloides* (Eucynodontia: Tritheledontidae) from the Late Triassic of Argentina, and a phylogeny of Ictidosauria. *J. Vertebr. Paleont.* **27,** 442-460 (2007).

Martinelli, A. G., Soares, M. B. & Schwanke, C. Two new cynodonts (Therapsida) from the middle-early Late Triassic of Brazil and comments on South American probainognathians. *PloS One* **11**, e0162945; 10.1371/journal.pone.0162945 (2016).

Martínez, R. N., May, C. L. & Forster, C. A. A new carnivorous cynodont from the Ischigualasto Formation (Late Triassic, Argentina), with comments on eucynodont phylogeny. *J. Vertebr. Paleont*. **16**, 271-284 (1996).

Martínez, R. N., Fernández, E. & Alcober, O. A.A new non-mammaliaform eucynodont from the Carnian-Norian Ischigualasto Formation, northwestern Argentina. *Rev. Bras. Paleontol.* **16**, 61-76; 10.4072/rbp.2013.1.05 (2013).

Martínez, R. N., Alcober, O. A. & Pol, D. A new protosuchid crocodyliform (Pseudosuchia, Crocodylomorpha) from the Norian Los Colorados Formation, northwestern Argentina. *J. Vertebr. Paleont.* **38**, e1491047; 10.1080/02724634.2018.1491047 (2019).

Martínez, R. N., Alcober, O. A. Heredia, G. & Colombi, C. E. Nuevos prosaurópodos de la Formación Los Colorados (Triásico Superior-Noriano), La Rioja, Argentina. *Ameghiniana* **41**, 55R (2004).

Martínez, R. N. *et al.* A new Late Triassic vertebrate assemblage from northwestern Argentina. *Ameghiniana* **52**, 379-390 (2015).

McPhee, B. W., Benson, R. B., Botha-Brink, J., Bordy, E. M. & Choiniere, J. N. A giant dinosaur from the earliest Jurassic of South Africa and the transition to quadrupedality in early sauropodomorphs. *Current Biology* **28**, 3143-3151 (2018).

McPhee, B. W., Bordy, E. M., Sciscio, L., Choiniere, J. N. The sauropodomorph biostratigraphy of the Elliot Formation of southern Africa: tracking the evolution of sauropodomorpha across the triassic-jurassic boundary. *Acta Palaeontol. Pol*. **62**, 441-465 (2017).

Milana, J. P. & Alcober, O. Modelo tectosedimentario de la cuenca triásica de Ischigualasto (San Juan, Argentina). *Revista de la Asociación Geológica Argentina* **49**, 217-235 (1994).

Mocke, H., Gaetano, L. C. & Abdala, F. A new species of the carnivorous cynodont *Chiniquodon* (Cynodontia, Chiniquodontidae) from the Namibian Triassic. *J. Vertebr. Paleont.* **39**, e1754231; 10.1080/02724634.2019.1754231 (2020).

Nesbitt, S. J., Smith, N. D., Irmis, R.B., Turner, A.H., Downs, A. & Norell, M. A. A complete skeleton of a Late Triassic Saurischian and the Early Evolution of Dinosaurs. *Science* **326**, 1530-1533 (2009).

Oliveira, E. V. Reevaluation of *Therioherpeton cargnini* Bonaparte & Barberena, 1975 (Probainognathia, Therioherpetidae) from the Upper Triassic of Brazil. *Geodiversitas* **28**, 447-465 (2006).

Oliveira, T. V. D., Soares, M. B. & Schultz, C. L. *Trucidocynodon riograndensis* gen. nov. et sp. nov. (Eucynodontia), a new cynodont from the Brazilian Upper Triassic (Santa Maria Formation). *Zootaxa* **2832**, 1-71 (2010).

Oliveira, T. V., Schultz, C. L. & Soares, M. B. A partial skeleton of *Chiniquodon* (Cynodontia, Chiniquodontidae) from the Brazilian Middle Triassic. *Revista Brasileira de Paleontologia*, **12**, 113-122 (2009).

Otero, A. *et al*. Nuevos aportes al conocimiento de la fauna de tetrápodos de la formación los Colorados (Triásico Superior, Argentina): aspectos tafonómicos y paleobiogeográficos. *2° Simposio Internacional de Paleontología del Perú* (2019).

Owen, R. On the skull and dentition of a Triassic mammal (*Tritylodon longaevus*) from South Africa. *Q. J. Geol. Soc. Lond.* **40**, 146-152 (1884).

Pacheco, C. P., Martinelli, A. G., Pavanatto, A. E. B., Soares, M. B. & Dias-da-Silva, S. *Prozostrodon brasiliensis*, a probainognathian cynodont from the Late Triassic of Brazil: second record and improvements on its dental anatomy, *Hist. Biol*. 10.1080/08912963.2017.1292423 (2017).

Patterson, B. & Olson, E. C. A triconodontid mammal from the Triassic of Yunnan. *International Colloquium on the Evolution of Lower and Non-specialized Mammals:* 129-191. Brussels: Koninklijke Vlaamse Academiie voor Wetenschappen, Letteren en Schone Kunsten van Belgie (1961).

Pol, D. & Powell, J. New information on *Lessemsaurus sauropoides* (Dinosauria: Sauropodomorpha) from the Upper Triassic of Argentina. *Spec. Pap. Palaeontol.* **77**, 223-243 (2007).

R Core Team. R: A language and environment for statistical computing. R Foundation for Statistical Computing, Vienna, Austria. URL. https://www.R-project.org/ (2021).

Rasband, W.S. ImageJ, U. S. National Institutes of Health, Bethesda, Maryland, USA, https://imagej.nih.gov/ij/ (1997-2018).

Reichel, M., Schultz, C. & Soares, M. B. A new traversodontid cynodont (Therapsida, Eucynodontia) from the Middle Triassic Santa Maria formation of Rio Grande do Sul, Brazil. *Palaeontology* **52**, 229-250 (2009).

Rodrigues, P. G., Ruf, I. & Schultz, C. L. Study of a digital cranial endocast of the non-mammaliaform cynodont *Brasilitherium riograndensis* (Later Triassic, Brazil) and its relevance to the evolution of the mammalian brain. *Paläontol. Z.* **88**, 329-352 (2014).

Romer, A. S. & Lewis, A. D. The Chañares (Argentina) Triassic reptile fauna. XIX. Postcranial materials of the cynodonts *Probelesodon* and *Probainognathus*. *Breviora* **407**, 1-26 (1973).

Romer, A. S. The Chañares (Argentina) Triassic reptile fauna. VI. A chiniquodontid cynodont with an incipient squamosal-dentary jaw articulation. *Breviora* **344**, 1-18 (1970).

Romer, A. S. Cynodont reptile with incipient mammalian jaw articulation. *Science* **166**, 881-882 (1969).

Romer, A. S. & Jensen, J. A. The Chañares (Argentina). Triassic Reptile Fauna II. Sketch of the Geology of the Rio Chañares–Río Gualo region *Breviora* **252**, 1-20 (1966).

Ronquist, F. & Huelsenbeck, J. P. MrBayes3: Bayesian phylogenetic inference undermixed models. *Bioinformatics* **19**, 1572-1574 (2003).

Rougier, G., de la Fuente, M. S. & Arucci, A. B. Late Triassic turtles from South America. *Science* **268**, 855-858 (1995).

Rowe ,T. Definition, diagnosis, and origin of Mammalia. *J. Vertebr. Paleont.* **8**, 241-264 (1988).

RStudio Team (2020). RStudio: Integrated Development for R. RStudio, PBC, Boston, MA URL http://www.rstudio.com/.

Ruf, I., Maier, W., Rodrigues, P. & Schultz, C. Nasal anatomy of the non-mammaliaform cynodont *Brasilitherium riograndensis* (Eucynodontia, Therapsida) reveals new insight into mammalian evolution. *The Anat. Rec.* **297**, 2018-2030; 10.1002/ar.23022 (2014).

Ruta, M., Botha-Brink, J., Mitchell, S. A. & Benton, M. J. The radiation of cynodonts and the ground plan of mammalian morphological diversity. *Proc. R. Soc. B* **280**, 20131865; http://dx.doi.org/10.1098/rspb.2013.1865 (2013).

Santi Malnis, P., Colombi, C. E., Rothis, L. M. & Alcober, O. Fluvial architecture and paleoenvironmental evolution of the Los Colorados Formation (Norian): postrift stage of the Ischigualasto–Villa Unión Basin, NW Argentina. *J. Sediment. Res.* **90**, 1436-1462 (2020).

Schultz, C. L. *et al.* Triassic faunal successions of the Paraná Basin, southern Brazil. *J. S. Am. Earth Sci.* **104**, 102846; 10.1016/j.jsames.2020.102846 (2020).

Schulz, M. & Schillinger, B. Cold neutron radiography and tomography facility. *J. Large-Scale Res. Facil.* **1**,17; 10.17815/jlsrf-1-42 (2015).

Schwarz, D., Meyer, C. A., Lehmann, E. H., Vontobel, P. & Bongartz, G. Testing Neutron tomography in comparison with X-ray computed tomography as a technique for the investigation of the internal structure of sauropod vertebrae and ribs. *Palaeontol. Electron.* **8**, 1-11 (2005).

Scotese, C. R. *Atlas of Earth History* Vol. 1, Paleogeography, 52 pp. (PALEOMAP Project, 2001).

Seoane, F. D., Roig Juñent, S. & Cerdeño, E. Phylogeny and paleobiogeography of Hegetotheriidae (Mammalia, Notoungulata). *J. Vertebr. Paleont.* **37**, e1278547; 10.1080/02724634.2017.1278547 (2017).

Shubin, N. H., Crompton, A. W., Sues, H.-D. & Olsen, P. E. New fossil evidence on the sister-group of mammals and early Mesozoic faunal distributions. *Science* **251**: 1063-1065 (1991).

Sidor, C. A. & Hancox, P. J. *Elliotherium kersteni*, a new tritheledontid from the Lower Elliot Formation (Upper Triassic) of South Africa. *J. Paleontol*. **80**, 333-342 (2006).

Soares, M. B., Martinelli, A. G. & Oliveira, T. V. A new prozostrodontian cynodont (Therapsida) from the late triassic *Riograndia* assemblage zone (Santa Maria supersequence) of southern Brazil. *An. Acad. Bras. Ciências* **86**, 1673-1691 (2014).

Soares, M. B., Schultz, C. L. & Horn, B. L. D. New information on *Riograndia guaibensis* Bonaparte, Ferigolo & Ribeiro, 2001 (Eucynodontia, Tritheledontidae) from the Late Triassic of southern Brazil: anatomical and biostratigraphic implications. *An. Acad. Bras. Ciências* **83**, 329-354 (2011).

Sues, H.-D. & Jenkins, F. A. The postcranial skeleton of *Kayentatherium wellesi* from the Lower Jurassic Kayenta Formation of Arizona and the phylogenetic significance of postcranial features in tritylodontid cynodonts in *Amniote Paleobiology: Perspectivers on the Evolution of Mammals, Birds, and Reptiles* (eds. Carrano, M. T., Gaudin, T. J., Blob, R. W., Wible, J. R) 114–152 (The University of Chicago Press, 2006).

Sotomayor J. C., Leardi J. M., Otero A. & Gaetano L. C. Nuevos registros de Coelophysoidea (Dinosauria; Saurischia) de la Formación Los Colorados (Triásico Tardío). *Publicación Electrónica de la Asociación Paleontológica Argentina* **19**, R13 (2019).

Spiekman, S. N. F., Ezcurra, M. D., Butler, R. J., Fraser, N. C. & Maidment, S. C. R. *Pendraig milnerae*, a new small-sized coelophysoid theropod from the Late Triassic of Wales. *R. Soc. Open Sci.* **8**, 210915; 10.1098/rsos.210915 (2021).

Sterli, J., de la Fuente, M. S. & Rougier, G. W. Anatomy and relationships of *Palaeochersis talampayensis*, a Late Triassic turtle from Argentina. *Palaeontographica Abteilung A* **281**, 1-61 (2007).

Stipanicic, P. N. & Bonaparte, J. F. Cuenca Triásica de Ischigualasto-Villa Unión (Provincias de La Rioja y San Juan) in *Segundo Simposio de Geología Regional Argentina*. (ed. Turner, J. C. M.) 523-575 (Academia Nacional de Ciencias, 1979).

Stipanicic, P. N. Introducción in *Léxico Estratigráfico de la Argentina: Triásico* (eds. Stipanicic, P. N., Marsicano C.), 1–24 (Asociación Geológica Argentina, 2002).

Sues, H.-D. The relationships of the Tritylodontidae (Synapsida). *Zool. J. Linn. Soc* **85**, 205-217 (1985).

Sues, H.-D. The skull and dentition of two tritylodontid synapsids from the Lower Jurassic of western North America. *Bull. M. Comp. Zool.* **151**, 217-268 (1986).

Sues, H.-D. On *Microconodon*, a Late Triassic cynodont from the Newark Supergroup of eastern North America. *Bull. M. Comp. Zool.* **156**, 37-48 (2001).

Sun, A. & Cui, G. Tritylodont reptile from Xinjiang. *Vert. PalAs.* **27**, 1-8 (1989). [In Chinese with English summary].

Sun, A., & Li, Y. 1985. The postcranial skeleton of the late tritylodont *Bienotheroides*. *Vert. PalAs.* **23**, 133-151 (1985). [In Chinese with English summary].

Sun, A.L. 1986. New material of *Bienotheroides* (tritylodont reptile) from the Shaximiao Formation of Sichuan. *Vert. PalAs.* 24:165-170.

Świło, M., Niedźwiedzki, G. & Sulej, T. Mammal-like tooth from the Upper Triassic of Poland. *Acta Paleontol. Pol.* **59**, 815-820 (2013).

Taborda, J. R. A., Desojo, J. B. & Dvorkin, E. N. Biomechanical skull study of the aetosaur *Neoaetosauroides engaeus* using finite element analysis. *Ameghiniana* **58**, 401-415; 10.5710/AMGH.23.07.2021.3412 (2021).

Viglietti, P. A. *et al*. Biostratigraphy of the *Scalenodontoides* assemblage zone (Stormberg group, Karoo Supergroup), South Africa. *S. Afr. J. Geol.* **123**, 239-248 (2020).

Wallace, R. V. S., Martínez, R. & Rowe, T. First record of a basal mammaliamorph from the early Late Triassic Ischigualasto Formation of Argentina. *PLoS ONE* **14**, e0218791; 10.1371/journal.pone.0218791 (2019.)

Watabe, M., Tsubamoto, T. & Tsogtbaatar, K. A new tritylodontid synapsid from Mongolia. *Acta Palaeontol. Pol.* **52**, 263-274 (2007).

Watson, D. M. S. On a new cynodont from the Stormberg. *Geol. Mag.* **10**, 145-148 (1913).

Wible, J. R. Origin of Mammalia: the craniodental evidence reexamined. *J Vertebr Paleont*. **11**, 1-28 (1991).

Wible, J. R. & Hopson, J. A. Basicranial evidence for early mammal phylogeny in *Mammal Phylogeny: Mesozoic Differentiation, Multituberculates, Monotremes, Early Therians, and Marsupials* (eds. Szalay, F. S., Novacek, M. J., McKenna, M. C.) 45-62 (Springer- Verlag, 1993).

Young, C. C. New materials of Therapsida from Lufeng, Yunnan. *Vertebr. PalAs.* **12**, 111-114 (1974). [In Chinese] .

Yu, Y., Blair ,C. & He, X. J. RASP 4: Ancestral State Reconstruction Tool for Multiple Genes and Characters. *Mol. Biol. Evol.* **37**, 604-606 (2020).
