# Supplementary material for: A new cynodont from the Upper Triassic Los Colorados Formation (Argentina, South America) reveals a novel paleobiogeographic context for mammalian ancestors
Source: Sci Rep. 2022 Apr 25;12:6451. doi: 10.1038/s41598-022-10486-4 (PMC9038739; doi:10.1038/s41598-022-10486-4)
Supplement: Supplementary file 4 — Supplementary Information 4. [file 41598_2022_10486_MOESM4_ESM.pdf]

Notes on taxa

Charassognathus gracilis= SAM-PK-K10369. Botha et al. (2007); Kammerer (2016). Hoedemaker Member of the Teekloof Formation (Botha et al., 2007), *Endothiodon* Assemblage Zone, *Tropidostoma-Grogonops* Subzone. Mid Wuchiapingian (Day and Smith, 2020).

Procynosuchus delaharpeae= BP/1/36 (holotype of *Silphedocynodon gymnotemporalis*), BP/1/226 (holotype of *Aelurodraco microps*), BP/1/591 (holotype of *Leavachia gracilis*), BP/1/650 (holotype of *Protocynodon pricei*), BP/1/756, BP/1/1545, BP/1/1559, BP/1/2244, BP/1/2600, BP/1/2611, BP/1/3747, BP/1/3748, BP/1/5832; NHMUKPV37054 (ex OUMNH TSK34); RC 5 (holotype of *Procynosuchus delaharpeae*), RC 12 (holotype of *Procynosuchus rubidgei*), RC 17 (holotype of *Paracynosuchus rubidgei*) RC 27 (holotype of *Nanictosuchus melinodon*), RC 48 (holotype of *Nanictosaurus robustus*), RC 56 (holotype of *Mygalesaurus platyceps*), RC 72 (holotype of *Galeophrys kitchingi*), RC 87 (holotype of *Galecranium liorhynchus*), RC 92 (holotype of *Leavachia duvenhagei*), RC 130, RC 132, RC 133, RC 227, RC 304; SAM-PK-K338, SAM-PK-K5819, SAM-PK-K7600, SAM-PK-K8511, SAM-PK-K10394; NMQR 1450; AMNH FR 8220; UMCZ T.810 (holotype of *Parathrinaxodon proops*). Broom (1937b, 1938, 1948), Brink and Kitching (1951), Brink (1963b), Anderson (1968), Mendrez (1972), Kemp (1979, 1980), Abdala and Allinson (2005), Weide et al. (2009), Jasinoski et al. (in prep.); most of the Balfour Formation, Karoo Basin, South Africa, lower *Daptocephalus* AZ (Viglietti, 2020); recent finds have shown the presence of *Procynosuchus* at the top of the *Cistecephalus* AZ (Botha et al. 2007; but see Smith, 2020); Madumabisa Mudstones, Luangwa Valley, Zambia (Kemp 1979); Kawinga Formation (= Usili Formation of Wopfner 2002), Ruhuhu Valley, Tanzania (Parrington 1936; von Huene 1950); fissure fillings within the lower Zechstein, Germany (Sues and Boy 1988); ~255.2 Ma (Rubidge et al., 2013), Changhsingian (Viglietti, 2020).

Galesaurus planiceps= AMNH FARB 2223, AMNH FARB 2227; BP/1/2513A (478, 223) (holotype of *Notictosaurus trigonocephalus*), BP/1/3892, BP/1/4506, BP/1/4597, BP/1/4602, BP/1/4637, BP/1/4714, BP/1/5064; NHMUK R36220 (holotype of *Galesaurus planiceps*); NMP 581; NMQR 135, NMQR 655, NMQR 860, NMQR 1451, NMQR 3340, NMQR 3542, NMQR 3716; RC 845; SAM-PK-

K1119, SAM-PK-K8549, SAM-PK-K9956, SAM-PK-K10465, SAM-PK-K10468;TM 24 (holotype of *Glochinodon dentidens*), TM 83 (holotype of *Glochinodontoi desgracilis*); UMCZ T.819, UMCZ T.823. Watson (1920), Broom (1932b), Parrington (1934), Boonstra (1935), Rigney (1938), Brink (1954b), Abdala (2003), Jasinoski and Abdala (2017), Pusch et al. (2019), Butler et al. (2019), Norton et al. (2020); upper portion of the Balfour Formation, and Katberg and Normandien formations, Karoo Basin, South Africa, lower *Lystrosaurus declivis* AZ (Botha and Smith, 2020) and thus most likely Induan (Neveling 2004; Rubidge 2005). Latest contributions suggest that the upper Palingkloof Member is not older than 252.24 +/-0.1 (Gastaldo et al., 2020) and may be as young as 251.7+/-0.3 (Botha et al., 2020).

Thrinaxodon liorhinus= AMMM 4283; AMNH R9563; BP/1/472 (holotype of *Notictosaurus gracilis*), 1375, 1376, 1693, 1730, 1737, 2776, 2793, 2824, 3848, 4263, 4280, 4282, 5208, 5372, 5905, 7199; BSP 1934VIII 506; FMNH 156; MCZ 8892; NHMUK R511 (holotype of *Thrinaxodon liorhinus*), R511a, R845, R1715 (holotype of *Nythosaurus larvatus*), R3731, R5480; NMQR 24, 809, 810 (?holotype of *Thrinaxodon putterilli*; see van Heerden 1972), 811, 812, 1416, 1533, 1864, 3210; RC 107 (holotype of *Notictosaurus luckhoffi*); SAM-PK-K378, K379, K380, K381, K1121, K1388, K1461, K1467, K1468, K1483, K1498, K1499, K3592, K8004, K10016, K10017, K10549, K10607; TM 80A, 80B, 81A,166, 167, 180, K377, 782, 1486 (holotype of *Micrictodon marionae*) 4984, 5074; UCMP 40466, 42865, 42866, 42877, 42878; UMZC T.811, T.813?T.817; USNM 22812. Broom (1911), Watson (1920), Parrington (1936, 1946), Brink (1954b), Estes (1961), Crompton (1963), van Heerden (1972), Fourie (1974), Gow (1985), Fernandez et al. (2013), Abdala et al. (2013), Jasinoski et al. (2015); upper portion of the Balfour Formation, and Katberg and Normandien formations, Karoo Basin, South Africa, Lystrosaurus declivis AZ (Botha and Smith, 2020); Lower Fremouw Formation, Antarctica (Colbert and Kitching 1977); Panchet Formation, India.Induan?Early Olenekian (Botha and Smith, 2020).

Platycraniellus elegans= NMQR 1633; TM 25 (holotype of *Platycraniellus elegans*). Van Hoepen (1916, 1917), Haughton (1924), Broom (1932), Abdala (2007). Harrismith Member of the Normandien Formation in northern Free State Province

(Abdala, 2007). Lower *Lystrosaurus declivis* AZ, more likely Induan in age (Botha and Smith, 2020).

Cynognathus crateronotus= AM 460 (holotype of *Cynognathus platyceps*), 2190, 3587 (described as ?*Cynognathus leptorhinus* by Seeley 1895b), 4202, 5800; AMNH R5538, R5641;BP/1/1181, 2095, 3755, 4664; BSP 1934VIII1, 1934VIII2, 1934VIII3, 1934VIII6 (holotype of *Cynidiognathus merenskyi*); NHMUK R2571 (holotype of *Cynognathus crateronotus*), R2572, R3580; NMQ R1227, R1444; PVL 3859 (holotype of Cynognathus minor); SAM-PK-1056 (holotype of *Cynidiognathus broomi*), 6224 (holotype of *Cynidiognathus longiceps*), 6235, 11264, 11484. Seeley (1895b), Broili and Schröder (1934, 1935a), Brink (1955b), Bonaparte (1969), Jenkins (1971), Hammer (1995), Abdala (1996b, 1999), Wynd et al. (2018). Burgersdorp Formation, Karoo Basin, South Africa, *Langbergia-Garjainia*, *Trirachodon-Kannemeyeria* and *Cricodon-Ufudocyclops* subzones(former A to C subzones) of the *Cynognathus* AZ (Hancox et al., 2020); lower Lifua Member, Tanzania (Wynd et al., 2018); lower Ntawere Formation, Zambia (Wynd et al., 2018); upper Omingonde Formation, Namibia (Smith and Swart 2002; Abdala and Smith, 2009); Rio Seco de la Quebrada Formation, Argentina (Bonaparte 1969); upper Fremouw Formation, Antarctica (Hammer 1995); late Olenekian? late Anisian (Hancox et al., 2020) or late Ladinian-early Carnian (Ottone et al., 2014; Abdala et al., 2020).

Diademodon tetragonus= AM 438, 458 (holotype of *Gomphognathus kannemeyeri*), 3753 (holotype of *Octagomphus woodi*); AMNH R5518; BP/1/1195, 2522, 3511, 3639 (holotype of *Diademodon rhodesiensis*), 3754, 3756-3758, 3769, 3771-3773, 3776 (holotype of *Cragievarus kitchingi*), 4647, 4669, 4677; BSP 1934VIII 14, 1934VIII 15, 1934VIII 16, 1934VIII 17 (holotype of *Gomphognathus grossarthi*), 1934VIII 18 (holotype of *Gomphognathus broomi*), 1934VIII 19 (holotype of *Gomphognathus haughtoni*), 1934VIII 20, 1936II 8 (holotype of *Sysphinctostoma smithi*); MB R1004; NHMUK R2574, R2575, R2576?7 (holotype of *Gomphognathus polyphagus*), R2578, R3303 (holotype of *Diademodon mastacus*), R3304 (holotype of *Diademodon browni*), R3305 (holotype of *Microgomphodon oligocynus*), R3308, R3581 (holotype of *Microgomphodon eumerus*); R3587, R3588, R3724, R3765 (holotype of *Diademodon entomophonus*), R4092 (portion of the skull of the holotype of *Diademodon entomophoneus*), R9216; SAM-PK-

3426, 4002, 5877, K-177, 5222, 5223, 5266, 5877; UCMP 42446; UMCZ T.430, T.436 (holotype of *Diademodon laticeps*), T.438, T.441, T.445, T.454, T.828, T.971. Seeley (1894, 1895a), Watson (1911, 1913a), Broili and Schröder (1935b), Brink (1955a), Fourie (1963), Jenkins (1971), Hopson (1971), Crompton (1972b), Grine (1977), Martinelli et al. (2009), Gaetano et al. (2018), Hendrickx et al. (2019). Burgersdorp Formation, Karoo Basin, South Africa, *Trirachodon-Kannemeyeria* and *Cricodon-Ufudocyclops* subzones(former B to C subzones) of the *Cynognathus* AZ (Hancox et al., 2020); lower Ntawere Formation, Zambia (Peecoock et al., 2018); upper Omingonde Formation, Namibia (Smith and Swart 2002; Abdala and Smith, 2009); Rio Seco de la Quebrada Formation, Argentina (Martinelli et al., 2009); early-late Anisian (Hancox et al., 2020) or late Ladinian-early Carnian (Ottone et al., 2014; Abdala et al., 2020).

Trirachodon spp.= AM 434, 461 (holotype of *Trirachodon kannemeyeri*); BP/1/3511, 3775, 4258, 4658, 4661, 5050; BSP 1934VIII 21-23; CGP/1/33, 1/79 (=CGP JNN 2000-7-2A); GSN R327; NHMUK R2807, R3306, R3307, R3350, R3579 (holotype of *Trirachodon berryi*), R3721 (holotype of *Trirachodon browni*), R3722; NMQR 122, 1399, 3251, 3255, 3256, 3268, 3280; SAM-PK-987, 5873 (holotype of *Trirachodon minor*), 5880, 12168(=K5821), K142, K170, K171, K4801, K4803, K7888, K10157, K10161, K10176, K10207, K10411. Seeley (1895a), Broom (1911), Broili and Schröder (1935c), Crompton (1972b), Neveling (2002), Abdala et al. (2006), Sidor and Hopson (2018), Hendrickx et al. (2019). Burgersdorp Formation, Karoo Basin, South Africa, *Trirachodon-Kannemeyeria* subzone (former B subzone) of the *Cynognathus* AZ (Abdala et al. 2006); upper Omingonde Formation, Namibia (Keyser 1973; Abdala and Smith, 2009). NMQR 3279 has a maxillary platform lateral to the postcanines indicating the presence of *T. berryi* in *Langbergia-Garjainia* subzone (see Abdala et al. 2006); late Olenekian-early Anisian (Hancox et al., 2020) or late Ladinian early Carnian (Ottone et al., 2014; Abdala et al., 2020).

Langbergia modisei= BP/1/5362, 5363, 5400, 5401, 5404, 5666; CGP/1/33 120; NMQR 3251, 3255 (holotype of *Langbergia modisei*), 3256, 3268, 3280, 3281; SAM-PK-11481. Abdala et al. (2006), Sidor and Hopson (2018), Hendrickx et al. (2019). Burgersdorp Formation, Karoo Basin, South Africa *Langbergia-Garjainia* subzone;

late Olenekian (Hancox et al., 2020) or Ladinian (Ottone et al., 2014; Abdala et al., 2020).

Sinognathus gracilis= IVPP V2339 (holotype of *Sinognathus gracilis*). Young (1959), Sun (1988), Hendrickx et al. (2019). Ermaying Formation, China, late Anisian (Liu et al., 2018).

Beishanodon youngi= PKUP V3007 (holotype of *Beishanodon youngi*); skull minus mandible. Gao et al. (2010). Dark shales of the Hongyanjing Formation, China; Olenekian (Gao et al., 2010). Scored after publication.

Scalenodon spp.= UMZC T.907, T.908, T.909, T.910, T.911, T.912, T.913, T.914, T.915, T.916, T.917, T.918; UFRGS-PV-0239-T. Parrington (1946), Crompton (1955, 1972a), Abdala and Teixeira (2004), Melo et al. (2017). Manda Formation, Tanzania, Santa Maria Formation, Brazil (Melo et al., 2017, but see Hendrcikx et al., 2020); Late Anisian (Hancox et al., 2020) or Carnian (Ottone et al., 2014) see also Peecook et al. (2018) and Abdala et al (2020). Generic identity of Scalenodon ribeiroae described by Melo et al. (2017) was recently questioned by Hendrickx et al. (2020).

Luangwa spp.= BP/1/3731 (holotype of *Luangwa drysdalli*), 3733; GSN OM-2, RK-4a, RK-4b, RK-4c, R 572, NHMUK PV R 36995 (ex OUMNH TSK 121), MCP 3167PV (holotype of *Luangwa sudamericana*), UFRGS 267PV. Brink (1963), Kemp (1980), Abdala and Teixeira (2004), Abdala and Smith (2009). Upper Ntawere Formation, Zambia; Santa Maria Formation, Brazil; late Anisian (Hancox et al., 2020) or late Ladinian, early Carnian)(Abdala and Smith, 2009; Peecook et al., 2018, see also Abdala et al., 2020).

Nanogomphodon wildi= SMNS 51962 (holotype of *Nanogomphodon wildi*). Hopson and Sues (2006). Sandige Pflanzenschiefer, Lower Lettenkeuper, Lower Keuper or Erfurt Formation; late Ladinian (Hopson and Sues, 2006). Scored after publication. Holotype and only specimen is an isolated left lower postcanine.

Mandagomphodon hirschsoni = NHMUK R8577 (holotype of *Mandagomphodon hirschsoni*). Crompton (1972), Hopson (2014). Lifua Member, Manda Beds; late Anisian (Hancox et al., 2020) or late Ladinian, early Carnian (Abdala and Smith, 2009; Peecook et al., 2018, see also Abdala et al., 2020).

Mandagomphodon attridgei = NHMUK R8578 (holotype of *Scalenodon attridgei*), CAMZM 922 (Ruhuhu Field catalogue no. 136) (holotype of *Scalenodon charigi*). Crompton (1972). Several scores are based in the unpublished illustrations by Hopson (lateral and palatal view of the holotype and details of both postcanine series). These species of Scalenodon were considered as member of Mandagomphodon and S. charigi synonymized as M. attridgei by Liu and Abdala (2014). Lifua Member, Manda Beds; late Anisian (Hancox et al., 2020) or late Ladinian, early Carnian (Abdala and Smith, 2009; Peecook et al., 2018, see also Abdala et al., 2020).

Gomphodontosuchus brasiliensis= GPIT/RE/09397 (holotype of *Gomphodontosuchus brasiliensis*). Snout and orbital region of the skull and mandible extremely deformed. Huene (1928, 1936), Hopson (1985). Santa Maria Formation, Brazil, *Hyperodapedon* Asemblage Zone, late Carnian (Abdala et al., 2020; Schultz et al., 2020).

Scalenodontoides macrodontes= BP/1/5395; MNHN 1955-25, 1957-23 (holotype of *Scalenodontoides macrodontes*), NMQR 3053, SAM-PK-K336.Crompton and Ellenberger (1957), Gow and Hancox (1993), Battail (2005). *Scalenodontoides* Assemblage Zone, lower Elliot Formation, South Africa, Middle Norian to Rhaetian (Viglietti et al., 2020a).

Pascualgnathus polanski= MLP 65-VI-18-1 (holotype), MLP 65-VI-18-2, PVL 3466, PVL 4416. Bonaparte (1966), Martinelli (2010). Río Seco de la Quebrada Formation, late Olenekian?late Anisian (Hancox et al., 2020) or late Ladinian-early Carnian (Ottone et al., 2014; Abdala et al., 2020).

Andescynodon mensozensis=PVL 3833 (holotype), PVL 3834, PVL 3840 (holotype of *Rusconiodon mignonei*), PVL 3890, PVL 3892 (a, b, c, d), PVL 3894, PVL 3894-1, PVL 3899, PVL 3900, PVL 4390, PVL 4423, scapula; PVL 4424?4427. Bonaparte (1969a, 1970),Goñi (1986) and Goñi and Goin (1987, 1988),Goñi and Abdala (1989), Liu and Powell (2009).Cerro de las Cabras Formacion. Anisian (Abdala et al., 2020).

Santacruzodon hopsoni= MCN PV 2768 (holotype), MCN PV 2751, MCN PV 2752, MCP 4044 PV, MCN PV 2770, MCP 4034 PV, MMACR-PV-002-T, MMACR-PV-004-T,MMACR-PV-005-T,MMACR-PV-006-T, MMACR-PV-015 T,

UFRGS-PV-1268-T, UFRGS-PV-0457-T, UFRGS-PV-0585-T, UFRGS-PV-0586-T, UFRGS-PV-0431-T, UFRGS-PV-0544-T,UFRGS-PV- 0576-T. Abdala and Ribeiro (2003), Melo et al. (2019). Santa Maria Formation, *Santacruzodon* Assemblage Zone, Early Carnian (Schultz et al., 2020).

Protuberum cabralense= MGB 368 ? 100 (holotype), UFRGS PV 0981T; UFRGS PV 0983T; UFRGS PV 0985T; UFRGS PV 0986T; UFRGS PV 1009T; UFRGS PV 1010T; UFRGS PV 1011T. Reichel et al. (2009). Santa Maria Formation, *Dinodontosaurus* Assemblage Zone, Early Carnian (Schultz et al., 2020).

Aleodon brachyramphus =UMZC T906 (holotype), NHMUK R9390, NHMUK R10048. Crompton (1955), Abdala and Giannini (2002). Lifua Member, ?Anisian, ?Late Ladinian-Early Carnian (Abdala, 2021).

Aleodon cromptoni = MPDC-501-117 (holotype), MCN-PV 10338, UFRGS-PV-0071-T, MMACR-PV-018-T, UFRGS-PV-0122-T, UFRGS-PV-0125-T, UFRGS-PV-0146-T, UFRGS-PV-0274-T. Specimens tentatively referred to this species include: GSN EN-3, MCP-PV-1695T.Martinelli et al. (2017a), Oliveira et al. (2009), Abdala and Smith (2009). Santa Maria Formation, *Dinodontosaurus* Assemblage Zone, Early Carnian (Schultz et al., 2020), ?upper Omingonde Formation.

Probainognathus jenseni= PVL 4447, PVL 4169, PVL 4673, PVL 4677, PVL 4725,PVL 4724, PVL no number, MCZ 4017, MCZ 4018, MCZ 4019, MCZ 4021. Romer (1970), Romer and Lewis (1973), Abdala (1996a), Lucas and Luo (1993), Luo (1994), Crompton et al. (2017). Early Carnian (Abdala, 2021).

Therioherpeton cargnini= MVP 05.22.04 (holotype). Bonaparte and Barberena (1975, 2001), Oliveira (2006), Martinelli et al. (2017c). Santa Maria Formation, *Hyperodapedon* Assemblage Zone, Late Carnian (Schultz et al., 2020).

Boreogomphodon jeffersoni= USNM 437632 (holotype), CM 20050, CM 76800, CM 76801, CM 76803, USNM 437634, USNM 437635, USNM 437636, USNM 448562, USNM 448570, USNM 448593, USNM 448599, USNM 448632, USNM 448633, VMNH 3575, VMNH 3578, CM 76815, CM 76818, USNM 448567, USNM 448602, USNM 448598, VMNH 3577, NCSM 20698, NCSM 20711, NCSM 21370, NCSM 11466, NCSM 15295 NCSM 16297 , NCSM 16292, NCSM 18300. Sues and Olsen (1990); Sues and Hopson (2010); Liu and Sues (2010); Liu

et al. (2017). Tomahawk Creek Member of the Vinita Formation, Pekin Formation. Carnian (Abdala, 2020).

Arctotraversodon plemmyridon= Hopson, 1984; Sues et al 1992.

Lumkuia fuzzi= BP/1/2669. Hopson and Kitching (2001). Burgersdorp Formation, *Trirachodon-Kannemeyeria* Subzone, *Cynognathus* Assemblage Zone. Anisian (Hancox et al., 2020).

Traversodon stahleckeri= von Huene 1936; Barberena, 1981. Santa Maria Formation, *Dinodontosaurus* Assemblage Zone. Early Carnian (Schultz et al., 2020).

Brasilodon tetragonus= UFRGS PV 611T(holotype), UFRGS PV 594T (holotype of *Brasilitherium riograndensis*), UFRGS PV 628T, UFRGS PV 765T, UFRGS PV 1042, UFRGS PV 760T, UFRGS PV 785T, UFRGS PV 804T, UFRGS PV 835T, UFRGS PV 922T, UFRGS PV 929T. Bonaparte et al. (2003, 2005); Bonaparte (2012), Ruf et al. (2014), Rodrigues et al. (2013). Guignard et al. (2019). Caturrita Formation, Riograndia Assemblage Zone, Norian (Schultz et al., 2020).

Chaliminia musteloides= PVL 3857, PULR 081. Bonaparte (1980); Martinelli and Rougier (2007). Los Colorados Formation. Norian (Abdala et al., 2020).

Riograndia guaibensis= MCN PV2274 (holotype), 2265, 2267, 2268, 2271.Bonaparte et al. (2001), Soares et al. (2011). Caturrita Formation, Riograndia Assemblage Zone, Norian (Schultz et al., 2020).

Massetognathus spp.= NHMUK R8430; MCZ 3691, MCZ 3786, MCZ 3789, MCZ 3801, MCZ 3804, MCZ 3806, MCZ 3807, MCZ 4021, MCZ 4138, MCZ 4208, MCZ 4215, MCZ 4216, MCZ 4258, MCZ 4265, MCZ 4627; PULR 10 (holotype of *Massetognathus pascuali*), PULR 11 (holotype of *Massetognathus major*), PULR 13 (holotype of *Massetognathus teruggii*), PULR without? number (holotype of *Megagomphodon oligodens*); PVL 3901?3904, PVL 3906, PVL 4613, PVL 4726, PVL 4727?4729. Romer (1967, 1972), Crompton (1972a, b); Abdala and Giannini (2000), Liu et al. (2008), Lai et al. (2018), Schmitt et al. (2019). Chanares Formation; Santa Maria Formation, *Dinodontosaurus* Assemblage Zone. Early Carnian (Schultz et al., 2020).

Dadadon isaloi= UA 10606, FMNH PR 2232, FMNH PR 3034?3038, UA 10605, UA 10608?10617. Flynn et al. (2000); Ranivoharimanana et al. (2011);

Ranivoharimanana (2012), Kammerer et al. (2012).Makay Formation. Early Carnian (Abdala et al., 2020).

Menadon besairiei= UA 10601, FMNH PR 2444, FMNH PR 2104, UFRGS-PV-0269-T, UFRGS-PV-0434-T, UFRGS-PV-0891-T, UFRGS-PV-0903-T, UFRGS-PV-0905-T, UFRGS-PV-1054-T, UFRGS-PV-1164-T, UFRGS-PV-1165-T, MCN-PV 0505, MCN-PV 2750. Flynn et al. (2000), Kammerer et al. (2008), Ranivoharimanana (2012), Melo et al. (2015), Melo et al. (2019). Makay Formation; Santa Maria Formation, *Santacruzodon* Assemblage Zone. Early Carnian (Abdala et al., 2020).

Exaeretodon spp.= MACN 18114, MACN 18125; MCZ 111-64A, MCZ 33458M, MCZ 377-58M, MCZ 4074, MCZ 4468?4470, MCZ 4480, MCZ 4482, MCZ 4483, MCZ 4486, MCZ 4493, MCZ 4500, MCZ 4502, MCZ 4510, MCZ 4781; MLP 43-VII-14-2, MLP 43-VII-14-3; MCP 1522 PV (holotype of Exaeretodon riograndensis), MCP 2361 PV, MCP 3843 PV; PVL 2056, 2079, 2082, 2083, 2094, 2473, 2554, 2565, 2750; PVSJ 157.Bonaparte (1962, 1963a,b,c, 1966), Chatterjee (1982), Hopson (1984), Goñi and Goin (1990), Abdala et al. (2002), Abdala and Malabarba (2007), Liu (2007), Oliveira, et al. (2007), Liparini et al. (2013), Kubo et al. (2017). Ischigualasto Formation, *Scaphonyx-Exaeretodon-Herrerasaurus* Biozone, Argentina; Santa Maria Formation, Paraná Basin, Brazil, *Dinodontosaurus* (*Exaeretodon major*) and *Hyperodapedon* Assemblage Zone (*Exaeretodon riograndensis*); Maleri Formation, India; the presence of *Exaeretodon* in the *Dinodontosaurus* Assemblage Zone of Brazil should be considered with caution, because the taxonomic identity of *E. major* is tentative (see Abdala et al. 2002). Late Carnian (Abdala et al., 2020).

Ecteninion lunensis= PVSJ 422 (holotype of *Ecteninion lunensis*), PVSJ 481, PVSJ 693. Martinez et al. (1996). Ischigualasto Formation, Ischigualasto-Villa Union Basin, *Scaphonyx-Exaeretodon-Herrerasaurus* Biozone, Argentina. Late Cranian (Abdala et al. 2020).

Trucidocynodon riograndensis= UFRGS PV-1051-T (holotype), UFRGS PV-1051-T, UFRGS PV-1053-T, UFRGS PV-1069-T, UFRGS PV-1070-T, UFRGS PV-1071-T, CAPPA/UFSM 0029. Oliveira et al. (2010, 2016), Stefanello et al. (2018). Santa Maria Formation, *Hyperodapedon* Assemblage Zone, Late Carnian (Schultz et al., 2020).

Chiniquodon spp.= GPIT 40 (holotype of *Chiniquodon theotonicus*), 1050 (holotype of *Belesodon magnificus*); NHMUK R8429, MCP PV1600 (holotype of *Probelesodon kitchingi*), PULR 12* (holotype of *Probelesodon minor*), PULR 18* (holotype of *Probelesodon lewisi*), UFRGS PV 66T, UFRGS PV66Tg, UFRGS PV122T,UFRGS PV274, UFRGS PV275T; PVL 4167,PVL 4444, PVL 4448, PVL 4674, PVL 4675; MCZ 1533, MCZ 3035, MCZ 3614, MCZ 3615, MCZ 3776, MCZ 3777, MCZ 3779, MCZ 3781, MCZ 4002, MCZ 4020, MCZ 4100, MCZ 4296, MCZ 8823, PVSJ 411 (holotype of *Probelesodon sanjuanensis*),CRILAR-PV109, Huene (1935-1942); Bonaparte (1966); Romer (1969a, 1969b, 1973), Romer and Lewis (1973), Teixeira (1982), Martinez and Foster (1996), Abdala (1996a), Abdala and Gianini (2002), Kemp (2009), Abala and Smith (2009), Kammerer et al. (2010), Mocke et al. (2019). Chanares and Ischigualasto formations, upper Omingonde Formation, Makay Formation, Santa Maria Formation, *Dinodontosaurus* Assemblage Zone. Early Carnian (Abdala et al., 2020, Schultz et al., 2020).

Adelobasileus cromptoni= NMMNH P-12971 (holotype). Lucas and Hunt (1990), Lucas and Luo (1993). Tecovas Formation, Norian (Abdala, 2021).

Oligokyphus spp.= NHMUK: several specimens, MCZ 8868- 8886, MCZ 8843, MCZ 8851-8867, MCZ 8846, MNA V3244, MCZ 8843, MCZ 8897, IVPP 4008. Kühne (1956), Crompton (1964), Luo and Sun (1984), Sues (1985), Fedak et al. (2015). Windsor Hill Quarry (?Mendip 14?), England (Kühne 1956); Rhaeto-Liassic bone bed, Baden-Württemberg, Germany (Simpson 1928); Kayenta Formation, northern Arizona, USA (Sues 1985b); Lower Lufeng Formation, Yunnan, China (Luo and Sun 1993); Rheatian-Pliensbachian (Abdala, 2021).

Kayentatherium wellesi= MCZ 8811, MCZ 8812. MCZ 8832, MCZ 8835, MCZ 8838, MNA V3141, MNA V3224, MNA V3235, USNM 317201, TMM 43690-5. Kermack (1982), Clark and Hopson (1985), Lewis (1986), Sues (1986), Sues et al. (1994), Sues and Jenkins (2007), Hoffman and Rowe (2018). Kayenta Formation, northern Arizona, USA; Sinemurian?Pliensbachian (Kielan-Jaworowska et al. 2004).

Tritylodon longaevus= NHMUK R8181 (holotype), NHMUK R8502, NHMUK R15976, NHMUK R15979, BSP 1936 II1-II6, BP/1/4965, BP/1/4976, BP/1/4261, BP/1/4778, BP/1/4782, BP/1/4785, BP/1/5167, SAM-PK-K1411, SAM-PK-K1330, SAM-PK-K1474, SAM-PK-K4799, SAM-PK-K7903, Owen (1894),

Broom (1905,1910), Broili and Schroder (1936), Ginsburg (1962), Gow (1986, 1991), Gaetano et al (2017).upper Elliot Formations, Massospondylus Assemblage Zone, Hettangian-Pliensbachian (Viglietti et al., 2020b).

Bienotherium spp.=IVPP V1-V5, IVPP V7-V12, IVPP V14, IVPP V65-V67, IVPP V68, IVPP V4009, IVPP V8536, IVPP V8537; GMC V1037 (holotype of *Bienotherium magnum*). Young (1940, 1947), Watson (1942); Chow (1962), Hopson (1964, 1966), Cui and Sun (1987), Luo and Wu (1994). Lower Lufeng Formation (Luo and Wu, 1994). (Hettangian-Sinnemurian (Abdala, 2021).

Elliotherium kersteni=BP/1/6106 (holotype). Sidor and Hancox (2006). ? lower Upper Elliot Formation (Bordy et al., 2020, Viglietti et al., 2020). ?Hettangian.

Pachygenelus monus= NHMUK R4091 (holotype); BP/1/4381,BP/1/ 4741, BP/1/4761, BP/1/5110, BP/1/5623, BP/1/5691; SAM-PK-K1329, SAM-PK-K1350, SAM-PK-K-1394. Watson (1913b); Gow (1980, 2001); Shubin et al. (1991), Wible and Hopson (1993). Upper Elliot Formation, Karoo Basin, South Africa; McCoy Brook Formation, Nova Scotia, Canada; Early Jurassic (Liassic) (Shubin et al. 1991; Lucas and Hancox 2001; Kielan-Jaworowska et al. 2004, Viglietti et al., 2020b).

Diarthrognathus broomi= NMQR 1536 (holotype), BP/1/4882. Crompton (1958,1963), Gow (1980,1994). upper Elliot and Clarens formations, *Massospondylus* Assemblage Zone, Hettangian-Pliensbachian (Viglietti et al., 2020b).

Sinoconodon rigneyi= FMNH CUP 1 (holotype), FMNH CUP 5, IVPP V4726, IVPP V4727, IVPP V4729, IVPP V6693, IVPP V6747, IVPP V7201, IVPP V7203, IVPP V8683, IVPP V8688, IVPP V8689, IVPP V8691, IVPP V8692. Patterson and Olson (1961), Crompton and Sun (1985), Lucas and Luo (1993), Crompton and Luo (1993), Luo (1994). Lufeng Formation, Sinnemurian (Abdala, 2021).

Morganucodon spp.= CUP 2320, IVPP 8682, IVPP 8684, several specimens at the NHMUK and UMCZ. Morganucodon material includes 100 teeth from Duchy Quarry in the United Kingdom; massive quantities of partial or complete bones and teeth from Ewenny, Pant and Pontalum quarries in Wales (for example 900 mandibular fragments); five skulls and several partial skulls in China; 30 isolated teeth in continental deposits from Europe (Abdala, 2021). Kermack et al. (1973); Kermack and Mussett (1981); Jenkins and Parrington (1976), Lucas and Luo

(1993), Wible and Hopson (1993), Luo (1994). Rhaetian-Pliensbachian, Bathonian (Abdala, 2021).

Dvinia prima= PIN 2005/2469 (holotype of *Permocynodon sushkini*), PIN 2005/2465. Sushkin (1929), Konjukova (1946), Tatarinov (1968), Ivakhnenko (2013). Sokolki Subassemblage (lower Vyatskian Gorizont) (Golubev 2000; Modesto and Rybczynski 2000); late Wuchiapingian (Cisneros et al. 2005), Changhsingian (Rubidge 2005).

Cynosaurus suppostus= AM 4947; NHMUK R1718 (holotype of *Cynosuchus suppostus*); SAM-PK-4333 (holotype of *Cynosuchus whaitsi*); AM 4947, BP/1/1563, BP/1/3926, BP/1/4469, SAM-PK-K5211, SAM-PK-K5819, SAM-PK-K10694. Owen (1876), Haughton (1918), Brink (1965b), van Heerden (1976), Van den Brandt and Abdala (2018); most of the Balfour Formation, Karoo Basin, South Africa, *Dicynodon* AZ (Kitching 1995); Brink (1965b) reported that the provenance of BP/1/3926 was from levels of the Lystrosaurus AZ (see also Sidor and Smith 2004), but Kitching (1977, p. 86) stated that this specimen unquestionably came from levels of the *Daptocephalus* Zone (= *Dicynodon* AZ); early to end of the Lopingian (Lucas 2002); late Wuchiapingian-Changhsingian (Cisneros et al. 2005); Changhsingian (Rubidge 2005).

Progalesaurus lootbergensis= SAM-PK-K-9954 (holotype of *Progalesaurus lootbergensis*). Sidor and Smith (2004); near the top of Palingkloof Member of the Balfour Formation, Karoo Basin, South Africa, lowermost Lystrosaurus AZ (Sidor and Smith 2004); Induan (Neveling 2004; Rubidge 2005).

Nanictosaurus kitchingi= TM 279, RC 133. Van Heerden (1976), Van Heerden and Rubidge (1990). Balfour Formation, Daptocephalus Assemblage Zone (Viglietti, 2020).

Cricodon metabolus= UMCZ T905, BP/1/5540, BP/1/5835, BP/1/6159, BP/1/6102, NHCC LB28, NMT RB227, NHMUK PV R36800; NHMUK PV R3722, SAM-PK-6212, 5881a,b. Crompton (1955), Abdala et al. (2005), Hendrickx et al. (2016, 2019), Sidor and Hopson (2018). middle to upper Lifua Member, Manda Beds; subzones *Trirachodon-Kannemeyeria* and *Cricodon-Ufudocyclops* (former B and C) of the *Cynognathus* AZ, Burgersdorp Formation, Beaufort Group, Karoo

Supergroup; upper Ntawere Formation. Early to late Anisian, Middle Triassic (Hendrickx et al., 2019).

Tessellatia bonapartei= PULR-V121 (holotype). Los Colorados Formation. Norian.

Bonacynodon =MCT-1716-R (holotype), MCT-1717-R. Martinelli et al. (2016). Santa Maria Formation, *Dinodontosaurus* Assemblage Zone. Early Carnian (Schultz et al., 2020).

Protheriodon estudianti= UFRGS PV0962T (holotype). Bonaparte et al. (2006), Martinelli et al. (2016). Santa Maria Formation, *Dinodontosaurus* Assemblage Zone. Early Carnian (Schultz et al., 2020).

Prozostrodon brasiliensis= UFRGS-PV-0248 (holotype), CAPPA/UFSM 0123. Barberena et al. (1987), Bonaparte and Barberena (2001), Pacheco et al. (2017). Santa Maria Formation, *Hyperodapedon* Assemblage Zone, Late Carnian (Schultz et al., 2020).

Alemoatherium huebneri= UFSM 11579b (holotype). Martinelli et al. (2017b). Santa Maria Formation, *Hyperodapedon* Assemblage Zone, Late Carnian (Schultz et al., 2020).

Botucaraitherium belarminoi= MMACR-PV-003-T (holotype). Soares et al. (2014). Caturrita Formation, *Riograndia* Assemblage Zone. Norian (Schultz et al. 2020).

Santacruzgnathus abdalai= UFRGS-PV-1121-T (holotype). Soares et al. (2011), Martinelli et al. (2016). Santa Maria Formation, *Santacruzodon* Assemblage Zone, Early Carnian (Schultz et al. (2020).

Candelariodon barberenai= MMACR PV-0001-T (holotype). Oliveira et al. (2011), Martinelli et al. (2017c). Santa Maria Formation, Dinodontosaurus Assemblage Zone. Early Carnian (Schultz et al., 2020).

Charruodon tetracuspidatus= MCP-3934 PV (holotype). Abdala and Ribeiro (2000), Martinelli et al. (2017b). Santa Maria Formation, *Hyperodapedon* Assemblage Zone. Late Carnian (Schultz et al., 2020).

Microconodon tenuirostris= ANSP 10248 (holotype), USNM 437637, ROM 44300, ROM 44301, USNM 448579, USNM 448600, ROM 44302. Simpson (1926); Sues et al. (1994), Sues(2001). Cumnock Formation, Sanford subbasin, Deep River Basin, Newark Supergroup (Sues, 2001).

Pseudotherium argentinus=PVSJ 882. Wallace et al. (2019). Ischigualasto Formation, upper portion of the La Peña Member and lower portion of the *Scaphonyx-Exaeretodon-Herrerasaurus* biozone. Late Carnian.

Diegocanis elegans= PVSJ 881. Martinez et al. (2013). Ischigualasto Formation, Middle *Scaphonyx-Exaeretodon-Herrerasaurus* Biozone (Martinez et al., 2013). Late Carnian.

Irajatherium hernandezi= UFRGS PV-0599-T (holotype), UFRGS PV-1029-T, UFRGS PV-1068-T, UFRGS PV-1167-T, UFRGS PV-1169-T, UFRGS PV-1170-T, UFRGS PV-1171-T, UFRGS PV-1172-T, UFRGS PV-1175-T.Martinelli et al. (2005), Oliveira et al. (2010). Caturrita Formation, *Riograndia* Assemblage Zone, Norian (Schultz et al., 2020).

Tritheledon riconoi= SAM 1885, SAM 2782. Broom (1912), Gow (1980). Upper Elliot Formation, *Massospondylus* Assemblage Zone, Hettangian-Sinemurian (Viglietti et al., 2020b)

Vetusodon elikhulu= BP/1/7971, CGP GHG141, SAMPK-K10702, SAM-PK-K10596. Abdala et al. (2019). Balfour Formation; *Lystrosaurus maccaigi-Moschorhinus* Subzone, Daptocephalus Assemblage Zone. Upper Changhsingian (Viglietti, 2020).

Abdalodon diastematicus= SAM-PK-K10138. Botha-Brink and Abdala (2008); Kammerer (2016). Hoedemaker Member of the Teekloof Formation; *Tropidostoma-Gorgonops* Sub Assemblage Zone (*Endothiodon* Assemblage Zone) (Day and Smith, 2020).

Abdalodon muchingaensis = NHCC LB277. Huttenlocker and Sidor (2020) and Pusch et al., 2021. Upper Madumabisa Mudstone Formation, Lopingian (late Permian, Wuchiapingian).

Etjoia dentitransitus= GSN F1591 (holotype). Hendrickx et al. (2020). upper Omingonde Formation, late Anisian (Hancox et al., 2020) or late Ladinian, early Carnian (Abdala and Smith, 2009; Peecook et al., 2018, see also Abdala et al., 2020).
