# Supplementary material for: A new cynodont from the Upper Triassic Los Colorados Formation (Argentina, South America) reveals a novel paleobiogeographic context for mammalian ancestors
Source: Sci Rep. 2022 Apr 25;12:6451. doi: 10.1038/s41598-022-10486-4 (PMC9038739; doi:10.1038/s41598-022-10486-4)
Supplement: Supplementary file 5 — Supplementary Information 5. [file 41598_2022_10486_MOESM5_ESM.pdf]

List of characters with notes on character definition and scorings

1. Internarial bar (Structure formed by the dorsal projection of the premaxillary anterior process, connecting dorsally with the anterior tips of the nasals and separating the external nasal opening. Also called internasal, ascending or prenasal process.)

0 present

1 absent

Charassognathus gracilis Character = 1 Changed from 0 to ? as this region is unknown.

Procynosuchus delaharpeae Character = 1 RC 92.

Galesaurus planiceps Character = 1 BP/1/4602, NHMQR 3340.

Platycraniellus elegans Character = 1 Abdala (2007, text-fig. 2E).

Cynognathus crateronotus Character = 1 BSP 1934VIII1, 1934VIII6.

Trirachodon spp. Character = 1 BP/1/4658.

Langbergia modisei Character = 1 NMQR 3255.

Sinognathus gracilis Character = 1 Anterior portion of the snout not preserved in IVPP V2339.

Beishanodon youngi Character = 1 Gao et al (2010:8): The premaxilla sends a slender internasal process first dorsally then turning posteriorly to separate the external nares.

Scalenodon spp. Character = 1 SAM-PK-11755.

Luangwa spp. Character = 1 MCP 3167PV.

Mandagomphodon hirschsoni Character = 1 The anterior portion of the snout dorsally is compressed and shattered and it is not possible to recognize this feature with certainty changed from 0 to ?.

Mandagomphodon attridgei Character = 1 Broken but preserved the tip between the nasals.

Scalenodontoides macrodontes Character = 1 Changed from 1 to 0. In MNHN 1955-25 seems to be present (Battail, 2005, fig. 8).

Pascualgnathus polanski Character = 1 Changed from 0 to 1.

Santacruzodon hopsoni Character = 1 Changed from 0 to ?.

Aleodon brachyramphus Character = 1 After NHMUK R9387, R9390.

Therioherpeton cargnini Character = 1 Changed from 0 to ?.

Boreogomphodon jeffersoni Character = 1 Scored after NCSM 20698.

Lumkuia fuzzi Character = 1 Changed from 0 to ?.

Chaliminia musteloides Character = 1 Changed from 0 to 1.

Exaeretodon spp. Character = 1 Incomplete.

Trucidocynodon riograndensis Character = 1 Changedfrom 0 to 1.

Oligokyphus spp. Character = 1 Changed from 0 to 1.

Tritylodon longaevus Character = 1 Changed from 0 to 1 as the process is incomplete according to Broom 1910.

Bienotherium spp. Character = 1 Changed from 0 to 1 according to Young, 1947.

Elliotherium kersteni Character = 1 Changed from 0 to ?.

Diarthrognathus broomi Character = 1 Changed from 0 to ?.

Sinoconodon rigneyi Character = 1 Changed from 0 to 1. It is apparently absent according to Kermack and Mussett 1981.

Morganucodon spp. Character = 1 Changed from 0 to 1. It is apparently absent according to Kermack and Mussett 1981.

Dvinia prima Character = 1 Changed from 0 to ?.

Cricodon metabolus Character = 1 Change from 1 to ?.

Prozostrodon brasiliensis Character = 1 Changed from ? to 0.

2. Extranasal process of the premaxilla (Dorsal projection on the lateroposterior margin of the premaxilla at the contact with the maxilla.)

0 small

1 large but not contacting nasal

2 contacting nasal

Charassognathus gracilis Character = 2 Changed from ? to 0.

Procynosuchus delaharpeae Character = 2 Observed in RC 304, see also Kemp (1979, fig. 3a).

Galesaurus planiceps Character = 2 BP/1/4602, NMQR 3542.

Thrinaxodon liorhinus Character = 2 UCMP 40466.

Platycraniellus elegans Character = 2 Typical NM cynodont style, with no projection dorsally.

Cynognathus crateronotus Character = 2 Changed from 0 to 1, after NMQR 1444.

Diademodon tetragonus Character = 2 UCMP 42446.

Trirachodon spp. Character = 2 BP/1/4658.

Beishanodon youngi Character = 2 Gao et al., 2010: figs. 3C, 4C).

Luangwa spp. Character = 2 Tentative score after MCP 3167PV.

Mandagomphodon attridgei Character = 2 Reconstruction by Jim Hopson.

Andescynodon mendozensis Character = 2 Changed from ? to 0.

Boreogomphodon jeffersoni Character = 2 Scored after NCSM 20698.

Traversodon stahleckeri Character = 2 Changed from 2 to 0.

Brasilodon tetragonus Character = 2 Changed from ? to 0.

Chaliminia musteloides Character = 2 Changed from 2 to ? (sutures not visible).

Dadadon isaloi Character = 2 Changed from ? to 0.

Menadon besairiei Character = 2 Changed from ? to 0.

Oligokyphus spp. Character = 2 Changed from 1&2 to 2. .

Bienotherium spp. Character = 2 Changed from 1 to 2 according to Young, 1947.

Dvinia prima Character = 2 It is not possible to be sure of the extension of this process from the illustrations (e.g. Sushkin, 1929: fig. 2).

Nanictosaurus kitchingi Character = 2 Changed from ? to 0 (TM279).

Cricodon metabolus Character = 2 As observed in C. kannemeyeri SAM-PK-12168.

Bonacynodon schultzi Character = 2 Changed from ? to 0.

Protheriodon estudianti Character = 2 It is interpreted by Bonaparte et al 2016 to have contacted the nasal.

Prozostrodon brasiliensis Character = 2 Changed from 2 to 1 after fig 1 of Barberena et al 1987 and Bonaparte and Barberena 2001.

Pseudotherium argentinus Character = 2 Inferred.

3. Septomaxilla facial process (Dorsoposterior projection of the septomaxilla located between nasal and maxilla.)

0 long

1 short

Charassognathus gracilis Character = 3 Changed from ? to 1.

Procynosuchus delaharpeae Character = 3 Observed in RC 5, 304.Brink 1963 (fig. 10c), Kemp (1979, fig. 3a).

Galesaurus planiceps Character = 3 BP/1/4602, NMQR 3542.

Thrinaxodon liorhinus Character = 3 NHMUK R511.

Platycraniellus elegans Character = 3 A sshort projection is clear in TM 25, altough is not evident in Abdala (2007) illustrations.

Cynognathus crateronotus Character = 3 Broili and Schroder (1935. figs. 1 and 2).

Diademodon tetragonus Character = 3 Changed from 0 to 1 after RC 112/P1, USNM V23352. See also (Hopson and Kitching, 2001: fig. 6C).

Trirachodon spp. Character = 3 BP/1/4658.

Langbergia modisei Character = 3 Changed from 0 to 1. Abdala et al. (2006, see fig. 3) identified the the tip of the posterodorsal process of the septomaxilla in NMQR 3255 embraced in the anterodorsal of the maxilla. The same condition is observed in SAM-PK-11481 and BP/1/5363. Liu and Olsen (2010, appendix) quoting the NMQR 3255 scored as long.

Sinognathus gracilis Character = 3 Changed from 0 to 1. the condition in IVPP V2339 is comparable to that of *Thrinaxodon liorhinus* and then short. .

Beishanodon youngi Character = 3 Gao et al., 2010: figs. 3A, 4A, C).

Mandagomphodon attridgei Character = 3 After unpublished figure of Jim Hopson.

Andescynodon mendozensis Character = 3 Changed from ? to 1.

Aleodon cromptoni Character = 3 Changed from 1 to 0.

Probainognathus jenseni Character = 3 Changed from 1 to 0. .

Boreogomphodon jeffersoni Character = 3 Scored after NCSM 20698.

Traversodon stahleckeri Character = 3 Changed frorm 0 to 1.

Brasilodon tetragonus Character = 3 Changed from ? to 0.

Dadadon isaloi Character = 3 Changed from ? to 1.

Menadon besairiei Character = 3 Changed from ? to 1.

Trucidocynodon riograndensis Character = 3 Changed from 0 to 1.

Bienotherium spp. Character = 3 Changed from 0 to 1 according to Young, 1947.

Dvinia prima Character = 3 Abdala: After Sushkin, 1929: fig. 1. Konjukova, 1946: fig. 1), reconfirmed after reconstruction by Jim Hopson, clearly visible on the right side.

Nanictosaurus kitchingi Character = 3 Changed from ? to 1 (TM279).

Cricodon metabolus Character = 3 I could not check this scoring in C. metabolus. Long in SAM-PK-12168.

4. Prefrontal (Bone located lateral to the frontal and anterior to the postorbital forming the anterodorsal margin of the orbit.)

0 present

1 absent

Procynosuchus delaharpeae Character = 4 RC 92, BPI/1/3747.

Galesaurus planiceps Character = 4 BP/1/4602, NMQR 3542, NMQR 860. All specimens with the area preserved.

Platycraniellus elegans Character = 4 Abdala (2007, text-fig. 1A, 2A).

Beishanodon youngi Character = 4 Gao et al. (2010: figs. 3A, C, 4A, C).

Mandagomphodon hirschsoni Character = 4 Hopson (2014: fig.14.1).

Scalenodontoides macrodontes Character = 4 Sutures are not clear in the specimens but there are some ridges suggesting the presence of the prefrontal separating the frontal from the orbits. This was interpreted by Gow and Hancox (1993) and Battail (2005) and it is followed here.

Boreogomphodon jeffersoni Character = 4 Changed from ? to 0.

Brasilodon tetragonus Character = 4 Wallace et al 2019 proposed changing this to 0 after Ruf et al 2014. They identify a prefrontal in a CT with doubts.

Dadadon isaloi Character = 4 Changed from ? to 0.

Nanictosaurus kitchingi Character = 4 Changed from 1 to 0.

Tessellatia bonapartei Character = 4 Yo creo que definitivamente está ausente (1). Pero Fer tiene dudas entonces lo paso a ?.

Bonacynodon schultzi Character = 4 This is not observable or mentioned by Martinelli et al 2016. Changed from 0 to ?.

Protheriodon estudianti Character = 4 Changed from ? to 1.

Prozostrodon brasiliensis Character = 4 Changed from 1 to 0 after Wallace et al 2019 who stated that Bonaparte and Barberena, 2001 figured the prefrontal as present.

5. Prefrontal postorbital contact (Suture between these bones is usually on the dorsal margin of the orbits, but the frontal can be interposed negating the suture between prefrontal and postorbital. These bones are evolutionary lost in later therapsids.)

0 absent

1 present

Procynosuchus delaharpeae Character = 5 RC 92, BPI/1/3748.

Platycraniellus elegans Character = 5 Abdala (2007, text-fig. 1A, 2A).

Beishanodon youngi Character = 5 Gao et al. (2010: figs. 3A, C, 4A, C).

Mandagomphodon hirschsoni Character = 5 Hopson (2014: fig.14.1).

Gomphodontosuchus brasiliensis Character = 5 Changed from 1 to ?.

Boreogomphodon jeffersoni Character = 5 Changed from ? to 1.

Dadadon isaloi Character = 5 Changed from ? to 1.

Morganucodon spp. Character = 5 Changed from 0 to -.

Cricodon metabolus Character = 5 Changed from ? to 1 following figs by Sidor and Hopson, 2018.

Pseudotherium argentinus Character = 5 Changed from ? to 0.

6. Postorbital (This bone forms the postorbital bar and the posterodorsal margin of the orbit. Postorbital is evolutionary lost resulting in the coalescence of the orbit and temporal opening.)

0 present and forms postorbital bar

1 present but does not form postorbital bar

2 absent

Charassognathus gracilis Character = 6 From paper Botha et al 2007.

Galesaurus planiceps Character = 6 BP/1/4602, NMQR 3542, NMQR 860. All specimens with the area preserved.

Platycraniellus elegans Character = 6 Abdala (2007, text-fig. 1A, 2A).

Beishanodon youngi Character = 6 Gao et al. (2010: figs. 3A, C, 4A, C).

Mandagomphodon hirschsoni Character = 6 Hopson (2014: fig.14.1).

Chaliminia musteloides Character = 6 The presence of the postorbital is uncertain (Bonaparte 1980).

Bienotherium spp. Character = 6 This is not preserved in the holotypes. I would change this to ? as I could not check the scoring.

Cricodon metabolus Character = 6 Changed from ? to 0 after C. metabolus NHCC LB28.

Protheriodon estudianti Character = 6 Changed from ? to 2.

Prozostrodon brasiliensis Character = 6 Changed from 2 to 1 after Wallace et al 2019 who stated that Bonaparte and Barberena, 2001 figured the postorbital as present.

7. Parietal pineal foramen (This opening is located on the temporal region of the skull surrounded by the parietal bone and lodging the pineal eye. The foramen is represented in several living diapsids but is unknown in living mammals.)

0 present

1 absent

Galesaurus planiceps Character = 7 BP/1/4602, NMQR 3542, NMQR 860. All specimens with the area preserved.

Platycraniellus elegans Character = 7 Abdala (2007, text-fig. 1A, 2A).

Beishanodon youngi Character = 7 Gao et al. (2010: figs. 3A,4A).

Massetognathus spp. Character = 7 Changed from 1 to 0. It is present at least in some specimens (eg., MCZ 3807). See also Romer, 1967.

Dadadon isaloi Character = 7 Kammerer et al 2012 illustrates a pineal foramen in the reconstruction of the juvenile of *Dadadon isaloi*. However, they do not mention or figure in which specimen this is visible. The adult specimens lack a pineal foramen. Ranivoharimanana et al 2011 said that young individuals have a pineal opening.

Menadon besairiei Character = 7 Changed from ? to 1.

Dvinia prima Character = 7 Abdala: I consider as the true foramen the vacuity mentioned by Tatarinov: the ?foramen parietale? mentioned by Sushkin and illustrated by Konzhukova for PIN 2005/2469 is actually merely a vacuity at the anterior end of the sagittal crest bordered on the sides by the edges of the frontal rising up to form a crest. This vacuity also occurs in PIN 2005/2465. A true parietal foramen is lacking in *Dvinia prima*, probably because of the intensive development of the sagittal crest? (Tatarinov, 1968: 8). After observation of the cast v98136 in Berkeley it seems that the foramen is really absent as indicated by Tatarinov (1968). So I must change to (1) in the DM. Addenda, in reconstruction by Jim Hopson the foramen is present and he also have described it in the text, although with some uncertainty. His final decision is based on the condition present in others basal cynodonts. This certainly is advisable but it must be considered that the observation of this feature in the material of the taxon is then ambiguous. I will score as present following Jim decision. Present (0).

Cricodon metabolus Character = 7 Changed from ? to 0. Present in C. kannemeyeri (SAM-PK-12168). .

8. Parietal region (Central portion of the temporal region of the skull formed by the parietal and sometimes by a small portion of the postorbital, where is located the sagittal crest.)

0 at same level as remaining skull profile

1 high

Procynosuchus delaharpeae Character = 8 The score is variable. For example the parietal region appears elevated in RC 5, RC12,RC 72, RC 87, RC 92, RC 130, RC 304 SAM Pk K8511 and BP/1/3748. But not in all specimens (e.g., BP/1/2600 or NHMUK PV37054. However in larger specimens is present and we scored like that.

Galesaurus planiceps Character = 8 BP/1/4602, NMQR 3542, NMQR 860. All specimens with the area preserved. It should be said that several specimens show the frontal and parietal region in the middle of the skull ascending (see for example Jasinoski and Abdala, 2017, fig.2), but these condition is completely different to the abrupt elevation of the parietal scored in *Dvinia prima* and *Procynosuchus delaharpeae*.

Platycraniellus elegans Character = 8 Abdala (2007, text-fig. 1D,E, 2D,E).

Beishanodon youngi Character = 8 Gao et al. (2010: figs. 3C, 4C).

Aleodon brachyramphus Character = 8 Changed from 0 to ?.

Therioherpeton cargnini Character = 8 Changed from 1 to 0.

Boreogomphodon jeffersoni Character = 8 Changed from ? to 0.

Chaliminia musteloides Character = 8 Changed from ? to 0.

Riograndia guaibensis Character = 8 Changed from 1 to 0.

Dadadon isaloi Character = 8 Changed from ? to 0 (although the parietal region is slightly higher, it is not comparable to what is observed in *Oligokyphus* spp. for example).

Bienotherium spp. Character = 8 Changed from ? to 1.

Cricodon metabolus Character = 8 Changed from ? to 0. This is observed in *C. kannemeyeri* (SAM-PK-12168) and inferred for *C. metabolus* (NHCC LB28).

Bonacynodon schultzi Character = 8 Changed from ? to 0.

Prozostrodon brasiliensis Character = 8 Changed from ? to 0.

Pseudotherium argentinus Character = 8 Changed from ? to 0.

9. Posterior extension of parietal (Refers to the limitation of the parietal to the sagittal crest or its extension posteriorly to form part of the occipital crest.)

0 anterior to or reaching the origin of the occipital crests

1 posterior to the origin of the occipital crests

Charassognathus gracilis Character = 9 Changed from ? to 0.

Galesaurus planiceps Character = 9 NMQR 1451, BP\1\4602^n^n.

Platycraniellus elegans Character = 9 The squamosal extends very near to the origin of the occipital crest. There is only a very small portion of the parietal after the end of the sagittal crest. This was scored as 0 by Abdala (2007) and the condition is certainly different from most other cynodonts in which the parietal forms a more extensive portion of the occipital crest. However, considering that the parietal is passing the posterior end of the sagittal crest is that we change the score to 1.

Diademodon tetragonus Character = 9 State 0 in BSP 1934 VIII19, State 1 in BSP 1934 VIII14, BSP 1934 VIII20, SAM-PK-K5222.

Trirachodon spp. Character = 9 Posterior AM 461, CGP JNN 2000/7/2A. Anterior BP/1/4661 and BP/1/4658.

Langbergia modisei Character = 9 Abdala et al. (2006, fig. 2). SAM-PK-11481.

Beishanodon youngi Character = 9 Following this description of Gao et al (2010: 10) we score tentatively as present: Posterolaterally, a thickened posttemporal process of the parietal diverges from the intertemporal crest and runs posteroventrally toward the posterior extremity of the temporal fenestra. .

Luangwa spp. Character = 9 UFRGS 267PV.

Scalenodontoides macrodontes Character = 9 Tentative score after Battail (2005, figure 8, left side).

Pascualgnathus polanski Character = 9 Changed from ? to 1.

Andescynodon mendozensis Character = 9 Changed from ? to 1.

Protuberum cabralense Character = 9 Changed from ? to 1.

Therioherpeton cargnini Character = 9 Changed from 0 to 1.

Boreogomphodon jeffersoni Character = 9 Changed from ? to 1.

Traversodon stahleckeri Character = 9 Changed from ? to 1 after von Huene 1936.

Brasilodon tetragonus Character = 9 Changed from ? to 1.

Dadadon isaloi Character = 9 Changed from ? to 1.

Menadon besairiei Character = 9 Probably 1 but not preserved.

Ecteninion lunensis Character = 9 Changed from 1 to 0.

Chiniquodon spp. Character = 9 Changed from 1 to 0.

Bienotherium spp. Character = 9 Changed from 1 to 0.

Diarthrognathus broomi Character = 9 Changed from ? to 1.

Dvinia prima Character = 9 Changed from ? to 0. Abdala: Following drawing by Hopson, the posterior border of the parietal seems to reach the origin of the occipital crest. Then based in this I score this state.

Progalesaurus lootbergensis Character = 9 Changed from 1 to 0.

Nanictosaurus kitchingi Character = 9 Changed from ? to 1. Observed on the specimens (contra van Heerden 1976 fig 1).

Cricodon metabolus Character = 9 Changed from ? to 1. Posterior to the occipital crests in C. kannemeyeri (SAM-PK-12168).

Pseudotherium argentinus Character = 9 Changed from ? to 1.

10. Snout in relation to temporal region (This compares the snout length, measured form the tip of the skull to the anterior margin of the orbit, versus the temporal length, measured from the posterior margin of the postorbital bar until the posterior tip of the parietal crest (see Abdala and Giannini, 2002, text-fig. 4).)

0 longer

1 subequal

2 shorter

Procynosuchus delaharpeae Character = 10 See Abdala and Giannini (2002, text-fig. 7).

Galesaurus planiceps Character = 10 Meaurements after photos in dorsal view using Digimizer. NMQR 860 is subequal (snout is 1.04 of the temporal region length). AMNH FARB 2223 is subequal (snout is 1.05 of the temporal region) NMQR 1451 is longer (snout is1.29 of temporal length). We scored here following the condition in the largest specimen of the taxon.

Thrinaxodon liorhinus Character = 10 Changed from 01 to 0 (see Abdala and Giannini, 2002, text-fig. 7).

Platycraniellus elegans Character = 10 Snout is 39% of basal skull length, Temporal region is 40% of basal skull length (Abdala, 2007, table 1).

Cynognathus crateronotus Character = 10 Abdala and Giannini (2002, text-fig. 7). .

Diademodon tetragonus Character = 10 Abdala and Giannini (2002, text-fig. 7). .

Trirachodon spp. Character = 10 Measurment with digimizer in BP/1/4658 are temporal length 5 cm and snout 3.9 cm. Skull length is 9.6 cm (Abdala et al., 2006, table 4). Snout is 41% and temporal length is 52% of the basal skull length. Changed from 1 to 2. .

Langbergia modisei Character = 10 Abdala et al. (2006, table 2).

Beishanodon youngi Character = 10 Gao et al. (2010: figs. 3A,4A).

Scalenodon spp. Character = 10 Measurment with digimizer in UMZC T.907 are temporal length 6.7 cm and snout estimated 6 cm. Estimated skull length is 13 cm (Abdala and Teixeira, 2004). Snout is 46% and temporal length is 51% of the basal skull length. Changed from 1 to 2 Tentative score.

Luangwa spp. Character = 10 Measurment with digimizer in BP/1/3731 are temporal length 3.9 cm and snout 5.3 cm. Skull length is 13.6 cm (Abdala and Teixeira, 2004). Snout is 40% and temporal length is 29% of the basal skull length. Changed from 1 to 2 Tentative score.

Scalenodontoides macrodontes Character = 10 Measured with digimizer on BP/1/5395. Tentative as the snout as the snout length is an estimation.

Aleodon brachyramphus Character = 10 Changed from 1 to ?.

Lumkuia fuzzi Character = 10 Changed from 1 to 2.

Traversodon stahleckeri Character = 10 Changed from ? to 2 after von Huene 1936.

Riograndia guaibensis Character = 10 Changed from 1 to 2.

Massetognathus spp. Character = 10 Changed from 1 to 1+2 as it is subequal in some specimens (e.g., MCZ3810) and shorter in others (eg., MCZ 3807. see also Romer 1967).

Dadadon isaloi Character = 10 Changed from 1 to 1/2 (it is subequal in .

Exaeretodon spp. Character = 10 In some specimens the snout is shorter (e.g., PVSJ 707).

Elliotherium kersteni Character = 10 Changed from ? to 2. Inferred.

Pachygenelus monus Character = 10 I am not sure about this scoring. The snout seems shorter than the temporal region.

Dvinia prima Character = 10 Measured from Tatarinov (1968: fig. 1 and Konjukova (1946: fig. 1). This was confirmed with the cast: Snout: 39.88 mm. temporal region: 49.54 mm.

Cynosaurus suppostus Character = 10 This depends on the specimen. There are specimens (e.g., BP/1/3926) in which the snout is shorter or subequal to the temporal region. In others, the temporal region is longer (e.g., BP/1/4469).

Cricodon metabolus Character = 10 Changed from ? to 0. Longer in C. kannemeyeri (SAM-PK-12168).

11. Frontal epipterygoid contact (This contact is located in the medial cranial wall, behind the orbit. In some nonmammaliaform cynodonts there are projections of the epipterygoid and frontal that are very close and can be interpreted as contacting. There are cases scored as absent that should perhaps due to the loss of the processes by fossilization or because they are covered by the ventral projection of the postorbital.)

0 absent

1 present

Charassognathus gracilis Character = 11 Changed from ? to 0.

Procynosuchus delaharpeae Character = 11 Scored after Kemp (1979, fig. 13), observed in RC 92.

Galesaurus planiceps Character = 11 NMQR 135. AMNH FARB 2227. Pusch et al. (2019).

Cynognathus crateronotus Character = 11 Changed from 0 to 1. Illustration by Broili and Schroeder (1934, Fig. 4) show absence of contact. This is representing the condition of BSP 1934 VIII2 (with a beautifully preserved and completely prepared lateral wall of the skull). Direct observation of the specimen however show that the anterodorsal margin of the epipterygoid is contacting a long posteroventral projection of the frontal.

Diademodon tetragonus Character = 11 Changed from 0 to 1. After observation of MB R1004, BSP 1934 VIII19 and UMCP 42446. In all of them the anterodorsal projection of the epipterygoid y well extended anteriorly and seems to contact the frontal.

Trirachodon spp. Character = 11 SAM-PK-K7888, SAM-PK-K4801.

Langbergia modisei Character = 11 Changed from 1 to 0. Liu and Olsen (2010, appendix) scored 1 after NMQR 3255, but the contact is not observed in the specimen (Abdala et al., 2006. Fig. 5). .

Sinognathus gracilis Character = 11 IVPP V2339.

Mandagomphodon hirschsoni Character = 11 Relevant area for scoring not preserved. Changed from 1 to ?.

Riograndia guaibensis Character = 11 Changed from 1 to ? (not preserved after Soares et al 2011).

Dadadon isaloi Character = 11 Changed from ? to 0. .

Menadon besairiei Character = 11 Changed from ? to 0. It is not described but the contact is apparently absent.

Exaeretodon spp. Character = 11 Changed from ? to 0.

Oligokyphus spp. Character = 11 Changed from ? to 0.

Dvinia prima Character = 11 Changed from ? to 0.

Cynosaurus suppostus Character = 11 Changed from 1 to 0.

Prozostrodon brasiliensis Character = 11 Changed from ? to 1.

12. Epipterygoid ascending process (Dorsal projection of the epipterygoid the contact posteriorly the prootic and is part of the lateral wall of the cranium.)

0 rodlike

1 moderately expanded

2 greatly expanded

Procynosuchus delaharpeae Character = 12 RC 5, NHMUK PV37054, Kemp (1979: fig.16). .

Galesaurus planiceps Character = 12 Several specimens with the area preserved.

Platycraniellus elegans Character = 12 Observed on both sides of TM 25.

Scalenodontoides macrodontes Character = 12 BP/1/5395.

Protuberum cabralense Character = 12 Changed from ? to 0.

Riograndia guaibensis Character = 12 Changed from 2 to ? (not preserved after Soares et al 2011).

Dadadon isaloi Character = 12 Changed from ? to 2.

Menadon besairiei Character = 12 Changed from ? to 2.

Ecteninion lunensis Character = 12 Changed from 2 to 1.

Trucidocynodon riograndensis Character = 12 Changed form 2 to 1.

Kayentatherium wellesi Character = 12 I am not sure about this scoring. I would say it is moderately expanded (1).

Elliotherium kersteni Character = 12 Should be 1 or 2. Changed from ? to 1/2.

Diarthrognathus broomi Character = 12 Changed from ? to 2.

Dvinia prima Character = 12 Changed from 2 to 1.

Bonacynodon schultzi Character = 12 Changed from ? to 1.

13. Prootic canal (Vascular canal enclosing the prootic sinus (middle cerebral vein) at its exit from the cranial cavity (Wible and Hopson, 1995). In nonmammaliaform cynodonts this structure is represented by one or more (three?) openings located in the prootic. The most ventral of these is located on the lateral flange of the prootic just lateral to the primary facial foramen, others openings are also on the prootic, only visible ventrally (they form an intramural canal) in the prootic behind the trigeminal foramen (Wible and Hopson, 1995). Observation of this feature requires a good preservation and preparation

of the base of the prootic and trigeminal region of the bone. The positive documentation of this feature is then not always possible.)

0 absent

1 present

Procynosuchus delaharpeae Character = 13 NHMUK PV37054, Kemp (1979: fig.16).

Galesaurus planiceps Character = 13 AMNH FARB 2223.

Thrinaxodon liorhinus Character = 13 Fourie (1974).

Platycraniellus elegans Character = 13 Abdala (2007, text-fig. 3).

Cynognathus crateronotus Character = 13 Broili and Schroeder (1934, Tafel III), BSP 1934 VIII4.

Diademodon tetragonus Character = 13 UMCP 42446, BSP 1934 VIII20.

Trirachodon spp. Character = 13 SAM-PK-K5821.

Scalenodon spp. Character = 13 We follows Liu and Olsen (2010, appendix) who score absent based in MCZ8905, (Parrington, 1946: fig.7). From the figure it sems clear that the foramen was absent, but the assignment of the braincase to *Scalenodon* spp. is not secure. I did not see specimen from the MCZ quoted.

Luangwa spp. Character = 13 Corrected from 1 to ? as scored by Liu and Olsen (2010, character 55).

Protuberum cabralense Character = 13 Changed from 1 to ?.

Brasilodon tetragonus Character = 13 It is present according to Rodrigues et al 2013. contra Bonaparte et al 2005 (it is said to be absent in *Brasilodon tetragonus* and Brasilitherium by Bonaparte et al 2005).

Riograndia guaibensis Character = 13 Changed from 0 to ? .

Dvinia prima Character = 13 Changed from ? to 0. .

Cynosaurus suppostus Character = 13 Changed from ? to 0.

Bonacynodon schultzi Character = 13 Changed from ? to 1.

Pseudotherium argentinus Character = 13 Changed from ? to 1.

14. Trigeminal exit (The maxillary and mandibular branches (V2, V3) of the trigeminal nerve leave the skull through the trigeminal foramen. Usually the trigeminal (Gasserian or semilunar) ganglion is located medial to the prootic and epipterygoid and the opening is also, in general, located between these bones.)

0 between prootic incisure and epipterygoid

1 via foramen between epipterygoid and prootic

2 via two foramina

Procynosuchus delaharpeae Character = 14 RC 5, Kemp (1979: fig. 16).

Galesaurus planiceps Character = 14 NMQR 1451, AMNH FARB 2223.

Thrinaxodon liorhinus Character = 14 Fourie (1974).

Platycraniellus elegans Character = 14 Observed on both sides of TM 25.

Cynognathus crateronotus Character = 14 BSP 1934 VIII2.

Diademodon tetragonus Character = 14 After Liu and Olsen (2010, appendix): (0&1) AMNH 7638, BP/1/4677, (Watson, 1911) fig5, (Brink, 1955) fig3.

Trirachodon spp. Character = 14 Gow (1986: fig. 3A).

Langbergia modisei Character = 14 Abdala et al., (2006: 394): Only one opening for the exit of the trigeminal nerve is observed between the alisphenoid and prootic in NMQR 3255, but the damaged condition of the epipterygoid in this specimen hampers confidence in the definitive number of openings. Two openings are present in the small NMQR 3280, the ventral one being more developed.

Scalenodon spp. Character = 14 Parrington (1946, fig. 7). Tentative score by taxonomic uuncertainty.

Luangwa spp. Character = 14 Tentative score based in UFRGS 267PV. However the bones preservation is not good with several cracks.

Scalenodontoides macrodontes Character = 14 After Gow and Hancox (1993. fig. 2).

Pascualgnathus polanski Character = 14 Changed from 2 to 1.

Protuberum cabralense Character = 14 Changed from ? to 1.

Riograndia guaibensis Character = 14 Changed from 1 to ? (not preserved according to Soares et al 2011).

Massetognathus spp. Character = 14 Changed from 2 to 1. See for example Rougier et al 1992.

Dadadon isaloi Character = 14 Changed from ? to 2.

Menadon besairiei Character = 14 Changed from ? to 1.

Exaeretodon spp. Character = 14 Changed from 2 to 1.

Bienotherium spp. Character = 14 Changed from ? to 1.

Dvinia prima Character = 14 Abdala: See Tatarinov (1968: fig. 1). The preservation of this area is not complete following Hopson's illustrations, but considering that the pila antotica and the incisure prootic are preserved and also the bones above that incisure we can assume that there was only one (apparently large) trigeminal foramen in the lateral wall of the skull. Then one foramen stand. Gaetano: it is described by Ivakhnenko 2013 as a fenestra, I would score this as (0) as it does not look like a foramen.

15. Prootic and opisthotic (These two bones get fused to form the petrosal bone (part of the temporal bone) of mammals and houses elements of the inner ear. Rowe (1988, pg. 250) proposed this feature and consider complete fusion of prootic and opistothic forming the petrosal bone starting with tritylodontids. Wible (1991, pg. 3) mention that in tritheledontids the situation is not clear: Crompton (1958: 153) described *Diarthrognathus broomi* lacking sutures between these bones, whereas clear sutures were observed by Wible (1991) in a juvenile of *Pachygenelus monus* (SAM-PK-K1350) but in the only adult specimen known of this taxon (SAM-PK-K1329) the condition is unclear. This is not easy to score because depends of preservation and preparation. By general the suture between prootic and opisthotic, when separated, is more clearly noted externally in the surface near the stapedial foramen.)

0 separated

1 fused to form petrosal

Procynosuchus delaharpeae Character = 15 It is difficult to see this suture in the material (in comparison with for example specimens of *Thrinaxodon liorhinus*). It seems to be present in NHMUK PV37054, illustrated by Kemp (1979, fig. 2) and we score as present.

.

Galesaurus planiceps Character = 15 NMQR 135, AMNH FARB 2223.

Thrinaxodon liorhinus Character = 15 Fourie (1974, fig. 24), NHMUK R511a.

Platycraniellus elegans Character = 15 Abdala (2007: 596): .. but the morphology of this region can be observed in NMQR 1633. Laterally, the suture between the prootic and the opisthotic in the fenestra ovalis is visible.

Cynognathus crateronotus Character = 15 BP/1/1181, SAM-PK?11264.

Diademodon tetragonus Character = 15 UCMP 42446.

Trirachodon spp. Character = 15 SAM-PK-K4801.

Langbergia modisei Character = 15 Tentative score after SAM-PK-11481.

Sinognathus gracilis Character = 15 IVPP V2339.

Scalenodon spp. Character = 15 Scored by Liu and Olsen (2020, appendix) quoting MCZ 8905.

Luangwa spp. Character = 15 NHMUK PV R 36995.

Scalenodontoides macrodontes Character = 15 Changed from 0 to ? Very poor preservation in the known skulls.

Andescynodon mendozensis Character = 15 Changed from ? to 0. MicroCt on loan in FZB.

Protuberum cabralense Character = 15 Changed from ? to 0.

Lumkuia fuzzi Character = 15 Changed from 1 to 0 after Hopson and Kitching 2001.

Brasilodon tetragonus Character = 15 Bonaparte et al 2005.

Dadadon isaloi Character = 15 Changed from ? to 0.

Menadon besairiei Character = 15 Changed from ? to 0.

Diarthrognathus broomi Character = 15 Changed from 0 to 1 (see Crompton 1958: p. 193).

Dvinia prima Character = 15 Changed from 1 to 0. Abdala: ?The two otic bones are completely fused into a typically mammalian periotic, and we can speak only tentatively of any distinction between prootic and opistotic (Tatarinov, 1968: 16). Also from the figures 2 and 4 it is not possible to distinguish sutures between these elements. Maybe

question mark? After notes of Hopson: However, an ossified zone medial to the fenestra ovalis marks the separation of the anterior prootic from the posterior opisthotic. Change the state to separated (0).

16. Internal auditory meatus (Scoring of this character necessitates observation of the inner brain cavity. The pars cochlearis forms a complete medial bony wall to separate the inner ear space from the brain cavity, which in turn defines the internal auditory meatus with an ossified floor for the vestibulo-cochlear nerve (= vestibulo-acoustic cranial nerve VIII). This differs from the incomplete ossification of the medial wall of the periotics in basal cynodonts (Luo et al., 2016). The general idea from the eighties is that this wall was absent in most non-mammalian cynodonts excepts tritylodontids (in particular *Oligokyphus* spp. described by Kühne, 1954) and also in tritheledontids following Hopson (1991) and Wible (1991), who mentioned this condition for the family but there is no internal cranial description of tritheledontids until now. Open in this character is meaning absence of an osseous wall separating the inner ear space from the brain cavity.)

0 open

1 walled

Procynosuchus delaharpeae Character = 16 Kemp 1979: fig. 13.

Galesaurus planiceps Character = 16 Rigney (1938), Pusch et al. (2019, fig. 14).

Thrinaxodon liorhinus Character = 16 Fourie (1974, Fig. 28).

Diademodon tetragonus Character = 16 Gow (1986, fig.5).

Trirachodon spp. Character = 16 Gow (1986, fig. 3).

Luangwa spp. Character = 16 Abdala and Teixeira (2004.

Aleodon brachyramphus Character = 16 Changed from 1 to ?.

Aleodon cromptoni Character = 16 Changed from 1 to ?.

Brasilodon tetragonus Character = 16 Changed from ? to 1.

Dadadon isaloi Character = 16 Changed from ? to 0.

Menadon besairiei Character = 16 Changed from ? to 0.

Ecteninion lunensis Character = 16 Changed from ? to 0.

Chiniquodon spp. Character = 16 I was unable to check this scoring.

Sinoconodon rigneyi Character = 16 Changed from ? to 1.

Dvinia prima Character = 16 Changed from ? to 0. (after Ivakhnenko 2013).

17. Zygomatic arch dorsoventral height (This intends to reflect differences in height (and in some way also robustness) of the zygoma. It is a visual feature that was not possible to convey to a numerical ratio (using either skull length or zygoma length for the intended ratios did not reflected the height of the zygoma as perceived visually).

0 slender

1 moderately deep

2 very deep

Galesaurus planiceps Character = 17 BP/1/5064, NMQR 1451.

Platycraniellus elegans Character = 17 Abdala (2007: text-figs. 1D,E, 2D,E).

Cynognathus crateronotus Character = 17 BP/1/4464.

Diademodon tetragonus Character = 17 UCMP 42446, MB R1004.

Trirachodon spp. Character = 17 BP/1/4658, BP/1/1181.

Langbergia modisei Character = 17 NMQR 3255, 3256, .

Sinognathus gracilis Character = 17 IVPP V2339 .

Beishanodon youngi Character = 17 Gao et al. (2010: figs. 3C,4C).

Scalenodon spp. Character = 17 Abdala and Teixeira (2004, fig. 8A).

Luangwa spp. Character = 17 BP/1/3731.

Mandagomphodon hirschsoni Character = 17 Hopson (2014: fig.14.3).

Scalenodontoides macrodontes Character = 17 BP/1/5395.

Protuberum cabralense Character = 17 Changed from ? to 1.

Boreogomphodon jeffersoni Character = 17 Changed from ? to 1.

Traversodon stahleckeri Character = 17 Changed from ? to 2 after von Huene 1936.

Dadadon isaloi Character = 17 Changed from ? to 2. This is difficult to score as it is no clear cut between states.

Ecteninion lunensis Character = 17 Changed from 0 to ?. The zygomatic arch was probably slender but it is not preserved.

Bienotherium spp. Character = 17 I could not check this scoring. This is not visible in the holotypes of *Bienotherium* spp..

Cricodon metabolus Character = 17 Changed from ? to 2. Very deep in C. kannemeyeri (SAM-PK-12168).

Tritheledon riconoi Character = 17 Moderatelly deep or very deep.

18. Anteroventral corner of zygomatic arch (This was scored as higher when the suborbital ramus of the zygomatic arch was higher than the dental line. As example compare *Sinoconodon rigneyi* (higher) versus *Langbergia modisei* (same level).)

0 lies at same level as

1 or lies significantly higher than postcanine line

Charassognathus gracilis Character = 18 From paper Botha et al 2007.

Procynosuchus delaharpeae Character = 18 BP/1/3748, RC 92.

Galesaurus planiceps Character = 18 SAM-PK-K10465, TM 83, NMQR 3542.

Thrinaxodon liorhinus Character = 18 Parrington (1946, fig. 10).

Platycraniellus elegans Character = 18 Abdala (2007: text-figs. 1D, 2D,).

Cynognathus crateronotus Character = 18 BSP 1934VIII6.

Diademodon tetragonus Character = 18 MB R1004.

Trirachodon spp. Character = 18 Changed to 0 and 1. 0 after BP/1/4658, 1 after BP/1/4661.

Langbergia modisei Character = 18 Changed from 1 to 0. Abdala et al. (2006, fig.3 and fig. 9).

Sinognathus gracilis Character = 18 Sun (1988, fig. 2).

Beishanodon youngi Character = 18 Gao et al. (2010: figs. 3C,4C).

Scalenodon spp. Character = 18 Abdala and Teixeira (2004, fig. 8).

Luangwa spp. Character = 18 NHMUK PV R 36995.

Mandagomphodon hirschsoni Character = 18 Changed from 1 to 0. Hopson (2014: fig.14.1c).

Mandagomphodon attridgei Character = 18 NHMUK R8578.

Gomphodontosuchus brasiliensis Character = 18 Hopson (1985, fig. 2).

Scalenodontoides macrodontes Character = 18 BP/1/5395.

Aleodon brachyramphus Character = 18 Changed from 0 to 1.

Aleodon cromptoni Character = 18 Changed from 0 to 1.

Therioherpeton cargnini Character = 18 Changed from 1 to 0.

Boreogomphodon jeffersoni Character = 18 Changed from ? to 0.

Cricodon metabolus Character = 18 Changed from ? to 0 after C. metabolus NHCC LB28 (it is inferred to be like this as the skull is distorted).

Bonacynodon schultzi Character = 18 Changed from 1 to 0.

19. Maximum dorsal extent of zygomatic arch (Extent of the dorsal margin of the zygomatic arch in the temporal region, in relation to the orbit.)

0 below middle of orbit

1 above middle of orbit but below upper border

2 above upper border of orbit

Charassognathus gracilis Character = 19 From paper Botha et al 2007.

Procynosuchus delaharpeae Character = 19 BP/1/3748, RC 92.

Galesaurus planiceps Character = 19 SAM-PK-K10465, TM 83, NMQR 3542, NMQR 860, BP/1/4602, AMNH FARB 2223. But in NMQR 1451 is above middle of the orbit. We score the state more represented.

Thrinaxodon liorhinus Character = 19 TM 167, TM 180.

Platycraniellus elegans Character = 19 Abdala (2007: text-figs. 1D,E, 2D,E).

Cynognathus crateronotus Character = 19 BSP 1934VIII6.

Diademodon tetragonus Character = 19 MB R1004.

Trirachodon spp. Character = 19 BP/1/4658.

Langbergia modisei Character = 19 NMQR 3255.

Sinognathus gracilis Character = 19 IVPP V2339.

Beishanodon youngi Character = 19 Gao et al. (2010: figs. 3C,4C).

Scalenodon spp. Character = 19 UMZC T. 907.

Luangwa spp. Character = 19 BP/1/3731.

Scalenodontoides macrodontes Character = 19 Changed from 0 to 1. BP/1/5395.

Traversodon stahleckeri Character = 19 Changed from ? to 1 after von Huene 1936.

Chaliminia musteloides Character = 19 Changed from ? to 0.

Ecteninion lunensis Character = 19 Changed from 0 to ?. Not preserved.

Tritylodon longaevus Character = 19 I am not sure about this scoring. It is not possible to know if it should be scored as 1 or 2 because the orbit is not completetelly closed.

Bienotherium spp. Character = 19 I could not check this scoring. This is not visible in the holotypes of *Bienotherium* spp..

Cricodon metabolus Character = 19 Changed from ? to 1. Above the middle of the orbit in *C. kannemeyeri.*

20. Infraorbital process (Here are considered the suborbital angulation of the jugal and the suborbital (or descendant) process of the jugal that are located in similar places and interpreted as connected with origin of occlusal muscles.)

0 absent

1 suborbital angulation between maxilla and jugal

2 descendant process of jugal

Charassognathus gracilis Character = 20 Changed from ? to 0.

Procynosuchus delaharpeae Character = 20 BP/1/3748, RC 92, NHMUK PV37054.

Galesaurus planiceps Character = 20 BP/1/5064, NMQR 1451, NMQR 860.

Thrinaxodon liorhinus Character = 20 TM 180.

Platycraniellus elegans Character = 20 Abdala (2007: 596): The ventral edge of the posterior portion of the maxilla presents an angle of c. 120 degrees in relation to the anteroventral margin of the jugal.

Cynognathus crateronotus Character = 20 BSP 1934VIII6.

Diademodon tetragonus Character = 20 MB R1004.

Trirachodon spp. Character = 20 BP/1/4658.

Langbergia modisei Character = 20 NMQR 3256.

Sinognathus gracilis Character = 20 IVPP V2339.

Beishanodon youngi Character = 20 Changed from ? to 1. Gao et al. (2010: figs. 3C,4C).

Scalenodon spp. Character = 20 UMZC T. 907.

Luangwa spp. Character = 20 NHMUK PV R 36995.

Scalenodontoides macrodontes Character = 20 Changed from ? to 0. Tentative scoring after BP/1/5395.

Andescynodon mendozensis Character = 20 Changed from ? to 0.

Protuberum cabralense Character = 20 Changed from ? to 2.

Boreogomphodon jeffersoni Character = 20 Changed from ? to 0.

Traversodon stahleckeri Character = 20 Changed from ? to 2 after Barberena 1981.

Brasilodon tetragonus Character = 20 Changed from ? to 0 (Agustin).

Chaliminia musteloides Character = 20 Changed from ? to 0.

Riograndia guaibensis Character = 20 Changed from ? to 0.

Dadadon isaloi Character = 20 In *Dadadon isaloi*: *Dadadon isaloi* differs from most if not all species currently referred to *Massetognathus* spp. in the presence of a large, rounded, ventrally projecting suborbital process (Flynn et al., 2000).

Menadon besairiei Character = 20 Kammerer et al. (2008): *Menadon besairiei* has a descending process of the jugal that contacts the pterygoids and excludes the maxilla from the margin of the subtemporal fenestra, which is present in most gomphodonts, but not in

Exaeretodon spp. or *Scalenodontoides macrodontes* (Abdala et al., 2006). BUT in Brazilian *Menadon besairiei*: suborbital process of jugal absent (Melo et al. 2015, emmended diagnosis).

Nanictosaurus kitchingi Character = 20 Changed from ? to 0.

Cricodon metabolus Character = 20 Descendant process of the jugal in C. kannemeyeri (SAM-PK-12168). Changed from ? to 2.

Tessellatia bonapartei Character = 20 The scoring of this character could be inferred to be: 0 absent.

Prozostrodon brasiliensis Character = 20 Changed from ? to 0.

21. Inferior margin of jugal in the zygoma (This is observing the skull (and zygoma) in lateral view and convey longitudinal extension and also height of the bone.)

0 poorly developed longitudinally not reaching posterior border of zygoma

1 well developed longitudinally and low

2 well developed and high

Procynosuchus delaharpeae Character = 21 RC 92 (left side), NHMUK PV37054 (right side, contra Kemp 1979, fig. 3a).

Galesaurus planiceps Character = 21 BP/1/5064, NMQR 1451, NMQR 860.

Thrinaxodon liorhinus Character = 21 BP/1/7199.

Platycraniellus elegans Character = 21 Abdala (2007, text-fig. 2D, E).

Cynognathus crateronotus Character = 21 BSP 1934VIII6.

Diademodon tetragonus Character = 21 MB R1004.

Trirachodon spp. Character = 21 BP/1/4658.

Langbergia modisei Character = 21 Changed from ? to 2.

Sinognathus gracilis Character = 21 Changed from ? to 1.

Beishanodon youngi Character = 21 Changed from 0 to 1. The extension of the jugal is posterior very close to the posterior margin of the zygoma and extends to approximately

half of height of the zygoma (therefore not cosnidered as high). The suture resemble that of Chiniquodontids.

Scalenodon spp. Character = 21 Changed from 2 to ? Not possible to know in the only specimen showing the zygoma UMZC T.907.

Luangwa spp. Character = 21 Changed from ? to 2.

Mandagomphodon hirschsoni Character = 21 Changed from ? to 2. (Hopson, 2014: fig. 14.3).

Scalenodontoides macrodontes Character = 21 Changed from 2 to 1. Tentative scoring after BP/1/5395.

Andescynodon mendozensis Character = 21 Changed from ? to 2.

Protuberum cabralense Character = 21 Changed from ? to 1.

Aleodon cromptoni Character = 21 Changed from 1 to 2.

Therioherpeton cargnini Character = 21 Changed from ? to 1.

Traversodon stahleckeri Character = 21 Von Huene 1936 reconstructs this as 1 whereas Barberena 1981 reconstructs this as 2.

Brasilodon tetragonus Character = 21 Changed from ? to 1.

Dadadon isaloi Character = 21 Changed from 0 to 2.

Menadon besairiei Character = 21 Changed from ? to 2. It is described as reaching the squamosal sulcus and the notch for the quadrate and quadratojugal. It appears to be low in the figure by Melo et al 2015 but high in the holotype (see Flynn 2000).

Ecteninion lunensis Character = 21 Changed from 1 to ?. Not preserved.

Bienotherium spp. Character = 21 I could not check this scoring. This is not visible in the holotypes of *Bienotherium* spp..

Diarthrognathus broomi Character = 21 Changed from ? to 1.

Cricodon metabolus Character = 21 Changed from ? to 2. Well developed and high in C. kannemeyeri (SAM-PK-12168).

Protheriodon estudianti Character = 21 Changed from ? to 1.

22. Posterior extension of squamosal dorsal to squamosal sulcus (This is observing the skull (and zygoma) in lateral view and is referring to a projection or lip of the posterodorsal margin of the squamosal that form a sort of roof over the squamosal sulcus.)

0 absent

1 incipient

2 well developed

Charassognathus gracilis Character = 22 Changed from ? to 0.

Procynosuchus delaharpeae Character = 22 RC 92, BP/1/3748.

Galesaurus planiceps Character = 22 BP/1/5064, NMQR 1451, AMNH FARB 2223.

Thrinaxodon liorhinus Character = 22 BP/1/4282, BP/1/5208, BP/1/5905.

Platycraniellus elegans Character = 22 Abdala (2007, text-fig. 2E).

Cynognathus crateronotus Character = 22 changed from 2 to 0. The construction of the temporal region in *Cynognathus crateronotus* is particularly different than in other cynodonts. In this pattern the squamosal is not producing a lip or projection posteriorly. See for example BS 1934 VIII4.

Diademodon tetragonus Character = 22 MB R1004.

Sinognathus gracilis Character = 22 IVPP V2339. Right side.

Beishanodon youngi Character = 22 Gao et al. (2010: figs. 3C,4C).

Scalenodon spp. Character = 22 UMZC T.907. Left zygoma preserved and there is a dorsal lip of the suqmosal that seems to be well developed. Tentative score.

Luangwa spp. Character = 22 BP/1/3731.

Mandagomphodon hirschsoni Character = 22 Hopson, 2014: fig. 14.3.

Scalenodontoides macrodontes Character = 22 BP/1/5395. Tentative score after right side of specimen.

Andescynodon mendozensis Character = 22 Changed from ? to 2.

Protuberum cabralense Character = 22 Changed from ? to 2.

Boreogomphodon jeffersoni Character = 22 Changed from ? to 2.

Traversodon stahleckeri Character = 22 Changed from ? to 2 after von Huene 1936.

Brasilodon tetragonus Character = 22 Changed from ? to 1.

Chaliminia musteloides Character = 22 Changed from ? to 1.

Dadadon isaloi Character = 22 Changed from ? to 2.

Menadon besairiei Character = 22 Changed from ? to 2. .

Diarthrognathus broomi Character = 22 Changed from ? to 0.

Morganucodon spp. Character = 22 I am not sure about this. It could also be absent.

Nanictosaurus kitchingi Character = 22 Changed from ? to 1.

Cricodon metabolus Character = 22 Changed from ? to 2. Well developed in C. kannemeyeri (SAM-PK-12168).

Pseudotherium argentinus Character = 22 Changed from ? to 2.

23. Latero posterior exposure of squamosal on zygoma (The squamosal sulcus (in some hypotheses interpreted as housing an external acoustic meatus, check for example Allin, 1975) is a pronounced sulcus starting on the posterior margin of the zygoma and turning downward to end behind the quadrate.)

0 without or with incipient depression

1 with deep squamosal sulcus

Charassognathus gracilis Character = 23 Changed from ? to 0.

Procynosuchus delaharpeae Character = 23 RC 92, BP/1/3748.

Galesaurus planiceps Character = 23 BP/1/5064, NMQR 1451, NMQR 860.

Thrinaxodon liorhinus Character = 23 BP/1/4282, BP/1/5208, BP/1/5905.

Platycraniellus elegans Character = 23 Abdala (2007, text-fig. 2D, E).

Cynognathus crateronotus Character = 23 BS 1934 VIII4, PVL 3859.

Diademodon tetragonus Character = 23 MB R1004.

Trirachodon spp. Character = 23 BP/1/4658.

Langbergia modisei Character = 23 The squamosal sulcus in the posterior portion of the zygomatic arch is only insinuated in NMQR 3256 and NMQR 3255. Abdala et al. (2006, pg. 394): The squamosal sulcus is an incipient projection on the lateral surface of the

zygoma and notably more developed on the posterior face of the squamosal (Fig. 3), where the sulcus is directed medially and then ventrally.

Sinognathus gracilis Character = 23 IVPP V2339.

Beishanodon youngi Character = 23 Squamosal sulcus structure is not gomphodont like, but more like in probainognathians.

Scalenodon spp. Character = 23 In the only specimen with preserved zygoma UMZC T.907, There is a lip projected laterally in the dorsal margin of the squamosal on the posterior portion of the zygoma. However there is no evidence of a squamosal sulcus produced between the ventral and dorsal margins of the squamosal zygomatic rama. Therefore the condition does not resemble the squamosal sulcus of other gomphodonts and *Cynognathus crateronotus*. Changed from 1 to 0. Tentative score.

Luangwa spp. Character = 23 BP/1/3731.

Mandagomphodon hirschsoni Character = 23 Hopson (2014: fig.14.3).

Gomphodontosuchus brasiliensis Character = 23 Changed from ? to 1.

Scalenodontoides macrodontes Character = 23 BP/1/5395.

Andescynodon mendozensis Character = 23 Changed from ? to 1.

Protuberum cabralense Character = 23 Changed from ? to 1.

Boreogomphodon jeffersoni Character = 23 Changed from ? to 1.

Traversodon stahleckeri Character = 23 Changed from ? to 1 after von Huene 1936.

Brasilodon tetragonus Character = 23 Changed from ? to 0.

Chaliminia musteloides Character = 23 Changed from ? to 0.

Dadadon isaloi Character = 23 Changed from ? to 1.

Menadon besairiei Character = 23 Changed from ? to 1.

Diarthrognathus broomi Character = 23 Changed from ? to 0.

Nanictosaurus kitchingi Character = 23 Changed from ? to 0.

Cricodon metabolus Character = 23 With deep squamosal sulcus in C. kannemeyeri (SAM-PK-12168). Changed from ? to 1.

Bonacynodon schultzi Character = 23 incipient depression.

Pseudotherium argentinus Character = 23 Changed from ? to 1.

24. Descending flange of squamosal lateral to quadratojugal (This is a ventral projection of the squamosal that cover the quadratojugale and can contact the surangular of the mandible, forming an additional craniomandibular contact.)

0 absent

1 present not contacting surangular

2 present contacting surangular

Procynosuchus delaharpeae Character = 24 RC 92, BP/1/3748.

Galesaurus planiceps Character = 24 AMNH FARB 2223, BP/1/5064.

Thrinaxodon liorhinus Character = 24 Parrington (1946, figures 6 and 10).

Platycraniellus elegans Character = 24 Abdala (2007, text-fig. 5). Postdentary elemnets are displaced of natural position. Therefore the lack of contact with surangular es tentative, because resemblance to the condition in *Thrinaxodon liorhinus*.

Cynognathus crateronotus Character = 24 PVL 3859 (right side). BP/1/1181 (right side).

Diademodon tetragonus Character = 24 BP/1/3639.

Trirachodon spp. Character = 24 SAM-PK-K7888.

Sinognathus gracilis Character = 24 Changed from ? to 2. The lateral projection of the surnagular is practically contacting the squamosal on the right side of IVPP V2339.

Beishanodon youngi Character = 24 The flange is clearly observed in Gao et al (2010, figs. 3B, 4B). The proximity of this surface to the quadrate trochlea suggest that surangular more likely contacted with the flange. tentative score.

Scalenodon spp. Character = 24 Changed from 2 to ? as the relevant area is not preserved.

Luangwa spp. Character = 24 Abdala and Teixeira (2004, pg. 15): In the left cranio-mandibular joint region of MCP3167PV, the squamosal accommodates the quadrate and shows a deep notch for the quadrato-jugal. Close to this notch, the squamosal shows a ventromedial surface for articulation with the posterior extension of the jugal (Fig. 4b). Another small surface on the posterior extremity of the squamosal appears to be for supplementary articulation with the surangular of the lower jaw. Tentative score.

Mandagomphodon hirschsoni Character = 24 relevant area not preserved and not possibe to score. Changed from 2 to ?.

Gomphodontosuchus brasiliensis Character = 24 Changed from 2 to ? Area not preserved in the only known specimen.

Aleodon brachyramphus Character = 24 Changed from 2 to ?.

Ecteninion lunensis Character = 24 Changed from 1 to 1/2. Martinez et al say that it may have contacted the postdentary rod but it is not possible to decide as it is not enough preserved.

Kayentatherium wellesi Character = 24 Changed from ? to 0. Sues (1986) stated that there the surangular lacks a boss for contact with the squamosal. Also, he did not describe a descending process of the squamosal lateral to the quadratojugal (nor is it visible in figure 1). .

Bienotherium spp. Character = 24 This appears to be (0) absent.

Diarthrognathus broomi Character = 24 Changed from ? to 0. There is an squamosal-dentary contact.

Sinoconodon rigneyi Character = 24 Changed from ? to 0. There is an squamosal-dentary contact.

Progalesaurus lootbergensis Character = 24 I am not sure aboutr this.

Pseudotherium argentinus Character = 24 Changed from ? to 1/2. Present, probably did not contact surangular.

25. Temporal fossa (General outline of the temporal fossa (behind the orbit) observed in dorsal view.)

0 widest in the middle

1 same width throughout

2 widest posteriorly

Charassognathus gracilis Character = 25 Changed from ? to 0.

Procynosuchus delaharpeae Character = 25 RC 92, BP/1/3748.

Galesaurus planiceps Character = 25 BP/1/5064, AMNH FARB 2223, NMQR 860.

Thrinaxodon liorhinus Character = 25 Jasinoski et al. 2015: figure 6.

Platycraniellus elegans Character = 25 Abdala (2007, text-figs. 1A, 2A).

Cynognathus crateronotus Character = 25 PVL 3859. BP/1/1181.

Diademodon tetragonus Character = 25 MB R1004.

Trirachodon spp. Character = 25 BP/1/4658.

Langbergia modisei Character = 25 Abdala et al. (2006, pg. 394): The temporal opening shows the same width throughout the entire temporal region.

Sinognathus gracilis Character = 25 Changed from 1 to 0. IVPP V2339.

Beishanodon youngi Character = 25 chenged from 1 to 0 after Gao et al (2010: fig. 3A, B, 4A,B).

Scalenodon spp. Character = 25 assembling the separated portion of the zygoma to the skull in UMZC T.709, it seems that the temporal opening in widest posteriorly (see Abdala and Teixeira, 2004, fig. 8b). tentative score.

Luangwa spp. Character = 25 BP/1/3731. Also some Namibian specimens.

Scalenodontoides macrodontes Character = 25 BP/1/5395.

Andescynodon mendozensis Character = 25 Changed from 1 to 2.

Santacruzodon hopsoni Character = 25 Changed from 1 to ?.

Protuberum cabralense Character = 25 Changed from ? to 1. .

Aleodon brachyramphus Character = 25 Changed from 0 to ?.

Therioherpeton cargnini Character = 25 Changed from 0 to 2.

Boreogomphodon jeffersoni Character = 25 Changed from ? to 0.

Traversodon stahleckeri Character = 25 Changed from ? to 1 after von Huene 1936.

Ecteninion lunensis Character = 25 Changed from 0 to ?. Not preserved.

Morganucodon spp. Character = 25 Changed from 0 to 2.

Cricodon metabolus Character = 25 Changed from ? to 1. Same width throughout in C. kannemeyeri (SAM-PK-12168).

Bonacynodon schultzi Character = 25 Changed from 0 to ?.

26. V shape notch separating lambdoidal crest from zygoma (This structure is observed in occipital view of the skull in the point of encounter of the lambdoidal (occipital) crest with the posterior root of the zygomatic arch.)

0 absent

1 incipient

2 deep

Charassognathus gracilis Character = 26 Changed from ? to 0.

Procynosuchus delaharpeae Character = 26 RC 92, BP/1/3748.

Galesaurus planiceps Character = 26 SAM-PK-K9956, NMQR 135.

Thrinaxodon liorhinus Character = 26 Changed from 0 to 1 the majority of specimens observed (besides of UCMP 40466, BP/1/4263, BP/1/7199) the v shape notch is present. Exception so far were BP/1/5905, NHMUK R3731 without the notch. We score 1 as the condition observed in the vast majotity of specimens.

Platycraniellus elegans Character = 26 Abdala (2007, text-figs. 1C ,2C).

Cynognathus crateronotus Character = 26 BS 1934 VIII4, PVL 3859.

Diademodon tetragonus Character = 26 MB R1004.

Trirachodon spp. Character = 26 GSN R327.

Langbergia modisei Character = 26 NMQR 3256, NMQR 3255. Abdala et al. (2006, pg. 394): In occipital view (Fig. 6), the skull is triangular, showing a well-developed V-shaped notch developed between the lambdoidal crest and the zygomatic arch.

Sinognathus gracilis Character = 26 IVPP V2339.

Beishanodon youngi Character = 26 Considering the pattern of the preserved left zygomatic arch, it is likely that the notch (that is only observable in occipital view) was deep. Tentative score.

Scalenodon spp. Character = 26 The preserved zygoma in UMCZ T.907 is very high and probably produced a deep v shape notch. Tentative score.

Luangwa spp. Character = 26 BP/1/3731.

Mandagomphodon hirschsoni Character = 26 Inferred after Hopson (2014: fig.14.3).

Scalenodontoides macrodontes Character = 26 BP/1/5395.

Protuberum cabralense Character = 26 Changed from 1 to 2.

Boreogomphodon jeffersoni Character = 26 Changed from ? to 2.

Brasilodon tetragonus Character = 26 Changed from ? to 1 (Agustin).

Menadon besairiei Character = 26 Changed from ? to 2.

Ecteninion lunensis Character = 26 This is broken. I am not sure about this scoring.

Diarthrognathus broomi Character = 26 Changed from ? to 1.

Morganucodon spp. Character = 26 Changed from 1 to 0.

Progalesaurus lootbergensis Character = 26 Changed from 0 to 1.

Nanictosaurus kitchingi Character = 26 Changed from ? to 0.

Cricodon metabolus Character = 26 Changed from ? to 1.

Pseudotherium argentinus Character = 26 Changed from ? to 1. It is scored as shallow in Wallace et al 2019 but this state does not correspond to the present matrix and the specimen is not figured in posterior view.

27. Interparietal postparietal in adult (This bone is located posteriorly to the parietal and is visible in dorsal but especially in occipital view as the most dorsal bone of the occiput.)

0 separate bone

1 absent or fused with other bones

Procynosuchus delaharpeae Character = 27 RC 5.

Galesaurus planiceps Character = 27 SAM-PK-K9956, NMQR 135.

Thrinaxodon liorhinus Character = 27 Fourie (1974, figs. 2, 29).

Platycraniellus elegans Character = 27 Abdala (2007, text-figs. 1C ,2C).

Cynognathus crateronotus Character = 27 BSP 1934 VIII4.

Diademodon tetragonus Character = 27 BSP 1934 VIII20.

Trirachodon spp. Character = 27 Not possible to se sutures in the area of this bone. General similitude with the condition in other cynodonts is considered to score the presence. tentative.

Langbergia modisei Character = 27 Abdala et al. (2006, fig. 6).

Sinognathus gracilis Character = 27 Not possible to se sutures in the area of this bone. General similitude with the condition in other cynodonts is considered to score the presence. tentative.

Beishanodon youngi Character = 27 Not described or illustrated in Gao et al. (2010). We change the score from 1 to ?.

Scalenodon spp. Character = 27 Same rationale than in *Sinognathus gracilis*. It is not possible to distintguish the sutre of this element in the material. Similarity in the construction of the occipital plate is considered to score as present.

Luangwa spp. Character = 27 UFRGS 267PV.

Scalenodontoides macrodontes Character = 27 The sutural condition in the occiput of BP/1/5395 is not visible. The construction of the occiput in this species (and in this particular specimen) is very different of other non-mammaliaform cynodonts. Becuase of this we are not very confident of scoring this bone as present in the species.

Andescynodon mendozensis Character = 27 Liu and Powell (2009): In occipital view (fig. 2D), the skull shows a well-developed V-shaped notch between the lambdoidal crest and the zygomatic arch. A round posttemporal fenestra lies in the concave area above the paroccipital process. Other features are obscured due to poor preservation.^n^nConsidering this it is scored as ?.

Boreogomphodon jeffersoni Character = 27 Changed to ? No information on the occiput of this taxon is published.

Traversodon stahleckeri Character = 27 Changed from ? to 0 after von Huene 1936.

Chaliminia musteloides Character = 27 Changed from ? to 1.

Oligokyphus spp. Character = 27 Kuhne (1956): "Interparietal: No sutures are visible between this bone and the tabular and the supraoccipital. As it is unlikely that the latter bone reached the parietal crest and actually covered the posterior and medial surface of

this structure, the presence of the interparietal is indicated."^n^nScored tentatively as absent.

Kayentatherium wellesi Character = 27 Sues (1986): Interparietal and supraoccipital . Inadequate preservation of the available specimens makes tracing of the sutural outlines of both bones impossible.

Tritylodon longaevus Character = 27 In specimen NHMUK R15976 there appears to be a separate interparietal.

Bienotherium spp. Character = 27 Young (1947): Interparietal (?).The summit of the occipital wall is formed by the interparietal. The superior part of the middle of the lambdoidal crest is damaged. No clear suture around the bone could be determined. Whether it is entirely fused with the parietal or still separated as an interparietal is not sure.

Diarthrognathus broomi Character = 27 Wible (1991: pg 4): "No suture is apparent between the parietal and interparietal in the tritheledontid *Diarthrognathus broomi* (Crompton, 1958:197)".

Sinoconodon rigneyi Character = 27 I was unable to check this scoring.

Dvinia prima Character = 27 Changed from ? to 0.

28. Lateral expansion of braincase in parietal region (Luo (1994, character 67) proposed this character with the state 1 (expanded) scored in Mammaliaformes and score 2 (very expanded) only in Triconodontidae. Liu and Olson (2010, character 11) change the wording of the states 1 to moderate and 2 to well developed. They score 1 in tritheledontidos and brasilitherium and state 2 in Mammaliaformes. Therefore they recognize differences in the condition of tritheledontids and Mammaliaformes. Wallace et al 2019 proposed changes to the state of this character after comparing figures of tritylodontids, *Tritheledon riconoi*ts, and *Brasilodon tetragonus*tids, whose braincases appear comparable in width relative to snout width, there is no reason to have character states beyond a narrow braincase (0), and a well-developed, laterally expanded braincase (1), where non-mammaliaforms exhibit the primitive condition, and mammaliaforms exhibit the derived condition. Even as not having a clear cut we decided to follow the

proposal by Liu and Olson (2010) as we recognize differences in the skull of some mammaliamorpha and *Brasilodon tetragonus*tid taxa.)

0 absent

1 moderate

2 well developed

Charassognathus gracilis Character = 28 From photo of the holotype.

Procynosuchus delaharpeae Character = 28 RC 92, BP/1/3748.

Galesaurus planiceps Character = 28 AMNH FARB 2223.

Thrinaxodon liorhinus Character = 28 Estes (1961, plate 1, fig. 1).

Platycraniellus elegans Character = 28 Abdala (2007, text-figs. 1A ,2A).

Cynognathus crateronotus Character = 28 BSP 1934 VIII4.

Diademodon tetragonus Character = 28 MB R1004.

Trirachodon spp. Character = 28 BP/1/4658.

Langbergia modisei Character = 28 NMQR 3255.

Sinognathus gracilis Character = 28 IVPP V2339.

Beishanodon youngi Character = 28 Narrow braincase in dorsal view, even when it is not completely prepared.

Scalenodon spp. Character = 28 UMZC T.907.

Luangwa spp. Character = 28 BP/1/3731.

Scalenodontoides macrodontes Character = 28 BP\ 1 \5395.

Brasilodon tetragonus Character = 28 Changed from 2 to 1.

Cricodon metabolus Character = 28 Changed from ? to 0. It is absent in C. kannemeyeri (SAM-PK-12168).

29. Quadrate notch in squamosal (Squamosal notch is a depression located in the anterior face of the squamosal in the area of the craniomandibular joint that usually will contain the dorsal plate of the quadrate and thus will fix this bone to the skull.)

0 absent

1 present

Procynosuchus delaharpeae Character = 29 Brink (1963, fig. 10E), AMNH 8220.

Platycraniellus elegans Character = 29 Abdala (2007, text-fig. 5).

Beishanodon youngi Character = 29 Gao et al. (2010: figs. 3B,4B).

Mandagomphodon hirschsoni Character = 29 Hopson (2014: fig.14.3).

Gomphodontosuchus brasiliensis Character = 29 Changed from 1 to ? Area not preserved in the only known specimen.

Andescynodon mendozensis Character = 29 Changed from ? to 1.

Protuberum cabralense Character = 29 Changed from ? to 1.

Aleodon brachyramphus Character = 29 Changed from 1 to ?.

Boreogomphodon jeffersoni Character = 29 Changed from ? to 1.

Traversodon stahleckeri Character = 29 Changed from ? to 1 after von Huene 1936.

Brasilodon tetragonus Character = 29 Chnaged from ? to 0.

Dadadon isaloi Character = 29 Changed from ? to 1.

Menadon besairiei Character = 29 Changed from ? to 1. A notch is described to be present for the quadrate.

Oligokyphus spp. Character = 29 I am not sure about this. There is a notch in the squamosal for the quadrate and another for the quadratojugal.

Pachygenelus monus Character = 29 Chnaged from 1 to ?.

Diarthrognathus broomi Character = 29 Changed from 1 to 0. The quadrate and squamosal are fused (no suture is visible).

Morganucodon spp. Character = 29 Changed from 0/1 to 1.

Cricodon metabolus Character = 29 It is decribed as present by Sidor and Hopson, 2018. Changed from ? to 1.

Pseudotherium argentinus Character = 29 Changed from ? to 1.

30. Quadrate, dorsal plate (The dorsal plate is the projection of the quadrate above the trochlea. This projection has an extended surficial contact with the quadrate notch of the squamosal. This is the condition represented in the majority of non-mammaliaform cynodonts and also in therocephalians (Luo and Crompton, 1994). The only projection of the quadrate, dorsal to the trochlea, in *Oligokyphus* spp. is the dorsal angle that we interpret as probable homologous to the dorsal plate, although the articulation is with the paraoccipital process. This angle is located latero-dorsally. In *Kayentatherium wellesi* there is no dorsal angle. Strange enough the quadrate in this tritylodontid does not show any dorsal projection and the articulation with the parotic crest is then basically horizontal. The quadrate in *Morganucodon* spp. is reconstructed by Luo and Crompton (1994) has having a latero-dorsally directed dorsal margin (approximately in the condition of *Oligokyphus* spp.) but this is not what is observed in the quadrate illustrated by Kermack et al. (1981: fig. 86a) where the dorsal margin (termed dorsal laminia of the quadrate) seems to extends mostly dorsally. We follow Luo and Crompton. State 2, absent, is added to score *Kayentatherium wellesi* and Bienotheroides after Luo and Crompton (1994: figs. 9, 10).

0 dorsally projected, contact squamosal

1 latero-dorsally projected, contactng paroccipital process

2 absent

Charassognathus gracilis Character = 30 It is not visible but should be 0.

Procynosuchus delaharpeae Character = 30 Kemp (1979, fig. 9).

Galesaurus planiceps Character = 30 Parrington (1934. 41, describing UMCZ T819): strong upward process. SAM-PK-K1119.

Thrinaxodon liorhinus Character = 30 Luo and Crompton (1994. figure 3).

Platycraniellus elegans Character = 30 Considering the preserved portion of the left quadrtae-quadratojugale in TM 25 (Abdala, 2007, text-fig. 5). the dorsal process is scored as dorsally projected and inserted in the squamosal.

Cynognathus crateronotus Character = 30 SAM-PK-11264.

Diademodon tetragonus Character = 30 BSP 1934 VIII20.

Trirachodon spp. Character = 30 SAM-PK-K5821.

Langbergia modisei Character = 30 Abdala et al. (2006, 394): The dorsal plate of the quadrate is directed dorsally, and the trochlea is cylindrical.

Luangwa spp. Character = 30 MCP 3167PV.

Mandagomphodon hirschsoni Character = 30 Lateral to the medial lappet, the squamosal forms a transverse plate that supports on its anterior face the preserved dorsal ends of the quadrate and quadratojugal.(Hopson, 2014:239).The upper part of the quadrate lies in a sulcus on the anterior face of the squamosal adjacent to the squamosal lappet that abuts the paroccipital process (Hopson, 2014:240). These passages imply the presence of the dorsal plate inserted in the squamosal.

Protuberum cabralense Character = 30 Changed from ? to 0.

Probainognathus jenseni Character = 30 Changed from ? to 0.

Lumkuia fuzzi Character = 30 Changed from ? to 0.

Riograndia guaibensis Character = 30 Changed from ? to 0.

Dadadon isaloi Character = 30 Changed from ? to 0.

Menadon besairiei Character = 30 Changed from ? to 0.

Exaeretodon spp. Character = 30 Changed from ? to 0.

Ecteninion lunensis Character = 30 Changed from ? to 0.

Trucidocynodon riograndensis Character = 30 Changed from ? to 0.

Chiniquodon spp. Character = 30 Changed from ? to 0.

Kayentatherium wellesi Character = 30 Changed from ? to 1.

Tritylodon longaevus Character = 30 This should be scored as 1 but I have not seen any quadrate of *Tritylodon longaevus* to check this.

Bienotherium spp. Character = 30 This should be scored as 1 but I have not seen any quadrate of *Tritylodon longaevus* to check this.

Pachygenelus monus Character = 30 Changed from ? to 0.

Diarthrognathus broomi Character = 30 Changed from ? to 0.

Sinoconodon rigneyi Character = 30 Changed from ? to 0.

Dvinia prima Character = 30 hanged from ? to 0.

Cynosaurus suppostus Character = 30 Changed from ? to 0.

Progalesaurus lootbergensis Character = 30 Unknown, should be 0.

Nanictosaurus kitchingi Character = 30 Inferred.

31. Quadrate, trochlear condyle (Articulation surface of the quadrate with the lower jaw (articular). The condylar sizes are compared only in cylindrical trochleae.)

0 lateral condyle larger

1 medial condyle at least as large as lateral condyle

Procynosuchus delaharpeae Character = 31 Kemp (1979, fig. 9c).

Galesaurus planiceps Character = 31 SAM-PK-K1119.

Thrinaxodon liorhinus Character = 31 Luo and Crompton (1994. figure 17). NHMUK R511, TM 180.

Platycraniellus elegans Character = 31 Abdala (2007, 597): The quadrate bears a cylindrical trochlea, with the medial condyle slightly more developed than the pointed lateral condyle (Text-fig. 5).

Cynognathus crateronotus Character = 31 Changed from 1 to 0. SAM-PK-K11264. NMQR 1444.

Diademodon tetragonus Character = 31 BP/1/3773.

Trirachodon spp. Character = 31 SAM-PK-K 5821.

Langbergia modisei Character = 31 After Liu and Olson (2010) who quote CGP 1/33.

Sinognathus gracilis Character = 31 IVPP V2339.

Beishanodon youngi Character = 31 By observing ventral views in Gao et al. (2010) the condyles appears to be subequals. Scored as ?.

Scalenodon spp. Character = 31 Changed from 0 to ? Not preserved quadrate of *Scalenodon angustifrons*.

Protuberum cabralense Character = 31 Lateral condyle incompletely preserved (Reichel et al., 2009. Text-fig. 9).

Lumkuia fuzzi Character = 31 Changed from 0 to ?. I was unable to check this scoring.

Brasilodon tetragonus Character = 31 Changed from 1 to ?.

Riograndia guaibensis Character = 31 Changed from 1 to 0 after Soares et al 2011.

Dadadon isaloi Character = 31 Changed from 1 to 0 as Ravinohamanana et al 2011 said that the quadrate and quadratojugal are almost identical to those of *Massetognathus* spp..

Ecteninion lunensis Character = 31 I could not check this scoring.

Chiniquodon spp. Character = 31 Changed from ? to 0.

Oligokyphus spp. Character = 31 I am not sure about this scoring. They look very similar to me.

Kayentatherium wellesi Character = 31 I was unable to check this score.

Tritylodon longaevus Character = 31 I was unable to check this scoring.

32. Quadrate, shape of trochlea (Different shapes of the trochlea result in differences in the contact surface between the quadrate and mandible.)

0 cylindrical

1 trough shaped

Procynosuchus delaharpeae Character = 32 AMNH 8220.

Platycraniellus elegans Character = 32 Abdala (2007, 597): The quadrate bears a cylindrical trochlea.

Cynognathus crateronotus Character = 32 SAM-PK-K11264.

Diademodon tetragonus Character = 32 Changed form 0 to 1. BSP 1934 VIII 20. The condition of the quadrate here show a poorly marked ventral projection of the lateral portion of the trochlea, producing an incipient trough (less developed than in *Cynognathus crateronotus*). Watson (1911, 310) mentions: "it is (the articulating surface) marked by a low and obscure ridge towards the outer side". This is interpreted as the incipient trough.

Trirachodon spp. Character = 32 SAM-PK-K 5821.

Langbergia modisei Character = 32 Changed from ? to 0. Abdala et al., (2006, 394). the trochlea is cylindrical without the ventrally projected lateral condyle that externally limits

the trochlear trough observed in *Cynognathus crateronotus* and some traversodontids (Luo & Crompton, 1994. Abdala & Teixeira, 2004).

Sinognathus gracilis Character = 32 IVPP V2339.

Beishanodon youngi Character = 32 Gao et al. (2010: figs. 3B,4B).

Andescynodon mendozensis Character = 32 Changed from ? to 1.

Ecteninion lunensis Character = 32 I could not check this scoring.

Tritylodon longaevus Character = 32 I was unable to check this scoring.

Diarthrognathus broomi Character = 32 Scored as cylindrical following reinterpretation of the bone in the type by Abdala (pers. obs). What Crompton (1958, Fig. 5) interpret as articular is a displaced quadrate, with the anterior margin being the trochlea in articulation with the articular (interpreted as surangular by Crompton (1958). .

33. Lateral notch and neck of quadrate separating lateral margin of contact facet from trochlea (This is a complicated character and there are some serious issues with the scoring of Luo and Crompton (1994, character 8). They score the same state for *Probainognathus jenseni* and *Massetognathus* spp. but the condition are very different because in *Massetognathus* spp. the quadratojugale is fused to the quadrate and then the latter does not have a free margin and a notch visible. A second part of the character appears to have more sense as it is about the presence of a neck with the trochlea located distally. This neck is definitively not observed in basal cynodonts, *Probainognathus jenseni* and *Massetognathus* spp. (following illustrations by Luo and Crompton, 1994, and personal observations) but it is present in tritylodontids, tritheledontids and basal mammaliaformes discussed by Luo and Crompton (1994).)

0 lateral notch absent or poorly developed

1 lateral notch developed separating lateral margin of contact facet from lateral end of trochlea

2 lateral notch broader separation of lateral margin of contact facet from trochlea wider lateral margin shifted medially

3 neck developed displacing contact facet away from trochlea

Procynosuchus delaharpeae Character = 33 The notch as reconstructed by Kemp (1979) and also by Luo and Crompton (1994, fig.2C-E) seems poorly deeveloped and probably because of that was scored as such by Liu and Olson (2010). AMNH 8220, has a nicely prepared quadrate with a complete lateral margin and in the specimen the notch is well developed. Following this we scored as 1 instead because it clearly separates the lateral margin from the trochlea.

Platycraniellus elegans Character = 33 Changed from 1 to ? Not possible to observe in posterior view the presence of a lateral margin directed posteriorly that would limit the lateral notch (as defined by Luo and Crompton 1994 for *Thrinaxodon liorhinus* for example.

Cynognathus crateronotus Character = 33 Changed from 1 to 0. No evidence of notch in the isolated quadrate of SAM-PK-3029.

Diademodon tetragonus Character = 33 Changed from 1 to ?. For scoring this condition in *Diademodon tetragonus* Liu and Olsen (2010, appendix, character 76) quote specimen NHMUK R3587 and Watson (figs. 3 and 7). In fact the quadrate is articulated and the posterior face where is located the notch is not visible (in the material and in Watsons figures).

Langbergia modisei Character = 33 Changed from 1 to ? Not possible to observe in posterior view the presence of a lateral margin directed posteriorly that would limit the lateral notch (as defined by Luo and Crompton 1994 for *Thrinaxodon liorhinus* for example.

Sinognathus gracilis Character = 33 IVPP V2339.

Protuberum cabralense Character = 33 Quadrate and quadratojugal fussed as in *Massetognathus* spp. It is not possible for a notch to be pressent.

Lumkuia fuzzi Character = 33 Changed from 0 to ?. I was unable to check this scoring.

Riograndia guaibensis Character = 33 Changed form 3 to 1/2 (there is notch but not a neck, similar to *Probainognathus jenseni*, Trhinaxodon and *Procynosuchus delaharpeae*. After Soares et al 2011). .

Massetognathus spp. Character = 33 Changed from 2 to ?. The quadrate is fussed to the quadratojugal in *Massetognathus* spp. thus it is imposible to ascertain the presence of a

notch (Contra Luo and Cromtpon 1994). The neck is present according to Luo and Crompton 1994. .

Ecteninion lunensis Character = 33 I could not check this scoring.

Trucidocynodon riograndensis Character = 33 The quadrate and quadratojugal are fused (suture line not visible).

Chiniquodon spp. Character = 33 Chnaged from ? to 2.

Tritylodon longaevus Character = 33 I was unable to check this scoring.

Vetusodon Character = 33 I am not sure about this.

34. Articulation of quadrate with stapes

0 via broad recess on medial margin and medial end of trochlea

1 stapedial contact restricted to medial end of trochlea

2 via projection from medial margin of dorsal plate

3 via medial vertical ridge on neck of quadrate

4 via projection from neck of quadrate (Proposed by Luo and Crompton (1994, character 14), considering contact area or else projection of the quadrate. Both formulation and scoring are based in Luo and Crompton (1994).)

Procynosuchus delaharpeae Character = 34 The score of *Procynosuchus delaharpeae* was based in the quadrate described by Kemp (1979), but the element was not articulated in the skull and is not complete. Changed from 0 to 1 after observation of the quadrate in AMNH FR 8220, where it is clear the medial projection of the trochlea for the contact of the stapes.

Galesaurus planiceps Character = 34 UMCZ T819.

Thrinaxodon liorhinus Character = 34 Luo and Crompton (1994).

Platycraniellus elegans Character = 34 Observed in NMQR 1633, where quadrate and stapes are preserved in natural position.

Cynognathus crateronotus Character = 34 SAM-PK-K11264.

Sinognathus gracilis Character = 34 IVPP V2339.

Beishanodon youngi Character = 34 Considering similitude of the quadrate to that of other cynodonts that have the state 1, we score this state as tentative.

Brasilodon tetragonus Character = 34 Changed from 3 to 4. Stapedial process well developed as in *Morganucodon* spp.

Massetognathus spp. Character = 34 Contra Luo and Cromtpon 1994.

Dadadon isaloi Character = 34 Changed from ? to 1.

Menadon besairiei Character = 34 Changed from ? to 1.

Oligokyphus spp. Character = 34 I am not sure about this scoring.

Tritylodon longaevus Character = 34 I was unable to check this scoring.

35. Squamosal articulation for lower jaw (Wallace et al 2019 (supplementary information, page 2), suggested non independency of this character regarding their character 79 (our character 85) arguing that ?a quadrate/articular jaw joint necessitates the absence of a mandibular articular surface on the squamosal?, which is not always the case as is stated in character 85 where craniomandibular joint can be represented by two articulations. The articulation is considered as present when it is confirmed the contact with the surangular lateral projection (or boss). This is important to mention because in *Thrinaxodon liorhinus* there is a squamosal flange (as defined by Crompton, 1972) but do not contact any element of the mandible.)

0 absent

1 narrow and medially directed

2 wide glenoid cavity ventrally directed

Charassognathus gracilis Character = 35 Changed from ? to 0.

Procynosuchus delaharpeae Character = 35 RC 92, AMNH FR 8220. See Crompton (1972, fig. 3).

Galesaurus planiceps Character = 35 The squamosal flange is present (e.g., AMNH 2223) but there is no contact of mandibular elements with the flange (e.g., BP/1/4602).

Thrinaxodon liorhinus Character = 35 Crompton (1972, fig. 1 and plate 1). Crompton identify an articular flange on the squamosal but this is not contacting the surangular.

Platycraniellus elegans Character = 35 suqamosal develop a descendent process located lateral to the quadratojugale but in that process there is no evidence of articulation with the mandible.

Cynognathus crateronotus Character = 35 BP/1/1181 and SAM-PK 3029.

Diademodon tetragonus Character = 35 Crompton (1972: fig. 5, art fl).

Trirachodon spp. Character = 35 Contact with the surangular (Crompton, 1972: fig. 4, art fl).

Langbergia modisei Character = 35 Abdala et al. (2006, 395): Somewhat anterior to the craniomandibular joint, the surangular shows a slight projection laterally (articular boss), that contacts the descending flange of the squamosal.

Sinognathus gracilis Character = 35 IVPP V2339.

Beishanodon youngi Character = 35 As mentioned before the flange of the suqamosal is well developed ne we assume the contact of this with the surangular as very likely. tentative score.

Scalenodon spp. Character = 35 Changed from 1 to ? No preserved to be sure of the structure and the contact of the mandibular element.

Luangwa spp. Character = 35 Abdala and Taixeira (2004: fig. 4b). The structure is erroneously identified as js (squamosal sutual surface for the jugal), but it more likely the articular flange of the squamosal.

Scalenodontoides macrodontes Character = 35 Changed from 1 to ? No preserved in any specimen.

Protuberum cabralense Character = 35 Preservation is not good enough. There is a ventral projection of the squamosal that probably contacted the surangular. Scored as narrow and medially directed tentatively.

Boreogomphodon jeffersoni Character = 35 Sues and Hopson (2010, see fig. 2): "A well-developed ventral flange of the squamosal extends anteromedially lateral to the slot for the quadratojugal." This is interpreted as the articulation area for the surangular. Tentative score.

Brasilodon tetragonus Character = 35 I am not sure how to score: incipiently developed.

Chaliminia musteloides Character = 35 Area not preserved in known specimens.

Riograndia guaibensis Character = 35 ?.

Massetognathus spp. Character = 35 See Crompton 1972.

Dadadon isaloi Character = 35 A narrow and medially directed articulation appears to be present but this is not described.

Pachygenelus monus Character = 35 Based in interpretation by Hopson and Barghusen (1986), fig. 14. Wible and Hopson (1993) fig. 5.2. But see Luo (1994, fig. 6.7A).

Diarthrognathus broomi Character = 35 After Crompton (1958, fig. 5).

Sinoconodon rigneyi Character = 35 Luo 1994, fig. 6.7B.

Morganucodon spp. Character = 35 Luo (1994, fig. 6.7D). Wible and Hopson (1993. fig. 5.2B).

Cricodon metabolus Character = 35 Changed from 0 to ?. Sidor and Hopson (2017) scored this in *Cricodon metabolus* but they recognize a new species *Cricodon kannemeyeri* whose taxonomic assignment is disputed (see Hendrickx et al., 2019). By now we consider that known specimen of *Cricodon metabolus* does not have a complete or good preservation of the area to allow scoring.

36. Hyoid muscle fossa in paroccipital process (The fossa for origin of the levator hyoidei or its derivative, the stapedius muscle (Wible, 1991) when present in non-mammalian cynodonts is located between the anterior (quadrate) and posterior (mastoid) processes of the paroccipital process (prootic portion of the petrosal). This structure is only recognized in tritylodontids and mammaliaformes and as a shallow depression in *Exaeretodon riograndensis* (Abdala et al., 2002, 319). Liu and Olsen (2010) dismissed the condition identified by Abdala et al (2002) in *Exaeretodon*, or at least scored as absent in *Exaeretodon argentinus*.)

0 absent

1 present and incipient

2 present and well developed

Procynosuchus delaharpeae Character = 36 RC 92, BP/1/3748.

Galesaurus planiceps Character = 36 SAM-PK-K9956.

Thrinaxodon liorhinus Character = 36 BP/1/7199.

Platycraniellus elegans Character = 36 Abdala (2007, text-figs. 2B, 4).

Cynognathus crateronotus Character = 36 BP/1/1181.

Diademodon tetragonus Character = 36 MB R1004.

Trirachodon spp. Character = 36 BP/1/4658.

Langbergia modisei Character = 36 NMQR 3255.

Sinognathus gracilis Character = 36 IVPP V2339.

Scalenodon spp. Character = 36 UMZC T907, Parrington (1946, fig. 9A).

Luangwa spp. Character = 36 NHMUK PV R 36995.

Mandagomphodon hirschsoni Character = 36 Hopson (2014: fig. 14.3).

Scalenodontoides macrodontes Character = 36 BP/1/5395.

Exaeretodon spp. Character = 36 Changed from 0 to 1 after Abdala et al 2002.

Trucidocynodon riograndensis Character = 36 Unable to check.

Tritylodon longaevus Character = 36 I was unable to check this scoring.

Dvinia prima Character = 36 Changed from ? to 0. A superficial fossa on the ventral face of the paroccpital process is identified as hyoid fossa (?) by Tatarinov (1968. see figure 4 and p. 43). ^nThis interpretation was downplayed by Hopson, who said that these structure were present on one of the side and not in the other and that were better interpreted as ?artefacts resulting from the clearing of the matrix from spaces within incompletely ossified paroccipital process.^nAbsent (0) is then scored for this character. June 2009, FA agrees with Hopsons interpretation.

Cynosaurus suppostus Character = 36 I was unable to check this.

Nanictosaurus kitchingi Character = 36 Changed from ? to 0.

37. Paroccipital process (Abdala (2007, character 44) proposed three states characters undifferentiated, differentiated in quadrate and mastoid process (state 1, scored in therocephalians only) and differentiated in anterior and posterior process (scored in tritylodontids, *Brasilodon tetragonus* and mammaliaforms). Something to discuss is if the

presence of the two separate processes in therocephalians and mammaliamorphs are really two different events or it is just one. In any case, we do not have therocephalians in this matrix and then only two states were scored. Abdala (2007) scored mistakenly *Morganucodon* spp. as 1 instead of 2. Wallace et al. (2019, supplementary information, page 2) discussed the formulation of this character and discarded one stage. We agree that the differentiation of the process in non-mammalian cynodonts should represent only one state.)

0 undifferentiated

1 differentiated into mastoid and quadrate processes

2 differentiated into anterior and posterior processes

Procynosuchus delaharpeae Character = 37 RC 92, BP/1/3748, Kemp (1979, fig. 2).

Galesaurus planiceps Character = 37 SAM-PK-K9956.

Thrinaxodon liorhinus Character = 37 BP/1/7199.

Platycraniellus elegans Character = 37 Abdala (2007, text-figs. 2B, 4).

Cynognathus crateronotus Character = 37 BP/1/1181.

Diademodon tetragonus Character = 37 MB R1004.

Trirachodon spp. Character = 37 SAM-PK-K5821.

Langbergia modisei Character = 37 SAM-PK-11481.

Sinognathus gracilis Character = 37 IVPP V2339.

Beishanodon youngi Character = 37 The partly exposed left paroccipital process seems to be undifferentiated. tentative score.

Scalenodon spp. Character = 37 UMZC T907, Parrington (1946, fig. 9A).

Luangwa spp. Character = 37 NHMUK PV R 36995.

Mandagomphodon hirschsoni Character = 37 Hopson (2014: fig. 14.3).

Scalenodontoides macrodontes Character = 37 BP/1/5395.

Andescynodon mendozensis Character = 37 Changed from ? to 0.

Protuberum cabralense Character = 37 Changed from ? to 0.

Boreogomphodon jeffersoni Character = 37 Changed from ? to 0.

Brasilodon tetragonus Character = 37 Wallace et al 2019 proposed changing this from undiferentiated (0) to differentiated (1/2 - it should be 2) based on Rodrigues et al 2014.

Dadadon isaloi Character = 37 Changed from ? to 0.

Menadon besairiei Character = 37 Changed from ? to 0.

Diarthrognathus broomi Character = 37 Changed from ? to 2.

Sinoconodon rigneyi Character = 37 Luo (1994) Fig. 6.7B.

Morganucodon spp. Character = 37 Scored after Luo (1994, fig. 6.3B) but see Wible and Hopson (1993, figs. 5.2. 5.3).

Irajatherium hernandezi Character = 37 after Oliveira et al. (2010, fig. 4D).

38. Paroccipital process in base of posttemporal fossa

0 present

1 absent

Procynosuchus delaharpeae Character = 38 The condition illustrated is absent (Kemp, 1979, fig. 3). Brink (1963, fig. 10E), and that seems to be the case in NHMUK PV37054, RC 5 and RC 12, but it is present in RC 87.

Galesaurus planiceps Character = 38 SAM-PK-K9956, NMQR 135 (Jasinoski and Abdala, fig. 12).

Thrinaxodon liorhinus Character = 38 TM 80, BP/1/7199.

Platycraniellus elegans Character = 38 Abdala (2007: 598): ..in NMQR 1633. In the latter specimen, the suture between the right exoccipital and paroccipital process is visible, whereas the small post-temporal canal is completely encircled by the tabular.

Cynognathus crateronotus Character = 38 SAM-PK-K 11264.

Diademodon tetragonus Character = 38 BP/1/3754.

Trirachodon spp. Character = 38 SAM-PK-K5821.

Langbergia modisei Character = 38 Abdala et al. (2006, 394): The circular post-temporal foramen is completely encircled by the tabular.

Sinognathus gracilis Character = 38 IVPP V2339.

Scalenodontoides macrodontes Character = 38 BP/1/5395. Tentative score.

Protuberum cabralense Character = 38 Changed from ? to 1.

Riograndia guaibensis Character = 38 Changed from 1 to 0 (Soares et al 2011).

Menadon besairiei Character = 38 Changed from ? to 0.

Exaeretodon spp. Character = 38 I am not sure of this scoring.

Adelobasileus cromptoni Character = 38 The post-temporal fossa (post-temporal canal of Lucas and Luo 1993) appears to be completely included in the petrosal. However, the proccipital procces does not seem to border the fossa. So I would change this scoring from 0 to 1.

Diarthrognathus broomi Character = 38 Changed from ? to 0.

Sinoconodon rigneyi Character = 38 I was unable to check this scoring.

Cynosaurus suppostus Character = 38 Changed from ? to 0.

Pseudotherium argentinus Character = 38 Changed from ? to 1.

39. Postemporal fossa in relation to foramen magnum (Postemporal fossa particularly large are uncommon and can be ascribed to *Dvinia prima* and *Cynognathus crateronotus*.)

0 of same size or slightly smaller

1 notably smaller

Procynosuchus delaharpeae Character = 39 RC 12, Kemp (1979, fig. 3b).

Galesaurus planiceps Character = 39 SAM-PK-K9956. Jasinoski and Abdala (2017).

Thrinaxodon liorhinus Character = 39 TM 180. Fourie (1974).

Platycraniellus elegans Character = 39 Abdala (2007. text-fig. 2C).

Cynognathus crateronotus Character = 39 SAM-PK-3029. PVL 3859.

Diademodon tetragonus Character = 39 MB R1004, BSP 1934 VIII19.

Langbergia modisei Character = 39 NMQR 3256.

Sinognathus gracilis Character = 39 IVPP V2339.

Scalenodon spp. Character = 39 UMZC T918.

Luangwa spp. Character = 39 NHMUK PV R 36995.

Scalenodontoides macrodontes Character = 39 BP/1/5395.

Protuberum cabralense Character = 39 Changed from ? to 1.

Riograndia guaibensis Character = 39 Changed from ? to 1 (Agustin).

Dadadon isaloi Character = 39 Changed from ? to 1.

Menadon besairiei Character = 39 Changed from ? to 1.

Adelobasileus cromptoni Character = 39 Changed from ? to 1.

Kayentatherium wellesi Character = 39 Changed from ? to 1.

Tritylodon longaevus Character = 39 Changed from 0 to 1.

Bienotherium spp. Character = 39 Changed from ? to 1.

Diarthrognathus broomi Character = 39 Changed from ? to 1.

Pseudotherium argentinus Character = 39 Changed from ? to 1.

Abdalodon muchingaensis Character = 39 The postemporal fossa is more than half the diameter of the foramen magnum.

40. Tabular (Usually large bone located at the side of the occiput.)

0 present

1 absent

Procynosuchus delaharpeae Character = 40 RC 5, RC 87.

Galesaurus planiceps Character = 40 SAM-PK-K9956.

Thrinaxodon liorhinus Character = 40 TM 180. Fourie (1974).

Platycraniellus elegans Character = 40 Abdala (2007. text-fig. 2C), also observed in NMQR 1633.

Cynognathus crateronotus Character = 40 BSP 1934 VIII4.

Diademodon tetragonus Character = 40 BP/1/3639, BP/1/3754.

Trirachodon spp. Character = 40 SAM-PK-K 5821.

Langbergia modisei Character = 40 NMQR 3255.

Sinognathus gracilis Character = 40 IVPP V2339.

Scalenodon spp. Character = 40 UMZC T918.

Luangwa spp. Character = 40 UFRGS 267PV.

Mandagomphodon hirschsoni Character = 40 These depressions contain matrix, but the lateral one is undoubtedly floored by the tabular and is penetrated by the small posttemporal foramen.(Hopson, 2014: 240).

Aleodon brachyramphus Character = 40 Changed from 0 to ?.

Sinoconodon rigneyi Character = 40 I was unable to check this scoring.

Morganucodon spp. Character = 40 Changed from 1 to ? after Wible (1991:5). "because of uncertainty, ? is scored for morganucodontidae here.".

41. Incisive foramen (Located on the anterior margin of the palate, behind the central incisors.)

0 absent

1 not closed

2 posteriorly closed by maxilla

3 completely enclosed by premaxilla

Procynosuchus delaharpeae Character = 41 RC 5,RC 87.

Galesaurus planiceps Character = 41 NMQR 135.

Thrinaxodon liorhinus Character = 41 NHMUK R511.

Platycraniellus elegans Character = 41 Abdala (2007, text-fig. 2B).

Cynognathus crateronotus Character = 41 BSP 1934 VIII6.

Diademodon tetragonus Character = 41 BP/1/2522.

Trirachodon spp. Character = 41 BP/1/4658.

Langbergia modisei Character = 41 NMQR 3251.

Beishanodon youngi Character = 41 Gao et al. (2010: 12): The incisive foramen is mostly bordered by the premaxillae, but is closed posteriorly by the maxillae.

Luangwa spp. Character = 41 Abdala and Smith (2009): Fig 2A. Tentative score as the suture between premaxilla and maxilla in the specimen are not secure.

Mandagomphodon hirschsoni Character = 41 The palatal suture between premaxilla and maxilla appears to extend posterointernally from the lingual side of the third incisor to the posterior part of the incisive foramen. As noted above, it is unclear whether the premaxilla or the maxilla forms the posterior border of the foramen (Hopson (2014: 238 and fig. 14.1). Score changed from 2 to ?.

Mandagomphodon attridgei Character = 41 NHMUK R8578.

Gomphodontosuchus brasiliensis Character = 41 Changed from 2 to ? Sutures between premaxilla and maxilla wre only interpreted (see Hopson,1985, fig. 3). The anterior extension of the maxilla seems to be very close to the posterior margin of the foramen.

Pascualgnathus polanski Character = 41 Changed from ? to 2.

Aleodon cromptoni Character = 41 Tentative score after Martinelli et al. (2016, figure 15 and 16).

Probainognathus jenseni Character = 41 Changed from 2 to 3.

Brasilodon tetragonus Character = 41 Changed from ? to 2.

Riograndia guaibensis Character = 41 Chnaged from 3 to 2 (Agustin).

Massetognathus spp. Character = 41 There is some participation of the vomer according to Crompton et al 2017 (fig. 7).

Menadon besairiei Character = 41 Changed from 2 to ?. It is not possible to decide if the maxilla participates or not.

Sinoconodon rigneyi Character = 41 Changed from ? to 2.

Morganucodon spp. Character = 41 Changed from ? to 2.

Cricodon metabolus Character = 41 Changed from 2 to ? - I could not check this scoring.

Prozostrodon brasiliensis Character = 41 Changed from ? to 3.

42. Paracanine fossa in relation to the upper canine (Fossa on the palate where occlude the lower canine.)

0 anterior

1 anteromedial

2 medial

3 posteromedial

4 paracanine fossa absent

Procynosuchus delaharpeae Character = 42 RC 5, Kemp (1979, fig. 2).

Galesaurus planiceps Character = 42 NMQR 135.

Thrinaxodon liorhinus Character = 42 NHMUK R511.

Platycraniellus elegans Character = 42 Abdala (2007, text-fig. 2B).

Cynognathus crateronotus Character = 42 BSP 1934 VIII6.

Diademodon tetragonus Character = 42 BP/1/2522.

Trirachodon spp. Character = 42 BP/1/4658.

Langbergia modisei Character = 42 NMQR 3251.

Sinognathus gracilis Character = 42 IVPP V2339.

Beishanodon youngi Character = 42 Gao et al. (2010: 12): Anteromedial to the enlarged canine, a deep depression, the paracanine fossa.

Scalenodon spp. Character = 42 UMZC T907.

Luangwa spp. Character = 42 UFRGS 267PV.

Mandagomphodon hirschsoni Character = 42 Hopson (2014:fig. 14.1).

Mandagomphodon attridgei Character = 42 NHMUK R8578.

Gomphodontosuchus brasiliensis Character = 42 Hopson (1984, fig. 3).

Scalenodontoides macrodontes Character = 42 BP/1/5395.

Santacruzodon hopsoni Character = 42 Changed from 1 to ?.

Boreogomphodon jeffersoni Character = 42 Changed from 0 to 1.

Lumkuia fuzzi Character = 42 Changed from 1 to ?. I was unable to check this scoring.

Brasilodon tetragonus Character = 42 Changed from ? to 1.

Oligokyphus spp. Character = 42 Changed from - to 4.

Kayentatherium wellesi Character = 42 Changed from - to 4.

Tritylodon longaevus Character = 42 Changed from - to 4.

Bienotherium spp. Character = 42 Changed from - to 4.

Nanictosaurus kitchingi Character = 42 Changed from ? to 1. Inferred from position of ocluded lower canine in specimens.

Cricodon metabolus Character = 42 Sidor and Hopson (2017, fig. 2C).

Protheriodon estudianti Character = 42 Changed from ? to 1. It is interpreted by Bonaparte et al 2016 to have been anteromedial.

43. Vomer internarial shape (This character was introduced by Hopson and Kitching (2001, character 8). As scored by them all taxa without closed secondary palate show a broad plate and remaining cynodonts with close palate have parallel-sided keel. The character was not used in successive cladistic analyses, besides of Sidor and Hopson (2018, character 6) with the same scoring as Hopson and Kitching (2001). The wording is not very clear to us. It seems that the broad plate of the vomer is only represented when the secondary palate is not completely closed. When the secondary palate is closed the vomer is covered and shows a narrow structure (see vomer illustrations in Fourie, 1974 serial sections of *Thrinaxodon liorhinus*). Then we will follow the rationale of Hopson and Kitching (2001) but it should be considered the fact that an internal view above the osseous palate is necessary to be absolutely certain of the scoring of this character in forms with complete secondary palate.

0 broad plate

1 with a narrow keel

Procynosuchus delaharpeae Character = 43 RC 5.

Galesaurus planiceps Character = 43 NMQR 135.

Thrinaxodon liorhinus Character = 43 Closed palate score then as 1.

Platycraniellus elegans Character = 43 Closed palate score then as 1.

Cynognathus crateronotus Character = 43 Closed palate score then as 1.

Diademodon tetragonus Character = 43 Closed palate score then as 1.

Trirachodon spp. Character = 43 Closed palate score then as 1.

Langbergia modisei Character = 43 Closed palate score then as 1.

Sinognathus gracilis Character = 43 Closed palate score then as 1.

Beishanodon youngi Character = 43 Closed palate score then as 1.

Scalenodon spp. Character = 43 Closed palate score then as 1.

Luangwa spp. Character = 43 Closed palate score then as 1.

Mandagomphodon hirschsoni Character = 43 Closed palate score then as 1.

Mandagomphodon attridgei Character = 43 Closed palate score then as 1.

Gomphodontosuchus brasiliensis Character = 43 Closed palate score then as 1.

Scalenodontoides macrodontes Character = 43 Closed palate score then as 1.

Aleodon brachyramphus Character = 43 Changed from 1 to ?.

Aleodon cromptoni Character = 43 Chnaged from 1 to ?.

Probainognathus jenseni Character = 43 Chnaged from 1 to 0 after Crompton et al 2017.

Lumkuia fuzzi Character = 43 Changed from 1 to ?. I was unable to check this scoring.

Traversodon stahleckeri Character = 43 Changed from 1 to ?.

Riograndia guaibensis Character = 43 Changed from 1 to ?.

Massetognathus spp. Character = 43 Changed from 1 to 0 after Crompton et al 2017.

Exaeretodon spp. Character = 43 I was unable to check this.

Ecteninion lunensis Character = 43 I was unable to check this.

Trucidocynodon riograndensis Character = 43 I was unable to check this.

Chiniquodon spp. Character = 43 I was unable to check this.

Elliotherium kersteni Character = 43 I was unable to check this.

Pachygenelus monus Character = 43 I was unable to check this.

Dvinia prima Character = 43 I was unable to check this.

Cynosaurus suppostus Character = 43 I was unable to check this.

Progalesaurus lootbergensis Character = 43 Changed from 0 to ?.

Pseudotherium argentinus Character = 43 Changed from ? to 0.

Diegocanis elegans Character = 43 I was unable to check this.

44. Vomer exposure in incisive foramen at anterior ends of maxillae on palate

0 present

1 absent

Procynosuchus delaharpeae Character = 44 RC 5.

Galesaurus planiceps Character = 44 NMQR 135.

Thrinaxodon liorhinus Character = 44 NHMUK R511.

Platycraniellus elegans Character = 44 TM 25.

Cynognathus crateronotus Character = 44 BSP 1934 VIII6.

Diademodon tetragonus Character = 44 BP/1/2522.

Trirachodon spp. Character = 44 BP/1/4658.

Langbergia modisei Character = 44 NMQT 3251.

Beishanodon youngi Character = 44 Gao et al. (2010: 13): The anterior extremity of the median vomer is narrowly exposed within the incisive foramen..

Mandagomphodon hirschsoni Character = 44 The area of interest is poorly preserved. Not possible to score. Changed from 1 to ?.

Mandagomphodon attridgei Character = 44 The premaxillae send a medial projection posteriorly that form the central bony section of the incisive foramen.

Gomphodontosuchus brasiliensis Character = 44 GPIT/RE/09397. Tentative score as the area is poorly preserved.

Aleodon brachyramphus Character = 44 Changed from ? to 1.

Aleodon cromptoni Character = 44 Changed from ? to 1.

Traversodon stahleckeri Character = 44 Changed from ? to 0 after Barberena 1981.

Brasilodon tetragonus Character = 44 Changed from ? to 0.

Massetognathus spp. Character = 44 Crompton et al 2017 (fig. 7).

Elliotherium kersteni Character = 44 Inferred to be absent but it is unknown because the premaxilla is missing. Changed from 1 to ?.

Nanictosaurus kitchingi Character = 44 Chnaged from 1 to ?.

Cricodon metabolus Character = 44 Probably absent as the secondary palate is complete.

45. Osseous secondary palate

0 complete with contribution of palatine

1 maxillo palatine extensions do not contact medially

2 absent

3 complete without contribution of palatine

Procynosuchus delaharpeae Character = 45 RC 5, NHMUK PV37054 .

Galesaurus planiceps Character = 45 NMQR 135.

Thrinaxodon liorhinus Character = 45 NHMUK R511.

Platycraniellus elegans Character = 45 Abdala (2007, text-fig. 2B).

Cynognathus crateronotus Character = 45 BSP 1934 VIII6.

Diademodon tetragonus Character = 45 BP/1/2522.

Trirachodon spp. Character = 45 BP/1/4658.

Langbergia modisei Character = 45 NMQT 3251.

Sinognathus gracilis Character = 45 Changed from ? to 0.

Beishanodon youngi Character = 45 Gao et al. (2010, figs. 3B, 4B).

Scalenodon spp. Character = 45 UMZC T907.

Luangwa spp. Character = 45 UFRGS 267PV.

Mandagomphodon hirschsoni Character = 45 Hopson (2014: fig. 14.1b).

Mandagomphodon attridgei Character = 45 Changed from ? to 0.

Gomphodontosuchus brasiliensis Character = 45 GPIT/RE/09397.

Scalenodontoides macrodontes Character = 45 BP/1/5395.

Santacruzodon hopsoni Character = 45 Changed from ? to 0.

Boreogomphodon jeffersoni Character = 45 Changed from ? to 0.

Chaliminia musteloides Character = 45 Changed from ? to 0.

Dadadon isaloi Character = 45 Changed from ? to 0.

Menadon besairiei Character = 45 Changed from ? to 0.

Dvinia prima Character = 45 Abdala: Maxillo-palatines extensions do not contact medially (1). After observation of the cast of the holotype in which the palatal plates do not seems to be in contact. This is also scored in a similar way by Hopson and Kitching (2001) and Sidor and Smith (2004). In the holotype of *Permocynodon* (the complete skull) the maxilla plates are forming a closed osseous palate but it seems quite evident that the plate of the left side has been displaced from its original placement and thus the close palate is therefore an artifact.

Bonacynodon schultzi Character = 45 Changed from ? to 0.

Protheriodon estudianti Character = 45 Changed from ? to 20.

Prozostrodon brasiliensis Character = 45 Changed from ? to 0.

Irajatherium hernandezi Character = 45 complete but the palatine is unknown.

46. Osseous palate extension

0 45 per cent of skull length or less

1 more than 45 per cent of skull length

Procynosuchus delaharpeae Character = 46 In RC 5 is 30% in RC 12 is 37%. Palate length is measured from the tip of the snout to where the medial platform of the palatine are closest, near the central portion of the palate.

Galesaurus planiceps Character = 46 NMQR 135.

Thrinaxodon liorhinus Character = 46 NHMUK R511.

Platycraniellus elegans Character = 46 33% (Abdala, 2007: table 1).

Cynognathus crateronotus Character = 46 BSP 1934 VIII6.

Diademodon tetragonus Character = 46 BP/1/2522.

Trirachodon spp. Character = 46 BP/1/4658.

Langbergia modisei Character = 46 NMQR 3255.

Sinognathus gracilis Character = 46 IVPP V2339.

Beishanodon youngi Character = 46 39%. Palate length: 8.7. Basal skull length: 22.3 (estimated after figure 4B of Gao et al. 2010, using Digimizer).

Luangwa spp. Character = 46 BP/1/3731.

Gomphodontosuchus brasiliensis Character = 46 Changed from 0 to ? The type and only specimen is incompletely preserved.

Scalenodontoides macrodontes Character = 46 BP/1/5395. Tentative score.

Protuberum cabralense Character = 46 Changed from ? to 1.

Aleodon brachyramphus Character = 46 Changed from 0 to ?.

Brasilodon tetragonus Character = 46 Changed from 1 to 0 after Bonaparte et al 2013.

Chaliminia musteloides Character = 46 Changed from 0 to 1.

Massetognathus spp. Character = 46 See for example PVL3906.

Dadadon isaloi Character = 46 Changed from ? to 0.

Menadon besairiei Character = 46 Changed from ? to 0.

Exaeretodon spp. Character = 46 Scored after MACN 18125 and MCP 1522 PV.

Elliotherium kersteni Character = 46 Inferred.

47. Length secondary palate relative to anterior border of orbit

    0 shorter

    1 about equal

    2 longer

Procynosuchus delaharpeae Character = 47 RC 5, RC 12. For this is considered the posterior extnesion of the palatine plate, near the center of the skull, where they are the closest.

Galesaurus planiceps Character = 47 NMQR 135.

Thrinaxodon liorhinus Character = 47 NHMUK R511.

Platycraniellus elegans Character = 47 Palate length 2.8 cmm. snout length: 3.3 cm.

Cynognathus crateronotus Character = 47 BSP 1934 VIII6.

Diademodon tetragonus Character = 47 BP/1/2522.

Trirachodon spp. Character = 47 BP/1/4658.

Langbergia modisei Character = 47 NMQR 3255.

Sinognathus gracilis Character = 47 IVPP V2339.

Beishanodon youngi Character = 47 Gao et al (2010: fig. 4A, B). Comparing these figures the posterior extension of the palate seems to extends beyond the anterior margin of the orbit.

Scalenodon spp. Character = 47 UMZC T907.

Luangwa spp. Character = 47 NHMUK PV R 36995, BP/1/3731.

Mandagomphodon hirschsoni Character = 47 Hopson (2014: fig. 14.1).

Mandagomphodon attridgei Character = 47 NHMUK R8578.

Gomphodontosuchus brasiliensis Character = 47 GPIT/RE/09397.

Scalenodontoides macrodontes Character = 47 BP/1/5395.

Andescynodon mendozensis Character = 47 Changed from ? to 0.

Protuberum cabralense Character = 47 Changed from ? to 1.

Boreogomphodon jeffersoni Character = 47 Changed from ? to 2.

Brasilodon tetragonus Character = 47 Changed from 2 to 1.

Massetognathus spp. Character = 47 See for example PVL3906.

Dadadon isaloi Character = 47 Changed from ? to 2.

Menadon besairiei Character = 47 Changed from ? to 0.

Diarthrognathus broomi Character = 47 Changed from 2 to ?.

Nanictosaurus kitchingi Character = 47 Changed from ? to 0.

Cricodon metabolus Character = 47 Changed from ? to 0 (the upper postcanines reach the anterior border of the orbit and the palate does not reach the last postcanines).

Bonacynodon schultzi Character = 47 Changed from 1 to 0.

48. Osseous palate posterior extent in relation to upper tooth row

0 anterior

1 at same level or posterior

Procynosuchus delaharpeae Character = 48 RC 5, RC 12.

Galesaurus planiceps Character = 48 NMQR 135.

Thrinaxodon liorhinus Character = 48 NHMUK R511.

Platycraniellus elegans Character = 48 Abdala, 2007: 596): The extent of the palate is almost the same as the snout length and reaches the penultimate postcanine. .

Cynognathus crateronotus Character = 48 BSP 1934 VIII6.

Diademodon tetragonus Character = 48 BP/1/2522.

Trirachodon spp. Character = 48 BP/1/4658.

Langbergia modisei Character = 48 NMQR 3255.

Sinognathus gracilis Character = 48 IVPP V2339.

Beishanodon youngi Character = 48 Gao et al (2010: fig. 4B).

Scalenodon spp. Character = 48 UMZC T907.

Luangwa spp. Character = 48 BP/1/3731.

Mandagomphodon hirschsoni Character = 48 Hopson (2014: fig. 14.1b).

Mandagomphodon attridgei Character = 48 At level of the fifth (out of eight) postcanine.

Gomphodontosuchus brasiliensis Character = 48 GPIT/RE/09397.

Scalenodontoides macrodontes Character = 48 BP/1/5395.

Santacruzodon hopsoni Character = 48 Melo (2020, PhD).

Protuberum cabralense Character = 48 Changed from ? to 0.

Therioherpeton cargnini Character = 48 Changed from 1 to 0.

Boreogomphodon jeffersoni Character = 48 Changed from 1 to 0.

Arctotraversodon plemmyridon Character = 48 Changed from 1 to ?.

Traversodon stahleckeri Character = 48 Changed from ? to 0 after Barberena 1981.

Dadadon isaloi Character = 48 Changed from 1 to 0.

Menadon besairiei Character = 48 Changed from ? to 0.

Oligokyphus spp. Character = 48 Changed from ? to 0.

Diarthrognathus broomi Character = 48 Changed from 1 to ?.

Bonacynodon schultzi Character = 48 Chnaged from 1 to 0.

49. Palatine

0 does not meet frontal

1 meets frontal but neither element contributes significantly to medial orbit wall

2 meets frontal and both elements contribute significantly to medial orbit wall

Procynosuchus delaharpeae Character = 49 NHMUK PV37054.

Galesaurus planiceps Character = 49 NMQR 135.

Thrinaxodon liorhinus Character = 49 TM 80b.

Platycraniellus elegans Character = 49 Abdala, 2007: 596): The frontal exhibits a long ventral process on the medial border of the orbit, but does not appear to make contact with the palatine.

Cynognathus crateronotus Character = 49 BSP 1934 VIII6.

Diademodon tetragonus Character = 49 1934 VIII19.

Trirachodon spp. Character = 49 SAM-PK-K4801.

Langbergia modisei Character = 49 NMQR 3251, 3255.

Scalenodon spp. Character = 49 Changed from 0 to ?.

Luangwa spp. Character = 49 NHMUK PV R 36995.

Mandagomphodon hirschsoni Character = 49 Relevant area not preserved. Changed from 1 to ?.

Lumkuia fuzzi Character = 49 Changed from 0 to 1.

Chaliminia musteloides Character = 49 Changed from ? to 2 as the frontal participates from the orbital margin.

Massetognathus spp. Character = 49 I am not sure of this scoring.

Dadadon isaloi Character = 49 This should be 0 or 1 (they do not contribute to the orbital wall). .

Menadon besairiei Character = 49 This should be 0 or 1 (they do not contribute to the orbital wall). .

Oligokyphus spp. Character = 49 Changed from ? to 0. It apparently does not meet the frontal (according to the frontal description), but the palatine is only little preserved.

Diarthrognathus broomi Character = 49 Changed from 1 to 2.

Sinoconodon rigneyi Character = 49 With doubts (see Crompton and Luo 1993, fig. 4.9).

Cynosaurus suppostus Character = 49 Changed from ? to 0.

Tessellatia bonapartei Character = 49 Para mi es claro que no hay contacto entre el frontal y el palatino (0) pero es cierto que el frontal contribuye a la pared interna de la órbita. Frente a las dudas de Abdala lo codifico como ?.

50. Sphenopalatine foramen (This foramen is rarely described in non-mammaliaform cynodonts. Original statement by Luo (1994) is separate opening for the lesser palatine nerve in the orbit, absent (0), present (1).There is an illustration of *Kayentatherium wellesi* by Luo (1994, fig. 6.2B) in which it is represented as a big foramen and another tiny, bearably visible, identified as greater and lesser palatine foramina; whereas Luo (1994, fig. 6.2C) identify two separated small foramina in *Sinoconodon rigneyi*. Martinez et al (1996) used the character and score as Luo (1994), but they scored the presence of the opening also in tritheledontids (scored as absent by Luo). Liu and Olsen (2010) changed the statement of the character to presence/absence of the foramen.)

0 absent

1 present *Procynosuchus delaharpeae* Character = 50 NHMUK PV37054 .

Platycraniellus elegans Character = 50 See comments in character statement. Not observed in TM 25.

Protuberum cabralense Character = 50 Changed from 1 to ?.

Exaeretodon spp. Character = 50 Changed from 1 to 0. This foramen is not illustrated by Bonaparte and I could not confirm its presence.

Trucidocynodon riograndensis Character = 50 Unable to check. The sphenopalatine foramen is not figured or described.

Oligokyphus spp. Character = 50 Changed from ? to 0.

Tritylodon longaevus Character = 50 I was unable to check this scoring. They appear to be absent.

Bienotherium spp. Character = 50 I was unable to check this scoring.

Diarthrognathus broomi Character = 50 I was unable to check this scoring.

Dvinia prima Character = 50 Changed from 1 to ?.

51. Palatine

0 excluded from subtemporal border of orbit

1 participates in subtemporal border by displacing pterygoid posteriorly

Procynosuchus delaharpeae Character = 51 NHMUK PV37054 .

Galesaurus planiceps Character = 51 NMQR 135.

Thrinaxodon liorhinus Character = 51 NHMUK R511.

Platycraniellus elegans Character = 51 Abdala, 2007. text-fig. 2B).

Cynognathus crateronotus Character = 51 BSP 1934 VIII3.

Diademodon tetragonus Character = 51 BSP 1934 VIII19.

Trirachodon spp. Character = 51 BP/1/4658.

Langbergia modisei Character = 51 NMQR 3255.

Sinognathus gracilis Character = 51 IVPP V2339.

Beishanodon youngi Character = 51 Gao et al (2010: fig. 4B).

Mandagomphodon hirschsoni Character = 51 Following interpretation of Hopson (2014: fig. 14.1b) of extension of the pterygoid it is scored as 0.

Mandagomphodon attridgei Character = 51 NHMUK R8578.

Gomphodontosuchus brasiliensis Character = 51 GPIT/RE/09397.

Scalenodontoides macrodontes Character = 51 BP/1/5395.

Therioherpeton cargnini Character = 51 Changed from ? to 0.

Chaliminia musteloides Character = 51 Changed from ? to 1. Inferred.

Riograndia guaibensis Character = 51 Changed from 1 to 0 (Agustin).

Trucidocynodon riograndensis Character = 51 Changed from 1 to ?. It is uncertain according to Oliveira et al 2010.

Oligokyphus spp. Character = 51 Changed from ? to 0.

Dvinia prima Character = 51 Changed from 1 to 0.

Bonacynodon schultzi Character = 51 Changed from 0 to ?.

52. Length of palatine relative to maxilla in secondary palate

0 shorter

1 about equal

2 longer

Procynosuchus delaharpeae Character = 52 RC 5, RC 12, NHMUK PV37054 .

Galesaurus planiceps Character = 52 NMQR 135.

Thrinaxodon liorhinus Character = 52 NHMUK R511.

Platycraniellus elegans Character = 52 Abdala, 2007. text-fig. 2B).

Cynognathus crateronotus Character = 52 BSP 1934 VIII6.

Diademodon tetragonus Character = 52 BP/1/2522.

Trirachodon spp. Character = 52 BP/1/4658.

Langbergia modisei Character = 52 NMQR 3255.

Beishanodon youngi Character = 52 Gao et al (2010: fig. 4B).

Scalenodon spp. Character = 52 Changed from 0 to ? Not possible to discriminate sutures between these bones in the available material.

Luangwa spp. Character = 52 UFRGS 267PV.

Mandagomphodon hirschsoni Character = 52 Hopson (2014: fig. 14.1b).

Mandagomphodon attridgei Character = 52 NHMUK R8578 .

Gomphodontosuchus brasiliensis Character = 52 GPIT/RE/09397.

Scalenodontoides macrodontes Character = 52 BP/1/5395.

Santacruzodon hopsoni Character = 52 After Melo et al (submitted).

Aleodon brachyramphus Character = 52 Scored after Hopson and Kitching (2001, char. 40).

Therioherpeton cargnini Character = 52 Changed from ? to 2.

Dadadon isaloi Character = 52 Changed from 1 to 2.

Chiniquodon spp. Character = 52 After Hopson and Kitching (2001, character 40).

Oligokyphus spp. Character = 52 Changed from ? to 0.

Tritylodon longaevus Character = 52 Changed from 2 to 1.

Bonacynodon schultzi Character = 52 Changed from 0 to ?.

Protheriodon estudianti Character = 52 Changed from ? to 0. .

53. Maxillary platform lateral to dentition (In this character, state 2, which is the well-developed platform, is scored for all traversodontids and some *Trirachodon* spp.tids. In traversodontids are cases in which, eventually, could be distinguished a platform less developed but the condition is not clear cut and Abdala et al. (2006) opted to consider only two state of characters for analysis of gomphodont cynodonts. This has been followed for most authors. Then the state 1 (incipient in posterior portion of the teeth row) is showing the condition developed in some Probainognathia (e.g., *Probainognathus jenseni*, Pachygenelus).)

0 absent

1 incipient in posterior portion of the teeth row

2 well developed

Charassognathus gracilis Character = 53 Changed from ? to 0.

Procynosuchus delaharpeae Character = 53 RC 5, RC 12, NHMUK PV37054.

Galesaurus planiceps Character = 53 NMQR 135.

Thrinaxodon liorhinus Character = 53 NHMUK R511.

Platycraniellus elegans Character = 53 Abdala, 2007. text-fig. 2B).

Cynognathus crateronotus Character = 53 BSP 1934 VIII6.

Diademodon tetragonus Character = 53 BP/1/2522.

Trirachodon spp. Character = 53 Abdala et al. (2006, character 22, pg. 410).

Langbergia modisei Character = 53 Abdala et al. (2006, character 22, pg. 410).

Sinognathus gracilis Character = 53 Changed from 1 to 2, after Liu and Olsen (2010), Abdala, pers. obs.

Beishanodon youngi Character = 53 Gao et al (2010: fig. 4B).

Scalenodon spp. Character = 53 Changed from 1 to 2. The platform is well developed along the entire line of postcanines (Abdala, pers. obs.).

Luangwa spp. Character = 53 Changed from 1 to 2. See Abdala et al. (2006, character 22, pg. 410).

Mandagomphodon hirschsoni Character = 53 The platform is well-developed (Hopson, 2014: fig. 14.1b). Changed from 1 to 2.

Mandagomphodon attridgei Character = 53 NHMUK R8578 .

Gomphodontosuchus brasiliensis Character = 53 Abdala et al. (2006). Liu and Abdala (2014). .

Scalenodontoides macrodontes Character = 53 See Gow and Hancox (1993) and Battail (2005).

Protuberum cabralense Character = 53 Changed from ? to 2.

Boreogomphodon jeffersoni Character = 53 Changed from ? to 2.

Brasilodon tetragonus Character = 53 After Bonaparte et al. (2005, fig. 5).

Chaliminia musteloides Character = 53 Small platform, especially lateral to the last two postcanines.

Riograndia guaibensis Character = 53 Changed from 0 to 1.

Dadadon isaloi Character = 53 Changed from ? to 2.

Menadon besairiei Character = 53 Incipiently developed (Abdala et al., 2006, character 22).

Elliotherium kersteni Character = 53 Sidor and Hancox (2006, fig. 4).

Pachygenelus monus Character = 53 Scored after Abdala (2007, character 23).

54. Maxilla in margin of subtemporal fenestra

0 excluded

1 included

Procynosuchus delaharpeae Character = 54 RC 5, RC 12.

Galesaurus planiceps Character = 54 NMQR 135.

Thrinaxodon liorhinus Character = 54 NHMUK R511.

Platycraniellus elegans Character = 54 Excluded by ectopterygoid and jugal. .

Cynognathus crateronotus Character = 54 BSP 1934 VIII3, BSP 1934 VIII6.

Diademodon tetragonus Character = 54 BSP 1934 VIII14, BSP 1934 VIII19.

Trirachodon spp. Character = 54 BP/1/4658.

Langbergia modisei Character = 54 NMQR 3255.

Sinognathus gracilis Character = 54 IVPP V2339.

Beishanodon youngi Character = 54 Gao et al (2010: fig. 4A, B). Excluded by the ectopterygoid and the jugale.

Scalenodon spp. Character = 54 Changed from 0 to ? Not possible to observe in the available material.

Luangwa spp. Character = 54 NHMUK PV R 36995.

Mandagomphodon hirschsoni Character = 54 Following interpretation of Hopson (2014: fig. 14.1b) of extension of the pterygoid it is scored as 0.

Mandagomphodon attridgei Character = 54 Last postcanines in eruption are placed in the margin of the subtemporal fenestra. These tooth are located in the maxilla and therefore this element form part of the margin of the fenestra.

Gomphodontosuchus brasiliensis Character = 54 Not possible to be certain in the only specimen.

Scalenodontoides macrodontes Character = 54 BP/1/5395.

Andescynodon mendozensis Character = 54 After Liu and Abdala (2014, char. 15).

Aleodon brachyramphus Character = 54 Changed from ? to 1.

Boreogomphodon jeffersoni Character = 54 After Liu and Abdala (2014, char. 15).

Brasilodon tetragonus Character = 54 Changed from ? to 1.

Chaliminia musteloides Character = 54 Inferred.

Ecteninion lunensis Character = 54 Changed from 0 to 1.

Chiniquodon spp. Character = 54 After Abdala (2007, char. 20).

Dvinia prima Character = 54 Abdala: Excluded (0). By the jugal and the ectopterygoid (see Tatarinov, 1968: fig. 2).^nBut observing the cast, the last postcanine is opening almost on the anterior margin of the fenestra. After this it seems likely that the maxilla were part of the subtemporal fenestra margin.^nIn the illustration by Hopson the maxilla seems to be also excluded from the margin by the jugal and pterygoid. I will accept this provisionally.^n.

Prozostrodon brasiliensis Character = 54 Changed from ? to 0.

55. Ectopterygoid

0 contacts maxilla

1 does not contact maxilla

2 absent

Procynosuchus delaharpeae Character = 55 RC 12.

Galesaurus planiceps Character = 55 NMQR 135, AMNH 2223.

Thrinaxodon liorhinus Character = 55 NHMUK R511.

Platycraniellus elegans Character = 55 Abdala, 2007. text-fig. 2B).

Cynognathus crateronotus Character = 55 BSP 1934 VIII6.

Diademodon tetragonus Character = 55 BSP 1934 VIII19.

Trirachodon spp. Character = 55 BP/1/4658. Tentative score.

Langbergia modisei Character = 55 NMQR 3255, NMQR 3251.

Beishanodon youngi Character = 55 Gao et al (2010: fig. 4B, pg. 13). The ectopterygoid is greatly reduced to a small bone occupying the more medial parts of the anterior border of the subtemporal fenestra. It articulates with the posterior border of the maxilla between the pterygoid and the curved anteromedial process of the jugal, and thereby prevents the maxilla from entering into the anterior border of the subtemporal fenestra.

Scalenodon spp. Character = 55 Available specimen poorly preserved.

Luangwa spp. Character = 55 NHMUK PV R 36995.

Mandagomphodon hirschsoni Character = 55 Following interpretation of Hopson (2014: fig. 14.1b) of extension of the pterygoid it is scored as 2.

Mandagomphodon attridgei Character = 55 Score tentative. The base of the pterygoid process is preserved on the right side and there is no efidence of the presence of the ectopterygoid.

Gomphodontosuchus brasiliensis Character = 55 Area not preserved in the only specimen.

Scalenodontoides macrodontes Character = 55 BP/1/5395.

Protuberum cabralense Character = 55 Changed from ? to 2.

Aleodon cromptoni Character = 55 Chnaged from ? to 2.

Ecteninion lunensis Character = 55 It is not possible to be sure of the contact or not with maxilla (Martinez, 1996, fig. 3B). Note that Hopson and Kitching (2001, char. 9) scored the bone as absent.^n^n.

Chiniquodon spp. Character = 55 Afater Abdala (2007, char. 20).

56. Interpterygoid vacuity in adults

0 present

1 absent

Procynosuchus delaharpeae Character = 56 RC5, RC 12, RC 5, RC 12, NHMUK PV37054.

Galesaurus planiceps Character = 56 NMQR 135.

Thrinaxodon liorhinus Character = 56 NHMUK R511.

Platycraniellus elegans Character = 56 Abdala, 2007. text-fig. 2B).

Cynognathus crateronotus Character = 56 BSP 1934 VIII3, BSP 1934 VIII6.

Diademodon tetragonus Character = 56 BSP 1934 VIII19.

Trirachodon spp. Character = 56 BP/1/4658.

Langbergia modisei Character = 56 NMQR 3255.

Sinognathus gracilis Character = 56 IVPP V2339.

Beishanodon youngi Character = 56 Gao et al. (2010: fig. 4B).

Scalenodon spp. Character = 56 Changed from 1 to ? Area not preserved in available specimens.

Luangwa spp. Character = 56 BP/1/3731.

Mandagomphodon hirschsoni Character = 56 Hopson (2014: fig. 14.2a).

Gomphodontosuchus brasiliensis Character = 56 Changed from 1 to ? Area not preserved i the only specimens known.

Scalenodontoides macrodontes Character = 56 BP/1/5395.

Andescynodon mendozensis Character = 56 Changed from ? to 1.

Aleodon brachyramphus Character = 56 Changed from 1 to ?.

Therioherpeton cargnini Character = 56 Changed from 1 to ?.

Boreogomphodon jeffersoni Character = 56 Changed from ? to 1.

Dadadon isaloi Character = 56 Changed from ? to 1.

Menadon besairiei Character = 56 Changed from ? to 1.

Adelobasileus cromptoni Character = 56 There is a foramen (pterygoid vacuity, pv) that is interpreted as homologous to the vacuity.

Cynosaurus suppostus Character = 56 It is present only in a small specimen, not in larger ones.

Abdalodon diastematicus Character = 56 After Botha-Brink and Abdala (2008). contra Kammerer 2016.

57. Boss crest anterior to the interpterygoid vacuity (This character is better represented in therocephalians, where several taxa have interpterygoid vacuity and a well-developed boss or crest anterior to the opening; a few cases are represented in cynodonts.)

0 reduced or absent

1 well developed

Procynosuchus delaharpeae Character = 57 RC5, RC 12, RC 5, RC 12, NHMUK PV37054.

Platycraniellus elegans Character = 57 Abdala, 2007. text-fig. 2B).

Chaliminia musteloides Character = 57 Changed from ? to 1.

Diarthrognathus broomi Character = 57 Changed from ? to 0. There is a small tuberosity.

Dvinia prima Character = 57 Abdala: There seems to be no crest or boss in the observed cast. So his score remain the same. But must be mentioned that in new illustration by Hopson the region anterior to the opening seems to be broke. .

Nanictosaurus kitchingi Character = 57 Changed from 0 to -.

Bonacynodon schultzi Character = 57 Changed from 0 to -.

Pseudotherium argentinus Character = 57 Wallace et al 2019 scored the presence of a distinct median crest in the pterygoid (their character 38). Check this scoring.

58. Pterygoid quadrate ramus

0 present

1 absent

Procynosuchus delaharpeae Character = 58 RC5, RC 12, BP/1/3748, NHMUK PV37054.

Galesaurus planiceps Character = 58 NMQR 135, SAM-PK-K9956.

Thrinaxodon liorhinus Character = 58 NHMUK R511. (Fourie, 1974, fig. 1).

Platycraniellus elegans Character = 58 Abdala, 2007. text-fig. 3).

Cynognathus crateronotus Character = 58 BSP 1934 VIII1.

Diademodon tetragonus Character = 58 MB R1004.

Trirachodon spp. Character = 58 BP/1/4658.

Langbergia modisei Character = 58 NMQR 3255.

Sinognathus gracilis Character = 58 IVPP V2339.

Beishanodon youngi Character = 58 Gao et al. (2010: fig. 4B). Tentative score, as preservation is not certain.

Scalenodon spp. Character = 58 UMZC T907. Tentative score.

Luangwa spp. Character = 58 BP/1/3731.

Mandagomphodon hirschsoni Character = 58 Relevant area not preserved. Changed from 1 to ?.

Gomphodontosuchus brasiliensis Character = 58 Changed from 1 to ? Relevant area not preserved in the only specimen known.

Scalenodontoides macrodontes Character = 58 Changed from 1 to ? Not or poorly preserved area in BP/1/5395.

Andescynodon mendozensis Character = 58 Changed from ? to 1.

Protuberum cabralense Character = 58 Changed from ? to 1.

Aleodon brachyramphus Character = 58 Changed from 1 to ?.

Boreogomphodon jeffersoni Character = 58 Changed from ? to 1.

Brasilodon tetragonus Character = 58 Changed from 1 to 0.

Chaliminia musteloides Character = 58 Not possible to distinguish the bones in the material.

Dadadon isaloi Character = 58 Ranivoharimanana et al (2011: 1297): "The quadrate rami of the pterygoids are short, with their posterior suture with the epipterygoid located at the mid-length of the temporal fenestra." We score as absent as they are not sending the projection laterally towards the quadrate. The only lengthy process described is for the

epipterygoid: "The epipterygoid has a lengthy posterior process that undercuts the prootic for most of its lateral exposure, extending nearly to the quadrate".

Menadon besairiei Character = 58 Melo et al. (2015): "The quadrate ramus of the pterygoid extends to the level of the anterior margin of the cavum epiptericum." As this structure are not projected lateraly towards the quadrate they are scored as absent.

Trucidocynodon riograndensis Character = 58 Changed from 1 to ?. It is uncertain according to Oliveira et al 2010.

Oligokyphus spp. Character = 58 Changed from ? to 1.

Tritylodon longaevus Character = 58 Changed from 1 to 0.

Diarthrognathus broomi Character = 58 Changed from 1 to 0.

Dvinia prima Character = 58 Changed from 0 to ?^nAbdala: The rama is not preserved in the cast and thus probably is better consider as ? .

Progalesaurus lootbergensis Character = 58 Changed from ? to 0.

59. Quadrate rami of epipterygoid

0 absent

1 present but do not contact quadrate

2 present and contact quadrate

Procynosuchus delaharpeae Character = 59 BP/1/3748, Brink (1963, fig. 10B).

Galesaurus planiceps Character = 59 BP/1/5064. Tentative score.

Thrinaxodon liorhinus Character = 59 Fourie (1974): These authors (Olson,1944 and Parrington, 1946) have also shown that the posterior tips of the quadrate ramus of the pterygoid (QRPT) and the lateral lamina of the prootic (PROF) meet the medial surface of the quadrate (Figs 1, 19C, 26).

Platycraniellus elegans Character = 59 Quadrate ramus of the epipterygoid appears very posteriorly extended after interpretation of Abdala (2007: text-fig. 3). Based on that it is scored as contacting quadrate. Tentative score.

Cynognathus crateronotus Character = 59 BSP 1934 VIII1.

Diademodon tetragonus Character = 59 USNM 22937.

Trirachodon spp. Character = 59 BP/1/4658. On the left side the quadrate ramus of the epipterygoid reaches near to the area of the squamosal where was the quadrate, suggesting the contact betwen these bones. Contact observed in CGP unnumbered.

Langbergia modisei Character = 59 NMQR 3255.

Sinognathus gracilis Character = 59 The process is present and it is long. On the left side is twisted posteriorly out of place and correction of deformation allow to infer that there was contact. Tentative score.

Beishanodon youngi Character = 59 Gao et al. (2010: fig. 4B).

Luangwa spp. Character = 59 BP/1/3731. Tentative score.

Mandagomphodon hirschsoni Character = 59 Relevant area not preserved. Changed from 1 to ?.

Scalenodontoides macrodontes Character = 59 Changed from 1 to ? Not posible to see in the available material.

Protuberum cabralense Character = 59 Changed from ? to 1.

Boreogomphodon jeffersoni Character = 59 Changed from ? to 1.

Brasilodon tetragonus Character = 59 Changed from ? to 1/2 (presente Agustin).

Riograndia guaibensis Character = 59 Changed from ? to 1/2 (probably present, Soares et al 2011).

Menadon besairiei Character = 59 Changed from ? to 1 (it is described as ending at the anterior margin of the cavum epiptericum.

Trucidocynodon riograndensis Character = 59 Changed from 1 to 1/2 as Oliveira states that it is present but its development is unknown.

Oligokyphus spp. Character = 59 Changed from ? to 0. It is absent or very short.

Kayentatherium wellesi Character = 59 Changed from 0 to 1 (see Sues 1986).

Bienotherium spp. Character = 59 Changed from 0 to 1.

Elliotherium kersteni Character = 59 Changed from ? to 1/2 (it is present but the posterior extension is unknown).

Pachygenelus monus Character = 59 Changed from ? to 1/2 as I cannot be certain about this.

Diarthrognathus broomi Character = 59 Changed from ? to 2.

Dvinia prima Character = 59 Changed from 1 to ? ^nAbdala: Following Tatarinov (1968: 15): ?The posterior ramus of the pterygoid is connected with the quadrate, but the epipterygoid evidently terminates at the lateral flank of the prooticum, external to the pterygoparaoccipital foramen?. ^nIn the cast observed in Berkley both epipterygoid and pterygoid quadrate rami do not seems to be preserved.^nThe expected/probable condition should be present and do not contact, but the fact is that the rama is not preserved in the material. Then the score should change to ? The same is clear from illustrations by Hopson.

Cynosaurus suppostus Character = 59 Changed from 1/2 to 2.

Bonacynodon schultzi Character = 59 Present but it is not possible to see whether it contacts the quadrate or not.

60. Quadrate ramus of pterygoid epipterygoid (Originally proposed by Rowe (1988, character 37), the state 1 is characterizing tritylodontids, with the epipterygoid notably extended ventrally below the level of the basicranium.)

0 at the same level as basioccipital

1 ventrally expanded below basioccipital

Procynosuchus delaharpeae Character = 60 BP/3748, RC 92.

Beishanodon youngi Character = 60 Considering the condition visible in ventral view of Gao et al (2010, fig. 4B) the pattern of the quadrate ramus seems to be similar to that of most nm cynodonts and different to the condition of tritylodontids. Score tentative.

Andescynodon mendozensis Character = 60 Changed from ? to 0.

Protuberum cabralense Character = 60 Changed from ? to 0.

Boreogomphodon jeffersoni Character = 60 Changed from ? to 0.

Chaliminia musteloides Character = 60 Changed from ? to 0.

Riograndia guaibensis Character = 60 Changed from ? to 0.

Dadadon isaloi Character = 60 Changed from ? to 0.

Menadon besairiei Character = 60 Changed from ? to 0.

Oligokyphus spp. Character = 60 Changed from ? to -.

Bienotherium spp. Character = 60 Changed from ? to 1.

Elliotherium kersteni Character = 60 Changed from ? to 0.

Diarthrognathus broomi Character = 60 Changed from ? to 0.

Nanictosaurus kitchingi Character = 60 Changed from ? to 0.

Bonacynodon schultzi Character = 60 Changed from ? to 0.

Pseudotherium argentinus Character = 60 Changed from ? to 0.

61. Parasphenoid ala (Character proposed by Rowe (1988, character 40), stated as 0, small, 1. widely flared ventrolaterally (scored only in tritylodontids) and 2. fused to auditory capsula (scored in mammaliaformes and mammals). Hopson and Kitching (2001, character 17) stated as ventral surface of basisphenoid depressed below occipital condyles, 0. less than 1/4 occipital height, 1. greater than 1/4 occipital height (scored in *Scalenodon angustifrons*, *Massetognathus* spp. and tritylodontids). Abdala (2007, character 29) stated as parasphenoid ala, 0. as same level as basicranium, 1. ventrally expanded below basicranium (scored only in *Kayentatherium wellesi*).)

0 at same level as basicranium

1 ventrally expanded below basicranium

Procynosuchus delaharpeae Character = 61 RC 92, BP/1/3748.

Beishanodon youngi Character = 61 Considering the condition visible in ventral view of Gao et al (2010, fig. 4B) the parasphenoid is at the same level than basicranium.

Mandagomphodon hirschsoni Character = 61 Relevant area not preserved. Changed from 0 to ?.

Andescynodon mendozensis Character = 61 Changed from ? to 0.

Protuberum cabralense Character = 61 Changed from ? to 0.

Boreogomphodon jeffersoni Character = 61 Changed from ? to 0.

Chaliminia musteloides Character = 61 Changed from ? to 0.

Dadadon isaloi Character = 61 Changed from ? to 0.

Menadon besairiei Character = 61 Changed from ? to 0.

Elliotherium kersteni Character = 61 Changed from ? to 0.

Diarthrognathus broomi Character = 61 Changed from ? to 0.

Nanictosaurus kitchingi Character = 61 Changed from ? to 0.

Bonacynodon schultzi Character = 61 Changed from ? to 0.

62. Parasphenoid ala

0 long and borders fenestra ovalis

1 slightly reduced and excluded from fenestra ovalis

2 absent

Procynosuchus delaharpeae Character = 62 BP/1/3748, NHMUK PV37054 .

Galesaurus planiceps Character = 62 SAM-PK-K9956.

Thrinaxodon liorhinus Character = 62 NHMUK R511.

Platycraniellus elegans Character = 62 The basisphenoid apper to extend well posteriorly in NMQR 1633 (Abdala, 2007, text-fig. 4).

Cynognathus crateronotus Character = 62 BP/1/1181, SAM-PK-K11264, BSP 1934 VIII4.

Diademodon tetragonus Character = 62 BP/1/3754. BP/1/3639.

Trirachodon spp. Character = 62 SAM-PK-K4801, BP/1/4658.

Langbergia modisei Character = 62 NMQR 3255.

Sinognathus gracilis Character = 62 IVPP V2339. Tentative score.

Scalenodon spp. Character = 62 UMZC T907. Tentative score.

Luangwa spp. Character = 62 BP/1/3731. Tentative score.

Boreogomphodon jeffersoni Character = 62 Changed from 0 to ?.

Trucidocynodon riograndensis Character = 62 Unable to check this scoring.

Tritylodon longaevus Character = 62 I was unable to check this scoring.

Sinoconodon rigneyi Character = 62 It was previously scored as: basisphenoid does not overlap prootic pars cochlearis.

Morganucodon spp. Character = 62 It was previously scored as: basisphenoid does not overlap prootic pars cochlearis.

Dvinia prima Character = 62 Long and borders the fenestra ovalis (0). The ala is quite long in comparison to other cynodonts but in the reconstruction provided by TAtarinov (1968: fig. 4) the ala is just excluded from the border of the fenestra ovalis. This is confirmed after drawing by Jim Hopson. But with new interpretations of the limits of the fenestra ovalis, that shows that the ala was not excluded from the border of the fenestra ovalis. Abdala observation of June 2009: the score 0 was observed clearly on the left side of specimen PIN 2005/2465.

Cynosaurus suppostus Character = 62 Changed from 0 to 1.

Progalesaurus lootbergensis Character = 62 Changed from 0 to ?.

Nanictosaurus kitchingi Character = 62 I was unable to check this.

Pseudotherium argentinus Character = 62 Wallace et al. (2019. 41): "Behind the rostrum are the parasphenoid wings or alae, which flare laterally and underlap the ventral surface of the basisphenoid. The parasphenoid ala terminates at the anterior end of the pars cochlearis and does not participate in the border of fenestra vestibuli."

63. Paroccipital process

0 does not contact quadrate

1 contacts quadrate

2 crista parotica contacts quadrate

Procynosuchus delaharpeae Character = 63 BP/1/3748, AMNH 8220.

Platycraniellus elegans Character = 63 Abdala (2007, 597): The paroccipital process of the left side is located very close to the medial condyle of the quadrate trochlea, but there seems to be no contact between them.

Langbergia modisei Character = 63 Abdala et al. (2006, 394): Medially, the quadrate is in contact with the quadrate ramus of the epipterygoid and the squamosal, the latter interposed between the paroccipital process and the quadrate.

Beishanodon youngi Character = 63 Gao et al. (2010: 13):The paroccipital process is partly exposed on the left side of the holotype. its lateral end contacts the squamosal and MAY ALSO have a small contact with the medial side of the quadrate. Tentative score.

Mandagomphodon hirschsoni Character = 63 Hopson (2014: fig. 14.3b).

Andescynodon mendozensis Character = 63 Changed from ? to 0.

Protuberum cabralense Character = 63 Changed from ? to 0.

Aleodon brachyramphus Character = 63 Changed from 0 to ?.

Boreogomphodon jeffersoni Character = 63 Changed from ? to 0.

Adelobasileus cromptoni Character = 63 Changed from 0 to ? as the lateral end of the proccipital process is unknown.

Oligokyphus spp. Character = 63 Changed from 2 to 1. There is not crista parotica in *Oligokyphus* spp.. .

Tritylodon longaevus Character = 63 I was unable to check this scoring.

Diarthrognathus broomi Character = 63 Changed from 0 to 1.

Cynosaurus suppostus Character = 63 Changed from 0 to 1.

Pseudotherium argentinus Character = 63 Changed from ? to 1.

64. Cavum epiptericum

0 open ventrally below trigeminal ganglion

1 partial prootic floor

2 complete prootic floor

Procynosuchus delaharpeae Character = 64 RC 92, BP/1/3748.

Galesaurus planiceps Character = 64 SAM-PK-K9956.

Thrinaxodon liorhinus Character = 64 NHMUK R511.

Platycraniellus elegans Character = 64 Abdala (2007, text-fig. 3).

Cynognathus crateronotus Character = 64 SAM-PK-K11264, BSP 1934 VIII4.

Diademodon tetragonus Character = 64 BP/1/3754. BP/1/3639.

Trirachodon spp. Character = 64 SAM-PK-K4801, BP/1/4658.

Langbergia modisei Character = 64 NMQR 3255.

Scalenodon spp. Character = 64 UMZC T907.

Luangwa spp. Character = 64 NHMUK PV R 36995.

Pascualgnathus polanski Character = 64 Changed from ? to 0.

Protuberum cabralense Character = 64 Changed from ? to 0.

Aleodon cromptoni Character = 64 Chnaged from 0 to ?.

Boreogomphodon jeffersoni Character = 64 Changed from ? to 0.

Dadadon isaloi Character = 64 Changed from ? to 0.

Menadon besairiei Character = 64 Changed from ? to 0.

Nanictosaurus kitchingi Character = 64 Changed from ? to 0.

65. Promontorium (Promontorium is part of the petrosal bone linked with the ears system, specifically housing the cochlea. This is structure is developed in Mammaliaformes and *Brasilodon tetragonus*tids. We are not certain that a promontorium is present in *Pseudotherium argentinus* as argued by Wallace et al. (2019). They discuss this structure in the text but there is no indication in any of their detailed figures illustrating the specimen.)

0 absent

1 present

Procynosuchus delaharpeae Character = 65 BP/1/3748, NHMUK PV37054 .

Platycraniellus elegans Character = 65 Abdala (2007, text-figs. 3, 4).

Beishanodon youngi Character = 65 Gao et al. (2010: fig. 4A). Considering the preserved portion of the parasphenoid it is clear the typial condition of nm cynodonts lacking the promontorium.

Mandagomphodon hirschsoni Character = 65 Changed from ? to 0 (see Hopson, 2014: fig. 14.3b). There is a ridge in front of the fenestra ovalis, showing that there is no promontorium.

Andescynodon mendozensis Character = 65 Changed from ? to 0.

Protuberum cabralense Character = 65 Changed from ? to 0.

Boreogomphodon jeffersoni Character = 65 Changed from ? to 0.

Dadadon isaloi Character = 65 Changed from ? to 0.

Menadon besairiei Character = 65 Changed from ? to 0.

Nanictosaurus kitchingi Character = 65 Changed from ? to 0.

66. Fenestra rotunda and jugular foramen (Wible (1991, 6): "Fenestra rotundum (sic)- Confluent with (0) or separated from jugular foramen (1). "[R60: Mammaliaformes]. Of the taxa considered in Rowe's (1988) principal analysis, the round window (fenestra rotunda) and jugular foramen also are separate in the tritylodontids *Oligokyphus* spp. (Crompton, 1964:fig.8) and *Tritylodon longaevus* (Gow, 1986a:fig. 16). Consequently, the derived state is scored for tritylodontidae here. In addition, a separate fenestra rotunda occurs in tritheledontids (*Pachygenelus monus* monus SAM K1350), *Sinoconodon rigneyi* (Kermack et al., 1981:fig. 105), the triconodontids Troracodon and Triconodon (Kermack, 1963:figs. 3, 8), and Vincelestes (Bonaparte and Rougier, 1987:fig. 4A). Liu and Olsen (2010, appendix): Luo, Character 42 coded tritylodontidae as confluent, Trithelodontidae as narrowly separated. Hopson and Kitching (2001, character 42) coded them as ?completely separated?. Although (Crompton, 1964) fig 8C showed the separation of two foramina in *Oligokyphus* spp., I did not observed it on specimens and this case is not known in other tritylodontids. Although they could be separated in *Oligokyphus* spp. major, this condition can only be coded in well preserved and prepared specimen; for the accuracy of coding, I use the simplest coding for this character.)

0 confluent

1 separated

Procynosuchus delaharpeae Character = 66 NHMUK PV37054, BP/1/3748 .

Platycraniellus elegans Character = 66 Abdala (2007, text-fig. 4).

Andescynodon mendozensis Character = 66 Changed from ? to 0.

Aleodon cromptoni Character = 66 Chnaged from 0 to ?.

Brasilodon tetragonus Character = 66 Changed from 1 to 0 (Agustín).

Dadadon isaloi Character = 66 Changed from ? to 0.

Menadon besairiei Character = 66 Changed from ? to 0.

Nanictosaurus kitchingi Character = 66 Changed from ? to 0.

Irajatherium hernandezi Character = 66 partially separated by finger-like projection from postero-lateral wall of jugular foramen.

67. Vascular foramen in posterior part of lateral flange (Liu and Olsen (2010) say that this character refers to foramen x of Rougier et al (1992): "A second vascular channel lies above the lateral flange vascular canal in some tritylodontids and early mammals. We refer to this second foramen through the lateral flange as foramen "X" in the discussion below. It passes through the lateral flange posterodorsal to the foramen for the third division of the trigeminal nerve to open into the vertical trough anterior to the paroccipital process. Foramen "X" has been described for the tritylodontids *Tritylodon longaevus* (Gow, 1986b), *Oligokyphus* spp. (Kühne, 1956; Crompton, 1964), and *Kayentatherium wellesi* (Sues, 1986) and in *Sinoconodon rigneyi* (Crompton and Luo, 1993) and the *Morganucodon* spp.tid *Megazostrodon* (Gow, 1986a). A small opening occurs in a topographically similar position, posterodorsal to the foramen for the mandibular nerve, in *Morganucodon* spp. (Fig. 8A: nf?; Kermack et al., 1981), but this may be a nutrient foramen as no posterior (or intracranial) outlet was detected (Hopson, unpubl. obs.)". To identify the structure see Gow (1986; fig. 16 f. St. a.), The foramen is located immediately anterior to the pterygoparaoccipital foramen in ventral view. Liu and Olsen (2010) also scored this foramen as present in *Brasilodon tetragonus*.

0 absent

1 present

Procynosuchus delaharpeae Character = 67 NHMUK PV37054.

68. Route of venous drainage from back of cavum epiptericum (Rowe (1988) character 48: Prootic posterolateral flange, absent (0) or present (1) is identified as a synapomorphy at node 1 (advanced cynodonts including traversodontids, tritylodontids, tritheledontids and mamaliaformes). Wible (1991) change the wording referring to the presence of a vascular foramen in the flange: character 53. Prootic lateral flange-vascular foramen or canal absent (0) or present (1). He mentions the presence of this structure in *Luangwa* spp., *Exaeretodon* spp. and tritylodontids. Wible and Hopson (1993) take the character as Wible (1991) with a small change in the wording: character 22. Vascular foramen in periotic (prootic) lateral flange, absent (0) or present (1). Hopson and Kitching (2001) modify this character substantially: character 27: Groove on prootic extending from pterygoparoccipital foramen to trigeminal foramen: absent (0), present and open (1), present and enclosed as a canal (2). In this case he incorporates also the presence of an open groove which is typical of several non-mammaliaform cynodonts.

0 only vascular groove on lateral flange

1 absent

2 vascular canal on lateral flange (foramina on lateral surface)

Procynosuchus delaharpeae Character = 68 Liu and Olsen (2010, appendix): AMNH8220, RC 5, [(Kemp, 1979) p104 stated the groove is absent in his specimen, but Rougier (1992) said a sulci present in AMNH 8220. Based on my observation, only a faint trace is present in AMNH 8220. and it is tentatively coded as groove present]. We agree with the comment by Liu and Olsen (2010). Also observed in BP/1/1545. But on the other hand on the left side of NHMUK PV37054 there seems not to be evidence of this feature.

Galesaurus planiceps Character = 68 AMNH 2223.

Thrinaxodon liorhinus Character = 68 TM 5074.

Platycraniellus elegans Character = 68 Observed in NMQR 1633.

Cynognathus crateronotus Character = 68 BSP 1934 VIII3. Well preserved lateral wall of the skull. No evidence of lateral flange groove.

Diademodon tetragonus Character = 68 MB R1004.

Trirachodon spp. Character = 68 BP/1/5050. Gow (1986, fig. 3a) illustrate this specimen but it seems to be remnant of an open groove. Hopson and Kitching (2001, character 27) scored this as showing a canal. We change from state 2 to state 0 tentatively.

Langbergia modisei Character = 68 Changed from 2 to 1. I canot recognize any structure between the trigeminal foramen and pterygoparaoccipital foramen. Tentative score.

Scalenodon spp. Character = 68 Changed from 2 to ?. Available specimens does not the area well preserved to be certain of the presence of the grove or the canal. The latter condition was scored by Hopson and Kitching (2001).

Luangwa spp. Character = 68 Kemp (1980: 211). Wible (1991) corroborate the presence of this foramen in *Luangwa* spp., but not Wible and Hopson (1993).

Pascualgnathus polanski Character = 68 Changed from 2 to ?.

Probainognathus jenseni Character = 68 Changed from 1 to 0.

Riograndia guaibensis Character = 68 Changed from 1 to ?. This is not vissible.

69. Pterygoparoccipital foramen

0 squamosal does not contribute to enclosure of foramen

1 squamosal contributes to enclosure of foramen

2 open as a notch

Procynosuchus delaharpeae Character = 69 NHMUK PV37054, AMNH FR 8020 .

Galesaurus planiceps Character = 69 NMQR 135.

Thrinaxodon liorhinus Character = 69 NHMUK R511.

Platycraniellus elegans Character = 69 Abdala (2007, text-fig. 3).

Cynognathus crateronotus Character = 69 SAM-PK-K11264, BSP 1934 VIII1.

Diademodon tetragonus Character = 69 MB R1004.

Trirachodon spp. Character = 69 SAM-PK-K4801.

Sinognathus gracilis Character = 69 IVPP V2339.

Scalenodon spp. Character = 69 UMZC T907. Parrington (1946, fig. 9A). Tentative score.

Scalenodontoides macrodontes Character = 69 Changed from 1 to ? Relevant area not preserved.

Andescynodon mendozensis Character = 69 I was not able to check this.

Probainognathus jenseni Character = 69 Changed from 0 to 1.

Menadon besairiei Character = 69 Changed from 0 to ?. This is not described or illustrated.

Kayentatherium wellesi Character = 69 I am not sure about this scoring. Sues (1986) stated that the squamosal borders the pterygoparoccipital foramen. .

Pachygenelus monus Character = 69 I am not sure about this. It appears to be a foramen not a notch.

Progalesaurus lootbergensis Character = 69 I was unable to check this.

70. Lateral flange of prootic (This character is about the curved vertical component of the lateral flange. A structure that is only known in tritylodontids and *Sinoconodon rigneyi*. Character proposed by Luo (1994): 52. Lateral flange of the petrosal (prootic) forming lateral shelf perpendicular to anterior lamina (0); lateral flange forming broad shelf with vertical component (L-shaped) anterior to pterygoparoccipital foramen (1); lateral shelf reduced (2). This was modified by Luo Crompton and Sun (2001) to character 25: vertical component flange (L-shaped and forming a vertical wall to the pterygoparaoccipital foramen): present (0); absent (1). Reworded by Liu and Olsen (2010, character 59) as used here.)

0 lacks vertical component

1 includes vertical component so that flange is L shaped and forms vertical wall adjacent to pterygoparoccipital foramen (This character is about the curved vertical component of the lateral flange. A structure that is only known in tritylodontids and *Sinoconodon rigneyi*. Character proposed by Luo (1994): 52. Lateral flange of the petrosal (prootic) forming lateral shelf perpendicular to anterior lamina (0); lateral flange forming broad shelf with vertical component (L-shaped) anterior to pterygoparoccipital foramen (1); lateral shelf reduced (2). This was modified by Luo Crompton and Sun (2001) to character 25: vertical component flange (L-shaped and forming a vertical wall

to the pterygoparaoccipital foramen): present (0); absent (1). Reworded by Liu and Olsen (2010), character 59 as used here.)

Protuberum cabralense Character = 70 Changed from 1 to ?. .

Morganucodon spp. Character = 70 Changed from 0 to 1.

71. Anterior part of paroccipital process

0 lateral aspect covered by squamosal

1 lateral aspect exposed due to dorsal withdrawal of squamosal (Original formulation by Luo (1994): 47. Anterior paroccipital process: laterally covered by squamosal (0); bulbous, without squamosal cover (1); crest of crista parotica and fossa incudis differentiated without squamosal cover (2). Score 1 is only for tritylodontids; score 0 for all NMC plus *Sinoconodon rigneyi* and Adelobasileus,; score 2 is for *Morganucodon* spp.. Character modified by Luo, et al. (2001) as follow: 22. Relationship of the squamosal to the anterior part of paroccipital process (Modified from Luo, 1989; Rougier et al., 1996a: ch 23): (a) Squamosal covers the lateral aspect of the anterior paroccipital process: *Probainognathus jenseni* (out-group), tritheledontids, Adelobasileus, *Sinoconodon rigneyi*, Zhangheotherium, Vincelestes, metatherians, eutherians; (b) Squamosal is dorsally withdrawn from the paroccipital region so the anterior part of paroccipital is exposed: tritylodontids, *Morganucodon* spp., Haldanodon, Hadrocodium,triconodontines, Ornithorhynchus, multituberculates. Liu and Olsen (2010, character 60) state the character as here.)

Procynosuchus delaharpeae Character = 71 NHMUK PV37054 .

Platycraniellus elegans Character = 71 Abdala (2007, text-fig. 4).

Beishanodon youngi Character = 71 Gao et al. (2010: 13): The paroccipital process is partly exposed on the left side of the holotype. its lateral end contacts the squamosal^n.

Mandagomphodon hirschsoni Character = 71 Hopson (2014: fig. 14.3b).

Scalenodontoides macrodontes Character = 71 BP/1/5395. Inferred after development of the left zygoma which is quite high and probably covered laterally the paroccipital process.

Brasilodon tetragonus Character = 71 Changed from ? to 1.

Sinoconodon rigneyi Character = 71 I am not sure about this.

Dvinia prima Character = 71 Changed from ? to 0.

Cynosaurus suppostus Character = 71 Changed from ? to 0.

Nanictosaurus kitchingi Character = 71 Changed from ? to 0.

72. Carotid artery foramina in basisphenoid

0 present

1 absent*Procynosuchus delaharpeae* Character = 72 NHMUK PV37054 .

Galesaurus planiceps Character = 72 SAM-PK-K1119.

Thrinaxodon liorhinus Character = 72 TM 80.

Platycraniellus elegans Character = 72 Score changed from 0 to 1. Abdala (2007, 597): There is no evidence of the carotid opening on the basisphenoid.

Cynognathus crateronotus Character = 72 SAM-PK ?K11264, BP/1/1181.

Diademodon tetragonus Character = 72 BP/1/3754.

Trirachodon spp. Character = 72 BP/1/4658.

Langbergia modisei Character = 72 NMQR 3255.

Sinognathus gracilis Character = 72 IVPP V2339.

Scalenodon spp. Character = 72 UMZC T907. Tentative score by poor preservation.

Luangwa spp. Character = 72 NHMUK PV R 36995.

Mandagomphodon hirschsoni Character = 72 Relevant area not preserved. Changed from 1 to ?.

Aleodon brachyramphus Character = 72 Changed from 0 to ?.

Exaeretodon spp. Character = 72 Changed from 1 to 0. It is illustrated by Bonaparte (1962) although not named. It can be observed, for example, in MACN 18125. It also appears to be present in the holotype of E. riograndensis.

Dvinia prima Character = 72 Changed from 0 to 1.^nAbdala: Present (0) after Tatarinov (1968. fig. 2, cc).^nHopson states that there is not carotid foramen identifiable. I then change the score to absent.^nAbsent (1).^n.

Cynosaurus suppostus Character = 72 Changed from 0 to ?.

Cricodon metabolus Character = 72 Changed from ? to 0 after C. metabolus (NHCC LB28).

73. Mandibular symphysis

0 unfused

1 fused

Procynosuchus delaharpeae Character = 73 AMNH FR 8220, NHMUK PV37054.

Galesaurus planiceps Character = 73 SAM-PK-K9956.

Thrinaxodon liorhinus Character = 73 BP/1/7199.

Cynognathus crateronotus Character = 73 BP/1/1181.

Diademodon tetragonus Character = 73 MB R1004.

Trirachodon spp. Character = 73 BP/1/4658.

Langbergia modisei Character = 73 NMQR 3356.

Sinognathus gracilis Character = 73 IVPP V2339.

Scalenodon spp. Character = 73 UMZC T908, UMZC T909.

Luangwa spp. Character = 73 BP/1/3731.

Mandagomphodon hirschsoni Character = 73 Hopson (2014. fig. 14.4a).

Gomphodontosuchus brasiliensis Character = 73 GPIT/RE/09397.

Scalenodontoides macrodontes Character = 73 MNHN 1957-23.

Boreogomphodon jeffersoni Character = 73 Changed from ? to 1.

Arctotraversodon plemmyridon Character = 73 Changed from ? to 1.

Traversodon stahleckeri Character = 73 Changed from ? to 1 after von Huene 1936.

Chaliminia musteloides Character = 73 Changed from 1 to 0.

Dadadon isaloi Character = 73 Changed from ? to 1.

Pachygenelus monus Character = 73 Changed from 1 to 0.

Diarthrognathus broomi Character = 73 Changed from 1 to ?.

Cricodon metabolus Character = 73 Changed from ? to 1 after Sidor and Hopson, 2018: C. metabolus (NHCC LB28).

Tessellatia bonapartei Character = 73 It is interpreted to be 0 unfused, but it is not possible to be sure.

Protheriodon estudianti Character = 73 Changed from 1 to 0 after fig 2 in Bonaparte et al 2016.

Microconodon tenuirostris Character = 73 Sues 2001 says the dentaries are fused.

74. Lateral crest of dentary

0 absent

1 incipient

2 well developed

3 strongly projected

Procynosuchus delaharpeae Character = 74 AMNH FR 8220, NHMUK PV37054, RC 92.

Galesaurus planiceps Character = 74 BP/1/5064.

Thrinaxodon liorhinus Character = 74 NHMUK 511.

Platycraniellus elegans Character = 74 Abdala (2007: 598):The lateral crest of the dentary, visible on the right side, is low,.

Cynognathus crateronotus Character = 74 PVL 3859, BP/1/1181.

Diademodon tetragonus Character = 74 MB R1004.

Trirachodon spp. Character = 74 BP/1/4658, AM 461.

Langbergia modisei Character = 74 NMQR 3256.

Sinognathus gracilis Character = 74 IVPP V2339.

Scalenodon spp. Character = 74 Changed from 2 to ? Not preserved in any available specimen.

Luangwa spp. Character = 74 BP/1/3731.

Mandagomphodon hirschsoni Character = 74 Observing photos and Hopson (2014:fig. 14.4b) does not seems to be a very protruded lateral crest on the dentary. Score tentative as incipient.

Santacruzodon hopsoni Character = 74 Changed from ? to 1.

Aleodon cromptoni Character = 74 I am not sure.

Boreogomphodon jeffersoni Character = 74 Changed from ? to 2.

Arctotraversodon plemmyridon Character = 74 Changed from ? to 1.

Traversodon stahleckeri Character = 74 Changed from ? to 1 after von Huene 1936.

Chaliminia musteloides Character = 74 Changed from ? to 2.

Dadadon isaloi Character = 74 Changed from ? to 0.

Menadon besairiei Character = 74 Changed from ? to 0.

Sinoconodon rigneyi Character = 74 I was unable to check this.

Cricodon metabolus Character = 74 Changed from ? to 2. It appears to be well developed in *C. metabolus* (NHCC LB28) according to Sidor and Hopson, 2018.

Protheriodon estudianti Character = 74 Changed from ? to 1after fig 1 in Bonaparte et al 2016.

Prozostrodon brasiliensis Character = 74 Changed from 3 to ? as only the medial view of the dentary is available.

Microconodon tenuirostris Character = 74 ROM 44300.

75. Masseteric fossa in dentary (The fossa high in the coronoid process is present in *Procynosuchus delaharpeae*, *Abdalodon diastematicus*, *Dvinia prima* and *Abdalodon muchingaensis* recently described (Huttenlocker and Sidor, 2020). Remaining non mammalian cynodonts show the fossa extended to the angle of the dentary.)

0 absent

1 fossa high on coronoid process

2 fossa extends to angle of dentary

Procynosuchus delaharpeae Character = 75 AMNH FR 8220, NHMUK PV37054, RC 92, BP/1/3748.

Galesaurus planiceps Character = 75 BP/1/5064.

Thrinaxodon liorhinus Character = 75 NHMUK 511.

Platycraniellus elegans Character = 75 TM 25. Tentative score, inferred after the preserved portion of the right dentary.

Cynognathus crateronotus Character = 75 PVL 3859, BP/1/1181.

Diademodon tetragonus Character = 75 MB R1004.

Trirachodon spp. Character = 75 BP/1/4658, AM 461.

Langbergia modisei Character = 75 NMQR 3256.

Sinognathus gracilis Character = 75 IVPP V2339.

Scalenodon spp. Character = 75 UMZC T907 (Abdala and Teixeira, 2004, fig. 8a).

Luangwa spp. Character = 75 BP/1/3731, MCP 3167PV.

Mandagomphodon hirschsoni Character = 75 Hopson (2014: fig. 14.4b).

Gomphodontosuchus brasiliensis Character = 75 Inferred after the right dentary of GPIT/RE/09397.

Scalenodontoides macrodontes Character = 75 MNHN 1957-23.

Andescynodon mendozensis Character = 75 Changed from ? to 2.

Boreogomphodon jeffersoni Character = 75 Changed from ? to 2.

Arctotraversodon plemmyridon Character = 75 Changed from ? to 2.

Traversodon stahleckeri Character = 75 Changed from ? to 2 after von Huene 1936.

Chaliminia musteloides Character = 75 Changed from ? to 2.

Riograndia guaibensis Character = 75 Changed from ? to 2.

Dadadon isaloi Character = 75 Changed from ? to 2.

Tritylodon longaevus Character = 75 Changed from ? to 2.

Bienotherium spp. Character = 75 Changed from ? to 2.

Cricodon metabolus Character = 75 Changed from ? to 2 after C. metabolus (NHCC LB28).

Bonacynodon schultzi Character = 75 Changed from ? to 2.

Protheriodon estudianti Character = 75 Changed from ? to 2.

Santacruzgnathus abdalai Character = 75 Probably extends to the dentary angle as the ventroanterior end of the masseteric fossa is visible. Changed from ? to 2.

Microconodon tenuirostris Character = 75 ROM 44300.

76. Base of coronoid process extension in lateral view (Character proposed by Abdala (2007, character 50). This is maintained as observational character as it is difficult to convey in ratios. There is a clear-cut between the conditions observed in therocephalians and cynodonts. There are two stages proposed for cynodonts but the proposed scored need more scrutiny. The moderately expanded was scored by Abdala (2007) in *Procynosuchus delaharpeae* and *Dvinia prima*.

0 relatively narrow

1 moderately expanded anteroposteriorly

2 very expanded anteroposteriorly

Charassognathus gracilis Character = 76 Changed from ? to 1.

Mandagomphodon hirschsoni Character = 76 After the preserved portion it is inferred it should be very expanded. Score tentative. See my comments in character.

Andescynodon mendozensis Character = 76 Changed from ? to 2.

Santacruzodon hopsoni Character = 76 Changed from ? to 2.

Aleodon brachyramphus Character = 76 Chnaged from ? to 2.

Boreogomphodon jeffersoni Character = 76 Changed from ? to 2.

Arctotraversodon plemmyridon Character = 76 Changed from ? to 2. Inferred.

Traversodon stahleckeri Character = 76 Changed from ? to 2 after von Huene 1936.

Dadadon isaloi Character = 76 Changed from ? to 2.

Cricodon metabolus Character = 76 Changed from ? to 2 after C. metabolus (NHCC LB28).

Bonacynodon schultzi Character = 76 Changed from ? to 2.

Protheriodon estudianti Character = 76 Changed from ? to 2.

Prozostrodon brasiliensis Character = 76 Changed from ? to 2.

Botucaraitherium belarminoi Character = 76 Changed from ? to 2.

Microconodon tenuirostris Character = 76 ROM 44300.

77. Dentary coronoid process height

0 below middle of orbit

1 above middle of orbit

Charassognathus gracilis Character = 77 Changed from ? to 0.

Procynosuchus delaharpeae Character = 77 RC 92.

Galesaurus planiceps Character = 77 BP/1/4602, BP/1/4714.

Thrinaxodon liorhinus Character = 77 BP/1/7199, BP/1/5905, BP/1/5208.

Cynognathus crateronotus Character = 77 BP/1/1181.

Diademodon tetragonus Character = 77 MB R1004, BP/1/4669.

Trirachodon spp. Character = 77 AM 461, BP/1/4658.

Langbergia modisei Character = 77 NMQR 3256.

Sinognathus gracilis Character = 77 IVPP V2339.

Scalenodon spp. Character = 77 Changed from 1 to ? Poor preservation and incomplete dentaries. No coronoid process is completely preserved.

Luangwa spp. Character = 77 BP/1/3731.

Mandagomphodon hirschsoni Character = 77 Definitively after what is preserved, a very high coronoid process.

Gomphodontosuchus brasiliensis Character = 77 Changed from 1 to ? Coronoid proceses incomplete in the holotype. Difficult to assess its dorsal extension.

Andescynodon mendozensis Character = 77 Changed from ? to 1.

Aleodon brachyramphus Character = 77 Chnaged from 1 to ?.

Aleodon cromptoni Character = 77 Changed from 1 to ?.

Lumkuia fuzzi Character = 77 Changed from 1 to 0.

Brasilodon tetragonus Character = 77 Changed from ? to 1.

Diarthrognathus broomi Character = 77 Changed from ? to 1.

Cricodon metabolus Character = 77 Inferred to be above the middle of the orbit in C. metabolus as observed in C. kannemeyeri. Changed from ? to 1.

Bonacynodon schultzi Character = 77 Changed from ? to 1.

Protheriodon estudianti Character = 77 Changed from ? to 0.

Prozostrodon brasiliensis Character = 77 Changed from ? to 1. Inferred.

78. Mediolateral thickening of anterior margin of coronoid process (Several non-mammalian cynodonts show some expansion of the anterior margin of the coronoid process, but this expansion is extreme in tritylodontids and some tritheledontids. This character was proposed by Hopson and Kitching (2001), character 50: coronoid mediolaterally thickening: 0, absent, 1, present and scored as present only in tritylodontids and tritheledontids. The character is considered here in the same way that Hopson and Kitching (2001).)

0 absent

1 present

Charassognathus gracilis Character = 78 Changed from ? to 0.

Procynosuchus delaharpeae Character = 78 RC 92, NHMUK PV37054 .

Scalenodon spp. Character = 78 UMCZ T.907.

Luangwa spp. Character = 78 NHMUK PV R 36995.

Mandagomphodon hirschsoni Character = 78 Hopson and Kitching (2001, character 50).

Gomphodontosuchus brasiliensis Character = 78 Changed from 0 to ? The left coronoid process is partially preserved but extremely deformed. The bone appears as exploded and give an appearance of thick structure to all the dentary.

Santacruzodon hopsoni Character = 78 Changed from ? to 1.

Aleodon brachyramphus Character = 78 Changed from 0 to ?.

Boreogomphodon jeffersoni Character = 78 Changed from ? to 1.

Arctotraversodon plemmyridon Character = 78 Changed from ? to 1.

Lumkuia fuzzi Character = 78 Changed from 0 to 1.

Traversodon stahleckeri Character = 78 Changed from ? to 1 after von Huene 1936.

Sinoconodon rigneyi Character = 78 I was unable to check this.

Bonacynodon schultzi Character = 78 Changed from ? to 1.

Protheriodon estudianti Character = 78 Changed from ? to 1.

Prozostrodon brasiliensis Character = 78 Changed from 1 to ?.

Botucaraitherium belarminoi Character = 78 Changed from 1 to ?.

79. Foramen on external surface of lower jaw between dentary and angular (Character introduced by Sidor and Smith (2004, character 41).)

0 absent

1 present

Procynosuchus delaharpeae Character = 79 RC 92, NHMUK PV37054 .

Galesaurus planiceps Character = 79 BP/1/5064.

Thrinaxodon liorhinus Character = 79 Changed from 0 to 1. BP/1/7199. MCZ 8892.

Platycraniellus elegans Character = 79 Changed from 0 to ? Preservation in TM 25 is not compete enough to score this character.

Cynognathus crateronotus Character = 79 UCMP 42749, PVL 3859.

Diademodon tetragonus Character = 79 BP/1/4669, MB R1004.

Trirachodon spp. Character = 79 BP/1/4658, BP/1/4661.

Langbergia modisei Character = 79 SAM-PK-K11481.

Sinognathus gracilis Character = 79 IVPP V2339.

Luangwa spp. Character = 79 BP/1/3731.

Andescynodon mendozensis Character = 79 Changed from ? to 0.

Brasilodon tetragonus Character = 79 Changed from ? to 0.

Diarthrognathus broomi Character = 79 Changed from ? to 0.

Dvinia prima Character = 79 Changed from ? to 1.

Cynosaurus suppostus Character = 79 Changed from ? to 0.

Progalesaurus lootbergensis Character = 79 Changed from ? to 0.

Nanictosaurus kitchingi Character = 79 Changed from ? to 0.

Cricodon metabolus Character = 79 Changed from ? to 0 after C. metabolus in Crompton, 1955. This foramen appears to be present in C. kannemeyeri according to Sidor and Hopson, 2018.

80. Angle of dentary

0 anterior to postorbital bar

1 at same level or slightly posterior

2 well to posterior

Procynosuchus delaharpeae Character = 80 RC 92.

Galesaurus planiceps Character = 80 BP/1/4602, BP/1/4506.

Thrinaxodon liorhinus Character = 80 BP/1/7199, BP/1/1375c, .

Platycraniellus elegans Character = 80 TM 25. tentative score.

Cynognathus crateronotus Character = 80 PVL 3859, NHMUK R2571.

Diademodon tetragonus Character = 80 BP/1/4669, NHMUK R2578.

Trirachodon spp. Character = 80 CGP 1/79, NMQR 3280, SAM-PK0K4801, but in SAM-PK-K5821 and BP/1/4661 the angle is located more posteriorly.

Langbergia modisei Character = 80 NMQR 3256.

Sinognathus gracilis Character = 80 IVPP V2339.

Luangwa spp. Character = 80 BP/1/3731.

Scalenodontoides macrodontes Character = 80 Changed from 2 to ? There is no articulated mandible to skull.

Santacruzodon hopsoni Character = 80 Changed from 1 to ?.

Aleodon cromptoni Character = 80 Changed from 1 to ?.

Chiniquodon spp. Character = 80 Changed from 1 to 2.

Pachygenelus monus Character = 80 The presence of a postorbital bar or a postorbital process is uncertain. The angle of the dentary is not 2 well posterior to the inferred position of the posterior end of the orbit.

Diarthrognathus broomi Character = 80 The presence of a postorbital bar or a postorbital process is uncertain. The angle of the dentary is not 2 well posterior to the inferred position of the posterior end of the orbit.

Sinoconodon rigneyi Character = 80 Changed from 1 to 2 . A postorbital bar or a postorbital process is absent. The angle of the dentary is 2 well posterior to the inferred position of the posterior end of the orbit.

Morganucodon spp. Character = 80 A postorbital bar or a postorbital process is absent.

Cricodon metabolus Character = 80 Inferred to be well posterior in C. metabolus as observed in C. kannemeyeri.

Tessellatia bonapartei Character = 80 The presence of a postorbital bar or a postorbital process is uncertain. The angle of the dentary is not 2 well posterior to the inferred position of the posterior end of the orbit. It is probably 1: at the same level or slightly posterior.

Bonacynodon schultzi Character = 80 Changed from ? to 1.

Protheriodon estudianti Character = 80 Changed from ? to 1. There is no postorbital bar. The posterior end of the orbit was considered by this scoring.

Prozostrodon brasiliensis Character = 80 Changed from ? to 1. Inferred.

81. Position of dentary surangular dorsal contact

0 closer to postorbital bar

1 midway

2 closer to jaw joint

Procynosuchus delaharpeae Character = 81 RC 92, BP/1/3748.

Galesaurus planiceps Character = 81 BP/1/4602.

Thrinaxodon liorhinus Character = 81 BP/1/7199.

Platycraniellus elegans Character = 81 TM 25. Tentative score because incomplete preservation of the dentary.

Cynognathus crateronotus Character = 81 BP/1/1181.

Diademodon tetragonus Character = 81 NHMUK R2578.

Trirachodon spp. Character = 81 CGP 1/79, NMQR 3280.

Langbergia modisei Character = 81 NMQR 3256.

Sinognathus gracilis Character = 81 IVPP V2339.

Scalenodon spp. Character = 81 Changed from 2 to ? There is no articulated mandible to the skull to check the character.

Luangwa spp. Character = 81 BP/1/3731.

Mandagomphodon hirschsoni Character = 81 Relevant area not preserved. Changed from 2 to ?.

Gomphodontosuchus brasiliensis Character = 81 Changed from 2 to ? by incomplete preservation of the mandible.

Pascualgnathus polanski Character = 81 Changed from 2 to ?.

Andescynodon mendozensis Character = 81 Changed from ? to 1.

Aleodon brachyramphus Character = 81 Changed from 2 to ?.

Aleodon cromptoni Character = 81 Changed from 2 to ?.

Diarthrognathus broomi Character = 81 Changed from ? to 1.

Nanictosaurus kitchingi Character = 81 Changed from 1 to 0.

Cricodon metabolus Character = 81 Changed from ? to 1. Inferred to be midway in C. metabolus as observed in C. kannemeyeri.

Bonacynodon schultzi Character = 81 Chandeg from 2 to 0.

Protheriodon estudianti Character = 81 Changed from ? to 1. There is no postorbital bar. The posterior end of the orbit was considered by this scoring.

Prozostrodon brasiliensis Character = 81 Changed from 2 to ?.

82. Reflected lamina of angular

0 corrugated plate

1 smooth plate with slight depressions

2 hook like laminae

3 thin projection

Procynosuchus delaharpeae Character = 82 AMNH FR 8220.

Galesaurus planiceps Character = 82 BP/1/4602.

Thrinaxodon liorhinus Character = 82 BP/1/7199.

Cynognathus crateronotus Character = 82 BSP 1934 VIII1, Broili and Schroder (1934, fig. 7).

Diademodon tetragonus Character = 82 NHMUK R2578.

Trirachodon spp. Character = 82 SAM-PK-K10157.

Langbergia modisei Character = 82 BP/1/5362.

Scalenodon spp. Character = 82 Changed from 2 to ? No preserved in any known specimen.

Luangwa spp. Character = 82 Changed from 2 to ? No preserved in any known specimen.

Mandagomphodon hirschsoni Character = 82 Relevant area not preserved. Changed from 2 to ?.

Gomphodontosuchus brasiliensis Character = 82 Changed from 2 to ? No preserved in the only known specimen.

Pascualgnathus polanski Character = 82 Changed from 2 to ?.

Aleodon brachyramphus Character = 82 Changed from 2 to ?.

Exaeretodon spp. Character = 82 Changed from ? to 3.

Nanictosaurus kitchingi Character = 82 Changed from ? to 1. See TM279.

Protheriodon estudianti Character = 82 Absent according to Bonaparte et al 2006.

83. Reflected lamina of angular posterior extent relative to distance from angle of dentary to jaw joint

0 greater than half the distance

1 less than half the distance

Charassognathus gracilis Character = 83 Changed from ? to 1.

Procynosuchus delaharpeae Character = 83 AMNH FR 8220: 19 of 33 mm (Liu and Olsen, 2010, appendix).

Galesaurus planiceps Character = 83 BP/1/4602.

Thrinaxodon liorhinus Character = 83 BP/1/7199.

Cynognathus crateronotus Character = 83 BSP 1934 VIII1, Broili and Schroder (1934, fig. 7).

Diademodon tetragonus Character = 83 NHMUK R2578.

Trirachodon spp. Character = 83 SAM-PK-K10157.

Langbergia modisei Character = 83 BP/1/5362.

Scalenodon spp. Character = 83 Changed from 1 to ? No preserved in any known specimen.

Luangwa spp. Character = 83 Changed from 1 to ? No preserved in any known specimen.

Mandagomphodon hirschsoni Character = 83 Relevant area not preserved. Changed from 1 to ?.

Gomphodontosuchus brasiliensis Character = 83 Changed from 1 to ? No preserved in the only known specimen.

Pascualgnathus polanski Character = 83 Changed from 1 to ?.

Aleodon brachyramphus Character = 83 Changed from 1 to ?.

Lumkuia fuzzi Character = 83 Inferred.

Ecteninion lunensis Character = 83 This is not observed, it is infered.

Dvinia prima Character = 83 Changed from 0 to ?.

84. Postdentary bones

0 large including tall surangular

1 angular surangular and prearticular medium in height and lying in dentary groove

2 single gracile rod in postdentary trough

Charassognathus gracilis Character = 84 From paper Botha et al 2007.

Procynosuchus delaharpeae Character = 84 RC 92, AMNH FR 2020.

Galesaurus planiceps Character = 84 BP/1/5064, NMQR 860.

Thrinaxodon liorhinus Character = 84 BP/1/7199.

Platycraniellus elegans Character = 84 TM 25.

Cynognathus crateronotus Character = 84 BP/1/1181.

Diademodon tetragonus Character = 84 NHMUK R2578.

Trirachodon spp. Character = 84 BP/1/4658.

Langbergia modisei Character = 84 BP/1/5362.

Sinognathus gracilis Character = 84 IVPP V2339.

Scalenodon spp. Character = 84 Changed from 1 to ? No completely preserved postdentary bar available to score. .

Luangwa spp. Character = 84 BP/1/3731.

Mandagomphodon hirschsoni Character = 84 The postdentary bones are not preserved, considering the development of the dentary trough medially we accept this score as tentative..

Aleodon cromptoni Character = 84 Chnaged from ? to 1.

Arctotraversodon plemmyridon Character = 84 Changed from ? to 1.

Lumkuia fuzzi Character = 84 Changed from 0 to 1.

Traversodon stahleckeri Character = 84 Changed from ? to 1 after von Huene 1936 (inferred).

Chaliminia musteloides Character = 84 Changed from ? to 2 after Martinelli and Rougier 2007.

Riograndia guaibensis Character = 84 Changed from 1 to 2.

Dadadon isaloi Character = 84 This is probably 1 but the postdentary bones ar not preserved.

Menadon besairiei Character = 84 This is probably 1 but the postdentary bones are not preserved.

Cricodon metabolus Character = 84 After C. metabolus UMCZ T905.

Botucaraitherium belarminoi Character = 84 Probably 1 or 2. .

85. Craniomandibular articulation

0 quadrate articular

1 main quadrate articular, secondary surangular squamosal

2 quadrate articular by an extensive reduction of surangular

3 main dentary squamosal

Charassognathus gracilis Character = 85 Changed from ? to 0.

Procynosuchus delaharpeae Character = 85 AMNH FR 8220, RC 92.

Galesaurus planiceps Character = 85 SAM-PK-K9956, BP/1/5064.

Thrinaxodon liorhinus Character = 85 BP/1/7199, UCMP 40466.

Platycraniellus elegans Character = 85 TM 25. Tentative score, mandibular articulation is out of place and it is not possible to be sure if it contacted the squamosal flange.

Cynognathus crateronotus Character = 85 BP/1/1181.

Diademodon tetragonus Character = 85 BP/1/4669, BP/1/3639.

Trirachodon spp. Character = 85 SAM-PK-K5821.

Langbergia modisei Character = 85 NMQR 3256.

Sinognathus gracilis Character = 85 IVPP V2339.

Scalenodon spp. Character = 85 Changed from 1 to ? No quadrate preserved articulated with the mandible.

Luangwa spp. Character = 85 BP/1/3731.

Mandagomphodon hirschsoni Character = 85 This is tricky. Hopson and Kitching (2001, character 25) scored descending flange of the squamosal lateral to quadratojugal present contacting surangular (2). The surangular is not preserved and we dont know if there was a lateral projection in this bone to contact the squamosal flange. In addition, the later is not complete (according to Hopson, 2014: fig. 14.3b). Considering this we opted to change the score from (0 and 1) to ?.

Scalenodontoides macrodontes Character = 85 Changed from 1 to ? Not preserved area in the available specimens.

Pascualgnathus polanski Character = 85 Changed from 0//1 to ?.

Brasilodon tetragonus Character = 85 En *Brasilodon tetragonus*/brasilitherium, habría que agregar un estado nuevo, que indique Main quadrate articular, secondary dentary squamosal.

Chaliminia musteloides Character = 85 Changed from 3 to 1. It is probably main quadrate-articular and secondary dentary-squamosal.

Riograndia guaibensis Character = 85 Changed from 3 to 2.

Dadadon isaloi Character = 85 It appears to be 1 but it is not described.

Pachygenelus monus Character = 85 Changed from 3 to 1.

Diarthrognathus broomi Character = 85 Changed from 3 to 1.

Dvinia prima Character = 85 Changed from 1 to 0. This is following Konjukova (1949), but Tatarinov (1968: 33) said ?The mandibular articulation of *Dvinia prima* is unusually complicated and appears to have been double (Tatarinov, 1966b). The surangular, which is thickened posteriorly, articulated with the exterior edge of the quadratojugal (figs. 5B, 6B: al). The quadratojugal at this point forms a falciform projection, which touches for a limited distance the inner surface of the surangular (Fig. 6, pf). Details of this articulation are unclear because of the condition of preservation, but there is little doubt of its

existence?. The observation of the cast and photos of the material cast doubt in the presence of the second contact proposed by Tatarinov (1968).

Cricodon metabolus Character = 85 Changed from ? to 0 after Crompton 1955.

86. Craniomandibular articulation

0 at same height as postcanine line

1 higher than postcanine line

2 much lower than postcanine line

Charassognathus gracilis Character = 86 Changed from ? to 0.

Procynosuchus delaharpeae Character = 86 AMNH FR 8220, RC 92.

Galesaurus planiceps Character = 86 SAM-PK-K9956.

Thrinaxodon liorhinus Character = 86 BP/1/7199.

Platycraniellus elegans Character = 86 TM 25.

Cynognathus crateronotus Character = 86 BP/1/1181.

Diademodon tetragonus Character = 86 BP/1/4669.

Trirachodon spp. Character = 86 SAM-PK-K5821.

Langbergia modisei Character = 86 NMQR 3256.

Sinognathus gracilis Character = 86 IVPP V2339.

Scalenodon spp. Character = 86 UMZC T907. Inferred after the putative position of the quadrate in the skull.

Luangwa spp. Character = 86 BP/1/3731.

Gomphodontosuchus brasiliensis Character = 86 Changed from o to ? Not preserved area in the only known specimen. .

Scalenodontoides macrodontes Character = 86 Inferred after the putative position of the quadrate in the skull of BP/1/5395.

Andescynodon mendozensis Character = 86 Changed from ? to 0.

Traversodon stahleckeri Character = 86 Changed from ? to 0 after Barberena 1981.

Dadadon isaloi Character = 86 Changed from ? to 0.

Menadon besairiei Character = 86 Changed from ? to 0.

Cricodon metabolus Character = 86 Changed from ? to 0.

Prozostrodon brasiliensis Character = 86 Changed from ? to 0. Inferred.

Microconodon tenuirostris Character = 86 USNM 437637.

87. Upper tooth series extension

0 anterior to orbit

1 below orbit

2 posterior to anterior border of subtemporal fenestra

Procynosuchus delaharpeae Character = 87 RC 92, BP/1/3748.

Galesaurus planiceps Character = 87 BP/1/5064.

Thrinaxodon liorhinus Character = 87 BP/1/7199.

Platycraniellus elegans Character = 87 Changed from 1 to 0 (after Abdala, 2007): six or seven postcanines in the upper dentition, which does not extend as far as the level of the orbits (Text-fig. 2B).

Cynognathus crateronotus Character = 87 BP/1/1181.

Diademodon tetragonus Character = 87 MB R1004.

Trirachodon spp. Character = 87 BP/1/4658, BP/1/4661.

Langbergia modisei Character = 87 BP/1/3751.

Sinognathus gracilis Character = 87 IVPP V2339.

Beishanodon youngi Character = 87 Consideirng figures 4A and 4B of Gao et al. (2010) the postcanines extends below the orbits. This is scored contra Gao et al (2010: 14): In contrast to *Sinognathus gracilis* gracilis,the upper dentition of PKUP V3007 ends at a level beneath the anterior border of the orbit,.

Scalenodon spp. Character = 87 UMZC T907.

Luangwa spp. Character = 87 BP/1/3731.

Mandagomphodon hirschsoni Character = 87 Hopson (2014: fig. 14.1).

Mandagomphodon attridgei Character = 87 Changed from 0 to 1, clearly postcanines extends below the orbit. .

Gomphodontosuchus brasiliensis Character = 87 Hopson (1984). GPIT/RE/09397.

Scalenodontoides macrodontes Character = 87 BP/1/5395.

Andescynodon mendozensis Character = 87 Changed from ? to 1.

Santacruzodon hopsoni Character = 87 Changed from 2 to 1.

Aleodon brachyramphus Character = 87 Changed from ? to 1.

Boreogomphodon jeffersoni Character = 87 Changed from ? to 1.

Menadon besairiei Character = 87 Changed from ? to 0.

Exaeretodon spp. Character = 87 Changed from 2 to 1.

Elliotherium kersteni Character = 87 Changed from ? to 2.

Pachygenelus monus Character = 87 Changed from 1 to 2.

Sinoconodon rigneyi Character = 87 Changed from 1 to 2.

Cricodon metabolus Character = 87 Changed from 0 to 1.

Protheriodon estudianti Character = 87 Changed from 1 to 0.

88. Upper incisors

0 more than four

1 four

2 fewer than four

Procynosuchus delaharpeae Character = 88 NHMUK PV37054, BP/1/3748 .

Galesaurus planiceps Character = 88 SAM-PK-K1119.

Thrinaxodon liorhinus Character = 88 BP/1/7199, TM 80.

Platycraniellus elegans Character = 88 TM 25.

Cynognathus crateronotus Character = 88 PVL 3859, BP/1/1181.

Diademodon tetragonus Character = 88 MB R1004.

Trirachodon spp. Character = 88 BP/1/4658.

Langbergia modisei Character = 88 NMQR 3255.

Sinognathus gracilis Character = 88 IVPP V2339.

Beishanodon youngi Character = 88 Gao et al. (2010: fig. 4B).

Scalenodon spp. Character = 88 UMZC T916.

Luangwa spp. Character = 88 BP/1/3731.

Mandagomphodon hirschsoni Character = 88 Three large incisors (Hopson, 2014: fig. 14.1).

Mandagomphodon attridgei Character = 88 Three upper incisors.

Gomphodontosuchus brasiliensis Character = 88 GPIT/RE/09397.

Scalenodontoides macrodontes Character = 88 BP/1/5395.

Boreogomphodon jeffersoni Character = 88 Changed from ? to 1.

Diarthrognathus broomi Character = 88 Changed from 2 to ?.

Cricodon metabolus Character = 88 Changed from 0 to 1 after Sidor and Hopson, 2018 (*C. metabolus* - NHCC LB28).

Protheriodon estudianti Character = 88 Changed from ? to 0. .

89. Lower incisors

0 four or more

1 three

2 fewer than three

Procynosuchus delaharpeae Character = 89 NHMUK PV37054, RC 304.

Galesaurus planiceps Character = 89 SAM-PK-K1119.

Thrinaxodon liorhinus Character = 89 BP/1/7199.

Cynognathus crateronotus Character = 89 BSP 1934 VIII1.

Diademodon tetragonus Character = 89 MB R1004.

Trirachodon spp. Character = 89 BP/1/4658.

Langbergia modisei Character = 89 NMQR 3251.

Sinognathus gracilis Character = 89 Sun (1988, 180).

Scalenodon spp. Character = 89 UMZC T910.

Luangwa spp. Character = 89 NHMUK PV R 36995.

Mandagomphodon hirschsoni Character = 89 Two large lower incisors (Hopson, 2014: fig. 14.4).

Gomphodontosuchus brasiliensis Character = 89 GPIT/RE/09397.

Scalenodontoides macrodontes Character = 89 MNHN 1957-23.

Santacruzodon hopsoni Character = 89 Changed from ? to 1.

Boreogomphodon jeffersoni Character = 89 Changed from 2 to 1.

Arctotraversodon plemmyridon Character = 89 Changed from ? to 1.

Traversodon stahleckeri Character = 89 Changed from ? to 1 after von Huene 1936.

Brasilodon tetragonus Character = 89 Changed from 0 to 1 (Agustin).

Dadadon isaloi Character = 89 Changed from ? to 1.

Menadon besairiei Character = 89 Changed from ? to 1.

Ecteninion lunensis Character = 89 Changed from 1 to ?. This is not observable.

Kayentatherium wellesi Character = 89 changed from 1 to 2.

Bienotherium spp. Character = 89 Changed from 1 to 2.

Diarthrognathus broomi Character = 89 Changed from 2 to ?.

Cricodon metabolus Character = 89 Changed from ? to 1 (three incisors) after Crompton 1955 and Sidor and Hopson 2018.

90. Incisors

0 all small

1 some or all enlarged

Charassognathus gracilis Character = 90 Changed from ? to 0.

Procynosuchus delaharpeae Character = 90 RC 92, NHMUK PV37054.

Galesaurus planiceps Character = 90 SAM-PK-K1119.

Thrinaxodon liorhinus Character = 90 BP/1/7199, NHMUK R511.

Platycraniellus elegans Character = 90 TM 25.

Cynognathus crateronotus Character = 90 BSP 1954 VIII2.

Trirachodon spp. Character = 90 BP/1/4658.

Langbergia modisei Character = 90 NMQR 3256.

Sinognathus gracilis Character = 90 IVPP V2339.

Scalenodon spp. Character = 90 UMZC T918.

Luangwa spp. Character = 90 BP/1/3731.

Mandagomphodon hirschsoni Character = 90 Incisors are definitively large elements.

Mandagomphodon attridgei Character = 90 Incisors appears to be relatively smaller than canine.

Gomphodontosuchus brasiliensis Character = 90 GPIT/RE/09397. Lower incisors as large as canines. Tentative score because the condition in the uppers seems to be different.

Scalenodontoides macrodontes Character = 90 MNHN 1957-23.

Protuberum cabralense Character = 90 Changed from 1 to 0. .

Boreogomphodon jeffersoni Character = 90 Changed from 1 to 0.

Arctotraversodon plemmyridon Character = 90 Changed from ? to 0.

Menadon besairiei Character = 90 Changed from 1 to 0.

Exaeretodon spp. Character = 90 Changed from 1 to 0.

Diarthrognathus broomi Character = 90 Changed from 1 to ?.

Tessellatia bonapartei Character = 90 Si consideramos el pedacito interpretado como i1 esto debería ser (0) all small. Pero es crieto que es dudoso, lo paso a ?.

91. Incisor cutting margins

0 serrated

1 smoothly ridged

2 denticulated

Procynosuchus delaharpeae Character = 91 RC 92, NHMUK PV37054.

Galesaurus planiceps Character = 91 SAM-PK-K1119.

Thrinaxodon liorhinus Character = 91 BP/1/7199, NHMUK R511.

Cynognathus crateronotus Character = 91 Changed from 0 to 1. We could not find incisors with serrations in the available material (contra Abdala et al., 2006, character 34).

Diademodon tetragonus Character = 91 Abdala et al. (2006, 411): Hopson & Kitching (2001: character 55) scored a question mark for Diademodon, but a serrated margin of incisors is present in BS1934 VIII14.

Trirachodon spp. Character = 91 Changed from 0 to 1 following Abdala et al. (2006, character 34).

Langbergia modisei Character = 91 Abdala et al (2006, 395): They are (the incisors) slightly recurved and circular in cross-section, with no ridges or serrated edges.

Sinognathus gracilis Character = 91 IVPP V2339.

Scalenodon spp. Character = 91 UMZC T910.

Luangwa spp. Character = 91 Abdala and Teixeira (2004, 16): The crown of the left third incisor is partially preserved, being canine-like, with a thick enamel layer labially that seems to be absent lingually. Faint denticulations are present on the posterior edge of this tooth, while the anterior edge appears to be smooth. .

Mandagomphodon hirschsoni Character = 91 Hopson (2014).

Mandagomphodon attridgei Character = 91 NHMUK R8578 .

Gomphodontosuchus brasiliensis Character = 91 GPIT/RE/09397.

Scalenodontoides macrodontes Character = 91 MNHN 1957-23.

Boreogomphodon jeffersoni Character = 91 Changed from 0 to 1.

Arctotraversodon plemmyridon Character = 91 Changed from 1 to 2.

Cricodon metabolus Character = 91 I could not check this scoring. Serrations are described for upper incisors by Sidor and Hopson, 2018.

Pseudotherium argentinus Character = 91 Changed from 1 to ?. Incisors unknown.

92. Incisor occlusion

0 teeth relatively evenly placed and sized (This is character 3 of Martinez et al. (1996) and character 81 of Abdala (2007). The state 1 is scored for tritylodontid and tritheledontid (tentatively in Pachygenelus). The state 1 is more likely after Hopson (1991, 677): the following are tritylodontid-tritheledontid characters not found in mammals: 1) the first lower incisor enlarged., 2) the tip of the lower incisor fitting into the gap between laterally placed upper incisors?)

1 first lower incisor enlarged and fits into a gap between first upper incisors

Charassognathus gracilis Character = 92 Changed from ? to 0.

Procynosuchus delaharpeae Character = 92 NHMUK PV37054.

Beishanodon youngi Character = 92 Considering the incisor alveoli is possible to infer this condition even when the teeth are not preserved.

Mandagomphodon attridgei Character = 92 NHMUK R8578 .

Scalenodontoides macrodontes Character = 92 BP/1/5395. Gow and Hancox (1993, fig. 4).

Pascualgnathus polanski Character = 92 Changed from ? to 0.

Andescynodon mendozensis Character = 92 Changed from ? to 0.

Santacruzodon hopsoni Character = 92 Changed from ? to 0.

Protuberum cabralense Character = 92 Changed from ? to 0. .

Aleodon brachyramphus Character = 92 Changed from ? to 0.

Arctotraversodon plemmyridon Character = 92 Changed from ? to 0.

Brasilodon tetragonus Character = 92 Changed from ? to 0.

Dadadon isaloi Character = 92 Changed from ? to 0.

Menadon besairiei Character = 92 Changed from ? to 0.

Bienotherium spp. Character = 92 Changed from ? to 1.

Cricodon metabolus Character = 92 Changed from ? to 0.

Tessellatia bonapartei Character = 92 Si consideramos el pedacito interpretado como i1 esto debería ser (0) teeth relatively evenly placed and sized. Pero es cierto que es dudoso, lo paso a ?.

Bonacynodon schultzi Character = 92 Changed from ? to 0.

Protheriodon estudianti Character = 92 Changed from ? to 0.

Prozostrodon brasiliensis Character = 92 Changed from ? to 0.

93. Upper incisor canine diastema (This character is referred to upper dentition, we rephrase it adding the word 'upper' to the original definition.)

0 present

1 absent

Character = 93 This character is referred to upper dentition, we rephrase it adding the word upper to the original definition.

Procynosuchus delaharpeae Character = 93 Score changed to -, because the presence of precanines (next character), imply that we cannot score the diastema between canine and incisor.

Galesaurus planiceps Character = 93 Changed from 0 to 1 (Liu and Olsen, 2010, character 98, contra Abdala, 2007, character 82) SAM-PK-K1119, NMQR 135. Jasinoski and Abdala (2017, fig. 6).

Thrinaxodon liorhinus Character = 93 BP/1/7199, NHMUK R511.

Platycraniellus elegans Character = 93 TM 25.

Cynognathus crateronotus Character = 93 BP/1/1181, BSP 1934 VIII2.

Diademodon tetragonus Character = 93 BSP 1934 VIII14.

Trirachodon spp. Character = 93 BP/1/4658.

Langbergia modisei Character = 93 NMQR 3255.

Sinognathus gracilis Character = 93 IVPP V2339.

Beishanodon youngi Character = 93 The paracanine fossa is located in the diastema between the last incisor and the canine.

Scalenodon spp. Character = 93 UMZC T907.

Luangwa spp. Character = 93 BP/1/3731.

Mandagomphodon hirschsoni Character = 93 Hopson (2014: fig. 14.1b). The paracanine fossa is placed between the last incisor and canine.

Mandagomphodon attridgei Character = 93 A small diastema is present were it is placed the paracanine fossa.

Gomphodontosuchus brasiliensis Character = 93 GPIT/RE/09397.

Scalenodontoides macrodontes Character = 93 Gow and Hancox (1993, fig. 4).

Boreogomphodon jeffersoni Character = 93 Changed from ? to 1.

Lumkuia fuzzi Character = 93 Changed from 0 to 1.

Brasilodon tetragonus Character = 93 Changed from ? to 0.

Dvinia prima Character = 93 Changed from 1 to 0.

94. Pre canine maxillary teeth (This is an incisor-like tooth in the maxilla (in front of the canine), only recognized in a few basal cynodonts such as *Procynosuchus delaharpeae* and *Dvinia prima*. It is also observed in different lineages of therocephalians.)

0 absent

1 present

Procynosuchus delaharpeae Character = 94 RC 92, NHMUK PV37054.

Mandagomphodon attridgei Character = 94 NHMUK R8578 .

Pascualgnathus polanski Character = 94 Changed from ? to 0.

Protuberum cabralense Character = 94 Changed from ? to 0. .

Boreogomphodon jeffersoni Character = 94 Changed from ? to 0.

Brasilodon tetragonus Character = 94 changed from present to absent after Bonaparte (2013), based in figures of UFRGS PV 1043 assigned to Brasilitherium.

Chaliminia musteloides Character = 94 There is doubt regarding the presence of the I4 in the maxilla.

Dadadon isaloi Character = 94 Changed from ? to 0.

Bonacynodon schultzi Character = 94 Changed from ? to 0.

Protheriodon estudianti Character = 94 Changed from 1 to 0 after Martinelli et al (2016): "Consequently, *Protheriodon estudianti* has at least four (and perhaps one more) upper and four lower incisors". The score is tentative as the preservation does not allow for a clear location of the upper incisors. Considering that most cynodonts does not feature precanines we opted to score as absent in this taxon too.

Prozostrodon brasiliensis Character = 94 Changed from ? to 0.

Pseudotherium argentinus Character = 94 Changed from ? to 0. .

95. Upper canine

0 large

1 reduced

2 absent

Procynosuchus delaharpeae Character = 95 BP/1/3748, .

Galesaurus planiceps Character = 95 SAM-PK-K1119, NMQR 1451.

Thrinaxodon liorhinus Character = 95 BP/1/7199, NHMUK R511, BP/1/2824.

Platycraniellus elegans Character = 95 TM 25.

Cynognathus crateronotus Character = 95 BP/1/1181.

Diademodon tetragonus Character = 95 MB R1004.

Trirachodon spp. Character = 95 BP/1/4658.

Langbergia modisei Character = 95 NMQR 3256.

Sinognathus gracilis Character = 95 IVPP V2339.

Beishanodon youngi Character = 95 Inferred after the size of the alveoli.

Scalenodon spp. Character = 95 UMZC T907.

Luangwa spp. Character = 95 BP/1/3731.

Mandagomphodon hirschsoni Character = 95 Upper canines seems to be same size than upper incisors (Hopson, 2014: fig. 14.1).

Mandagomphodon attridgei Character = 95 Canines are broken near their bases. They seems to be larger than the incisors, but is not possible to be certain if they are or no enlarged. Changed from 0 to ? .

Gomphodontosuchus brasiliensis Character = 95 GPIT/RE/09397. Canine seems to be only slightly larger than incisors. Tentative score.

Scalenodontoides macrodontes Character = 95 BP/1/5395 (Gow and Hancox, 1993, fig. 4), inferred by alveolar size.

Santacruzodon hopsoni Character = 95 Changed from ? to 1.

Brasilodon tetragonus Character = 95 Changed from 1 to 0 (Agustin).

Riograndia guaibensis Character = 95 Contra Soares et al 2011 (they say it is reduced, but see figures).

Massetognathus spp. Character = 95 I am not sure about this. Why reduced? It is notably higher than the incisors and postcanines. We should be more specific in the states of this character. Changed from 1 to 0 (see for example PVL 4448).

Dadadon isaloi Character = 95 Changed from ? to 1 to 0. See Kammerer et al 2012.

Botucaraitherium belarminoi Character = 95 Changed from ? to 0.

Pseudotherium argentinus Character = 95 Changed from ? to 0. .

96. Lower canine

0 large

1 reduced

2 absent

Procynosuchus delaharpeae Character = 96 NHMUK PV37054.

Galesaurus planiceps Character = 96 SAM-PK-K1119.

Thrinaxodon liorhinus Character = 96 BP/1/7199, TM 80.

Platycraniellus elegans Character = 96 TM 25.

Cynognathus crateronotus Character = 96 BSP 1934 VIII1.

Diademodon tetragonus Character = 96 NHMUK R3588, MB R1004.

Trirachodon spp. Character = 96 BP/1/4658.

Langbergia modisei Character = 96 NMQR 3251.

Sinognathus gracilis Character = 96 IVPP V2339.

Scalenodon spp. Character = 96 UMZC T907, UMZC T909. It is inferred by difference in canine alveolus size in comparison with those of incisors. Tentative score.

Luangwa spp. Character = 96 In NHMUK PV R 36995 there is a large difference in the alveoli of the canine and those of the incisors. In MCP 3167PV the canine appears to be only slightly larger than incisors. However this could be the erupting canine as in the upper jaw it is clear than both canines are erupting. We score as large but it is tentative.

Mandagomphodon hirschsoni Character = 96 Lower canines seems to be of the same size than incisors (Hopson, 2014: fig. 14.4). Score changed from 0 to 1.

Gomphodontosuchus brasiliensis Character = 96 GPIT/RE/09397 .

Scalenodontoides macrodontes Character = 96 MNHN 1957-23. Same size than incisors.

Santacruzodon hopsoni Character = 96 Changed from ? to 1.

Traversodon stahleckeri Character = 96 Inferred because of the presence of a paracanine fossa.

Massetognathus spp. Character = 96 Changed from 1 to 0 (see for example PVL 4726).

Dadadon isaloi Character = 96 Changed from ? to 0. See Ravinohamanana et al 2011 and Kammerer et al., 2012.

Ecteninion lunensis Character = 96 Chnged fro 0 to ? as this is not observable.

Sinoconodon rigneyi Character = 96 Changed from 1 to 0.

Bonacynodon schultzi Character = 96 Changed from ? to 0. It is inferred from the alvolus size.

Protheriodon estudianti Character = 96 Changed from ? to 1. .

Microconodon tenuirostris Character = 96 hanged from 1 to 0 after Sues 2001.

97. Canine serrations

0 present

1 absent

Procynosuchus delaharpeae Character = 97 NHMUK PV37054.

Galesaurus planiceps Character = 97 BP/1/4602, SAM-PK-K1119.

Thrinaxodon liorhinus Character = 97 BP/1/7199, TM 80.

Platycraniellus elegans Character = 97 TM 25.

Cynognathus crateronotus Character = 97 BP/1/1181.

Diademodon tetragonus Character = 97 USNM 23352, BP/1/4669.

Trirachodon spp. Character = 97 BP/1/4658.

Langbergia modisei Character = 97 NMQR 3251.

Sinognathus gracilis Character = 97 IVPP V2339.

Scalenodon spp. Character = 97 UMZC T910.

Luangwa spp. Character = 97 BP/1/3731.

Mandagomphodon hirschsoni Character = 97 For upper canines Hopson (2014: 243) mention distal cutting ridge without serrations.

Mandagomphodon attridgei Character = 97 No serration visible in the small portion preserved of the upper canines.

Gomphodontosuchus brasiliensis Character = 97 GPIT/RE/09397.

Scalenodontoides macrodontes Character = 97 MNHN 1957-23.

Protuberum cabralense Character = 97 Changed from ? to 1. .

Boreogomphodon jeffersoni Character = 97 Changed from ? to 1.

Arctotraversodon plemmyridon Character = 97 Changed from ? to 1.

Cricodon metabolus Character = 97 Serrations are described for upper canines by Sidor and Hopson, 2018.

Prozostrodon brasiliensis Character = 97 Changed from 0 to ?.

98. Axis of posterior part of maxillary tooth row

0 directed lateral to subtemporal fossa

1 directed toward centre of fossa

2 directed toward medial rim of fossa and curved

3 directed toward medial rim of fossa and parallel

Procynosuchus delaharpeae Character = 98 NHMUK PV37054, RC 5.

Galesaurus planiceps Character = 98 NMQR135, SAM-PK-K1119.

Thrinaxodon liorhinus Character = 98 BP/1/7199, TM 80.

Platycraniellus elegans Character = 98 TM 25.

Cynognathus crateronotus Character = 98 BSP 1934 VIII1.

Diademodon tetragonus Character = 98 BSP 1934 VIII14, MB R1004.

Trirachodon spp. Character = 98 BP/1/4658.

Langbergia modisei Character = 98 NMQR 3255.

Sinognathus gracilis Character = 98 IVPP V2339.

Beishanodon youngi Character = 98 Gao et al. (2010: figs. 4B, 5).

Scalenodon spp. Character = 98 UMZC T907.

Luangwa spp. Character = 98 BP/1/3731.

Mandagomphodon hirschsoni Character = 98 Hopson (2014: fig. 14.1b). In the specimen the axis seems to be solightly more curved than the illustrated by Hopson (2014).

Mandagomphodon attridgei Character = 98 NHMUK R8578 .

Gomphodontosuchus brasiliensis Character = 98 GPIT/RE/09397.

Scalenodontoides macrodontes Character = 98 BP/1/5395.

Santacruzodon hopsoni Character = 98 Changed from 2 to ?^n^n.

Therioherpeton cargnini Character = 98 Changed from 0 to 1.

Boreogomphodon jeffersoni Character = 98 Changed from ? to 1.

Chaliminia musteloides Character = 98 I am not sure about this scoring.

Exaeretodon spp. Character = 98 Changed from 2 to 1 (e.g., MACN 18125 and holotype *E. riograndensis*).

Trucidocynodon riograndensis Character = 98 Changed from 0 to 1. I am not sure about this character.

Diarthrognathus broomi Character = 98 Changed from 3 to ?.

Sinoconodon rigneyi Character = 98 Changed from 1 to 0.

Progalesaurus lootbergensis Character = 98 Changed from 0 to ?.

Nanictosaurus kitchingi Character = 98 Changed from 0 to ?.

Cricodon metabolus Character = 98 Changed from ? to 0 after Crompton, 1955.

Tessellatia bonapartei Character = 98 This is scored considered the inferred position of the subtemporal fossa.

Bonacynodon schultzi Character = 98 Changed from 1 to ?.

Protheriodon estudianti Character = 98 Changed from 1 to 0 after Bonaparte et al 2006 fig 2.

99. Postcanine occlusion (Rowe (1988) character 84: postcanines- bilateral (0) or unilateral (1) postcanine occlusion in adults. Bilateral was scored in outgroup, *Exaeretodon* spp., tritylodontids and multituberculata, whereas unilateral was scored in morganucodontidae, marsupialia and placentalia. It was considered as non-comparable in Monotremata. Character unchanged by Wible (1991, his character 33), but he scored 0 in *Sinoconodon rigneyi* and ? for multituberculata, whereas remaining mammaliafomes (including monotremata) are considered as having unilateral occlusion. Crompton and Luo (1993; table 4.2) provide the following wording and distribution: unilateral occlusion without consistent positional relationships between the upper and lower postcanines is an apomorphy of tritheledontids and Liassic mammaliaforms. Precise unilateral occlusion and consistent positional relationships between the upper and lower molars is apomorphic for (*Megazostrodon* (*Morganucodon* spp., *Dinnetherium*) *Kuehneotherium*)) For *Morganucodon* spp. and *Dinnetherium*, cusp a occludes between cusps B and A. For *Kuehneotherium*, dual shearing surface between A+C, a+b. For tritylodontids, occlusion of the upper and lower postcanines by eruption and bilateral occlusion. Luo (1994), discriminate one character (character 1) as mode of occlusion: bilateral (0), unilateral (1) and a very complex character 17: relationships of wear facets to main cusps: wear facet absent (0), simple longitudinal facet that extends entire length of crown (1), principal cusp

bears two longitudinal wear facets (cusp a of lower tooth bears two facets, which either contact facets of cusp B of opposing tooth and cusp C of the preceding tooth or contact facets A and B of opposing tooth) (2), single facet supported by two cusps (cusps a and c of lower tooth contact the facet supported by cusps A and B of opposing tooth; single facet supported by cusps a and b contacts facet supported by cusps A and C of preceding tooth) (3), multiple cusps, with each cusp bearing one or two transverse facets (4). The scoring of character 1 is bilateral for most non mammaliaform cynodonts (including gomphodonts and tritylodontids) and unilateral in tritheledontids, and all mammaliaforms (including *Sinoconodon rigneyi*). For character 17 wear facets absent in thrinaxodontidae, Probainognathidae and *Sinoconodon rigneyi*; state 1 only in tritheledontidae, state 2 for all mamaliaformes except Kuehneotheridae which is scored 3 and gomphodonts and tritylodontids are scored 4. *Dinnetherium* is scored as 2, but in the figure of Crompton and Luo (1993, fig. 4.5c) only one wear facet is represented in the mean cusp a of the lower tooth. There are two wear facets but one on cusp a and c and the other on cusp b. Our wording of the character follows Abdala (2007, character 88): Postcanine occlusion: (0) absent; (1) unilateral without forming a consistent pattern between upper and lower teeth; (2) precise unilateral occlusion; (3) tooth to tooth contact because of widened postcanines. State 0 is scored in most basal cynodonts and also in several probainognathians. Including *Probainognathus jenseni* in which there is clear wear of the postcanine dentition but no occlusal surfaces (Romer, 1970). State 3 is scored in cynodonts with widened postcanine teeth (and bilateral occlusion) and will include *Abdalodon*, *Dvinia prima*, gomphodonts and tritylodontids. State 1 is for tritheledontids and state 2 for mammaliaforms with occlusion (all except *Sinoconodon rigneyi*).

0 absent

1 unilateral without forming a consistent pattern between upper and lower teeth

2 precise unilateral occlusion

3 tooth to tooth contact because of widened postcanines

Charassognathus gracilis Character = 99 Changed from ? to 0.

Procynosuchus delaharpeae Character = 99 No facets observed in any postcanine. See also Crompton (1972: 32).

Platycraniellus elegans Character = 99 inferred because morphology of postcanines.

Beishanodon youngi Character = 99 The postcanines are less expanded than typical gomphodont teeth, but is possible to infer the possible contact between upper and lower postcanines. Tentative score.

Scalenodon spp. Character = 99 Gomphodont postcanines.

Luangwa spp. Character = 99 Gomphodont postcanines.

Nanogomphodon wildi Character = 99 Gomphodont postcanines.

Mandagomphodon hirschsoni Character = 99 Gomphodont postcanines.

Mandagomphodon attridgei Character = 99 Gomphodont postcanines.

Gomphodontosuchus brasiliensis Character = 99 Gomphodont postcanines.

Scalenodontoides macrodontes Character = 99 Gomphodont postcanines.

Andescynodon mendozensis Character = 99 Changed from ? to 3.

Santacruzodon hopsoni Character = 99 Changed from ? to 3.

Protuberum cabralense Character = 99 Changed from ? to 3. .

Therioherpeton cargnini Character = 99 Changed from ? to 1.

Boreogomphodon jeffersoni Character = 99 Changed from ? to 3.

Arctotraversodon plemmyridon Character = 99 Changed from ? to 3.

Traversodon stahleckeri Character = 99 Changed from ? to 3.

Chaliminia musteloides Character = 99 Changed from ? to 1.

Riograndia guaibensis Character = 99 Changed from ? to 1.

Dadadon isaloi Character = 99 Changed from ? to 3.

Menadon besairiei Character = 99 Changed from ? to 3.

Trucidocynodon riograndensis Character = 99 I think this should be changed to 1 because there are wear facets.

Elliotherium kersteni Character = 99 Changed from ? to 1.

Sinoconodon rigneyi Character = 99 Changed from 0 to 1.

Bonacynodon schultzi Character = 99 Changed from 1 to 0.

Protheriodon estudianti Character = 99 1 or 2.

Prozostrodon brasiliensis Character = 99 Changed from 1 to 0.

Pseudotherium argentinus Character = 99 Changed from 1 to 0.

100. Postcanines (The state 1 is recognized only in some Mammaliaformes.)

0 undifferentiated

1 differentiated into premolariforms and molariforms

Procynosuchus delaharpeae Character = 100 RC 92, BP/1/3748.

Langbergia modisei Character = 100 Changed from ? to 0.

Beishanodon youngi Character = 100 Gao et al. (2010: fig.6).

Nanogomphodon wildi Character = 100 Changed from ? to 0.

Mandagomphodon attridgei Character = 100 Changed from ? to 0.

Gomphodontosuchus brasiliensis Character = 100 Changed from ? to 0.

Pascualgnathus polanski Character = 100 Changed from ? to 0.

Andescynodon mendozensis Character = 100 Changed from ? to 0.

Protuberum cabralense Character = 100 Changed from ? to 0.

Boreogomphodon jeffersoni Character = 100 Changed from ? to 0.

Arctotraversodon plemmyridon Character = 100 Changed from ? to 0.

Traversodon stahleckeri Character = 100 Changed from ? to 0.

Dadadon isaloi Character = 100 Changed from ? to 0.

Menadon besairiei Character = 100 Changed from ? to 0.

Elliotherium kersteni Character = 100 Changed from ? to 0.

Bonacynodon schultzi Character = 100 Changed from ? to 0.

Protheriodon estudianti Character = 100 Changed from ? to 0.

Prozostrodon brasiliensis Character = 100 Changed from ? to 0.

Alemoatherium huebneri Character = 100 Changed from ? to 0.

Botucaraitherium belarminoi Character = 100 Changed from ? to 0.

Santacruzgnathus abdalai Character = 100 Changed from ? to 0.

Charruodon Character = 100 Changed from ? to 0.

Microconodon tenuirostris Character = 100 Changed from ? to 0.

Pseudotherium argentinus Character = 100 Changed from ? to 0. .

101. Postcanine morphology (lowers and uppers)

0 conical or simple

1 sectorial without or with incipient cingulum broadening the crown

2 sectorial with a well developed lingual cingulum

3 bucco lingually expanded including multicuspidate with their cusps aligned in series

Procynosuchus delaharpeae Character = 101 NHMUK PV37054.

Galesaurus planiceps Character = 101 SAM-PK-K1119.

Thrinaxodon liorhinus Character = 101 Abdala et al (2013).

Platycraniellus elegans Character = 101 TM 25, NMQR 1633.

Cynognathus crateronotus Character = 101 BSP 1934 VIII1, BSP 1934 VIII2.

Diademodon tetragonus Character = 101 The postcanine series in *Diademodon tetragonus* is complex with different morphologies ranging form conical to sectorial and to gomphodont. The latter are predominant in the dental series and we score this morphology as the typical of the taxon. MB R1004.

Trirachodon spp. Character = 101 Sometimes the dental series is composed by sectorial and gomphodonts and these are the predominant element in the series. BP/1/4658.

Langbergia modisei Character = 101 The dental series is composed by sectorial and gomphodonts and these are the predominant element in the series. NMQR 3251, NMQR 3255.

Sinognathus gracilis Character = 101 IVPP V2339.

Beishanodon youngi Character = 101 This is an expanded postcanine. There are three cusps forming a transverse crest. They are quite different to those of any other gomphodonts but we think that retains some features of this postcanines.

Scalenodon spp. Character = 101 UMZC T907.

Luangwa spp. Character = 101 BP/1/3731.

Nanogomphodon wildi Character = 101 A lower gomphodont tooth is the only element representing this taxon.

Mandagomphodon hirschsoni Character = 101 Gomphodont postcanines.

Mandagomphodon attridgei Character = 101 Gomphodont postcanines.

Gomphodontosuchus brasiliensis Character = 101 GPIT/RE/09397.

Scalenodontoides macrodontes Character = 101 BP/1/5395.

Aleodon brachyramphus Character = 101 Changed from 3 to 2.

Aleodon cromptoni Character = 101 Changed from 3 to 2.

Probainognathus jenseni Character = 101 Changed from 2 to 1.

Boreogomphodon jeffersoni Character = 101 Changed from 2 to 3.

Arctotraversodon plemmyridon Character = 101 Changed from 1 to 3.

Brasilodon tetragonus Character = 101 Changed from 2 to 1.

Morganucodon spp. Character = 101 Changed from 2 to 1.

Bonacynodon schultzi Character = 101 Changed from 2 to 1 but teeth are not observable in lingual view.

Protheriodon estudianti Character = 101 Changed from 2 to 1.

Prozostrodon brasiliensis Character = 101 Changed from 3 to 1.

Alemoatherium huebneri Character = 101 Changed from ? to 1 (only lower postcanines visible).

Botucaraitherium belarminoi Character = 101 Changed from 3 to 1.

Candelariodon Character = 101 I do not know if the other teeth were expanded as in Aleodon.

Pseudotherium argentinus Character = 101 Changed from 2 to 1.

102. Posterior postcanines with strongly curved main cusp

0 absent

1 present

Procynosuchus delaharpeae Character = 102 RC 92.

Mandagomphodon hirschsoni Character = 102 All postcanines are gomphodont.

Mandagomphodon attridgei Character = 102 All postcanines are gomphodont.

Andescynodon mendozensis Character = 102 Changed from ? to 0.

Santacruzodon hopsoni Character = 102 Changed from ? to 0.

Protuberum cabralense Character = 102 Changed from ? to 0.

Therioherpeton cargnini Character = 102 Changed from ? to 0.

Arctotraversodon plemmyridon Character = 102 Changed from ? to 0.

Dadadon isaloi Character = 102 Changed from ? to 0.

Menadon besairiei Character = 102 Changed from ? to 0.

Elliotherium kersteni Character = 102 Changed from ? to 0.

Cricodon metabolus Character = 102 Changed from 1 to 0.

103. Transverse crest in upper postcanines (This is discriminating patterns of gomphodont postcanines.)

0 absent

1 present with two cusps

2 present with three or more cusps

Charassognathus gracilis Character = 103 Changed from ? to 0.

Procynosuchus delaharpeae Character = 103 Not gomphodont.

Galesaurus planiceps Character = 103 Not gomphodont.

Thrinaxodon liorhinus Character = 103 Not gomphodont.

Platycraniellus elegans Character = 103 Not gomphodont.

Cynognathus crateronotus Character = 103 Not gomphodont.

Diademodon tetragonus Character = 103 AM 3753, MB R1004.

Trirachodon spp. Character = 103 BP/1/4658.

Langbergia modisei Character = 103 NMQR 3255.

Sinognathus gracilis Character = 103 IVPP V2339 .

Beishanodon youngi Character = 103 Gao et al. (2010: 14): ..a well-defined transverse crest located posteriorly on the crown. Three cusps are developed along the transverse crest.

Scalenodon spp. Character = 103 NHM PV R36801, UMZC T910.

Luangwa spp. Character = 103 BP/1/3731, NHMUK PV R 36995.

Mandagomphodon hirschsoni Character = 103 Hopson (2014: fig. 14.6b).

Mandagomphodon attridgei Character = 103 This is clear in erupting posterior postcanines.

Gomphodontosuchus brasiliensis Character = 103 GPIT/RE/09397.

Scalenodontoides macrodontes Character = 103 BP/1/5395.

Traversodon stahleckeri Character = 103 Changed from 2 to 1 after Barberena 1981.

Trucidocynodon riograndensis Character = 103 Changed from 0 to -.

Oligokyphus spp. Character = 103 Changed from 2 to 0.

Kayentatherium wellesi Character = 103 Changed from 2 to 0.

Tritylodon longaevus Character = 103 Changed from 2 to 0.

Bienotherium spp. Character = 103 Changed from 2 to 0.

Diarthrognathus broomi Character = 103 This is inferred from the lowers.

Nanictosaurus kitchingi Character = 103 Changed from ? to 0.

Tritheledon riconoi Character = 103 Martinelli and Rougier 2007 said it had one cusp.

104. Upper postcanine roots

0 single

1 constricted root with incipient longitudinal groove

2 divided into two longitudinal aligned roots

3 multiple roots more than two

Charassognathus gracilis Character = 104 From paper Botha et al 2007.

Procynosuchus delaharpeae Character = 104 RC 133.

Galesaurus planiceps Character = 104 Pusch et al (2019), Norton et al. (2020).

Thrinaxodon liorhinus Character = 104 Abdala et al. (2013).

Platycraniellus elegans Character = 104 TM 25.

Cynognathus crateronotus Character = 104 BSP 1934 VIII1, BSP 1934 VIII2. Inferred for the undivided alveoli but not observed.

Diademodon tetragonus Character = 104 NHMUK R3723.

Trirachodon spp. Character = 104 SAM-PK-K171.

Langbergia modisei Character = 104 NMQR 3251.

Beishanodon youngi Character = 104 Gao et al. (2010: fig. 5).

Scalenodon spp. Character = 104 UMZC T911.

Luangwa spp. Character = 104 MCP 3167PV .

Mandagomphodon hirschsoni Character = 104 This is inferred because roots in all known traversodontid tooth are single. But it was not observed.

Mandagomphodon attridgei Character = 104 This is inferred because roots in all known traversodontid tooth are single. But it was not observed.

Gomphodontosuchus brasiliensis Character = 104 This is inferred because roots in all known traversodontid tooth are single. But it was not observed.

Scalenodontoides macrodontes Character = 104 Observed in isolated teeth in the BP/1/5395. Gow and Hancox (1993, fig. 6). .

Elliotherium kersteni Character = 104 Changed from ? to 0.

Diarthrognathus broomi Character = 104 Changed from 0 to ?.

Progalesaurus lootbergensis Character = 104 Should be 0 single.

105. Lower postcanine roots

0 single

1 incipiently bifurcated

2 divided

3 divided with multiple cusps

Charassognathus gracilis Character = 105 Changed from ? to 0.

Procynosuchus delaharpeae Character = 105 RC 133.

Galesaurus planiceps Character = 105 Pusch et al. (2019), Norton et al (2020).

Thrinaxodon liorhinus Character = 105 Abdala et al (2013).

Platycraniellus elegans Character = 105 TM 25.

Cynognathus crateronotus Character = 105 BSP 1934 VIII1, BSP 1934 VIII2. Inferred for the undivided alveoli but not observed.

Diademodon tetragonus Character = 105 SAM-PK-571b, SAM-PK-K177.

Trirachodon spp. Character = 105 SAM-PK-K171.

Langbergia modisei Character = 105 NMQR 3251.

Scalenodon spp. Character = 105 UMZC T910.

Luangwa spp. Character = 105 GSN RK-4a.

Mandagomphodon hirschsoni Character = 105 This is inferred because roots in all known traversodontid tooth are single. But it was not observed.

Santacruzodon hopsoni Character = 105 Changed from ? to 0.

Therioherpeton cargnini Character = 105 Changed from 1 to ?.

Boreogomphodon jeffersoni Character = 105 Changed from ? to 0.

Arctotraversodon plemmyridon Character = 105 Changed from ? to 0.

Traversodon stahleckeri Character = 105 Changed from ? to 0 after von Huene 1936.

Chaliminia musteloides Character = 105 Changed from 1 to 0.

Dadadon isaloi Character = 105 Changed from ? to 0.

Menadon besairiei Character = 105 Changed from ? to 0.

Diarthrognathus broomi Character = 105 Changed from 1 to 0.

Sinoconodon rigneyi Character = 105 Changed from 1/2 to 2.

Morganucodon spp. Character = 105 Changed from 1 to 2.

Dvinia prima Character = 105 Changed from ? to 0. FA observation in June 2009. Part of the roots of the pc are observed on the right series and it is one root.

Progalesaurus lootbergensis Character = 105 Should be 0 single.

Nanictosaurus kitchingi Character = 105 Changed from ? to 0. It seems to be a furrow in pc2 in RC47 but we do not consider it a double rooted tooth. .

106. Position of upper transverse cusp row on crown (State 3, absent, is added for gomphodont teeth lacking transverse cusp row which is the case of *Gomphodontosuchus*, *Scalenodontoides macrodontes*, *Exaeretodon* spp. and *Siriusgnathus*.)

0 on anterior half of crown

1 midcrown almost to posterior margin

2 at posterior margin with no posterior cingulum

3 absent

Charassognathus gracilis Character = 106 Changed from ? to -.

Diademodon tetragonus Character = 106 SAM-PK-571a.

Trirachodon spp. Character = 106 NHM PV R3307, BSP 1934 VIII21. Abdala et al. (2006, 408): The location of the crest in the first taxon (*Trirachodon* spp.) is clearly central in many specimens (e.g. BP/1/4661, SAM-PK-K-171), but is quite close to the posterior margin in some postcanines of BSP 1934 VIII 21 (see Broili & Schröder, 1935a: fig. 3). .

Langbergia modisei Character = 106 NMQR 3251, NMQR 3255.

Sinognathus gracilis Character = 106 IVPP V2339.

Scalenodon spp. Character = 106 Abdala et al. (2006, 408-409): In UMCZ T910 (the best-preserved series of postcanines in *Scalenodon angustifrons*), the upper postcanines of the middle of the series show the crest close to the middle of the^n^ncrown, whereas in the posterior teeth the crest is closer to the posterior margin.

Luangwa spp. Character = 106 BP/1/3971.

Mandagomphodon hirschsoni Character = 106 Hopson (2014: fig. 14.6b).

Mandagomphodon attridgei Character = 106 This is clear in erupting posterior postcanines.

Arctotraversodon plemmyridon Character = 106 Changed from ? to 2.

Dadadon isaloi Character = 106 Changed from ? to 2.

Dvinia prima Character = 106 Changed from 1 to - as there is no transverse crest.

Nanictosaurus kitchingi Character = 106 Changed from ? to -.

107. Central cusp of upper transverse row

0 absent

1 midway between buccal and lingual cusps

2 closer to lingual cusp

3 close to labial cusp

Charassognathus gracilis Character = 107 Changed from ? to -.

Diademodon tetragonus Character = 107 SAM-PK-571a.

Trirachodon spp. Character = 107 NHM PV R3307, BSP 1934 VIII21.

Langbergia modisei Character = 107 NMQR 3251, NMQR 3255.

Beishanodon youngi Character = 107 Gao et al (2010: fig. 6A). This is contra Gao et al (2010:14): Three cusps are developed along the transverse crest, with the central cusp close to the middle. In the figure the central cusp lokks closer to the labial one (see also Hendrick et al., 2019: 56).

Scalenodon spp. Character = 107 UMCZ T910.

Luangwa spp. Character = 107 BP/1/3731.

Mandagomphodon hirschsoni Character = 107 Hopson (2014: fig. 14.6b).

Mandagomphodon attridgei Character = 107 This is clear in erupting posterior postcanines.

Gomphodontosuchus brasiliensis Character = 107 GPIT/RE/09397.

Scalenodontoides macrodontes Character = 107 BP/1/5395.

Andescynodon mendozensis Character = 107 Changed from ? to 0.

Protuberum cabralense Character = 107 Changed from ? to 0. .

Traversodon stahleckeri Character = 107 Changed from 2 to 0 after Barberena 1981.

Chaliminia musteloides Character = 107 Changed from 0 to -. .

Menadon besairiei Character = 107 Changed from ? to 0.

Kayentatherium wellesi Character = 107 I would score this as - because the transverse crest is that of traversodontids. If not, we should also score character 113.

Bienotherium spp. Character = 107 I would score this as - because the transverse crest is that of traversodontids. If not, we should also score character 113.

Dvinia prima Character = 107 Changed from 1 to - as there is no transverse crest.

Nanictosaurus kitchingi Character = 107 Changed from ? to -.

108. Upper postcanine buccal cingulum

0 absent

1 present

Charassognathus gracilis Character = 108 Changed from ? to 0.

Procynosuchus delaharpeae Character = 108 NHMUK PV37054.

Galesaurus planiceps Character = 108 Pusch et al. (2019). Norton et al (2020).

Thrinaxodon liorhinus Character = 108 Abdala et al. (2013).

Platycraniellus elegans Character = 108 TM 25, NMQR 1633.

Cynognathus crateronotus Character = 108 BSP 1934 VIII1.

Diademodon tetragonus Character = 108 SAM-PK-571a.

Trirachodon spp. Character = 108 NHM PV R3307, BSP 1934 VIII21.

Langbergia modisei Character = 108 NMQR 3251, NMQR 3255.

Sinognathus gracilis Character = 108 IVPP V2339.

Luangwa spp. Character = 108 BP/1/3731. NHMUK PV R 36995.

Mandagomphodon hirschsoni Character = 108 Hopson (2014: fig. 14.6b).

Mandagomphodon attridgei Character = 108 NHMUK R8578.

Gomphodontosuchus brasiliensis Character = 108 GPIT/RE/09397.

Scalenodontoides macrodontes Character = 108 BP/1/5395.

Pascualgnathus polanski Character = 108 Changed from ? to 0.

Therioherpeton cargnini Character = 108 Changed from 0 to -.

Traversodon stahleckeri Character = 108 Changed from 1 to 0 after Barberena 1981.

Diarthrognathus broomi Character = 108 Changed from 1 to ?.

Dvinia prima Character = 108 Changed from - to 0.

Nanictosaurus kitchingi Character = 108 Changed from ? to 0.

Bonacynodon schultzi Character = 108 Changed from ? to 0.

Protheriodon estudianti Character = 108 Changed from ? to 0.

Prozostrodon brasiliensis Character = 108 Changed from ? to 0.

Botucaraitherium belarminoi Character = 108 Changed from ? to 0.

Pseudotherium argentinus Character = 108 Changed from ? to 0. .

Tritheledon riconoi Character = 108 Different from Martinelli and Rougier 2007 scoring.

109. Upper anterobuccal accessory cusp in labiolingually expanded teeth (Character introduced by Hopson and Kitching (2001, character 67). The scoring is restricted to bucco-lingually expanded postcanines.)

0 present

1 absent

Charassognathus gracilis Character = 109 Changed from 0 to -.

Diademodon tetragonus Character = 109 Changed from 0 to 1 after SAM-PK-571a (see Hendrickx et al., 2019, fig. 3).

Trirachodon spp. Character = 109 Changed from 0 to 1. after Hendrickx et al. (2019, fig. 3I and 3J).

Langbergia modisei Character = 109 Hendrickx et al (2019, fig. 3E).

Sinognathus gracilis Character = 109 Changed from 0 to 1. I cannot observe evidence of accesory cusps in the labial margin of the postecanines (see also Sun, 1988, fig. 4).

Beishanodon youngi Character = 109 Hendrickx et al. (2019, fig. 3L).

Scalenodon spp. Character = 109 Hendrickx et al. (2019, fig. 2C).

Luangwa spp. Character = 109 Cusp absent in BP/1/3731 but present in NHMUK PV R 36995 (see Abdala and Teixeira, 2004, fig. 9a and 9b).

Mandagomphodon hirschsoni Character = 109 Hopson (2014: fig. 14.6a,b).

Mandagomphodon attridgei Character = 109 Only two cusps in the buccal margin, the main posterior one and an anterobuccal accesory.

Gomphodontosuchus brasiliensis Character = 109 Hopson (1985, fig. 4A).

Scalenodontoides macrodontes Character = 109 Isolated tooth of BP/1/5395. Tentative score.

Pascualgnathus polanski Character = 109 Changed from 0 to 1.

Andescynodon mendozensis Character = 109 Changed from ? to 1.

Probainognathus jenseni Character = 109 Changed from 0 to -.

Therioherpeton cargnini Character = 109 Changed from 0 to -.

Boreogomphodon jeffersoni Character = 109 Changed from ? to 0.

Arctotraversodon plemmyridon Character = 109 Changed from ? to 1.

Lumkuia fuzzi Character = 109 Changed from 0 to -.

Dadadon isaloi Character = 109 Changed from ? to 0.

Menadon besairiei Character = 109 Changed from ? to 0.

Ecteninion lunensis Character = 109 Changed from 0 to -.

Trucidocynodon riograndensis Character = 109 Changed from 0 to -.

Chiniquodon spp. Character = 109 Changed from 0 to -.

Oligokyphus spp. Character = 109 Changed from 0 to -.

Tritylodon longaevus Character = 109 Changed from 0 to -.

Elliotherium kersteni Character = 109 Changed from 1 to -.

Pachygenelus monus Character = 109 Changed from 1 to -.

Diarthrognathus broomi Character = 109 Changed from 1 to -.

Sinoconodon rigneyi Character = 109 Changed from 0 to -.

Morganucodon spp. Character = 109 Changed from 1 to -.

Dvinia prima Character = 109 Changed from ? to -.

Cynosaurus suppostus Character = 109 Changed from 0 to -.

Progalesaurus lootbergensis Character = 109 Changed from 0 to -.

Nanictosaurus kitchingi Character = 109 Changed from 0 to -.

Irajatherium hernandezi Character = 109 Changed from 1 to -.

110. Upper posterobuccal accessory cusp in labiolingually expanded teeth (Character introduced by Hopson and Kitching (2001, character 68). The scoring is restricted to bucco-lingually expanded postcanines.)

0 present

1 absent

Charassognathus gracilis Character = 110 Changed from 0 to -.

Diademodon tetragonus Character = 110 SAM-PK-571a (Hendrickx et al., 2019, fig. 3).

Trirachodon spp. Character = 110 A posterobuccal accesory cusp is present in Hendricks et al. (2019, fig. 3J) but absent in Hendrick et al. (2019, fig. 3I) because the main labial cusp is located in the distal margin of the tooth.

Langbergia modisei Character = 110 Hendrickx et al (2019, fig. 3E).

Sinognathus gracilis Character = 110 Changed from 0 to 1. I cannot observe evidence of accesory cusps in the labial margin of the postecanines (see also Sun, 1988, fig. 4).

Beishanodon youngi Character = 110 Hendrickx et al. (2019, fig. 3L). .

Scalenodon spp. Character = 110 Hendrickx et al. (2019, fig. 2C).

Luangwa spp. Character = 110 Abdala and Teixeira (2004, fig. 9a and 9b).

Mandagomphodon hirschsoni Character = 110 Hopson (2014: fig. 14.6a,b).

Mandagomphodon attridgei Character = 110 Only two cusps in the buccal margin, the main posterior one and an anterobuccal accesory.

Gomphodontosuchus brasiliensis Character = 110 Changed form 0 to 1 (Hopson, 1985, fig. 4A).

Scalenodontoides macrodontes Character = 110 Isolated tooth of BP/1/5395. Tentative score. Gow and Hancox, 1993, 165): Worn in upper postcanines have the three in-line labial cusps flanking a near vertical shearing plane, the most distal of these cusps being separated from the major (central) cusp by a vertical depression, which Chattetjee has aptly described as fluting. Tentative score.

Pascualgnathus polanski Character = 110 Changed from 1 to 0.

Andescynodon mendozensis Character = 110 Changed from ? to 0.

Santacruzodon hopsoni Character = 110 Changed from 0 to 1.

Probainognathus jenseni Character = 110 Changed from 0 to -.

Therioherpeton cargnini Character = 110 Changed from 0 to -.

Boreogomphodon jeffersoni Character = 110 Changed from ? to 0.

Arctotraversodon plemmyridon Character = 110 Changed from ? to 1.

Lumkuia fuzzi Character = 110 Changed from 0 to -.

Ecteninion lunensis Character = 110 Changed from 0 to -.

Trucidocynodon riograndensis Character = 110 Changed from 0 to -.

Chiniquodon spp. Character = 110 Changed from 0 to -.

Oligokyphus spp. Character = 110 Changed from 0 to -.

Tritylodon longaevus Character = 110 Changed from 1 to -.

Elliotherium kersteni Character = 110 Changed from 1 to -.

Pachygenelus monus Character = 110 Changed from 0 to -.

Diarthrognathus broomi Character = 110 Changed from 1 to -.

Sinoconodon rigneyi Character = 110 Changed from 0 to -.

Morganucodon spp. Character = 110 Changed from 0 to -.

Dvinia prima Character = 110 Changed from 1 to -.

Cynosaurus suppostus Character = 110 Changed from 0 to -.

Progalesaurus lootbergensis Character = 110 Changed from 0 to -.

Nanictosaurus kitchingi Character = 110 Changed from 0 to -.

Cricodon metabolus Character = 110 CHECK.

Irajatherium hernandezi Character = 110 Changed from 0 to -.

111. Upper anterolingual accessory cusp in labiolingually expanded teeth (Character introduced by Hopson and Kitching (2001, character 69). The scoring is restricted to bucco-lingually expanded postcanines.)

0 absent

1 present

Charassognathus gracilis Character = 111 Changed from 0 to -.

Diademodon tetragonus Character = 111 SAM-PK-571a (Hendrickx et al., 2019, fig. 3).

Trirachodon spp. Character = 111 An anterolingual accesory cusp is observed in Hendrickx et al. (2019) fig 3I, but it is absent in fig. 3J.

Langbergia modisei Character = 111 Changed from 0 to 1. Hendrickx et al (2019, fig. 3E).

Sinognathus gracilis Character = 111 Hendrickx et al (2019, fig.11J).

Beishanodon youngi Character = 111 Hendrickx et al. (2019, fig. 3L). .

Scalenodon spp. Character = 111 Changed from 0 to 1. Hendrickx et al. (2019, fig. 2C).

Luangwa spp. Character = 111 Abdala and Teixeira (2004, fig. 9a and 9b).

Mandagomphodon hirschsoni Character = 111 Hopson (2014: fig. 14.6 b,c).

Mandagomphodon attridgei Character = 111 There are three cusps in the lingual margin of the postcanines. The largest posterior one and one central and other anterior of approximately similar size.

Gomphodontosuchus brasiliensis Character = 111 Hopson (1985, fig. 4A.

Andescynodon mendozensis Character = 111 Changed from ? to 0.

Santacruzodon hopsoni Character = 111 Changed from 1 to 0.

Protuberum cabralense Character = 111 Changed from 1 to 0. .

Therioherpeton cargnini Character = 111 Changed from 0 to -.

Traversodon stahleckeri Character = 111 Changed from 1 to 0 after Barberena 1981.

Brasilodon tetragonus Character = 111 Chnaged form 1 to -.

Chiniquodon spp. Character = 111 The cu.

Oligokyphus spp. Character = 111 Changed from 1 to -.

Kayentatherium wellesi Character = 111 Changed from 0 to -.

Tritylodon longaevus Character = 111 Changed from 1 to -.

Elliotherium kersteni Character = 111 Changed from 0 to -.

Diarthrognathus broomi Character = 111 Changed from 0 to ?.

Sinoconodon rigneyi Character = 111 Changed from 1 to -.

Morganucodon spp. Character = 111 Changed from 0 to -.

Dvinia prima Character = 111 Changed from 1 to -.

Cynosaurus suppostus Character = 111 Changed from 0 to -.

Progalesaurus lootbergensis Character = 111 Changed from 0 to -.

Nanictosaurus kitchingi Character = 111 Changed from 0 to -.

Cricodon metabolus Character = 111 CHECK.

Diegocanis elegans Character = 111 Changed from 0 to -.

Irajatherium hernandezi Character = 111 Changed from 0 to -.

112. Upper anterior transverse cingulum or ridge

0 low

1 high

Charassognathus gracilis Character = 112 Changed from ? to -.

Procynosuchus delaharpeae Character = 112 No anterior transverse crest or ridge.

Galesaurus planiceps Character = 112 No anterior transverse crest or ridge.

Thrinaxodon liorhinus Character = 112 No anterior transverse crest or ridge.

Platycraniellus elegans Character = 112 No anterior transverse crest or ridge.

Cynognathus crateronotus Character = 112 No anterior transverse crest or ridge.

Diademodon tetragonus Character = 112 SAM-PK-571a (Hendrickx et al., 2019, fig. 3).

Trirachodon spp. Character = 112 Hendrickx et al (2019, fig. 3I, 3J).

Langbergia modisei Character = 112 Hendrickx et al (2019, fig. 3E).

Sinognathus gracilis Character = 112 Following Sun (1988, fig. 4).

Beishanodon youngi Character = 112 Gao et al (2010: 14) describe the first postcanine in this way: "The crown displays weakly developed anterior and posterior crests or cingula, each bearing two to three small cuspules", In the other postcanines they describe only aposterior crest or cingulum. Considering the condition in the majority of the postcanine known we score - for this character.

Scalenodon spp. Character = 112 Hendrickx et al. (2019, fig. 2C).

Luangwa spp. Character = 112 Abdala and Teixeira (2004, fig. 9a and 9b).

Mandagomphodon hirschsoni Character = 112 Hopson (2014: fig. 14.6b,c).

Mandagomphodon attridgei Character = 112 Observed in posterior postcanine in eruption.

Gomphodontosuchus brasiliensis Character = 112 Scoring of high is for teeth in which is identified the anterior wall (see Hopson, 1985, fig. 4a and 5).

Scalenodontoides macrodontes Character = 112 BP/1/5395. The anterior wall is also present as in *Exaeretodon* spp..

Andescynodon mendozensis Character = 112 Changed from ? to 0.

Protuberum cabralense Character = 112 absent.

Ecteninion lunensis Character = 112 Changed from ? to -.

Trucidocynodon riograndensis Character = 112 Changed form ? to -.

Dvinia prima Character = 112 Changed from 1 to -. CHECK.

Cynosaurus suppostus Character = 112 Changed from 0 to ?.

Nanictosaurus kitchingi Character = 112 Changed from 1 to -.

Tessellatia bonapartei Character = 112 This is absent.

Bonacynodon schultzi Character = 112 Changed from ? to -. Absent.

Prozostrodon brasiliensis Character = 112 absent.

Botucaraitherium belarminoi Character = 112 Absent. Changed from ? to -.

Pseudotherium argentinus Character = 112 absent.

Diegocanis elegans Character = 112 absent.

Irajatherium hernandezi Character = 112 absent.

113. Upper lingual ridge (Character originally proposed by Hopson and Kitching (2001, character 71). The basic distinction is if the lingual margin of the gomphodont postcanine is rounded (absent) or straight (present). Score 0 was in Diademodon, *Trirachodon* spp., *Pascualgnathus polanski*, and *Scalenodon* spp.. Remaining traversodontids and tritylodontids scored 1.)

0 absent

1 present

Procynosuchus delaharpeae Character = 113 States to score in gomphodont pc.

Galesaurus planiceps Character = 113 States to score in gomphodont pc.

Thrinaxodon liorhinus Character = 113 States to score in gomphodont pc.

Platycraniellus elegans Character = 113 States to score in gomphodont pc.

Cynognathus crateronotus Character = 113 States to score in gomphodont pc.

Diademodon tetragonus Character = 113 SAM-PK-571a (Hendrickx et al., 2019, fig. 3).

Trirachodon spp. Character = 113 Hendrickx et al (2019, fig. 3I, 3J).

Langbergia modisei Character = 113 Hendrickx et al (2019, fig. 3E).

Sinognathus gracilis Character = 113 Hendrickx et al (2019, fig.11J).

Beishanodon youngi Character = 113 Hendrickx et al. (2019, fig. 3L). .

Scalenodon spp. Character = 113 Hendrickx et al. (2019, fig. 2C).

Luangwa spp. Character = 113 NHMUK PV R 36995.

Mandagomphodon hirschsoni Character = 113 Hopson (2014, fig. 14.5a).

Mandagomphodon attridgei Character = 113 The lingual margin is straight in the posterior unworn postcanines.

Gomphodontosuchus brasiliensis Character = 113 Hopson (1985, fig. 4A).

Scalenodontoides macrodontes Character = 113 Isolated tooth of BP/1/5395.

Andescynodon mendozensis Character = 113 Changed from ? to 0.

Santacruzodon hopsoni Character = 113 Changed from ? to 0.

Protuberum cabralense Character = 113 Changed from ? to 0. .

Therioherpeton cargnini Character = 113 Changed from 0 to -.

Boreogomphodon jeffersoni Character = 113 Changed from ? to 0.

Arctotraversodon plemmyridon Character = 113 Changed from ? to 0.

Dadadon isaloi Character = 113 Changed from ? to 0.

Menadon besairiei Character = 113 Changed from ? to 0.

Oligokyphus spp. Character = 113 Changed from 0 to -.

Kayentatherium wellesi Character = 113 Changed from 0 to -.

Tritylodon longaevus Character = 113 Changed from 1 to -.

Bienotherium spp. Character = 113 Changed from 1 to -.

Pachygenelus monus Character = 113 Changed from 1 to -.

Diarthrognathus broomi Character = 113 Changed from 0 to ?.

Sinoconodon rigneyi Character = 113 Changed from 1 to -.

Morganucodon spp. Character = 113 Changed from 1 to -.

Dvinia prima Character = 113 Changed from 0 to -.

Cynosaurus suppostus Character = 113 Changed from 0 to -.

Nanictosaurus kitchingi Character = 113 Changed from 1 to -.

Diegocanis elegans Character = 113 Changed from 0 to -.

Irajatherium hernandezi Character = 113 Changed from 0 to -.

114. Transverse axis of crown strongly oblique to midline axis (Character introduced by Hopson and Kitching (2001, character 72) and scored only in gomphodont cynodonts and tritylodontids. State 1 was scored in *Exaeretodon* spp. and Gomphodontosuchus. Scoring is not possible in sectorial toothed cynodonts because postcanines are following the snout margin and the posterior postcanines tend to be indefectibly oblique to the midline axis of the skull.)

0 absent

1 present

Charassognathus gracilis Character = 114 Changed from ? to 0.

Procynosuchus delaharpeae Character = 114 To score only in bucco-lingually expanded crowns.

Galesaurus planiceps Character = 114 To score only in bucco-lingually expanded crowns.

Thrinaxodon liorhinus Character = 114 To score only in bucco-lingually expanded crowns.

Platycraniellus elegans Character = 114 To score only in bucco-lingually expanded crowns.

Cynognathus crateronotus Character = 114 To score only in bucco-lingually expanded crowns.

Diademodon tetragonus Character = 114 Hendrickx et al. (2019, fig. 3B).

Trirachodon spp. Character = 114 Hendrickx et al (2019, fig. 3K).

Langbergia modisei Character = 114 Changed from 1 to 0. Hendrickx et al (2019, fig. 3F). For this were considered the axis of gomphodont postcanines only.

Sinognathus gracilis Character = 114 IVPP V2339.

Beishanodon youngi Character = 114 Gao et al. (2010, p. 14): The last upper postcanine is not preserved on either side of PKUP V3007, but the alveolus at this tooth position is set at an oblique angle (roughly 45 degrees) in relation to the axis of the tooth row (fig. 5).

Scalenodon spp. Character = 114 UMZC T907.

Luangwa spp. Character = 114 NHMUK PV R 36995.

Mandagomphodon hirschsoni Character = 114 Hopson (2014: fig. 14.1b).

Mandagomphodon attridgei Character = 114 The transverse axis of crown strongly oblique to midline axis at least in the posterior postcanines.

Gomphodontosuchus brasiliensis Character = 114 GPIT/RE/09397. Hopson (1985, fig. 3).

Scalenodontoides macrodontes Character = 114 BP/1/5395.

Andescynodon mendozensis Character = 114 Changed from ? to 0.

Santacruzodon hopsoni Character = 114 Changed from ? to 0.

Protuberum cabralense Character = 114 Changed from ? to 0. .

Probainognathus jenseni Character = 114 Changed from ? to 0.

Boreogomphodon jeffersoni Character = 114 Changed from ? to 0.

Lumkuia fuzzi Character = 114 Changed from ? to 0.

Traversodon stahleckeri Character = 114 Changed from 0 to 1 after Barberena 1981.

Brasilodon tetragonus Character = 114 Changed from ? to 0.

Chaliminia musteloides Character = 114 Changed from ? to 0.

Dadadon isaloi Character = 114 Changed from ? to 0.

Menadon besairiei Character = 114 Changed from 1 to 0.

Ecteninion lunensis Character = 114 Changed from ? to -.

Trucidocynodon riograndensis Character = 114 Changed form ? to 0.

Chiniquodon spp. Character = 114 Changed from ? to 0.

Bienotherium spp. Character = 114 Changed from ? to 0.

Elliotherium kersteni Character = 114 Changed from ? to 0.

Pachygenelus monus Character = 114 Changed from ? to 0.

Morganucodon spp. Character = 114 Changed from ? to 0.

Progalesaurus lootbergensis Character = 114 Changed from ? to 0.

Nanictosaurus kitchingi Character = 114 Changed from ? to 0.

Tessellatia bonapartei Character = 114 This character is poorly defined if it refers to the teeth being obliquely placed regarding the long axis of the tooth row. Check this scoring.

Bonacynodon schultzi Character = 114 Changed from ? to 0.

Protheriodon estudianti Character = 114 Changed from ? to 0.

Prozostrodon brasiliensis Character = 114 Changed from ? to 0.

Botucaraitherium belarminoi Character = 114 Changed from ? to 0.

Santacruzgnathus abdalai Character = 114 Changed from ? to 0 (on the basis of the last lower postcanine).

Pseudotherium argentinus Character = 114 Changed from ? to 0. .

115. Number of lower cusps in transverse row (This character was introduced by Hopson and Kitching (2001, character 73). They score 0 (one cusp) in sectorial toothed cynodonts but they do not have a transverse row and they should be scored as "-" instead.)

0 1

1 2

2 3 or more

Charassognathus gracilis Character = 115 Changed from ? to -.

Procynosuchus delaharpeae Character = 115 This is to score in gomphodont pc.

Galesaurus planiceps Character = 115 This is to score in gomphodont pc.

Thrinaxodon liorhinus Character = 115 This is to score in gomphodont pc.

Platycraniellus elegans Character = 115 This is to score in gomphodont pc.

Cynognathus crateronotus Character = 115 This is to score in gomphodont pc.

Diademodon tetragonus Character = 115 Score changed from 1 to 2 after Hendrickx et al (2019, figure 3N).

Trirachodon spp. Character = 115 Hendrickx et al (2019, fig. 10A).

Langbergia modisei Character = 115 Hendrickx et al (2019, fig. 7M).

Sinognathus gracilis Character = 115 Hendickx et al (2019, 55):The transverse crest is slightly mesially deflected and includes labial, central, and lingual cusps roughly sharing the same height. But Sun (1988): they are narrower than those uppers, and each has two cusps.

Beishanodon youngi Character = 115 No mandible.

Scalenodon spp. Character = 115 Hendrickx et al. (2019, fig. 2D).

Luangwa spp. Character = 115 Hendrickx et al. (2020, fig. 13D).

Nanogomphodon wildi Character = 115 Hopson and Sues (2006, fig 1).

Mandagomphodon hirschsoni Character = 115 Hopson (2014: fig. 14.7b,d).

Gomphodontosuchus brasiliensis Character = 115 Hopson (1985, fig. 4B).

Scalenodontoides macrodontes Character = 115 MNHN 1957-23.

Traversodon stahleckeri Character = 115 I was unable to check this.

Dadadon isaloi Character = 115 Changed from ? to 1.

Menadon besairiei Character = 115 Changed from 0 to ?.

Oligokyphus spp. Character = 115 Changed from 1 to -.

Kayentatherium wellesi Character = 115 Changed from 1 to -.

Tritylodon longaevus Character = 115 Changed from 1 to -.

Bienotherium spp. Character = 115 Changed from 1 to -.

Dvinia prima Character = 115 Changed from 2 to -. CHECK.

Progalesaurus lootbergensis Character = 115 Changed from ? to -.

Nanictosaurus kitchingi Character = 115 Changed from ? to -.

116. Lingual cingulum in lower postcanines (Character proposed by Abdala (2007, character 94) and scored as - for gomphodont cynodonts. Is then a character referring to the cingulum in sectorial toothed cynodonts. Perhaps could also be scored in gomphodont cynodonts with sectorial postcanines in part of their dentition.)

0 absent

1 small

2 well developed

Procynosuchus delaharpeae Character = 116 NHMUK PV37054 .

Galesaurus planiceps Character = 116 Norton et al. (2020).

Thrinaxodon liorhinus Character = 116 Abdala et al. (2013).

Platycraniellus elegans Character = 116 There is not direct evidence of this structure described in lower postcanines. Chnaged from 0 to ?.

Cynognathus crateronotus Character = 116 BSP 1934 VIII2.

Beishanodon youngi Character = 116 No mandible.

Santacruzodon hopsoni Character = 116 Changed from ? to 0.

Boreogomphodon jeffersoni Character = 116 Changed from ? to 0.

Arctotraversodon plemmyridon Character = 116 Changed from ? to 0.

Brasilodon tetragonus Character = 116 Changed from 2 to 1.

Dadadon isaloi Character = 116 Changed from 2 to -.

Ecteninion lunensis Character = 116 Changed from 2 to ?. This is not observable.

Chiniquodon spp. Character = 116 I was unable to check this scoring.

Kayentatherium wellesi Character = 116 Changed from - to 0.

Tritylodon longaevus Character = 116 Changed from - to 0.

Bienotherium spp. Character = 116 Changed from - to 0.

Pachygenelus monus Character = 116 Changed from 2 to 0.

Diarthrognathus broomi Character = 116 Changed from ? to 2.

Sinoconodon rigneyi Character = 116 There is only a a small swelling (Patterson and Olson 1961). This could be also scored as (0) absent.

Dvinia prima Character = 116 Changed from - to 0.

Progalesaurus lootbergensis Character = 116 Changed from 0 to ?.

Cricodon metabolus Character = 116 In sectorial lower postcanine.

Prozostrodon brasiliensis Character = 116 Changed from ? to 2.

Alemoatherium huebneri Character = 116 Changed from ? to 0.

Botucaraitherium belarminoi Character = 116 Changed from ? to 2.

Santacruzgnathus abdalai Character = 116 Changed from ? to 1.

Candelariodon Character = 116 Check, I am not sure.

Charruodon Character = 116 At least in the replacing tooth of the fifth alveolus.

Microconodon tenuirostris Character = 116 Changed from ? to 0.

117. Lower anterior cingulum or cusp in expanded teeth

0 absent

1 present

Procynosuchus delaharpeae Character = 117 NHMUK PV37054 .

Galesaurus planiceps Character = 117 Most of the specimens observed do not have an anterior cusp, but only one postcanine observed in three specimens (see Norton et al., 2020). Considering these thre cases as an exception we scored as absent.

Thrinaxodon liorhinus Character = 117 Abdala et al. (2013).

Cynognathus crateronotus Character = 117 BSP 1934 VIII1 (observed in posterior lower postcanines).

Diademodon tetragonus Character = 117 Hendrickx et al. (2019, fig. 3N).

Trirachodon spp. Character = 117 BP/1/4658. Hendrickx et al. (2019, fig. 3X).

Langbergia modisei Character = 117 Hendrickx et al. (2019, fig. 3T).

Beishanodon youngi Character = 117 No mandible.

Scalenodon spp. Character = 117 Hendrickx et al. (2019, fig. 2D).

Luangwa spp. Character = 117 Hendrickx et al. (2020, fig. 13D).

Nanogomphodon wildi Character = 117 Hopson and Sues (2006, fig 1).

Mandagomphodon hirschsoni Character = 117 Hopson (2014: fig. 14.7b,d).

Gomphodontosuchus brasiliensis Character = 117 Hopson (1985, fig. 4B). Tentative score for poor preservation.

Scalenodontoides macrodontes Character = 117 MNHN 1957-23.

Probainognathus jenseni Character = 117 Changed from 1 to -.

Therioherpeton cargnini Character = 117 Changed from ? to -.

Lumkuia fuzzi Character = 117 Changed from 1 to -.

Traversodon stahleckeri Character = 117 I was unable to check this.

Menadon besairiei Character = 117 Changed from 1 to ?.

Chiniquodon spp. Character = 117 Changed from 1 to -.

Oligokyphus spp. Character = 117 Changed from 0/1 to -.

Kayentatherium wellesi Character = 117 Changed from 0 to -.

Pachygenelus monus Character = 117 Changed from 1 to -.

Diarthrognathus broomi Character = 117 Changed from 0 to -.

Sinoconodon rigneyi Character = 117 Changed from 0 to -.

Morganucodon spp. Character = 117 Changed from 1 to -.

Dvinia prima Character = 117 Changed from 1 to -.

Progalesaurus lootbergensis Character = 117 Changed from 0 to -.

Nanictosaurus kitchingi Character = 117 Changed from 1 to -.

Charruodon Character = 117 Changed from 0 to -.

Irajatherium hernandezi Character = 117 Changed from 0 to -.

118. Lower posterior basin

0 absent

1 present

Charassognathus gracilis Character = 118 Changed from ? to 0.

Procynosuchus delaharpeae Character = 118 NHMUK PV37054. Kemp (1979).

Galesaurus planiceps Character = 118 Norton et al. (2020).

Thrinaxodon liorhinus Character = 118 Abdala et al. (2013).

Cynognathus crateronotus Character = 118 BSP 1934 VIII1.

Diademodon tetragonus Character = 118 Hendrickx et al. (2019, fig. 3N).

Trirachodon spp. Character = 118 BP/1/4658.

Langbergia modisei Character = 118 Hendrickx et al. (2019, fig. 3T).

Sinognathus gracilis Character = 118 Inferred for similarity with *Trirachodon* spp.tid teeth.

Beishanodon youngi Character = 118 No mandible.

Scalenodon spp. Character = 118 Hendrickx et al. (2019, fig. 2D).

Luangwa spp. Character = 118 Hendrickx et al. (2020, fig. 13D).

Nanogomphodon wildi Character = 118 Hopson and Sues (2006, fig 1).

Mandagomphodon hirschsoni Character = 118 Hopson (2014: fig. 14.7b,d).

Mandagomphodon attridgei Character = 118 Changed from 1 to ? No lower postcanines are known.

Gomphodontosuchus brasiliensis Character = 118 Hopson (1985, fig. 4B).

Scalenodontoides macrodontes Character = 118 MNHN 1957-23.

Traversodon stahleckeri Character = 118 Changred from ? to 1 after von Huene 1936.

Brasilodon tetragonus Character = 118 Chnaged from ? to 0.

Chaliminia musteloides Character = 118 Changed from ? to 0.

Trucidocynodon riograndensis Character = 118 Changed from ? to 0.

Progalesaurus lootbergensis Character = 118 Changed from ? to 0.

Cricodon metabolus Character = 118 Changed from 1 to 0.

119. Widest lower cusp in transverse row (Character proposed by Hopson and Kitching (2001, character 76), only can be scored in traversodontid cynodonts. In other cynodonts we scored ? Even basal gomphodonts are scored as ?)

0 lingual

1 buccal

Beishanodon youngi Character = 119 No mandible.

Scalenodon spp. Character = 119 Hendrickx et al. (2019, fig. 2D).

Luangwa spp. Character = 119 Hendrickx et al. (2020, fig. 13D. 14I, 14J).

Nanogomphodon wildi Character = 119 Hopson and Sues (2006, fig 1).

Mandagomphodon hirschsoni Character = 119 Hopson (2014: fig. 14.7b).

Gomphodontosuchus brasiliensis Character = 119 Hopson (1985). Tentative score.

Scalenodontoides macrodontes Character = 119 MNHN 1957-23.

Andescynodon mendozensis Character = 119 Changed from 1 to 0.

Santacruzodon hopsoni Character = 119 They are subequal.

Boreogomphodon jeffersoni Character = 119 Changed from ? to 0.

Arctotraversodon plemmyridon Character = 119 Changed from ? to 0.

Traversodon stahleckeri Character = 119 I was unable to check this.

Dadadon isaloi Character = 119 Changed from ? to 0.

Ecteninion lunensis Character = 119 Changed from ? to -.

Trucidocynodon riograndensis Character = 119 Changed from ? to -.

Dvinia prima Character = 119 Changed from ? to -.

Progalesaurus lootbergensis Character = 119 Changed from ? to -.

Nanictosaurus kitchingi Character = 119 Changed from ? to -.

Cricodon metabolus Character = 119 Central cusp is wider. Labial and lingual are subequal according to Sidor and Hopson 2018.

Bonacynodon schultzi Character = 119 Changed from ? to -.

Alemoatherium huebneri Character = 119 Changed from ? to -.

Botucaraitherium belarminoi Character = 119 Changed from ? to -.

Santacruzgnathus abdalai Character = 119 Changed from ? to -.

120. Contacts between adjacent lower postcanines

0 simple with no interlocking

1 distal cuspule of anterior molar fits into embayment between cusps of succeeding molar

Charassognathus gracilis Character = 120 Changed from ? to 0.

Procynosuchus delaharpeae Character = 120 NHMUK PV37054 .

Galesaurus planiceps Character = 120 SAM-PK-K1119.

Thrinaxodon liorhinus Character = 120 BP/1/7199. Abdala et al. (2013).

Cynognathus crateronotus Character = 120 BSP 1934 VIII1, BSP1934 VIII2.

Diademodon tetragonus Character = 120 Hendrickx et al. (2019, fig. 3B, 3O).

Trirachodon spp. Character = 120 Hendrickx et al. (2019, fig. 3K, 3Y, 3AA).

Langbergia modisei Character = 120 Hendrickx et al. (2019, fig. 3F, 3U).

Sinognathus gracilis Character = 120 Sun (1988, fig. 4).

Beishanodon youngi Character = 120 No mandible.

Scalenodon spp. Character = 120 UMZC T910.

Luangwa spp. Character = 120 NHMUK PV R 36995.

Mandagomphodon hirschsoni Character = 120 Hopson (2014: fig. 14.7b,d).

Gomphodontosuchus brasiliensis Character = 120 GPIT/RE/09397.

Scalenodontoides macrodontes Character = 120 MNHN 1957-23.

Arctotraversodon plemmyridon Character = 120 Changed from ? to 0.

Traversodon stahleckeri Character = 120 Changred from ? to 0 after von Huene 1936.

Ecteninion lunensis Character = 120 Changed from 0 to ?. This is not observable.

Alemoatherium huebneri Character = 120 There is an anterior groove but interlocking cannot be confirmed.

Botucaraitherium belarminoi Character = 120 Changed from 1 to 0. The teeth are barely in contact.

121. Posteriormost postcanines gomphodont (Character proposed by Hopson and Kitching (2001, character 80). It is difficult to understand considering the score in their data matrix. They scored ? in *Dvinia prima* and *Procynosuchus delaharpeae*, which are known by complete dentition. They scored present in some traversodontids: Megagomphodon hirschsoni, *Luangwa* spp., *Massetognathus* spp., Gomphodontosuchus, *Exaeretodon* spp. and tritylodontids. They scored absent in *Pascualgnathus polanski*, *Scalenodon* spp., *Trirachodon* spp., *Diademodon tetragonus* and all the sectorial toothed cynodonts. Several scores here are puzzling, like those of *Pascualgnathus polanski*, *Scalenodon* spp., *Dvinia prima* and *Procynosuchus delaharpeae*. The score in tritylodontid is 1 if we consider the *Tritylodon longaevus* postcanine as a gomphodont tooth.)

0 absent

1 present

Procynosuchus delaharpeae Character = 121 NHMUK PV37054 .

Diademodon tetragonus Character = 121 MB R 1004.

Trirachodon spp. Character = 121 BP/1/4658. Hendrickx et al. (2019, fig. 3K, 3Y, 3AA).

Langbergia modisei Character = 121 Hendrickx et al. (2019, fig. 3F, 3U).

Sinognathus gracilis Character = 121 IVPP V2339. Sun (1988, fig. 4). .

Scalenodon spp. Character = 121 UMZC T910.

Luangwa spp. Character = 121 Entire dentition is gomphodont.

Mandagomphodon hirschsoni Character = 121 Entire dentition is gomphodont.

Mandagomphodon attridgei Character = 121 Entire dentition is gomphodont.

Gomphodontosuchus brasiliensis Character = 121 GPIT/RE/09397.

Scalenodontoides macrodontes Character = 121 BP/1/5395.

Andescynodon mendozensis Character = 121 Changed from ? to 1. But note that in juveniles lower posterior postcanines are sectorial.

Boreogomphodon jeffersoni Character = 121 Changed from 1 to 0.

Traversodon stahleckeri Character = 121 Changred from ? to 1 after von Huene 1936.

Trucidocynodon riograndensis Character = 121 Changed from ? to 0.

Kayentatherium wellesi Character = 121 This is not correct. Posterior postcanines are expanded but not gomphodont.

Tritylodon longaevus Character = 121 This is not correct. Posterior postcanines are expanded but not gomphodont.

Bienotherium spp. Character = 121 This is not correct. Posterior postcanines are expanded but not gomphodont.

Progalesaurus lootbergensis Character = 121 Changed from ? to 0.

122. Postcanine replacement pattern

0 alternating

1 alternating delayed

2 sequential with replacement of posterior pc

3 sequential no replacement of postcanines

4 sequential replacement preserving two morphologies of postcanines in adults

5 sequential replacement preserving three morphologies of postcanines in adults

Procynosuchus delaharpeae Character = 122 Kemp (1979).

Platycraniellus elegans Character = 122 Consideirng pattern of postcanines it is scored as alternating. Tentative score.

Trirachodon spp. Character = 122 Changed from 1 to ? No study exists about tooth replacement in *Trirachodon* spp.tids.

Langbergia modisei Character = 122 Changed from 1 to ? No study exists about tooth replacement in *Trirachodon* spp.tids.

Beishanodon youngi Character = 122 Considering the kind of different pattern of the known upper postcanines we leave this score as ?.

Scalenodon spp. Character = 122 This is inferred because it is traversodontid.

Luangwa spp. Character = 122 This is inferred because it is traversodontid.

Mandagomphodon hirschsoni Character = 122 This is inferred because it is traversodontid.

Mandagomphodon attridgei Character = 122 This is inferred because is a traversodontid.

Gomphodontosuchus brasiliensis Character = 122 Tentative score. Inferred as traversodontid.

Scalenodontoides macrodontes Character = 122 Tentative score. Inferred as the same replacement is recognized in all traversodontids and there is evidence in BP/1/5395 of a last traversodontid tooth in eruption.

Boreogomphodon jeffersoni Character = 122 Changed from ? to 1 after Liu and Sues 2010.

Lumkuia fuzzi Character = 122 After scoring by Hopson and Kitching 2001.

Ecteninion lunensis Character = 122 Changed from 0 to ?. This is unknown.

Prozostrodon brasiliensis Character = 122 Possibly alternated with evidence of replacement in some elements of the series.

Botucaraitherium belarminoi Character = 122 Changed from 0 to ?. Soares et al interpret that some teeth are lost and added at the posterior end whereas others are replaced.

123. Mandibular movement during occlusion inferred from wear facets, postcanine morphology, craniomandibular articulation

0 orthal movement during power stroke

1 restricted anteroposteriorly directed power stroke

2 free anteroposteriorly directed power stroke

Charassognathus gracilis Character = 123 Scored considering the presence of relativelly simple sectorial teeth.

Procynosuchus delaharpeae Character = 123 There is no wear facets in the dentition. Orthal movement was interpreted by Crompton (1972) and Kemp (1979).

Platycraniellus elegans Character = 123 Inferred after postcanine pattern.

Beishanodon youngi Character = 123 Considering the kind of different pattern of the known upper postcanines, inference on the morphology of the lower postcanine is not certain. Because of this we prefer to score a ? here.

Scalenodon spp. Character = 123 Condition in traversodontid in which there is a small anterior movement until the contact of the transverse crest of the postcanines. By inference could be applied for any traversodontid.

Luangwa spp. Character = 123 Condition in traversodontid in which there is a small anterior movement until the contact of the transverse crest of the postcanines. By inference could be applied for any traversodontid.

Nanogomphodon wildi Character = 123 Condition in traversodontid in which there is a small anterior movement until the contact of the transverse crest of the postcanines. By inference could be applied for any traversodontid.

Mandagomphodon hirschsoni Character = 123 Condition in traversodontid in which there is a small anterior movement until the contact of the transverse crest of the postcanines. By inference could be applied for any traversodontid.

Mandagomphodon attridgei Character = 123 Condition in traversodontid in which there is a small anterior movement until the contact of the transverse crest of the postcanines. By inference could be applied for any traversodontid.

Gomphodontosuchus brasiliensis Character = 123 Condition in traversodontid in which there is a small anterior movement until the contact of the transverse crest of the postcanines. By inference could be applied for any traversodontid.

Scalenodontoides macrodontes Character = 123 Condition in traversodontid in which there is a small anterior movement until the contact of the transverse crest of the postcanines. By inference could be applied for any traversodontid.

Andescynodon mendozensis Character = 123 Changed from ? to 1.

Santacruzodon hopsoni Character = 123 Changed from ? to 1.

Protuberum cabralense Character = 123 Changed from ? to 1. .

Boreogomphodon jeffersoni Character = 123 Changed from ? to 1.

Arctotraversodon plemmyridon Character = 123 Changed from ? to 1.

Traversodon stahleckeri Character = 123 Changed from ? to 1.

Chaliminia musteloides Character = 123 Changed from ? to 0.

Dadadon isaloi Character = 123 Changed from ? to 1.

Menadon besairiei Character = 123 Changed from ? to 1.

Elliotherium kersteni Character = 123 Changed from ? to 0.

Diarthrognathus broomi Character = 123 Changed from ? to 0.

Sinoconodon rigneyi Character = 123 Changed from 2 to 0.

Morganucodon spp. Character = 123 Changed from 2 to 0.

Cynosaurus suppostus Character = 123 Changed from ? to 0.

Progalesaurus lootbergensis Character = 123 Changed from ? to 0.

Nanictosaurus kitchingi Character = 123 Changed from ? to 0.

Cricodon metabolus Character = 123 Changed from ? to 1.

Bonacynodon schultzi Character = 123 Changed form ? to 0.

Protheriodon estudianti Character = 123 Changed from ? to 0.

Alemoatherium huebneri Character = 123 Changed form ? to 0. Probably orthal movement.

Botucaraitherium belarminoi Character = 123 Changed from ? to 0.

Irajatherium hernandezi Character = 123 Dorsomedial movement.

124. Axial centrum

0 cylindrical

1 depressed

Procynosuchus delaharpeae Character = 124 RC 92, NHMUK PV37054 .

Galesaurus planiceps Character = 124 Parrington (1934, text-fig. 3). Jenkins (1971, fig. 3A, 3B).

Thrinaxodon liorhinus Character = 124 Jenkins (1971, fig. 12B, 12C).

Cynognathus crateronotus Character = 124 Seeley (1895, fig. 13, fig. 14).

Diademodon tetragonus Character = 124 AM 458, Gaetano et al. (2018, fig. 2).

Trirachodon spp. Character = 124 CGP 1/79.

Tritylodon longaevus Character = 124 Changed from ? to 1.

Cricodon metabolus Character = 124 After UMCZ T905.

125. Dens

0 absent or vestigial

1 strongly developed

Charassognathus gracilis Character = 125 From photo of the holotype.

Procynosuchus delaharpeae Character = 125 NHMUK PV37054. Kemp (1980, fig. 1) .

Galesaurus planiceps Character = 125 Butler et al. (2018, fig. 4A, 4C).

Thrinaxodon liorhinus Character = 125 Jenkins (1971, fig. 2A, 2B).

Cynognathus crateronotus Character = 125 Seeley (1895, Figs. 15, 16).

Diademodon tetragonus Character = 125 USNM V23352, Gaetano et al. (2018, fig. 2A).

Tritylodon longaevus Character = 125 Changed from ? to 1.

Cricodon metabolus Character = 125 After UMCZ T905.

126. Posterior thoracic vertebrae or mid dorsal vertebrae

0 neural spines nearly vertical or slightly inclined

1 strongly inclined

Procynosuchus delaharpeae Character = 126 NHMUK PV37054 .

Galesaurus planiceps Character = 126 Butler et al (2018, figs. 2, 3).

Thrinaxodon liorhinus Character = 126 Jenkins (1971, fig. 12C).

Cynognathus crateronotus Character = 126 NHMUK R2571.

Diademodon tetragonus Character = 126 USNM V23352.

Trirachodon spp. Character = 126 CGP 1/79.

Probainognathus jenseni Character = 126 Changed from ? to 0.

127. Anapophysis

0 absent

1 present

Procynosuchus delaharpeae Character = 127 NHMUK PV37054, RC 92 .

Galesaurus planiceps Character = 127 Butler et al (2019, figs. 2, 3).

Thrinaxodon liorhinus Character = 127 Jenkins (1971, fig. 12C).

Cynognathus crateronotus Character = 127 NHMUK R2571.

Diademodon tetragonus Character = 127 Changed from 1 to ? It was scored present as Brink (1955) by Liu and Olsen (2010). Identity of the material as *Diademodon tetragonus* was questioned by Gaetano et al. (2018).

Trirachodon spp. Character = 127 NMQR 3521.

Luangwa spp. Character = 127 Kemp (1980, fig. 16).

Andescynodon mendozensis Character = 127 Changed from ? to 1.

Brasilodon tetragonus Character = 127 Changed from ? to 0 (Agustin).

Ecteninion lunensis Character = 127 Changed from 1 to ?. No postcranium preserved.

Cricodon metabolus Character = 127 Changed from ? to 1.

128. Vertebral centra

0 amphicoelous

1 platycoelous

Procynosuchus delaharpeae Character = 128 NHMUK PV37054 .

Galesaurus planiceps Character = 128 BP/1/5064.

Thrinaxodon liorhinus Character = 128 BP/1/5905, AMM 4283.

Cynognathus crateronotus Character = 128 Abdala (1999).

Diademodon tetragonus Character = 128 Gaetano et al. (2018).

Trirachodon spp. Character = 128 NMQR 3521.

Luangwa spp. Character = 128 Kemp (1980, fig. 17).

Boreogomphodon jeffersoni Character = 128 Changed from ? to 0.

Lumkuia fuzzi Character = 128 After Hopson and Kitching 2001 scoring.

Traversodon stahleckeri Character = 128 Changred from ? to 0 after von Huene 1936.

Ecteninion lunensis Character = 128 Changed from 0 to ?. No postcranium preserved.

Cricodon metabolus Character = 128 Changed from ? to 0 after UMCZ T905.

129. Expanded costal plates on ribs

0 absent

1 present

Procynosuchus delaharpeae Character = 129 NHMUK PV37054, RC 92 .

Galesaurus planiceps Character = 129 Butler et al (2018, fig. 1).

Thrinaxodon liorhinus Character = 129 Jenkins (1971, fig. 12).

Cynognathus crateronotus Character = 129 Seeley (1895, figs. 17, 18) .

Diademodon tetragonus Character = 129 USNM V23352.

Trirachodon spp. Character = 129 NMQR 3521.

Langbergia modisei Character = 129 NMQR 3281.

Scalenodon spp. Character = 129 Crompton (1955, 653): A few expanded lumbar ribs are preserved and these do not appear to differ from those of *C. metabolus*. Crompton (1995) numerous postcranial fragments coming from site B29. In this site the remians of *S. angustifrons* are much numerous compared with that of other cynodonts also known from that site (Aleodon and three indeterminated mandibles). because of this they are tentatively identified as pertaining to *Scalenodon angustifrons*.

Luangwa spp. Character = 129 NHMUK PV R 36995.

Boreogomphodon jeffersoni Character = 129 Changed from ? to 0.

Traversodon stahleckeri Character = 129 Changred from ? to 1 after von Huene 1936.

Riograndia guaibensis Character = 129 Changed from 0 to ? (no ribs published).

Menadon besairiei Character = 129 Changed from 0/1 to 1.

Trucidocynodon riograndensis Character = 129 Changed from 1 to 0.

Dvinia prima Character = 129 I was unable to check this.

130. Lumbar costal plates with ridge overlapping preceding rib

0 absent

1 present

Procynosuchus delaharpeae Character = 130 NHMUK PV37054, RC 92 .

Galesaurus planiceps Character = 130 Butler et al (2018, figs. 1, 3B).

Thrinaxodon liorhinus Character = 130 Jenkins (1971, fig. 12).

Cynognathus crateronotus Character = 130 Jenkins (1971, fig. 15A).

Diademodon tetragonus Character = 130 USNM V23352.

Trirachodon spp. Character = 130 CGP 1/79 .

Langbergia modisei Character = 130 NMQR 3281.

Luangwa spp. Character = 130 NHMUK PV R 36995.

Aleodon brachyramphus Character = 130 Changed from 0 to ?.

Ecteninion lunensis Character = 130 Changed from 0 to ?. No postcranium preserved.

Tritylodon longaevus Character = 130 Changed from 0 to ?.

Cricodon metabolus Character = 130 Changed from 1 to ?.

Prozostrodon brasiliensis Character = 130 Changed from 0 to ?. This region is preserved.

131. Acromion process

0 absent

1 present

Procynosuchus delaharpeae Character = 131 NHMUK PV37054, Kemp (1980, fig. 7), RC 92.

Galesaurus planiceps Character = 131 Changed from 0 to 1. Butler et al. (2018, 10): In anterior view, the anterior margin forms a noticeable convexity, which is directed laterally where the acromial area is located (Fig. 6B). Butler (2009) described the acromion process in specimen NMQR 135 as a thickening on the averted edge of the scapula that terminates in a small protrusion anteriorly. Jenkins (1971) noted that the acromion process was not developed in *Thrinaxodon liorhinus*, because in all well-preserved specimens the averted edge is very thin and appears complete. However, he did describe a local thickening of the bone edge that could represent the point of acromio-clavicular articulation. We score presence of a proper process because appears well developed in NMQR 860, on of the largest specimen of the taxon.

Thrinaxodon liorhinus Character = 131 Jenkins (1971, Fig. 18). SAM-PK-K1395. However in BP/1/7199 the acromial process seems to be present. Changed from 0 to 1. Tentative score.

Cynognathus crateronotus Character = 131 UCMP 42749.

Diademodon tetragonus Character = 131 Changed from 1 to 0, after Gaetano et al. (2018).

Trirachodon spp. Character = 131 NMQR 3521.

Luangwa spp. Character = 131 NHMUK PV R 36995.

Boreogomphodon jeffersoni Character = 131 Changed from ? to 1.

Lumkuia fuzzi Character = 131 After Hopson and Kitching 2001 scoring.

Traversodon stahleckeri Character = 131 Changred from ? to 1 after von Huene 1936.

Ecteninion lunensis Character = 131 Changed from 1 to ?. No postcranium preserved.

Dvinia prima Character = 131 I was unable to check this.

Cricodon metabolus Character = 131 Changed from ? to 1 after Sidor and Hopson, 2018.

132. Scapular constriction below acromion

0 absent

1 present

Procynosuchus delaharpeae Character = 132 Change score from 0 to 1, after RC 92, that show complete scapula, better preserved than NHMUK PV37054 and in one case it is clear the presence of the constriction following the acromial area of the lamina.

Galesaurus planiceps Character = 132 Changed from 0 to 1 based on NMQR 860.

Thrinaxodon liorhinus Character = 132 BP/1/7199.

Cynognathus crateronotus Character = 132 Changed from 0 to 1 after UCMP 42749.

Diademodon tetragonus Character = 132 Gaetano et al. (2018).

Trirachodon spp. Character = 132 Changed from 0 to 1 after NMQR 3521 (contra Liu and Olsen, 2010).

Luangwa spp. Character = 132 NHMUK PV R 36995.

Andescynodon mendozensis Character = 132 Changed from ? to 0.

Boreogomphodon jeffersoni Character = 132 Changed from ? to 1.

Traversodon stahleckeri Character = 132 Changred from ? to 1 after von Huene 1936.

Ecteninion lunensis Character = 132 Changed from 1 to ?. No postcranium preserved.

Cricodon metabolus Character = 132 Changed from ? to 1 after Sidor and Hopson, 2018.

133. Scapular elongation between acromion and glenoid (This is introduced by Hopson and Kitching (2001, character 87). The elongation is scored as present only in *Morganucodon* spp. and Pachygenelus. It is a clear extension observed in *Morganucodon* spp. (Jenkins and Parrington, 1976, fig. 4).

0 absent

1 present

Procynosuchus delaharpeae Character = 133 NHMUK PV37054, RC 92 .

Pascualgnathus polanski Character = 133 Changed from 0 to 1.

Boreogomphodon jeffersoni Character = 133 Changed from ? to 1.

Lumkuia fuzzi Character = 133 After Hopson and Kitching 2001 scoring.

Traversodon stahleckeri Character = 133 Changed from ? to 0 after von Huene 1936.

Menadon besairiei Character = 133 Changed from ? to 0.

Ecteninion lunensis Character = 133 Changed from 0 to ?. No postcranium preserved.

Pachygenelus monus Character = 133 Changed from 1 to 0.

Cricodon metabolus Character = 133 Changed from ? to 0 after Sidor and Hopson, 2018.

134. Procoracoid in glenoid

0 present

1 barely present or absent

Procynosuchus delaharpeae Character = 134 NHMUK PV37054, RC 92 .

Galesaurus planiceps Character = 134 NMQR 860.

Thrinaxodon liorhinus Character = 134 SAM-PK-K 1395.

Cynognathus crateronotus Character = 134 NHMUK R2571.

Trirachodon spp. Character = 134 NMQR 3521.

Boreogomphodon jeffersoni Character = 134 Changed from ? to 1.

Lumkuia fuzzi Character = 134 After Hopson and Kitching 2001 scoring.

Tritylodon longaevus Character = 134 Changed from ? to 1.

Dvinia prima Character = 134 I was unable to check this.

135. Procoracoid contact with scapula

0 greater than coracoid contact

1 equal to or less than corocoid contact

Procynosuchus delaharpeae Character = 135 NHMUK PV37054.

Galesaurus planiceps Character = 135 Butler et al. (2019, fig. 7E).

Thrinaxodon liorhinus Character = 135 BP/1/7199.

Cynognathus crateronotus Character = 135 UCMP 42749.

Trirachodon spp. Character = 135 NMQR 3521. Coracoid is not visible then this is inferred. Tentative score.

Aleodon brachyramphus Character = 135 Changed from 0 to ?.

Lumkuia fuzzi Character = 135 After Hopson and Kitching 2001 scoring.

Menadon besairiei Character = 135 Changed from ? to 1.

136. Humerus ectepicondylar foramen

0 present

1 absent

Procynosuchus delaharpeae Character = 136 NHMUK PV37054, RC 92 .

Galesaurus planiceps Character = 136 Butler et al. (2018, fig. 9).

Thrinaxodon liorhinus Character = 136 SAM-K-K1395.

Platycraniellus elegans Character = 136 TM 25.

Cynognathus crateronotus Character = 136 Changed from 0 to 0 and 1 after Wynd et al., (2019): As with the humerus described by Abdala (1999), both humeri assigned to NMT RB459 lack an ectepicondylar foramen. This contrasts with the condition figured by Jenkins (1971), where an ectepicondylar foramen is present. Importantly, the humerus described by Abdala (1999) was associated with a skull, whereas the material described by Jenkins (1971) came from a bonebed containing both *Cynognathus crateronotus* and *Diademodon tetragonus* cranial elements, throwing into doubt the taxonomic identification of the postcrania. It is tempting to use the lack of an ectepicondylar foramen as a diagnostic for *Cynognathus crateronotus*, but the Zambian material described below complicates this proposition.

Diademodon tetragonus Character = 136 USNM V23352.

Trirachodon spp. Character = 136 NMQR 3521.

Luangwa spp. Character = 136 NHMUK PV R 36995.

Therioherpeton cargnini Character = 136 Changed from 1 to 0 after Martinelli et al 2016.

Boreogomphodon jeffersoni Character = 136 Changed from ? to 1.

Traversodon stahleckeri Character = 136 Changred from ? to 1 after von Huene 1936.

Brasilodon tetragonus Character = 136 Changed from 1 to 0.

Riograndia guaibensis Character = 136 Changed from 0 to ? (no humerus published).

Ecteninion lunensis Character = 136 Changed from 0 to ?. No postcranium preserved.

Tritylodon longaevus Character = 136 Changed from ? to 1.

Sinoconodon rigneyi Character = 136 Changed from 1 to ?.

137. Ulna olecranon process

0 absent unossified

1 present

Procynosuchus delaharpeae Character = 137 There is no olecranon en NHMUK PV37054 but it seems to be present in RC 92, altough clearly as not developed as for example in *Oligokyphus* spp. Scored as 0 by Hopson and Kitching (2001) and Liu and Olson (2010). There is a third specimen with ulna at the BP collection. Need to check.

Galesaurus planiceps Character = 137 Butler et al. (2019, 12).

Thrinaxodon liorhinus Character = 137 SAM-K-K1395.

Cynognathus crateronotus Character = 137 BP/1/1675 (Jenkins, 1971, fig. 31). Tentative score identified as ?*Cynognathus crateronotus* (?*Diademodon*) by Jenkins (1971).

Diademodon tetragonus Character = 137 USNM 23352.

Trirachodon spp. Character = 137 NMQR 3521.

Luangwa spp. Character = 137 NHMUK PV R 36995.

Andescynodon mendozensis Character = 137 Changed from ? to 0.

Boreogomphodon jeffersoni Character = 137 Changed from ? to 0.

Trucidocynodon riograndensis Character = 137 Changed from 1 to 0.

Tritylodon longaevus Character = 137 Changed from ? to 1.

Cricodon metabolus Character = 137 Changed from ? to 1 after *C. metabolus* by Sidor and Hopson, 2018.

138. Manual digit III phalanx number

0 four

1 three

Procynosuchus delaharpeae Character = 138 RC 92 .

Galesaurus planiceps Character = 138 Butler et al. (2019, 14): The inferred phalangeal formula for SAM-PK-K10465 (lacking disc-like phalanges) is ?2-3-3-3-?3. In contrast, the presence of a disc-like phalanx in digit IV of NMQR 3716 followed by a nonungueal phalanx indicates the putative presence of four phalanges in the digit. Finally, even when there is a disc-like phalanx in digit III of SAM-PK-K10468, the evidence shows that there were only three phalanges in the digit.

Cynognathus crateronotus Character = 138 There is not currently known articulated manus of *Cynognathus crateronotus*. Circumstancial evidence proposed by Jenkins (1971, 129) is the absence of disc-like phalanges in the extensive sample of associated postcranial elements that he described as ?*Cynognathus crateronotus* (?*Diademodon*). This is accepted here. Tentative score.

Diademodon tetragonus Character = 138 USNM 23352.

Trirachodon spp. Character = 138 Changed from 1 to ? No articulated manus of *Trirachodon* spp. is known.

Aleodon brachyramphus Character = 138 Changed from ? to 1. No discoidal phalanges. I am not sure about the asignation of the specimen.

Trucidocynodon riograndensis Character = 138 Changed from ? to 1.

Cricodon metabolus Character = 138 Changed from ? to 1 after UMCZ T905.

139. Manual digit IV phalanx number

0 five

1 four

2 three

Procynosuchus delaharpeae Character = 139 RC 92.

Galesaurus planiceps Character = 139 Butler et al. (2019, 14): The inferred phalangeal formula for SAM-PK-K10465 (lacking disc-like phalanges) is ?2-3-3-3-?3. In contrast, the presence of a disc-like phalanx in digit IV of NMQR 3716 followed by a nonungueal phalanx indicates the putative presence of four phalanges in the digit. Finally, even when there is a disc-like phalanx in digit III of SAM-PK-K10468, the evidence shows that there were only three phalanges in the digit.

Cynognathus crateronotus Character = 139 There is not currently known articulated manus of *Cynognathus crateronotus*. Circumstancial evidence proposed by Jenkins (1971, 129) is the absence of disc-like phalanges in the extensive sample of associated postcranial elements that he described as ?*Cynognathus crateronotus* (?*Diademodon*). This is accepted here. Tentative score.

Diademodon tetragonus Character = 139 There is not currently known articulated fourth digit of *Diademodon*. Circumstancial evidence proposed by Jenkins (1971, 129) is the absence of disc-like phalanges in the extensive sample of associated postcranial elements that he described as ?*Cynognathus crateronotus* (?*Diademodon*). This is accepted here. Tentative score.

Trirachodon spp. Character = 139 Changed from 1 to ? No articulated manus of *Trirachodon* spp. is known.

Aleodon brachyramphus Character = 139 Changed from ? to 2. No discoidal phalanges. I am not sure about the assignation of the specimen.

Trucidocynodon riograndensis Character = 139 Changed from ? to 2.

Cricodon metabolus Character = 139 Probably 3 phalanges in digit IV according to UMCZ T905. Changed from ? to 2.

140. Lateral surface of iliac blade

0 concave or nearly flat

1 convex

2 divided by longitudinal ridge into dorsal and ventral portions

Procynosuchus delaharpeae Character = 140 NHMUK PV37054, RC 92 .

Galesaurus planiceps Character = 140 Butler et al. (2019, fig. 12A, 12B).

Thrinaxodon liorhinus Character = 140 BP/1/5208.

Cynognathus crateronotus Character = 140 NHMUK R2571.

Diademodon tetragonus Character = 140 USNM 23352.

Trirachodon spp. Character = 140 NMQR 3521.

Langbergia modisei Character = 140 NMQR 3281.

Luangwa spp. Character = 140 NHMUK PV R 36995.

Aleodon brachyramphus Character = 140 I am not sure about the asignation of the specimen.

Ecteninion lunensis Character = 140 Changed from 0to ?. No postcranium preserved.

Pachygenelus monus Character = 140 I was unable to check this scoring.

Cricodon metabolus Character = 140 After NHCC LB28.

141. Length of anterior process of ilium anterior to acetabulum relative to diameter of acetabulum

0 less than 1.0

1 1.0 to 1.5

2 greater than 1.5

Procynosuchus delaharpeae Character = 141 NHMUK PV37054, RC 92 .

Galesaurus planiceps Character = 141 Butler et al. (2019, fig. 12B).

Thrinaxodon liorhinus Character = 141 BP/1/5208. BP/1/7199.

Cynognathus crateronotus Character = 141 NHMUK R2571.

Diademodon tetragonus Character = 141 USNM 23352.

Trirachodon spp. Character = 141 NMQR 3521.

Langbergia modisei Character = 141 NMQR 3281.

Scalenodon spp. Character = 141 Changed from 1 to ? No preserved ilium known.

Luangwa spp. Character = 141 NHMUK PV R 36995.

Andescynodon mendozensis Character = 141 Changed from ? to 2.

Aleodon brachyramphus Character = 141 Changed from ? to 1. I am not sure about the asignation of the specimen.

Menadon besairiei Character = 141 Changed from ? to 2.

Pachygenelus monus Character = 141 I was unable to check this scoring.

Cricodon metabolus Character = 141 Changed from ? to 1 after C. metabolus NHCC LB28.

142. Posterior iliac spine

0 robust and extends beyond acetabulum

1 small nub that lies entirely anterior to acetabulum

Procynosuchus delaharpeae Character = 142 NHMUK PV37054, RC 92 .

Galesaurus planiceps Character = 142 Butler et al. (2019, fig. 12B).

Thrinaxodon liorhinus Character = 142 BP/1/5208. BP/1/7199.

Cynognathus crateronotus Character = 142 NHMUK R2571.

Diademodon tetragonus Character = 142 USNM 23352.

Trirachodon spp. Character = 142 NMQR 3521.

Langbergia modisei Character = 142 NMQR 3281.

Luangwa spp. Character = 142 NHMUK PV R 36995.

Aleodon brachyramphus Character = 142 I am not sure about the asignation of the specimen.

Pachygenelus monus Character = 142 I was unable to check this scoring.

Cricodon metabolus Character = 142 After NHCC LB28.

143. Dorsal profile of ilium

0 strongly convex

1 flat to concave

Procynosuchus delaharpeae Character = 143 NHMUK PV37054, RC 92 .

Galesaurus planiceps Character = 143 Butler et al. (2019, fig. 12B.

Thrinaxodon liorhinus Character = 143 BP/1/5208. BP/1/7199.

Cynognathus crateronotus Character = 143 NHMUK R2571.

Diademodon tetragonus Character = 143 USNM 23352.

Trirachodon spp. Character = 143 Changed from ? to 0 after NMQR 3521.

Langbergia modisei Character = 143 NMQR 3281.

Luangwa spp. Character = 143 NHMUK PV R 36995.

Andescynodon mendozensis Character = 143 Variable sensu Liu and Powell 2009. Changed from ? to 0/1.

Aleodon brachyramphus Character = 143 Changed from ? to 1. I am not sure about the asignation of the specimen.

Pachygenelus monus Character = 143 I was unable to check this scoring.

Cricodon metabolus Character = 143 Changed from ? to 0 after C. metabolus NHCC LB28.

144. Cotyloid acetabular notch

0 lies between ischial and iliac parts of acetabulum but mainly on ilium

1 lies entirely on ischium between acetabular facet and pubic process

Procynosuchus delaharpeae Character = 144 NHMUK PV37054.

Galesaurus planiceps Character = 144 Butler et al. (2019, fig. 12B).

Thrinaxodon liorhinus Character = 144 BP/1/5208. BP/1/7199.

Cynognathus crateronotus Character = 144 NHMUK R2571.

Diademodon tetragonus Character = 144 USNM 23352.

Trirachodon spp. Character = 144 NMQR 3521.

Langbergia modisei Character = 144 Changed from ? to 0 based on NMQR 3281.

Luangwa spp. Character = 144 NHMUK PV R 36995.

Aleodon brachyramphus Character = 144 I am not sure about the asignation of the specimen.

Lumkuia fuzzi Character = 144 Changed from 0 to ?.

Kayentatherium wellesi Character = 144 Changed from ? to 1.

Pachygenelus monus Character = 144 I was unable to check this scoring.

Cricodon metabolus Character = 144 Changed from ? to 0.

145. Diameter of obturator foramen (Character introduced by Rowe (1988, character 139). The foramen is not always rounded, then it would be worth to consider the anteroposterior length of the foramen, in relation to the anteroposterior length of the acetabulum. For example the dorsoventral length of the obturator foramen in *Thrinaxodon liorhinus* seems to be much longer than the anteroposterior length (BP/1/7199).)

0 less than or equal to that of acetabulum

1 greater than that of acetabulum

Procynosuchus delaharpeae Character = 145 NHMUK PV37054, RC 92 .

Galesaurus planiceps Character = 145 Butler et al. (2018, fig. 12B, 12C).

Thrinaxodon liorhinus Character = 145 AMMM 5265. BP/1/7199.

Cynognathus crateronotus Character = 145 NHMUK R2571.

Diademodon tetragonus Character = 145 USNM V23352.

Trirachodon spp. Character = 145 Changed from 0 to ? The foramen is not visible in NMQR 3521.

Langbergia modisei Character = 145 NMQR 3281. Tentative score.

Luangwa spp. Character = 145 NHMUK PV R 36995.

Aleodon brachyramphus Character = 145 I am not sure about the asignation of the specimen.

Brasilodon tetragonus Character = 145 Changed from ? to 1 (Agustin).

Pachygenelus monus Character = 145 I was unable to check this scoring.

Prozostrodon brasiliensis Character = 145 Changed from 1 to ?. Not preserved.

146. Total length of pubis relative to acetabulum diameter (Proposed originally by Hopson and Kitching (2001, character 97). It is comparing a diameter with a length, then it should be changed to maximum length of acetabulum.)

0 greater than 1.5

1 between 1.5 and 1.0

2 less than 1.0

Procynosuchus delaharpeae Character = 146 RC 92: 1.29 (measured after a photo of the right side, using digimizer).

Galesaurus planiceps Character = 146 Butler et al. (2018, fig. 12B, 12C).

Thrinaxodon liorhinus Character = 146 AMMM 5265.

Cynognathus crateronotus Character = 146 NHMUK R2571.

Diademodon tetragonus Character = 146 Changed from 2 to 1. In USNM V23352 the maximum length of the pubis is as least as long as the acetabular length.

Scalenodon spp. Character = 146 Changed from 2 to ? This score is probably based in NHMUK R9391 (Jenkins, 1971, fig. 46) which is not associated with skull material ans is identified as ?Aleodon or ?*Scalenodon* spp. by Jenkins (1971). In addition their compared dimensions are subequals.

Luangwa spp. Character = 146 The preservation of the pubis in NHMUK PV R 36995 is incomplete, but it could be inferred that iys size was smaller than the length of the acetabulum.

Pascualgnathus polanski Character = 146 Changed from 2 to 0.

Andescynodon mendozensis Character = 146 Changed from ? to 0.

Aleodon brachyramphus Character = 146 Changed from ? to 1. I am not sure about the asignation of the specimen.

Aleodon cromptoni Character = 146 Chnaged from 2 to ?.

Exaeretodon spp. Character = 146 I think this should be chaged to 1 (between 1.5 and 1.0) according to Bonaparte (1963) drawing.

Trucidocynodon riograndensis Character = 146 Changed from ? to 1.

Chiniquodon spp. Character = 146 I am not sure about this scoring. .

Pachygenelus monus Character = 146 I was unable to check this scoring.

147. Head of femur (Character introduced by Liu and Olsen (2010). They scored as subspherical and inflected dorsally in tritheledontids, tritylodontids and *Morganucodon* spp.. Comparison of the heads of *Kayentatherium wellesi* (Sues and Jenkins, 2006) and ?*Cynognathus crateronotus* (?*Diademodon*) does not look very different. Perhaps a little more protrusion in *Kayentatherium wellesi*.)

0 rounded and predominantly in plane of shaft

1 subspherical and inflected dorsally

Charassognathus gracilis Character = 147 Changed from 1 to 0. From paper Botha et al 2007.

Procynosuchus delaharpeae Character = 147 The head in NHMUK PV37054 and RC 92 is just slightly directed dorsally.

Galesaurus planiceps Character = 147 Butler et al. (2018, fig. 13).

Thrinaxodon liorhinus Character = 147 SAM-K-K1395.

Traversodon stahleckeri Character = 147 Changred from ? to 0 after von Huene 1936.

Pachygenelus monus Character = 147 I was unable to check this scoring.

Cricodon metabolus Character = 147 After UMCZ T905 and NHCC LB28.

Prozostrodon brasiliensis Character = 147 Changed from 0 to ?. Not preserved.

148. Greater trochanter separated from femoral head by distinct notch

0 absent

1 present

Procynosuchus delaharpeae Character = 148 NHMUK PV37054.

Galesaurus planiceps Character = 148 Butler et al. (2018, fig. 13D).

Thrinaxodon liorhinus Character = 148 SAM-PK-K 1395.

Cynognathus crateronotus Character = 148 NHMUK R2571.

Diademodon tetragonus Character = 148 USNM V23352.

Trirachodon spp. Character = 148 NMQR 3521.

Luangwa spp. Character = 148 NHMUK PV R 36995.

Andescynodon mendozensis Character = 148 Changed from ? to 1.

Boreogomphodon jeffersoni Character = 148 Changed from ? to 1.

Traversodon stahleckeri Character = 148 Changred from ? to 0 after von Huene 1936.

Brasilodon tetragonus Character = 148 Changed from ? to 1 (Agustin).

Pachygenelus monus Character = 148 I was unable to check this scoring.

Sinoconodon rigneyi Character = 148 Changed from 1 to ? as there are no unambiguously asigned femora to the genus.

Cricodon metabolus Character = 148 Changed from ? to 0 after C. metabolus NHCC LB28.

Prozostrodon brasiliensis Character = 148 Changed from 0 to ?. Not preserved.

149. Greater trochanter joined to femoral head by ridge (This character was introduced by Hopson and Kitching (2001, character 99). We fail to identify a ridge uniting this structures in the nicely preserved femur of *Luangwa* spp. in which Hopson and Kitching (2001) score this as present. In fact this character for Hopson and Kitching (2001) is an autapomorphy of *Morganucodon* spp. Considering Jenkins and Parrington (1976) figures of the femur of *Morganucodon* spp. it is more likely a ridge in this bone than in those of other non-mammaliaform cynodonts. Perhaps they confused the character states?)

0 present

1 absent

Procynosuchus delaharpeae Character = 149 NHMUK PV37054.

Luangwa spp. Character = 149 NHMUK PV R 36995.

Andescynodon mendozensis Character = 149 Changed from ? to 1.

Boreogomphodon jeffersoni Character = 149 Changed from ? to 0.

Traversodon stahleckeri Character = 149 Changed from ? to 0 after von Huene 1936.

Brasilodon tetragonus Character = 149 Changed from ? to 0 (Agustin).

Tritylodon longaevus Character = 149 Changed from ? to 1.

Bienotherium spp. Character = 149 Changed from ? to 1.

Pachygenelus monus Character = 149 I was unable to check this scoring.

Cricodon metabolus Character = 149 Changed from ? to 0 after C. metabolus NHCC LB28.

Irajatherium hernandezi Character = 149 The greater trochanter is continuous with the head.

150. Lesser trochanter position (This is a clear difference between the bones of most non mammaliamorph cynodonts and those of tritylodontids and mammaliaformes in which the lesser trochanter is clearly located medial to the head as a much independent feature of the diaphysis.)

0 on ventromedial surface of femoral shaft

1 on medial surface of femoral shaft

Procynosuchus delaharpeae Character = 150 NHMUK PV37054. Kemp (1980, fig. 14, tr.int). We interpret the trochanter internus of Kemp (1980) as the lesser trochanter.

Galesaurus planiceps Character = 150 Butler et al. (2018, fig. 13D).

Thrinaxodon liorhinus Character = 150 Jenkins (1971, fig. 49). SAM-PK-K 1395.

Cynognathus crateronotus Character = 150 Wynd et al. (2018, fig. 4D).

Diademodon tetragonus Character = 150 USNM V23352.

Trirachodon spp. Character = 150 NMQR 3521.

Luangwa spp. Character = 150 NHMUK PV R 36995 .

Andescynodon mendozensis Character = 150 Changed from ? to 1.

Boreogomphodon jeffersoni Character = 150 Changed from ? to 0.

Traversodon stahleckeri Character = 150 Changred from ? to 0 after von Huene 1936.

Brasilodon tetragonus Character = 150 Chnaged from ? to 1.

Pachygenelus monus Character = 150 I was unable to check this scoring.

Sinoconodon rigneyi Character = 150 Changed from 1 to ? as there are no unambiguously asigned femora to the genus.

Cricodon metabolus Character = 150 Changed from ? to 0 after C. metabolus NHCC LB28.

Prozostrodon brasiliensis Character = 150 Changed from 0 to ?. Not preserved.

151. Lesser trochanter

0 far distally from femoral head

1 near level of femoral head

Procynosuchus delaharpeae Character = 151 NHMUK PV37054.

Galesaurus planiceps Character = 151 Butler et al. (2018, fig. 13E).

Aleodon cromptoni Character = 151 Chnaged from ? to 0.

Traversodon stahleckeri Character = 151 Changred from ? to 0 after von Huene 1936.

Pachygenelus monus Character = 151 I was unable to check this scoring.

Dvinia prima Character = 151 Changed from ? to 1.

Cricodon metabolus Character = 151 After UMCZ T905 and NHCC LB28.

Prozostrodon brasiliensis Character = 151 Changed from 0 to ?. Not preserved.

Character = 34 STATE = 4 Proposed by Luo and Crompton (1994, character 14), considering contact area or else projection of the quadrate. Both formulation and scoring is based in Luo and Crompton (1994).

Character = 70 STATE = 1 This character is about the curved vertical component of the lateral flange. A structure that is only known in tritylodontids and *Sinoconodon rigneyi*. Character proposed by Luo (1994): 52. Lateral flange of the petrosal (prootic) forming lateral shelf perpendicular to anterior lamina (0). lateral flange forming broad shelf with vertical component (L-shaped) anterior to pterygoparoccipital foramen (1). lateral shelf reduced (2). This was modified by Luo Crompton and Sun (2001) to character 25: vertical component flange (L-shaped and forming a vertical wall to the pterygoparaoccipital

foramen): present (0). absent (1). Reworded by Liu and Olsen (2010), character 59 as used here.

Character = 71 STATE = 1 Original formulation by Luo (1994): 47. Anterior paroccipital process: laterally covered by squamosal (0). bulbous, without squamosal cover (1). crest of crista parotica and fossa incudis differentiated without squamosal cover (2). Score 1 is only for tritylodontids. score 0 for all NMC plus *Sinoconodon rigneyi* and Adelobasileus,. score 2 is for *Morganucodon* spp.. Character modified by Luo, et al. (2001) as follow: 22. Relationship of the squamosal to the anterior part of paroccipital process (Modified from Luo, 1989. Rougier et al., 1996a: ch 23): (a) Squamosal covers the lateral aspect of the anterior paroccipital process: *Probainognathus jenseni* (out-group), tritheledontids, Adelobasileus, *Sinoconodon rigneyi*, *Zhangheotherium, Vincelestes,* metatherians, eutherians. (b) Squamosal is dorsally withdrawn from the paroccipital region so the anterior part of paroccipital is exposed: tritylodontids, *Morganucodon* spp., *Haldanodon, Hadrocodium,* triconodontines, *Ornithorhynchus*, multituberculates. Liu and Olsen (2010, character 60) state the character as here.

Character = 92 STATE = 0 This is character 3 of Martinez et al. (1996) and character 81 of Abdala (2007). The state 1 is scored for tritylodontid and tritheledontid (tentatively in Pachygenelus). The state 1 is more likely after Hopson (1991, 677): the following are tritylodontid-tritheledontid characters not found in mammals: 1) the first lower incisor enlarged., 2) the tip of the lower incisor fitting into the gap between laterally placed upper incisors?.
